# Genome-Wide Identification and Expression Profiling Analysis of the Xyloglucan Endotransglucosylase/Hydrolase Gene Family in Tobacco (*Nicotiana tabacum* L.)

**DOI:** 10.3390/genes9060273

**Published:** 2018-05-24

**Authors:** Meng Wang, Zongchang Xu, Anming Ding, Yingzhen Kong

**Affiliations:** 1 Key Laboratory for Tobacco Gene Resources, Tobacco Research Institute, Chinese Academy of Agricultural Sciences, Qingdao 266101, China; menglihua@yeah.net (M.W.); xuzc1110@163.com (Z.X.); dinganming@caas.cn (A.D.); 2 Graduate School of Chinese Academy of Agricultural Science, Beijing 100081, China

**Keywords:** xyloglucan endotransglucosylase/hydrolases, phylogenetic, gene expression, abiotic stress, *Nicotiana tabacum*

## Abstract

Xyloglucan endotransglucosylase/hydrolase genes (*XTHs*) encode enzymes required for the reconstruction and modification of xyloglucan backbones, which will result in changes of cell wall extensibility during growth. A total of 56 *NtXTH* genes were identified from common tobacco, and 50 cDNA fragments were verified by PCR amplification. The 56 *NtXTH* genes could be classified into two subfamilies: Group I/II and Group III according to their phylogenetic relationships. The gene structure, chromosomal localization, conserved protein domains prediction, sub-cellular localization of NtXTH proteins and evolutionary relationships among *Nicotiana tabacum*, *Nicotiana sylvestrisis*, *Nicotiana tomentosiformis*, *Arabidopsis*, and rice were also analyzed. The *NtXTHs* expression profiles analyzed by the TobEA database and qRT-PCR revealed that *NtXTHs* display different expression patterns in different tissues. Notably, the expression patterns of 12 *NtXTHs* responding to environment stresses, including salinity, alkali, heat, chilling, and plant hormones, including IAA and brassinolide, were characterized. All the results would be useful for the function study of *NtXTHs* during different growth cycles and stresses.

## 1. Introduction

Xyloglucan is the most abundant hemicellulose in the primary cell walls of dicotyledonous and nongraminaceous monocots plants, where it coats and cross-links adjacent cellulose microfibrils through a non-covalent bond to form a dynamic cellulose-xyloglucan load-bearing cell wall framework [1,2]. In addition, the use of xyloglucan acts as a storage reserve in the seeds of some plant species, such as nasturtium (*Tropaeolum majus*), where it accumulates as large deposits on the inside of the cotyledon cell wall during seed development and is subsequently hydrolyzed during germination [3].

The xyloglucan endotransglucosylase/hydrolases (XTH) gene family is a subfamily of GH16 based on the carbohydrate-active enzymes (CAZy) classification [4]. Multigene families of XTHs have been identified in a wide variety of plant species using genome sequencing, including *Arabidopsis* (33), rice (*Oryza sativa*, 29), poplar (*Populus* spp. L., 41), wheat (*Triticum aestivum* L., >57), sorghum (*Sorghum bicolor*, 35), tomato (*Solanum lycopersicum*, 25), kiwifruit (*Actinidia deliciosa*, 14), and apple (*Malus sieversii*, 11) [2,5,6,7,8,9,10,11]. Thirty-three XTH proteins encoded by the *Arabidopsis* XTH gene family were clustered into three main phylogenetic groups, Group I, Group II, and Group III, based on the similarity of deduced amino acid sequences using CLUSTALW software [10]. However, *XTH* gene members in rice have been classified into only two subfamilies, Group I/II and Group III, based on the classification for *Arabidopsis XTH* genes [11]. In other species, *XTH* genes clustered into two or three phylogenetic groups, which were consistent with rice or *Arabidopsis* [5,6]. The enzymes encoded by the XTH gene family are mainly responsible for the cleavage and/or rearrangement of xyloglucan backbones in plants. The XTH proteins have two distinct catalytic activities; xyloglucan endo-transglucosylase (XET activity, EC 2.4.1.207) and xyloglucan endo-hydrolase (XEH activity, EC 3.2.151). The XET domain cleaves a xyloglucan chain and rejoins the reducing end to another xyloglucan molecule, resulting in the elongation of xyloglucan. The XEH domain rejoins the xyloglucan reducing end to a water molecule resulting in irreversible xyloglucan chain shortening [12]. Hence XTHs are considered key enzymes in the regulation of cell wall extensibility during cell growth [13], resulting in primary root elongation [14], hypocotyl growth [15], vein differentiation [16], flower opening [17], petal abscission [18], and wood formation [19]. In addition, XTH affected fruit softening such as tomato, kiwi fruit, pear, litchi, or grape berry [8]. All of the 10 different *SlXTHs* were expressed at the red ripe stage, and both XET and XEH activities were higher during fruit growth and decreased during fruit ripening [8]. Additionally, previous expression profiling studies have shown that *XTHs* were the most highly expressed cell wall enzymes of CAZymes in poplar [6]. Overexpression of the chili pepper (*Capsicum annum*) *XTH* gene, *CaXTH3*, in *Arabidopsis* resulted in severely wrinkled leaf morphology due to increased numbers of small cells [20]. A transgenic cotton plant that over-expressed *GhXTH1* produced mature cotton fibers that were between 15% and 20% longer than those produced by wild-type cotton plants under both greenhouse and field growth conditions [21]. These previous studies indicated that *XTHs* play essential roles in many growth and differentiation processes.

In addition, numerous studies have shown that the expression of *XTH* genes are also regulated by plant hormones and abiotic stresses. *Arabidopsis XTH3* -*4*, -*17*, -*22*, and -*23* were found to be significantly induced by auxin and brassinolide [10,22]. *AtXTH23* was up-regulated by gibberellin and abscisic acid. Similarly, three *CaXTHs* were effectively up-regulated by ethylene in leaf tissue [20]. In contrast, the expression of *AtXTHs* could also be repressed by plant hormones. Transcripts of *AtXTH15* and *AtXTH21* were markedly down-regulated by auxin, whereas gibberellic acid could significantly repress the expression of *AtXTH26* [10]. Transgenic *Arabidopsis* and tomato plants over-expressing *CaXTH3* from pepper showed high resistance to drought and salinity. In addition, some other *CaXTHs* were significantly induced by a broad spectrum of abiotic stresses, including cold temperature, drought, and high salinity [20,23]. A previous proteomic analysis in maize also showed that *XTHs* were differentially regulated under drought stress [24]. Previous studies have reported that some Chinese cabbage (*Brassica rapa* L.) *XTH* genes were up-regulated under heat stress [25], and the rice *OsXET9* gene was highly induced under cold stress [26], suggesting that *XTH* genes could also be altered in response to heat or cold stresses. These studies highlighted the key role of *XTHs* in abiotic stress tolerance.

Tobacco is one of the most important economic crops in the world and a perfect model plant for use in scientific research. Although *XTH* genes have multiple functions during plant growth and development, only four tobacco *XTH* transcripts have been cloned [27,28]. Following the recent publication of the tobacco (*Nicotiana tabacum* L.) genome sequence [29], the availability of this resource, together with the *Arabidopsis* genome database, offers the chance to undertake comparative phylogenetic and expression analyses of the whole complement of the *XTH* gene family. Microarray data is a useful tool for analyzing gene expression and location. TobEA is a custom-designed Affymetrix tobacco expression microarray generated from over 40,000 unigenes and used to measure gene expression in 19 different tissues throughout the tobacco lifecycle [30]. This tool will facilitate the study of tobacco gene expression. In this study, we report the identification of 56 *NtXTH* genes from the tobacco genome; the results of routine bioinformatics analysis showing gene structure, phylogenetic tree, and chromosomal location are presented. Additionally, this gene family is analyzed with a focus on the evolution and divergence after multiple duplications in relation to tobacco genome fusion. To provide useful information for the further functional study of *XTH* genes in *N. tabacum*, the expression patterns of *XTH* genes in different tobacco tissues, as well as in response to stresses such as plant hormones, salinity, alkali, heat, and cold, were characterized by qRT-PCR analysis. We further isolated 50 *NtXTH* cDNA fragments by PCR amplification. To provide more information for the functional characterization of *NtXTHs*, the sub-cellular localization of four *NtXTHs* was identified as well.

## 2. Materials and Methods

### 2.1. Identification of Xyloglucan Endo-Transglucosylase/Hydrolase Family Members in Tobacco and Other Species

Two methods were used to identify tobacco XTH proteins in this study. The first method was based on a previous study [31]. All annotated proteins of the tobacco variety K326 from the Solanaceae crops genome database (SOL Genomics Network, SGN, https://solgenomics.net/organism/Nicotiana_tabacum/genome) were considered. We employed the Hidden Markov Model (HMM) profile of the XTH protein domains PF00722 and PF06955 [32] as queries to search the database using the program HMMER3.0 with the default E-value. The online program SMART (http://smart.embl-heidelberg.de/) was used to assess the conserved domain of candidate tobacco XTHs with an *E*-value < 0.1. Only proteins that contain both PF00722 and PF06955 domains were regarded as NtXTHs and reserved for further analysis. The second method was based on the blast of homologous proteins. Thirty-three published *Arabidopsis thaliana* XTH protein sequences acquired from TAIR 10 (http://www.arabidopsis.org) were used as queries to blast the Solanaceae crops genome database in order to identify tobacco homologous XTH proteins with the parameters *E*-value < 10^−15^ and id% > 50%. After manually removing the redundant sequences, candidate XTH protein sequences with conserved PF00722 and PF06955 domains were filtered by the SMART tool. Finally, tobacco XTH proteins were identified based in the two methods described above. Information regarding coding sequence (CDS) sequences, genomic sequences, and chromosome locations of *NtXTHs* was obtained from the SGN database. The names of tobacco *XTH* genes were nominated according to a previous study [12]. Physicochemical parameters of each gene were calculated using the ProtParam tool (http://web.expasy.org/protparam/). The signal peptide cleavage sites were predicted with SignalP v4.1 server (http://www.cbs.dtu.dk/services/SignalP/). The sub-cellular localizations were predicted with ProtComp 9.0 (http://linux1.softberry.com).

The protein sequences of XTH genes from *Nicotiana tomentosiformis* and *Nicotiana sylvestris*, which are considered to be the paternal and maternal donors of *N. tabacum* [33], were also downloaded from the SGN database to investigate the evolutionary relationships of the XTH gene family in ancestor species and common tobacco. In addition, unique members of the XTH gene family in tomato, potato, pepper, eggplant, petunia, and coffee were also downloaded from the SGN database.

### 2.2. Phylogenetic Analysis

Multiple sequence alignments and phylogenetic tree construction of the full-length XTH protein sequences at amino acid level were performed using ClustalW and MEGA 6.0 software [34], respectively. Detailed parameters have been described previously [35]; the gap extension penalty 0.2 was replaced with 0.1.

### 2.3. Gene Structure Analysis and Weblogo of Conserved Catalytic Site

The exon/intron organization of the *NtXTH* genes was identified with the Gene Structure Display Server (GSDS) tool (http://gsds.cbi.pku.edu.cn/) [36] by aligning the cDNA sequences with the corresponding genomic DNA sequences. The weblogo of conserved catalytic domains was illustrated using the MEME tool (http://meme-suite.org/tools/meme).

### 2.4. Chromosomal Location and Gene Duplication

The chromosomal locations of *NtXTHs* were determined based on the chromosomal information derived from the SGN database. The positions of the *NtXTH* genes were physically mapped to each chromosome according to their coordinates on the tobacco genome. Tandem duplications were defined as adjacent *NtXTH* genes separated by five or fewer genes in a 100 kb region within an individual chromosome [37].

### 2.5. Ka/Ks Values Estimation

The Ka/Ks value is the ratio between the number of nonsynonymous substitutions per nonsynonymous site (Ka) and the number of synonymous substitution per synonymous site (Ks). To estimate the type of selection *NtXTH* genes are under, the ratio of the rates of nonsynonymous to synonymous substitutions (Ka/Ks) of all sister pairs was calculated for each terminal branch of the phylogenetic trees of *N. tabacum* using DnaSPv5 software [38]. To confirm the selection pressure, a Ka/Ks ratio greater than 1, less than 1, and equal to 1 represented positive selection, negative selection, and neutral selection, respectively [39]. For each gene pair, the Ks value was used to estimate the divergence time in millions of years based on a rate of 6.1 × 10^−9^ substitutions per site per year, and the divergence time (*T*) was calculated as *T* = Ks/(2 × 6.1 × 10^−9^) × 10^−6^ million years ago (Mya) [40].

### 2.6. Microarray Expression Profiles of NtXTHs

The 56 *NtXTHs* sequences identified were used as queries to blast against the tobacco SGN Unigene databases to find out and identify the corresponding Unigene IDs in the TobEA microarray database [30] in order to analyze the expression profiles of *NtXTHs* in different tobacco tissues. Then, expression data for *NtXTHs* in 19 tobacco tissues were extracted from TobEA and normalized by log_10_ (FPKM+1). A hierarchical map was constructed and viewed with the software Mev [41] based on the normalization data.

### 2.7. Plant Materials, Growth Conditions, and Stress Treatments

Seedlings of *N. tabacum* species K326 were used to study gene expression in all experiments. To analyze the expression of tobacco *XTH* genes under abiotic stresses, 4-week-old seedlings were grown in a culturing room at 23 ± 1 °C under a 16-h light/8-h dark cycle, with relative humidity controlled at approximately 60%. Solutions of 150 mM NaCl [42], 50 mM Na_2_CO_3_, 1 µM 2, 4-epi-brassinolide (epiBL, Solarbio, B8780, Beijing, China), and 1 µM 3-Indole acetic acid (IAA, Solarbio, I8020, Beijing, China) [10] were used to water the tobacco plants. Leaves of stress-treated plants were collected at 0.5, 2, and 6 h after treatment initiation. Leaves that were collected at 0.5, 2, and 6 h intervals from tobacco plants that were only fed water were used as the corresponding control. To induce heat and chilling stress, tobacco seedlings were placed in a constant temperature incubator or a refrigerator at 37 °C [25] and 4 °C, respectively. Leaves of stress-treated plants were collected at 0.5, 2, and 6, h after treatment initiation. Non-treated seedlings were used as a control for heat and chilling stress treatments. After the materials were collected, they were immediately frozen in liquid nitrogen and stored at −80 °C for RNA extraction. Three biological replicates were employed per sample. The root, stalk, leaves, and flower tissues of the adult plants grown in the culturing room were also collected and stored at −80 °C and subsequently used for the amplification of *NtXTH* cDNA fragments.

### 2.8. RNA Extraction and qRT-PCR Analysis

Total RNA was extracted with the EasyPure Plant RNA Kit (TransGen, ER301-01, Beijing, China) and treated with RNase-free DNase I (TransGen, K21109, Beijing, China). First-strand cDNA synthesis and qRT-PCR amplification were performed as previously described [43]. Expression was calculated using the 2^−∆∆Ct^ method [44] and normalized to that of the *NtACTIN* gene (XM_019370655.1). Because members of the *NtXTH* gene family share homology in their coding regions, gene-specific primers based on the 3′ non-coding regions of *NtXTHs* were designed for qRT-PCR using Oligo Calc (http://biotools.nubic.northwestern.edu/OligoCalc.html) to avoid non-specific amplification. The qRT-PCR primers used in this study are shown in Supplementary File 1.

### 2.9. Amplification and Sequencing of NtXTH cDNAs

A mixture of cDNA extracted from root, leaf, stem, and flower tissues of K326 species was used as an amplification template. Gene-specific primers to amplify each of the *NtXTHs* were designed using the Oligo Calc software. Forward/reverse primers were designed in the nearest upstream/downstream regions close to the start/stop codon of the CDS. To obtain the correct cDNA sequences, the super-high fidelity *TranStart* FastPfu Fly DNA polymerase (Transgen, AP231-13, Beijing, China) was used to amplify the *NtXTHs*. PCR products that generated bright and single bands, or multiple bands that could be easily separated, were sequenced directly. Other PCR products with multiple bands that did not separate well were cloned into the pEasy-Blunt vector (Transgen, CB101-02, Beijng, China). After transformation into *Escherichia coli*, the positive colonies were sequenced to obtain the *NtXTHs* sequence.

### 2.10. Subcellular Localization of NtXTH Proteins

The CDS fragments of *NtXTH11*, *NtXTH19*, *NtXTH40*, and *NtXTH45* were amplified using cDNA from K326 leaves by gene-specific primers with a homologous recombination arm (Supplementary File 2) using *TranStart* FastPfu Fly DNA polymerase, as mentioned above. The fragments were cloned into the binary pcam35tlegfps2#4 vector (modified based on pCAMBIA1300) to generate *35S*::NtXTHs-GFP fusion proteins using seamless cloning kits (CloneSmarter, 5891-25, Houston, TX, USA). *AtCESA1* was then cloned into the binary pcam35tlerfps2#4 vector (modified based on pCAMBIA1300) to generate the *35S*::AtCESA1-RFP fusion protein as a plasma membrane-anchored marker [45]. Positive clones were confirmed by DNA sequencing and transformed into *Agrobacterium tumefaciens* strain GV3101. *Nicotiana benthamiana* plants with six leaves were used for transient expression using the infiltrated method [46]. Confocal laser scanning microscopy was used to analyze the GFP and RFP fluorescent signals.

## 3. Results

### 3.1. Identification of NtXTHs Based on the SGN Database

**Figure 1:**
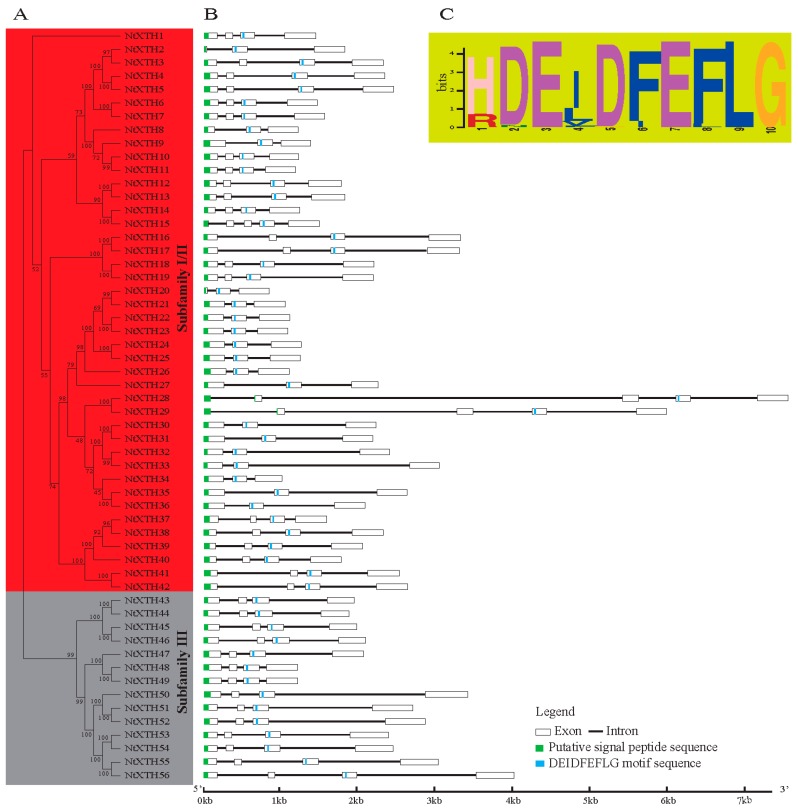
Neighbor-Joining phylogenetic relationships, gene structure, and conserved protein domain of common tobacco *XTH* genes. (**A**) The unrooted phylogenetic tree of 56 *NtXTHs*; (**B**) Exon/intron organization of common tobacco *XTH* genes; (**C**) Schematic representation of the conserved catalytic domain in common tobacco XTH proteins.

Gene family members are generally conserved in different species. In the present study, 56 members of candidate tobacco XTH proteins containing both PF00722 and PF06955 domains were identified. All of these have orthologous genes in *Arabidopsis*, and the identity ranges from 54.68 to 82.53% (Table 1). The 56 *NtXTHs* were renamed as *NtXTH1* to *NtXTH56* based on the results of the phylogenetic analysis (Table 1, Figure 1A). The length of proteins encoded by the *NtXTH* genes varied from 217 to 365 amino acids, and the average length was 296 amino acids. All of the NtXTHs proteins possess a signal peptide sequence. The prediction results relating to sub-cellular localization revealed that 48 NtXTHs were plasma membrane-anchored proteins and the other eight NtXTHs were extracellular proteins. Information on parameters such as isoelectric point (PI), molecular weight (MW), and intron numbers of NtXtH proteins are provided in Table 1. The CDS, genomic sequences, and protein sequences of NtXTHs are shown in Supplementary File 3, Supplementary File 4, and Supplementary File 5, respectively.

In our study, 56 *XTH* genes were identified in tobacco, which is greater than the number identified previously in other representative species, including *Arabidopsis*, rice, sorghum, poplar, tomato, kiwifruit, and apple. To gain insight into the size characteristics of *XTH* genes in other species, members of the *XTH* gene family in *N. sylvestris* (37), *N. tomentosiformis* (30), *N. benthamiana* (47), potato (32), pepper (23), eggplant (22), petunia (32), and coffee (23) were identified from the SGN database using the same method mentioned above. The unique accession numbers used to search the genome database are listed in Supplementary File 6. The number of *XTH* genes in these 17 species varies, ranging from 11 to 57. We noticed that multiploid plants, such as wheat and tobacco, have a higher number of *XTH* genes compared with other species. In particular, *N. tabacum* carries more *XTH* genes than its two ancestral species *N. tomentosiformis* and *N. sylvestris*, but less than the sum of the two species.

### 3.2. Phylogenetic and Structural Analyses of NtXTH Genes

Unlike *AtXTH* genes, which could be classified into three groups, the divergence between Group I and Group II *NtXTHs* was no longer apparent. However, Group II was clearly distinct from Groups I and II, indicating that the *NtXTH* genes could be divided appropriately into two major subfamilies: subfamilies I/II and subfamily III (Figure 1A). Subfamilies I/II consisted of 42 gene members and subfamily III has 14 members. A total of 23 sister pairs were identified according to phylogenetic analysis (Table 2) and all sister pairs showed high bootstrap support (>96%). Interestingly, the Ka/Ks ratios of these 23 *NtXTHs* sister pairs were less than 1 and divergence was estimated between 5.4 to 20.4 Mya (Table 2).

To gain further insight into the structural diversity of the *NtXTH* genes, we compared the exon/intron organization in the coding sequences of individual *NtXTH* genes in tobacco. As shown in Figure 1B, the most closely related genes in the subfamilies share similar exon/intron structures and intron numbers, which was consistent with the characteristics defined in the phylogenetic analysis. Overall, 55 out of 56 *NtXTH* genes contained three-to-four exons, except for *NtXTH15*, which contained five exons (Table 1, Figure 1B). To obtain intron gain/loss information of all sister pairs, the intron/exon structures of the genes that clustered together at the terminal branch of the phylogenetic tree were also compared. Among these, four pairs showed substantial changes in their intron/exon structure, including *NtXTH2/-3*, *NtXTH14/-15*, *NtXTH20/-21*, and *NtXTH28/-29*. Comparison of the four gene pairs with neighboring genes, *NtXTH2* and *NtXTH14* lost one exon compared to *NtXTH3* and *NtXTH15*, respectively. During the long evolutionary period, sister pairs *NtXTH20/-21* and *NtXTH28/-29* presented exons and introns of different lengths. In particular, *NtXTH28* and *NtXTH29* presented longer introns than the other genes (Figure 1B). Notably, the recognition splicing site of the first exon and intron of *NtXTH10*, *NtXTH11*, *NtXTH37*, *NtXTH38*, *NtXTH39*, and *NtXTH40* was GC, which differs from the regular GT splicing site (Supplementary File 4).

SignalP analysis predicted a signal peptide for entry into the secretory pathway for each of the 56 candidate NtXTH proteins (Table 1). A total of 54 out of 56 signal peptides were located in the first exon, except for NtXTH28 and NtXTH29, whose signal peptides were coded by two exons (Figure 1B). Furthermore, the conserved diagnostic amino acid sequence motif DEIDFEFLG was found in all the NtXTHs (Supplementary File 5, Figure 1C). Unlike *Arabidopsis*, the DEIDFEFLG is not confined to the second exon, and is also found in either the second, third, or fourth exons. Among the 56 *NtXTHs*, 18 genes encode the motif in the second exon, there are 35 genes in the third exon, and three genes in the fourth exon (Figure 1B).

### 3.3. Chromosomal Location, Gene Duplication, and Evolution

**Figure 2:**
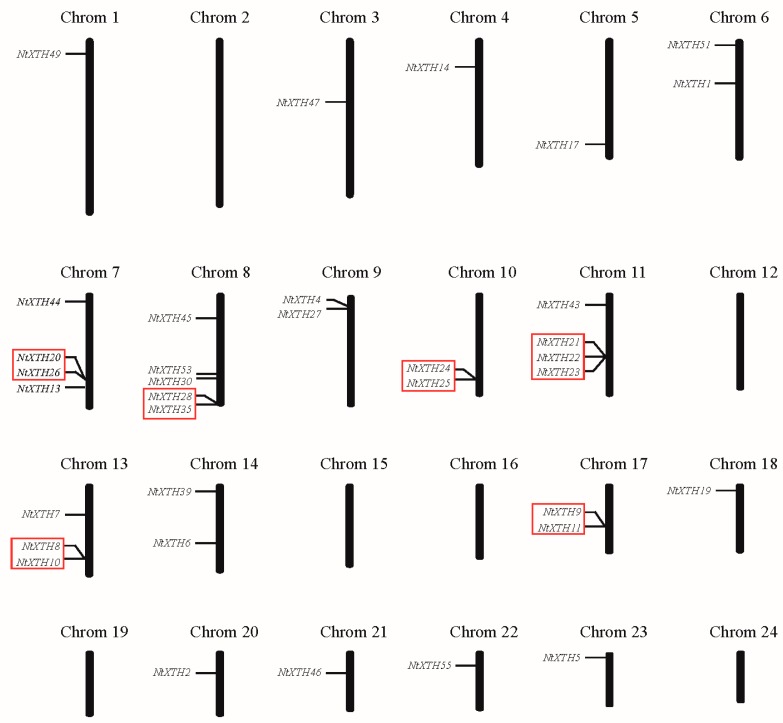
Distribution of *NtXTH* genes in common tobacco chromosomes. Red rectangles represent the putative tandem duplicate genes.

A total of 37 members of 56 tobacco *XTH* genes were mapped to the 18 chromosomes, while 19 genes were not (Figure 2). The tobacco *XTH* genes are unevenly distributed among all chromosomes. None of the *NtXTH* genes were mapped to the six chromosomes, including chromosomes 2, 12, 15, 16, 19, and 24. Chromosome 8 was found to carry five *NtXTH* genes, and the maximum number of *NtXTH* genes among chromosomes 7, 11, and 13 were four, four, and three, respectively. All other chromosomes only contained one or two *NtXTH* genes. Among the four *NtXTH* genes previously reported, *NtXTH11*, *NtXTH19*, and *NtXTH35* were mapped to chromosome 17, 18, and 8, respectively, while *NtXTH18* was mapped to unattributed scaffolds.

Five or fewer genes located in a of 100 kb range are usually regarded as tandem duplicates [47]. Notably, among the 37 genes, eight gene pairs were detected within a distance of less than 10 to 100 kb on chromosomes 7, 8, 10, 11, 13, and 17, which may be resulted from tandem duplication (Figure 2). The results of Smith-Waterman algorithm (http://www.ebi.ac.uk/Tools/psa/) alignment showed that the sequence similarities of five pairs of genes (*NtXTH21*/-*22*, *NtXTH21*/-*23*, *NtXTH9*/-*11*, *NtXTH22*/-*23* and *NtXTH24*/-*25*) exceeded 90%. Hence, the five pairs were regarded as tandem duplicates. Detailed information on the sequence similarity of these paralogous pairs is shown in Supplementary File 7.

**Figure 3:**
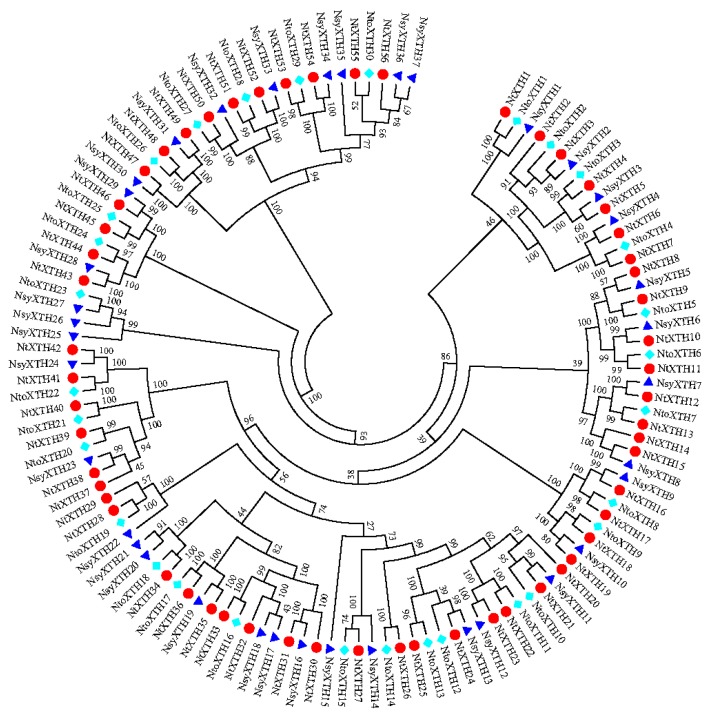
Neighbor-Joining phylogenetic tree of full-length XTH proteins in *N. tabacum* (red circle), *Nicotiana sylvestris* (dark blue triangle) and *Nicotiana tomentosiformis* (light blue diamond).

To investigate the evolutionary relationships of the identified XTH proteins, the predicted full-length amino acid sequences from *N. sylvestris*, *N. tomentosiformis*, and *N. tabacum* were used to generate a phylogenetic tree. The XTHs protein sequences of *N. sylvestrisis* (Supplementary File 8) and *N. tomentosiformis* (Supplementary File 9) were also downloaded from the SGN database using the aforementioned method. As illustrated in the Neighboring-Joining phylogenetic tree (Figure 3), XTH proteins from the two ancestral species and *N. tabacum* were organized in a similar way, and some orthologous relationships were identified. The two ancestral *XTH* genes were named based on their relationship to known *N. tabacum XTHs* according to the phylogenetic tree. The phylogenetic tree results suggested that gene fusion or elimination greatly impacted the evolution of this gene family in the common tobacco genome. Forty-seven of 56 *NtXTHs* were found to be orthologous genes of *N. sylvestrisis* or/and *N. tomentosiformis*. Among them, *NtXTH1*, *NtXTH24*, and *NtXTH27* clustered with *XTH* genes from both *N. sylvestrisis* and *N. tomentosiformis* indicating that those donor *XTHs* fused during evolution. However, other genes, such as *NtXTH14*, *NtXTH20*, *NtXTH29*, *NtXTH30*, *NtXTH32*, *NtXTH37*, and *NtXTH56* were clustered closely with other *NtXTHs* indicating that the gene duplications occurred during evolution (Figure 3). In addition, some donor *XTH* genes, for example, *NtoXTH23*, *NsyXTH25*, *NsyXTH26*, and *NsyXTH27* did not cluster with any *NtXTHs*, suggesting that a gene loss event occurred in the *XTH* gene family after polyploidization in the allotetraploid tobacco (Figure 3).

### 3.4. Analysis of XTH Genes in N. tabacum, N. tomentosiformis, N. sylvestris, Arabidopsis, and Rice

**Figure 4:**
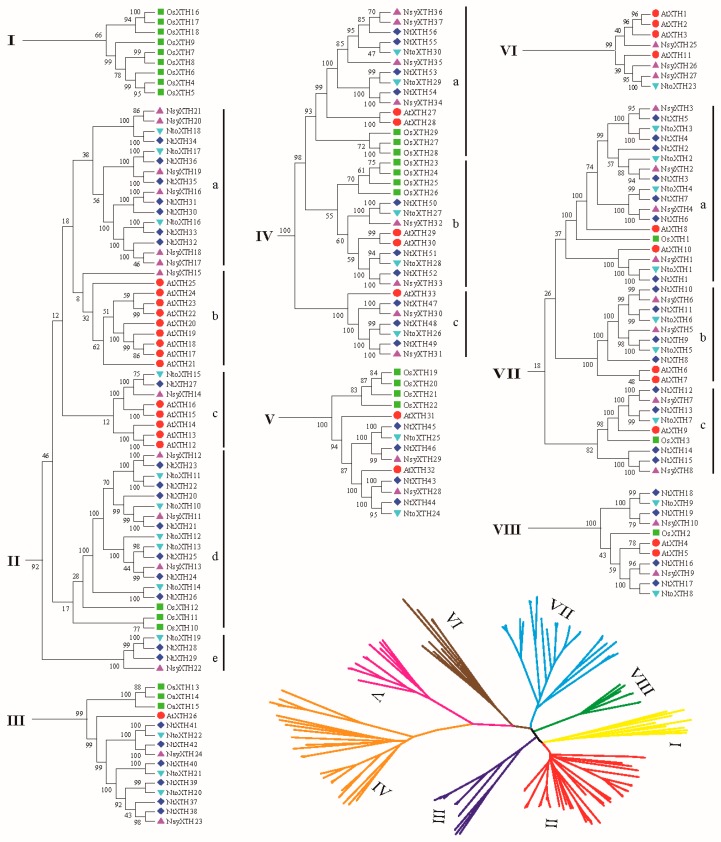
Neighbor-Joining phylogenetic tree of full-length XTH proteins in *N*. *tabacum* (dark blue diamond), *N. sylvestris* (purple triangle), *N. tomentosiformis* (light blue triangle)*, Arabidopsis* (red circle), and rice (green square).

To further examine the phylogenetic relationships of XTH proteins in dicotyledons and monocotyledon, a phylogenetic tree was constructed using the full-length XTH protein sequence alignments from *N. tabacum*, *N. tomentosiformis*, *N. sylvestris*, *Arabidopsis*, and rice. Initially, it appeared that the tree was no longer divided into two or three subfamilies, hence, eight subclades were generated, as indicated in Figure 4 (I to VIII). To clarify the evolutionary relationships among dicotyledons and monocotyledon, subclades II, IV, and VII were further divided into five, three, and three subclasses, respectively, i.e., a, b, c, d, and e. The phylogenetic tree shows that the plant *XTH* sequence distribution predominates with species bias (Figure 4). For example, Subclade I contained only rice *XTH* genes, indicating that they may have been lost in dicotyledons, such as *Arabidopsis* and some tobacco species, or acquired in the monocotyledon rice after divergence from the last common ancestor. Conversely, subclades II-c, IV-c, VI, and VII-b contained only dicotyledons *N. tabacum*, *N. tomentosiformis*, *N. sylvestris*, and *Arabidopsis* while no rice genes were detected in these subclades. Subclades II-a, II-b, and II-e contained mainly tobacco species or *Arabidopsis* (Figure 4), revealing that these *XTHs* may have occurred following duplication among these species after the dicot-monocot split. However, subclades II-d, III, IV-a, IV-b, V, VII-a, VII-c, and VIII represented all five species, suggesting that some *XTH* genes were generated before the dicot-monocot split.

### 3.5. Isolation and Sequencing of NtXTH cDNAs

**Figure 5:**
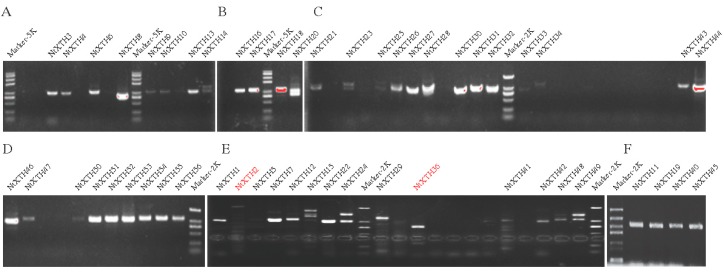
Amplification of cDNA fragments of the *NtXTHs*-. (**A**–**E**) Different rounds to amplify cDNA fragments of *NtXTH* genes. (**F**) Electrophoresis of four *NtXTHs*, which were used to study the subcellular localization.

To verify the *NtXTH* genes predicted in silico, cDNA of *NtXTH* was amplified and sequenced. A total of 55 primer pairs were designed based on the SGN database sequence, to amplify *NtXTHs*. The same primers were used for *NtXTH38* and *NtXTH39* due to their high level of sequence similarity. Additionally, *NtXTH48* and *NtXTH49* share the same forward primer. The primers that amplified *NtXTH11*, *NtXTH19*, *NtXTH40*, and *NtXTH45* were fused to a homologous arm which can also be used to construct GFP fusion proteins (Supplementary File 2). In total, 52 *NtXTHs* were amplified successfully excluding *NtXTH35*, *NtXTH37*, *NtXTH38*, and *NtXTH39* (Figure 5). Among these successfully amplified *NtXTH* genes, 44 *NtXTHs* showed a single clear and sharp band, while another eight *NtXTHs* (*XTH2*, *XTH14*, *XTH15*, *XTH20*, *XTH23*, *XTH24*, *XTH25* and *XTH49*) presented at least two bands.

Among the 44 *NtXTH* genes with a single band, the sequences of 38 *NtXTHs* were consistent with the SGN database. However, *NtXTH41* and *NtXTH44* presented one single nucleotide polymorphism (SNP) in contrast to the SGN database. *NtXTH3*, *NtXTH4*, and *NtXTH28* exhibited a 6–64 bp indel before the stop codon. However, the sequence we obtained for *NtXTH36* did not match that obtained using the SGN database. Intriguingly, among the eight genes with multiple bands, the sequence of one splice variant was consistent with the SGN database, and the other splice variants, with the insertion of one to four short fragments, formed a stop codon near the start codon to truncate the proteins (Supplementary File 10). Additionally, none of the splice variants obtained for *NtXTH2* matched those obtained with the SGN database.

Unfortunately, amplification of *NtXTH35*, *NtXTH37*, *NtXTH38*, and *NtXTH39* failed after at least four attempts using different optimization conditions. In total, the cDNA fragments of six genes (*NtXTH35*, *NtXTH37*, *NtXTH38*, *NtXTH39*, *NtXTH2*, and *NtXTH36*) were not obtained by PCR amplification.

### 3.6. Subcellular Localization of NtXTH Proteins

**Figure 6:**
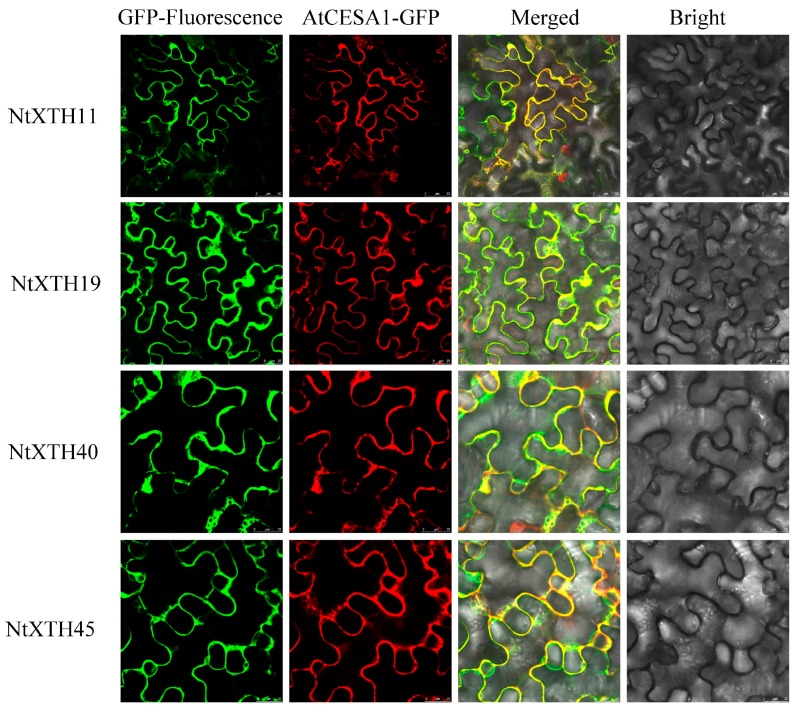
Subcellular localization of NtXTHs proteins. Transient expression of *35S*::*NtXTHs-GFP* and *35S*::*AtCESA1-RFP* in tobacco epidermal cells. AtCESA1-RFP is a plasma membrane marker protein. The green fluorescent signals of NtXTHs-GFP can be merged with the red fluorescent signals of the plasma membrane marker.

Information on the subcellular localization of proteins can help to clarify their functions, and can also be used to predict the subcellular localization of homologous genes in other species. Most NtXTHs were predicted to be localized at the plasma membrane (Table 1). To confirm this, four *NtXTH* genes (*NtXTH11*, *NtXTH19*, *NtXTH40*, and *NtXTH45*), which belonged to different groups and were predicated to occur in the plasma membrane and extracellular space, were selected to construct NtXTHs-GFP fusion proteins. After transient expression of NtXTHs-GFP fusion proteins in tobacco leaves, the GFP signals from all four NtXTHs were merged with the RFP signals of the plasma membrane marker gene *AtCESA1* (Figure 6), suggesting that *NtXTH11*, *NtXTH19*, *NtXTH40*, and *NtXTH45* were localized to the plasma membrane.

### 3.7. Expression of NtXTH Genes during the Growth Cycle

**Figure 7:**
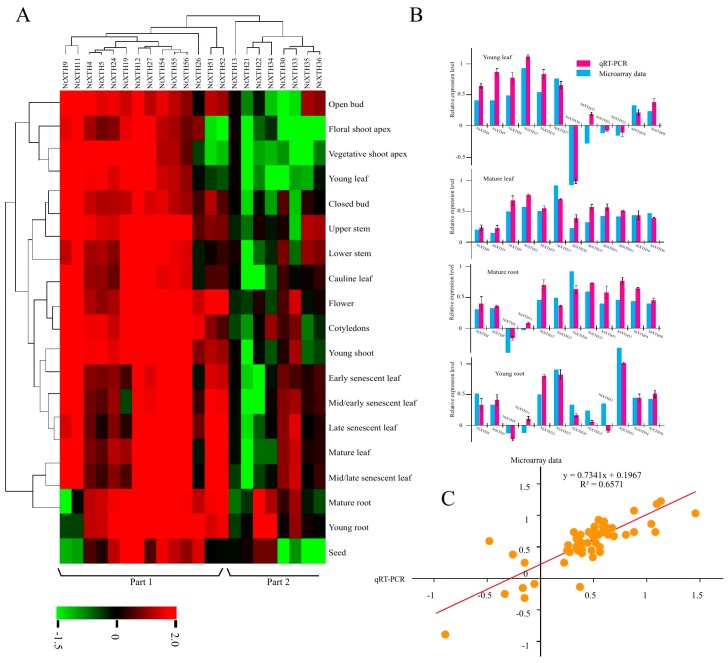
Microarray and RT-PCR analysis of *NtXTHs* in different tissues. (**A**) Hierarchical cluster analysis of *NtXTH* gene expression in different tissues. The expression data were collected through the public RNA-seq database TobEA. The mean expression value of each gene was log_2_ transformed to create the hierarchical map. Each column represents the transcriptional units of *XTH* genes. The rows represent the different tobacco tissues. Green represents the lowest expression and red represents the highest expression. (**B**) Comparison of changes in the abundance and expression levels of selected *NtXTH* genes. (**C**) Coefficient analysis between gene expression ratios obtained from TobEA and qRT-PCR data.

BLASTN analysis identified 22 *NtXTHs* with Unigene IDs corresponding to the microarray data. A hierarchical map was built with MeV software to analyze the expression profiles of the 22 *NtXTHs* in 19 different tobacco tissues, representing the tobacco growth cycle (Figure 7). The expression patterns of *NtXTHs* were divided into two distinct clusters and the two expression patterns were different. In part 1, nine *NtXTHs* belonging to the Group I/II subfamily and five *NtXTHs* belonging to the Group III subfamily presented similar expression patterns, with high gene expression in all 19 tissues. *NtXTH9* and *NtXTH11* were expressed at lower levels in mature roots, young roots, and seeds compared with other tissues. *NtXTH51* and *NtXTH52* were expressed at lower levels in the floral shoot apex, vegetative shoot apex, and young leaves compared with other tissues. Among the genes in part 1 of the Group I/II subfamily, *NtXTH4*, *NtXTH5*, *NtXTH12*, *NtXTH19*, *NtXTH24*, and *NtXTH27* generally presented relatively high expression in 19 specific tissues. However, in part 2, *NtXTH13*, *NtXTH21*, *NtXTH22*, *NtXTH30*, *NtXTH33*, *NtXTH34*, *NtXTH35*, and *NtXTH36*, which belong to the Group I/II subfamily, were expressed at low levels in the 19 studied tissues. *NtXTH13* and *NtXTH21* may be expressed at the lowest level in all 22 *XTHs* (Figure 7). However, among the 22 *NtXTHs* identified by microarray, *NtXTH35* and *NtXTH36* were not amplified successfully and two transcripts of *NtXTH24* were found (Figure 5, Supplementary File 10).

To verify whether the microarray data represented true variation in the transcripts, 12 *NtXTH* genes were randomly chosen and their expression was confirmed in four tobacco tissues (young leaf, mature leaf, young root, and mature root) using qRT-PCR. The results clearly showed that most of the qRT-PCR data were consistent with the microarray data output (Figure 5B). Linear regression analysis showed a significant correlation (*R*^2^ = 0.6571), which indicates good reproducibility between transcript abundance generated by microarray data and the expression profiles obtained from the qRT-PCR data (Figure 5C).

### 3.8. Expression Profiling of NtXTH Genes under Abiotic Stress Using qRT-PCR

**Figure 8:**
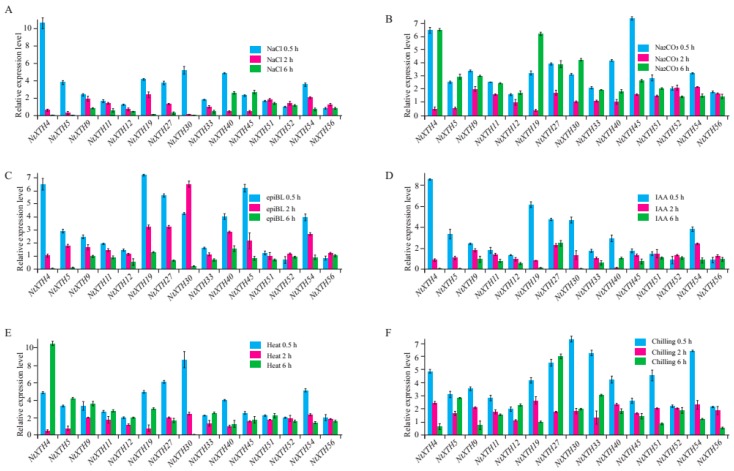
qRT-PCR analysis of *NtXTH* gene expression in response to hormone treatments and abiotic stresses. Four-weeks-old common tobacco (K326) seedlings were treated with 150 mM NaCl (**A**), 50 mM Na_2_CO_3_ (**B**), 1 µM 2,4-epi-brassinolide (epiBL) (**C**), 1 µM IAA (**D**), 37 °C heat (**E**), and 4 °C chilling (**F**).

To explore the expression profiles of *NtXTHs* in response to different abiotic stress conditions, the expression patterns of 12 selected *NtXTHs* in response to salinity and alkaline stresses, hormone epiBL and IAA treatment, heat, and chilling stress were studied by qRT-PCR in this study (Figure 8A–F). Differential expression of *NtXTHs* was observed under different stress conditions. Almost all of the *NtXTHs* were found to be up-regulated following various stress treatments (Figure 8). For NaCl stress, the expression level of *NtXTHs* reached a peak at 0.5 h time points. *NtXTH4* was significantly induced and *NtXTH5*, *NtXTH19*, *NtXTH27*, *NtXTH30*, *NtXTH40* and *NtXTH54* were moderately induced at 0.5 h compared with control (Figure 8A). For Na_2_CO_3_ stress, the expression level of *NtXTHs* reached peaks at 0.5 h and 6 h time points. The expression of most *NtXTHs* presented a trend of up-regulation at first, then down-regulation and finally up-regulation again. *NtXTH4* was induced obviously at 0.5 h and 6 h, and the highest expression time point of *NtXTH19* was 6 h. *NtXTH45* was significantly induced at 0.5 h compared with control (Figure 8B). For epiBL stress, the expression level of most *NtXTHs* reached peaks at 0.5 h and 2 h time points. *NtXTH4*, *NtXTH19*, *NtXTH27*, *NtXTH40*, *NtXTH45*, and *NtXTH54* was induced obviously at 0.5 h, however, *NtXTH30* was significantly induced at 2 h compared with control (Figure 8C). For IAA stress, the expression of *NtXTHs* was significantly induced at 0.5 h time points compared with control (Figure 8D). The expression patterns of *NtXTHs* that responded to heat and chilling stresses were similar. Most *NtXTHs* were induced at 0.5 h and 6 h time points compared with control (Figure 8E,F).

## 4. Discussion

Xyloglucan is a hemicellulosic polysaccharide presented in the primary cell walls of all land plants studied to date [1,48]. *XTHs* reconstruct the cell wall by cutting and/or rejoining xyloglucan to regulate the composition and organization of the cell wall [12]. Only a few tobacco XTH sequences have been isolated so far, including *AB017025.1* [28], *HQ108341.1*, *KJ730270*, and *D86730.1* [27]. The recent release of the genome database of common tobacco provides the opportunity to systematically study certain gene families [29]. In our study, 56 *NtXTHs* were divided into two subfamilies, Group I/II and Group III, for the first time (Figure 1).

Common tobacco is an allotetraploid species formed about 200,000 years ago by the hybridization of two diploid interspecific species: *N. sylvestrisis* (maternal donor) and *N. tomentosiformis* (paternal donor) [33]. Polyploid evolution has been accompanied by changes in chromosome number and genome size, probably through dysploid reductions via chromosome deletions or fusions [33]. A previous study reported that genome downsizing was a widespread biological response to polyploidization based on a large-scale analysis of the genome size of 3008 angiosperms [49]. The mean genome size of three *N. tabacum* species was estimated to be 4.53 Gb [29], which represents a reduction of 5.8% compared with the sum of the ancestral *N. sylvestrisis* (2.59 Gb) and *N. tomentosiformis* (2.22 Gb) genomes. This result is consistent with the previously estimated downsizing of 3.7% [33]. The genome downsizing of *N. tabacum* was also illustrated in the *XTH* gene family. We found that the number of *NtXTHs* was higher than the ancestral donors, but less than the sum of these two donors (Supplementary File 6). Phylogenetic analysis of the *XTH* gene family among *N. tabacum*, *N. sylvestrisis*, and *N. tomentosiformis* in our study revealed that most *NtXTHs* assembled at the same terminal branches as *N. sylvestrisis* or/and *N. tomentosiformis* (Figure 2). These results suggested that gene loss and fusion occurred during the long evolutionary history. Thus, the genome sequences of *N. sylvestris* and *N. tomentosiformis* are assumed to have high identity to the genome of *N. tabacum*. Furthermore, we could speculate that the 23 *NtXTHs* sister pairs could be derived from *N. sylvestrisis* and *N. tomentosiformis*, respectively, not considering the sister pairs *NtXTH22/-23* and *NtXTH24/-25*, which were regarded as tandem duplicated genes (Supplementary File 7). This hypothesis would also explain the 5.4 to 20.4 Mya divergence time of these 23 sister pairs estimated by the Ka/Ks ratio (Table 2). This divergence time is consistent with the findings of a previous study, whereby *N. sylvestris* and *N. tomentosiformis* diverged about 15 million years ago [50].

Comparison of amino acid sequences of *XTH* genes isolated from azuki bean (*Vigna angularis*), soybean (*Glycine max*), tomato (*Lycopersicum esculentum*), *Arabidopsis* (*A. thaliana*), and common wheat (*Triticum aestivum*) revealed they were highly conserved despite considerable variability in protein size [51]. XTH proteins were reported to contain several conserved modular structures, such as a short hydrophobic amino area that probably functions as a signal peptide to guide the protein to the plant cell wall, and a highly conserved DEIDFEFLG domain that acts as the catalytic site for both XET and XEH activity [52]. The high degree of conservation of XTH amino acid sequences among various plant species implies the functional conservation of these proteins in the plant kingdom. The catalytic site domain DEIDFEFLG was reported to be conserved among all the XTHs characterized thus far [10,12], and is a structural feature of all XTH proteins. In our study, the conserved domain DEIDFEFLG was identified in each NtXTH (Table 1). However, only the second, sixth, eighth, and ninth residues of the DEIDFEFLG motif were conserved in all 56 NtXTHs, and the other residues showed slight variation among NtXTHs (Table 1). Campbell and Braam compared some *Arabidopsis* XTH catalytic domains and found out that the third residue, isoleucine (I), may also be replaced by another hydrophobic residues; either leucine (L) or valine (V), and the phenylalanine (F) (fifth residue) may be substituted by I [53]. These substitutions also occurred in NtXTH proteins. These changes are predicted to have no effect on the cleavage of xyloglucan glycan chain linkages because the apolar and uncharged nature of the residues are maintained. However, the change in the first glutamate residue (E), which acts as the active site, has been shown to inactivate the protein [53].

Although the putative signal peptides varied in length, most of the NtXTHs were predicted to be secreted to the cell wall, exhibiting a membrane sub-cellular localization (Table 1), which is consistent with their ability to modify the cell wall. However, a previous study performed a transient expression test and reported that DkXTH8 derived from persimmon was anchored to the cell wall in onion epidermal cells [54]. The cell wall localization of DkXTH8 is consistent with its function in the promotion of fruit ripening and softening, due to xyloglucan endotransglycosylase activity. Furthermore, *DkXTH8* overexpression in *Arabidopsis* resulted in increased leaf senescence [54], which is an important sign of maturity in tobacco leaves. In our study, four NtXTHs derived from different phylogenetic groups and predicted to reside in the plasma membrane (NtXTH11, NtXTH19, and NtXTH40) or extracellular (NtXTH45) space, were co-localized with the plasma membrane-anchored protein AtCESA1 (Figure 6). This indicates that the four NtXTH proteins may have some biological activity in the cell wall due to the close linkage of the cell wall and plasma membrane. Further study is needed to confirm whether the four NtXTH proteins are anchored to the cell wall and to determine the enzyme (XET or XEH) they inhibit.

TobEA is an ideal database to examine gene expression in tobacco, due to the abundant genes and various tested tissues. The expression profiles of 22 *NtXTHs* in 19 different tissues of tobacco were exhibited in this study (Figure 7). Interestingly, *NtXTH35* and *NtXTH36* were found to be highly expressed in the open bud, stem, flower, and leaves based on the microarray data, while no cDNA fragments were obtained with several rounds hard amplification (Figure 5). It is possible that the different environmental conditions in the present study and the previous study, such as soil nutrients, and the various stress stimuli affecting tobacco growth, alter the gene expression profile. In addition, unpredictable and unidentified factors may prevent normal amplification. Additionally, we do not exclude the possibility that the complex allotetraploid genome of common tobacco would lead to some errors in sequence alignment for microarray data. Furthermore, *NtXTH35, NtXTH36*, and the other four unsuccessfully amplified *NtXTHs* may be pseudogenes.

Environmental factors such as salinity, alkali, heat, chilling, and plant endogenous hormones affect the expression of plant genes, resulting in the alteration of morphology and physiology. Given that the growth and productivity of tobacco are frequently threatened by environmental factors during the growth cycle, we simulated abiotic stress to examine the expression profiles of *NtXTHs*, such as salinity, heat, chilling, and alkali conditions. For stress induced by plant hormones, only IAA and epiBL treatments were used. This was due to the publication of a previous comprehensive expression analysis of all *AtXTHs*, which revealed that *AtXTHs* can be induced by a variety of plant hormones, especially brassinolide and IAA [10]. Consistent with previous studies, our qRT-PCR results revealed a complex expression profile of NtXTHs under a variety of stress treatments (Figure 8). It is the first time an exploration of the expression patterns of *NtXTHs* under alkali stress has been undertaken. A previous study reported that mRNA expressions of *AtXTH22* peaked after 2 h following treatment with 1 µM brassinolide and then declined [55]. *NtEXGT* (*NtXTH35*) was reported to be induced by auxins, brassinosteroids, salinity, and cold in leaves [27]. In our study, all of the stress treatments induced high expression of *NtXTHs* at the beginning of treatment, which was maximal at 0.5 or 2 h and then declined (Figure 8). Expressional profiling of *NtXTHs* suggested that *NtXTHs* might be involved in resistance to adverse environmental factors, or are regulated by plant hormones. A previous study reported that *CaXTH1*, *2*, and *3*, which have been isolated from another solanaceae species, the chili pepper, were concomitantly induced by a broad range of abiotic stresses, including cold temperature, drought, and high salinity [20]. Importantly, the *CaXTH3* gene was reported to enhance tolerance to high salinity in transgenic *Arabidopsis* [20] and tomato [23]. Although XTH showed multifunctional activity in relation to biotic stress tolerance, the actual function of NtXTHs involved in plant growth or development must be verified by overexpression or knock-out plants. Xyloglucan is one of the most important components of the primary cell wall and plays extremely important roles in cellulose-xyloglucan load-bearing cell wall frameworks [1,2]. However, the XET and XEH activity of XTH proteins could modify the structure of xyloglucan resulting in the changes in wall loosening, wall strengthening, cell elongation, fruit softening and so on [13]. A previous study reported that XET activity was greatest in the root hair elongation region where the epidermal cell wall formed bulge in *Arabidopsis* [56], suggesting the key role of XET in the root hair cell elongation. However, mutants *atxth27-1* and *atxth27-2* showed multiple phenotypes alteration, such as short-shaped tracheary elements in tertiary veins and yellow lesion-mimic spots in mature leaves [16]. These results indicated that XTHs were required for proper morphogenesis.

## 5. Conclusions

The physiological properties of the plant cell wall are modified by XTHs, which could cut or reconnect the xyloglucan molecules. In this study, 56 *NtXTH* genes were identified from the recently completed genomic database of tobacco. The highly conserved evolution relationship and phylogenetic analysis of NtXTH proteins in *N. tabacum*, *N. sylvestrisis*, *N. tomentosiformis*, *Arabidopsis*, and rice revealed the similar characteristics of NtXTHs in different species. The high expression level of *NtXTH4*, *NtXTH5*, *NtXTH12*, *NtXTH19*, *NtXTH24*, and *NtXTH27* in all 19 specific tissues based on the TobEA database suggests that they may play a universal cell wall modification function in various tissues. In addition, the rapidly induced expression of most of *NtXTHs* with different stresses indicated the possible functions of *NtXTHs* in tobacco against stresses. To our knowledge, this is the first time the expression pattern of *NtXTHs* in response to alkali stress has been reported. The membrane-anchored subcellular localization of NtXTH11, NtXTH19, NtXTH40, and NtXTH45, which belong to different phylogenetic groups, confirms the validity of the subcellular localization predication of NtXTHs proteins. All the results presented in this study will provide a foundation for further investigation of the function of XTH genes in common tobacco.

## Figures and Tables

**Table 1:** Characteristics of fifty-six *NtXTHs* identified in the *Nicotiana tabacum* genome.

Gene Name	Accession NO.	Previous Gene Name	Signal Position	Orthologous in *Arabidopsis* (ID%)	Protein Length	Intron	Sub-Cellular Localization	MW (Da)	PI
*NtXTH1*	mRNA_14204		1-23	67.59	293	4	Plasma membrane	34,168.57	7.68
*NtXTH2*	mRNA_63239		1-16	79.09	219	4	Plasma membrane	25,787.6	4.76
*NtXTH3*	mRNA_137463		1-20	72.26	291	4	Plasma membrane	33,977.82	4.72
*NtXTH4*	mRNA_134286		1-27	73.56	298	4	Plasma membrane	35,038.32	5.46
*NtXTH5*	mRNA_87242		1-27	73.47	298	4	Plasma membrane	34,932.28	5.46
*NtXTH6*	mRNA_14392		1-27	68.58	299	4	Plasma membrane	34,613.85	6.05
*NtXTH7*	mRNA_82272		1-24	68.29	296	4	Plasma membrane	34,346.59	6.23
*NtXTH8*	mRNA_35364		1-18	82.53	242	3	Plasma membrane	27,862.47	6.99
*NtXTH9*	mRNA_93233		1-27	79.25	291	3	Plasma membrane	33,061.33	7.04
*NtXTH10*	mRNA_35366		1-28	76.16	292	4	Plasma membrane	33,239.62	8.15
*NtXTH11*	mRNA_50079	KJ730270 [27]	1-28	76.51	292	4	Plasma membrane	33,221.52	7.64
*NtXTH12*	mRNA_23269		1-25	70.93	298	4	Plasma membrane	34,042.1	5.69
*NtXTH13*	mRNA_58849		1-25	71.28	298	4	Plasma membrane	33,987.21	5.89
*NtXTH14*	mRNA_78608		1-20	54.68	283	4	Plasma membrane	31,667.75	5.74
*NtXTH15*	mRNA_92550		1-21	56.49	288	5	Plasma membrane	32,624.85	6.75
*NtXTH16*	mRNA_86110		1-18	73.08	299	4	Plasma membrane	34,646.33	8.87
*NtXTH17*	mRNA_68366		1-21	74.13	302	4	Plasma membrane	35,183.88	8.95
*NtXTH18*	mRNA_104426	AB017025.1 [28]	1-21	70.85	295	4	Plasma membrane	33,923.42	8.65
*NtXTH19*	mRNA_123885	D86730.1 [27]	1-21	71.77	295	4	Plasma membrane	33,882.41	8.64
*NtXTH20*	mRNA_95691		1-10	66.51	217	3	Plasma membrane	24,690.52	8.95
*NtXTH21*	mRNA_39798		1-25	61.97	291	3	Plasma membrane	32,880.96	9.13
*NtXTH22*	mRNA_18536		1-20	63.54	284	3	Plasma membrane	31,877.64	8.7
*NtXTH23*	mRNA_34533		1-20	62.95	284	3	Plasma membrane	31,884.77	8.98
*NtXTH24*	mRNA_9973		1-25	61.05	290	3	Plasma membrane	32,549.69	8.97
*NtXTH25*	mRNA_122713		1-25	60.87	290	3	Plasma membrane	32,705.83	8.97
*NtXTH26*	mRNA_60328		1-30	63.25	300	3	Plasma membrane	33,809.7	8.17
*NtXTH27*	mRNA_52501		1-21	73.19	273	3	Plasma membrane	31,689.83	9.3
*NtXTH28*	mRNA_99357		1-36	62.31	337	5	Plasma membrane	38,692.06	9.37
*NtXTH29*	mRNA_110655		1-36	61.98	337	5	Plasma membrane	38,660.91	9.17
*NtXTH30*	mRNA_75358		1-23	66.78	285	3	Plasma membrane	32,501.78	7.58
*NtXTH31*	mRNA_51471		1-23	67.25	288	3	Plasma membrane	32,728.07	6.82
*NtXTH32*	mRNA_84816		1-17	71.54	279	3	Plasma membrane	31,762.65	7.61
*NtXTH33*	mRNA_55584		1-17	71.59	279	3	Plasma membrane	31,527.43	7.61
*NtXTH34*	mRNA_83457		1-22	69	272	3	Plasma membrane	30,676.35	5.84
*NtXTH35*	mRNA_8391	HQ108341.1 [27]	1-23	71.26	286	3	Plasma membrane	32,690.84	8.47
*NtXTH36*	mRNA_83477		1-23	71.1	289	3	Plasma membrane	32,898.04	8.12
*NtXTH37*	mRNA_21254		1-26	60.5	290	4	Plasma membrane	33,221.98	9.11
*NtXTH38*	mRNA_77290		1-26	62.06	290	4	Plasma membrane	33,268.95	9
*NtXTH39*	mRNA_11279		1-26	61.21	290	4	Plasma membrane	33,184.82	9.11
*NtXTH40*	mRNA_126220		1-25	60.76	289	4	Plasma membrane	33,142.73	9.1
*NtXTH41*	mRNA_126221		1-30	59.01	294	4	Plasma membrane	33,286.39	6.48
*NtXTH42*	mRNA_21253		1-30	60.5	294	4	Plasma membrane	33,467.71	7.66
*NtXTH43*	mRNA_18656		1-20	82.5	296	4	Extracellular	34,255.72	9.4
*NtXTH44*	mRNA_2368		1-20	80.99	296	4	Extracellular	34,223.71	9.38
*NtXTH45*	mRNA_110606		1-20	70.71	295	4	Extracellular	33,679.71	8.73
*NtXTH46*	mRNA_99979		1-19	71.01	294	4	Extracellular	33,602.71	8.95
*NtXTH47*	mRNA_36960		1-26	57.1	316	4	Extracellular	36,336.03	6.26
*NtXTH48*	mRNA_124647		1-26	58.09	316	4	Plasma membrane	35,865.25	6.24
*NtXTH49*	mRNA_98504		1-26	58.39	316	4	Plasma membrane	36,054.57	6.14
*NtXTH50*	mRNA_32955		1-30	63.88	365	4	Extracellular	41,821.13	8.95
*NtXTH51*	mRNA_60999		1-23	63.73	339	4	Extracellular	39,602.53	7.19
*NtXTH52*	mRNA_33005		1-25	61.95	343	4	Extracellular	39,847.83	6.92
*NtXTH53*	mRNA_20939		1-20	67.7	331	4	Plasma membrane	37,918.84	6.64
*NtXTH54*	mRNA_8840		1-20	67.61	331	4	Plasma membrane	37,970.88	6.64
*NtXTH55*	mRNA_119817		1-20	68.1	330	4	Plasma membrane	37,969.77	6.56
*NtXTH56*	mRNA_89227		1-20	69.9	330	4	Plasma membrane	37,820.69	7.64

MW: molecular weight; PI: isoelectric point.

**Table 2:** Pairwise identities and divergence period estimation of common tobacco *XTH* genes.

Paralogous pairs	Score	Identities	Similarity	Gaps	SynDif	SynPos	Ks	NSynDif	NSynPos	Ka	Ka/Ks	*T* (Mya)
*NtXTH2-NtXTH3*	1234	214/291 (73.5%)	218/291 (74.9%)	72/291 (24.7%)	16	138.4	0.13	5	518.58	0.01	0.08	10.29
*NtXTH4-NtXTH5*	1655	292/298 (98.0%)	297/298 (99.7%)	0/298 (0.0%)	31	184.5	0.19	6	709.50	0.01	0.04	15.59
*NtXTH6-NtXTH7*	1574	282/299 (94.3%)	287/299 (96.0%)	3/299 (1.0%)	35	186.5	0.22	15	701.50	0.02	0.10	17.70
*NtXTH10-NtXTH11*	1569	287/292 (98.3%)	289/292 (99.0%)	0/292 (0.0%)	23	199.3	0.13	5	676.67	0.01	0.06	10.27
*NtXTH12-NtXTH13*	1565	287/298 (96.3%)	291/298 (97.7%)	0/298 (0.0%)	39	197.1	0.23	14	696.92	0.02	0.09	18.83
*NtXTH14-NtXTH15*	1390	256/288 (88.9%)	266/288 (92.4%)	5/288 (1.7%)	19.3	191.1	0.11	44.7	654.92	0.07	0.66	8.91
*NtXTH16-NtXTH17*	1610	292/302 (96.7%)	296/302 (98.0%)	3/302 (1.0%)	24	189.3	0.14	11	707.67	0.02	0.11	11.39
*NtXTH18-NtXTH19*	1603	291/295 (98.6%)	294/295 (99.7%)	0/295 (0.0%)	27	200.6	0.15	4	684.42	0.01	0.04	12.16
*NtXTH20-NtXTH21*	1179	213/291 (73.2%)	213/291 (73.2%)	74/291 (25.4%)	19	142.7	0.15	5	508.33	0.01	0.07	12.02
*NtXTH22-NtXTH23*	1510	276/284 (97.2%)	280/284 (98.6%)	0/284 (0.0%)	35	191.8	0.21	9	660.25	0.01	0.07	17.15
*NtXTH24-NtXTH25*	1543	284/290 (97.9%)	286/290 (98.6%)	0/290 (0.0%)	41.5	195.8	0.25	7.5	674.17	0.01	0.04	20.41
*NtXTH28-NtXTH29*	1757	326/337 (96.7%)	333/337 (98.8%)	0/337 (0.0%)	23	227.7	0.11	11	783.33	0.01	0.13	8.89
*NtXTH30-NtXTH31*	1455.5	271/288 (94.1%)	277/288 (96.2%)	3/288 (1.0%)	24	191.3	0.14	15	663.67	0.02	0.17	11.25
*NtXTH32-NtXTH33*	1441	264/279 (94.6%)	274/279 (98.2%)	0/279 (0.0%)	18	184.9	0.10	15	652.08	0.02	0.22	8.55
*NtXTH35-NtXTH36*	1458	271/289 (93.8%)	274/289 (94.8%)	3/289 (1.0%)	30	187.9	0.18	16	670.08	0.02	0.14	14.71
*NtXTH37-NtXTH38*	1540	277/290 (95.5%)	283/290 (97.6%)	0/290 (0.0%)	28	191.2	0.16	13	678.83	0.02	0.12	13.36
*NtXTH41-NtXTH42*	1562	282/294 (95.9%)	285/294 (96.9%)	0/294 (0.0%)	22	195.2	0.12	15	686.83	0.02	0.18	10.02
*NtXTH43-NtXTH44*	1625	288/296 (97.3%)	294/296 (99.3%)	0/296 (0.0%)	23	204.9	0.12	8	683.08	0.01	0.10	9.97
*NtXTH45-NtXTH46*	1558	283/295 (95.9%)	291/295 (98.6%)	1/295 (0.3%)	13	204.9	0.07	11	677.08	0.02	0.25	5.43
*NtXTH48-NtXTH49*	1591	295/316 (93.4%)	303/316 (95.9%)	0/316 (0.0%)	15	222.5	0.07	22	725.50	0.03	0.44	5.79
*NtXTH51-NtXTH52*	1772	330/343 (96.2%)	333/343 (97.1%)	4/343 (1.2%)	25.5	219.4	0.13	10.5	797.58	0.01	0.11	10.35
*NtXTH53-NtXTH54*	1739	320/331 (96.7%)	325/331 (98.2%)	0/331 (0.0%)	16	227.0	0.07	11	766.60	0.01	0.20	6.07
*NtXTH55-NtXTH56*	1692	313/330 (94.8%)	320/330 (97.0%)	0/330 (0.0%)	34	225.8	0.17	18	764.25	0.02	0.14	13.78

SynDif: total number of synonymous difference; SynPos: total number of synonymous sites; Ks: number of synonymous substitution per synonymous site; NSynDif: total number of non-synonymous difference; NSynPos: total number of no synonymous sites; Ka: number of nonsynonymous substitutions per nonsynonymous site; Ka/Ks: the ratio of the rates of nonsynonymous to synonymous substitutions; *T*: divergence time; Mya: Million years ago.

## Supplementary Materials

The following are available online at http://www.mdpi.com/2073-4425/9/6/273/s1. Supplementary File 1: qRT-PCR primers used in this study. Supplementary File 2: Gene-specific primers for the amplification of *NtXTH* genes. Supplementary File 3: Coding sequences of *N. tabacum NtXTHs*. Supplementary File 4: Genomic sequences of *N. tabacum NtXTHs*. Supplementary File 5: Protein sequences of *N. tabacum* NtXTHs. Supplementary File 6: Accession numbers of *XTHs* from various species. Supplementary File 7: Pairwise identities between paralogous pairs of *NtXTH* genes. Supplementary File 8: Protein sequences of *N. sylvestris* NsyXTHs. Supplementary File 9: Protein sequences of *N. tomentosiformis* NtoXTHs. Supplementary File 10: Sequences of NtXTH fragments obtained from PCR amplification.

## References

[1] O’Neill M.A., York W.S. (2003). The composition and structure of plant primary cell walls. Plant Cell Wall.

[2] Saladie M., Rose J.K., Cosgrove D.J., Catala C. (2006). Characterization of a new xyloglucan endotransglucosylase/hydrolase (XTH) from ripening tomato fruit and implications for the diverse modes of enzymic action. Plant J..

[3] Buckeridge M.S., Santos H.P., Tiné M.A.S. (2000). Mobilisation of storage cell wall polysaccharides in seeds. Plant Physiol. Biochem..

[4] Cantarel B.L., Coutinho P.M., Rancurel C., Bernard T., Lombard V., Henrissat B. (2009). The Carbohydrate-Active EnZymes database (CAZy): An expert resource for Glycogenomics. Nucleic Acids Res..

[5] Atkinson R.G., Johnston S.L., Yauk Y.K., Sharma N.N., Schröder R. (2009). Analysis of xyloglucan endotransglucosylase/hydrolase (XTH) gene families in kiwifruit and apple. Postharvest Biol. Technol..

[6] Geisler L.J., Geisler M., Coutinho P.M., Segerman B., Nishikubo N., Takahashi J., Aspeborg H., Djerbi S., Master E., Andersson G.S. (2006). Poplar carbohydrate-active enzymes. Gene identification and expression analyses. Plant Physiol..

[7] Liu Y., Liu D., Zhang H., Gao H., Guo X., Wang D., Zhang X., Zhang A. (2007). The alpha- and beta-expansin and xyloglucan endotransglucosylase/hydrolase gene families of wheat: Molecular cloning, gene expression, and EST data mining. Genomics.

[8] Miedes E., Lorences E.P. (2009). Xyloglucan endotransglucosylase/hydrolases (XTHs) during tomato fruit growth and ripening. J. Plant Physiol..

[9] Rai K.M., Thu S.W., Balasubramanian V.K., Cobos C.J., Disasa T., Mendu V. (2016). Identification, characterization, and expression analysis of cell wall related genes in *Sorghum bicolor* (L.) Moench, a food, fodder, and biofuel crop. Front. Plant Sci..

[10] Yokoyama R., Nishitani K. (2001). A comprehensive expression analysis of all members of a gene family encoding cell-wall enzymes allowed us to predict cis-regulatory regions involved in cell-wall construction in specific organs of *Arabidopsis*. Plant Cell Physiol..

[11] Yokoyama R., Rose J.K., Nishitani K. (2004). A surprising diversity and abundance of xyloglucan endotransglucosylase/hydrolases in rice. Classification and expression analysis. Plant Physiol..

[12] Rose J.K., Braam J., Fry S.C., Nishitani K. (2002). The XTH family of enzymes involved in xyloglucan endotransglucosylation and endohydrolysis: Current perspectives and a new unifying nomenclature. Plant Cell Physiol..

[13] Cosgrove D.J. (2005). Growth of the plant cell wall. Nat. Rev. Mol. Cell Biol..

[14] Osato Y., Yokoyama R., Nishitani K. (2006). A principal role for *AtXTH18* in *Arabidopsis thaliana* root growth: A functional analysis using RNAi plants. J. Plant Res..

[15] Wu Y., Jeong B.R., Fry S.C., Boyer J.S. (2005). Change in XET activities, cell wall extensibility and hypocotyl elongation of soybean seedlings at low water potential. Planta.

[16] Matsui A., Yokoyama R., Seki M., Ito T., Shinozaki K., Takahashi T., Komeda Y., Nishitani K. (2005). *AtXTH27* plays an essential role in cell wall modification during the development of tracheary elements. Plant J..

[17] Harada T., Torii Y., Morita S., Onodera R., Hara Y., Yokoyama R., Nishitani K., Satoh S. (2011). Cloning, characterization, and expression of xyloglucan endotransglucosylase/hydrolase and expansin genes associated with petal growth and development during carnation flower opening. J. Exp. Bot..

[18] Singh A.P., Tripathi S.K., Nath P., Sane A.P. (2011). Petal abscission in rose is associated with the differential expression of two ethylene-responsive xyloglucan endotransglucosylase/hydrolase genes, *RbXTH1* and *RbXTH2*. J. Exp. Bot..

[19] Nishikubo N., Takahashi J., Roos A.A., Derba M.M., Piens K., Brumer H., Teeri T.T., Stalbrand H., Mellerowicz E.J. (2011). Xyloglucan endo-transglycosylase-mediated xyloglucan rearrangements in developing wood of hybrid aspen. Plant Physiol..

[20] Cho S.K., Kim J.E., Park J.A., Eom T.J., Kim W.T. (2006). Constitutive expression of abiotic stress-inducible hot pepper *CaXTH3*, which encodes a xyloglucan endotransglucosylase/hydrolase homolog, improves drought and salt tolerance in transgenic *Arabidopsis* plants. FEBS Lett..

[21] Lee J., Burns T.H., Light G., Sun Y., Fokar M., Kasukabe Y., Fujisawa K., Maekawa Y., Allen R.D. (2010). Xyloglucan endotransglycosylase/hydrolase genes in cotton and their role in fiber elongation. Planta.

[22] Xu W., Campbell P., Vargheese A.K., Braam J. (1996). The *Arabidopsis* XET-related gene family: Environmental and hormonal regulation of expression. Plant J..

[23] Choi J.Y., Seo Y.S., Kim S.J., Kim W.T., Shin J.S. (2011). Constitutive expression of *CaXTH3*, a hot pepper xyloglucan endotransglucosylase/hydrolase, enhanced tolerance to salt and drought stresses without phenotypic defects in tomato plants (*Solanum lycopersicum* cv. Dotaerang. Plant Cell Rep..

[24] Zhu J., Alvarez S., Marsh E.L., Lenoble M.E., Cho I.J., Sivaguru M., Chen S., Nguyen H.T., Wu Y., Schachtman D.P. (2007). Cell wall proteome in the maize primary root elongation zone. II. Region-specific changes in water soluble and lightly ionically bound proteins under water deficit. Plant Physiol..

[25] Yang K.A., Lim C.J., Hong J.K., Park C.Y., Cheong Y.H., Chung W.S., Lee K.O., Lee S.Y., Cho M.J., Lim C.O. (2006). Identification of cell wall genes modified by a permissive high temperature in Chinese cabbage. Plant Sci..

[26] Dong J., Jiang Y., Chen R., Xu Z., Gao X. (2011). Isolation of a novel xyloglucan endotransglucosylase (*OsXET9*) gene from rice and analysis of the response of this gene to abiotic stresses. Afr. J. Biotechnol..

[27] Kuluev B., Mikhaylova E., Berezhneva Z., Nikonorov Y., Postrigan B., Kudoyarova G., Chemeris A. (2017). Expression profiles and hormonal regulation of tobacco *NtEXGT* gene and its involvement in abiotic stress response. Plant Physiol. Biochem..

[28] Alexandersson E., Becker J.V., Jacobson D., Nguema E.O., Steyn C., Denby K.J., Vivier M.A. (2011). Constitutive expression of a grapevine polygalacturonase-inhibiting protein affects gene expression and cell wall properties in uninfected tobacco. BMC Res. Notes.

[29] Sierro N., Battey J.N., Ouadi S., Bakaher N., Bovet L., Willig A., Goepfert S., Peitsch M.C., Ivanov N.V. (2014). The tobacco genome sequence and its comparison with those of tomato and potato. Nat. Commun..

[30] Edwards K.D., Bombarely A., Story G.W., Allen F., Mueller L.A., Coates S.A., Jones L. (2010). TobEA: An atlas of tobacco gene expression from seed to senescence. BMC Genom..

[31] Ali J., Chen X., Chen Z., Zhao H., Zhao Y., Cheng B., Xiang Y. (2014). Genome-wide analysis of soybean HD-Zip gene family and expression profiling under salinity and drought treatments. PLoS ONE.

[32] He H., Serraj R., Yang Q. (2009). Changes in *OsXTH* gene expression, ABA content, and peduncle elongation in rice subjected to drought at the reproductive stage. Acta Physiol. Plant..

[33] Leitch I.J., Hanson L., Lim K.Y., Kovarik A., Chase M.W., Clarkson J.J., Leitch A.R. (2008). The ups and downs of genome size evolution in polyploid species of *Nicotiana* (Solanaceae). Ann. Bot..

[34] Tamura K., Stecher G., Peterson D., Filipski A., Kumar S. (2013). MEGA6: Molecular evolutionary genetics analysis version 6.0. Mol. Biol. Evol..

[35] Guo M., Lu J.P., Zhai Y.F., Chai W.G., Gong Z.H., Lu M.H. (2015). Genome-wide analysis, expression profile of heat shock factor gene family (CaHsfs) and characterisation of *CaHsfA2* in pepper (*Capsicum annuum* L.). BMC Plant Biol..

[36] Hu B., Jin J., Guo A.Y., Zhang H., Luo J., Gao G. (2015). GSDS 2.0: An upgraded gene feature visualization server. Bioinformatics.

[37] Wang L., Guo K., Yu L., Tu Y., Hu H., Wang B., Cui X., Peng L. (2010). Expression profiling and integrative analysis of the CESA/CSL superfamily in rice. BMC Plant Biol..

[38] Librado P., Rozas J. (2009). DnaSP v5: A software for comprehensive analysis of DNA polymorphism data. Bioinformatics.

[39] Doerks T., Copley R.R., Schultz J., Ponting C.P., Bork P. (2002). Systematic identification of novel protein domain families associated with nuclear functions. Genome Res..

[40] Lynch M., Conery J.S. (2000). The evolutionary fate and consequences of duplicate genes. Science.

[41] Saeed A.I., Sharov V., White J., Li J., Liang W., Bhagabati N., Braisted J., Klapa M., Currier T., Thiagarajan M. (2003). TM4: A free, open-source system for microarray data management and analysis. Biotechniques.

[42] Han Y., Wang W., Sun J., Ding M., Zhao R., Deng S., Wang F., Hu Y., Wang Y., Lu Y. (2013). *Populus euphratica XTH* overexpression enhances salinity tolerance by the development of leaf succulence in transgenic tobacco plants. J. Exp. Bot..

[43] Xu Z., Wang M., Shi D., Zhou G., Niu T., Hahn M.G., O’Neill M.A., Kong Y. (2017). DGE-seq analysis of *MUR3*-related *Arabidopsis* mutants provides insight into how dysfunctional xyloglucan affects cell elongation. Plant Sci..

[44] Livak K.J., Schmittgen T.D. (2001). Analysis of relative gene expression data using real-time quantitative PCR and the 2^-ΔΔCt^ method. Methods.

[45] Kimura S., Laosinchai W., Itoh T., Cui X., Linder C.R., Brown R.M. (1999). Immunogold labeling of rosette terminal cellulose-synthesizing complexes in the vascular plant Vigna angularis. Plant Cell.

[46] Batoko H., Zheng H.Q., Hawes C., Moore I. (2000). A Rab1 GTPase is required for transport between the endoplasmic reticulum and golgi apparatus and for normal golgi movement in plants. Plant Cell.

[47] Hu R., Qi G., Kong Y., Kong D., Gao Q., Zhou G. (2010). Comprehensive analysis of NAC domain transcription factor gene family in *Populus trichocarpa*. BMC Plant Biol..

[48] Pena M.J., Darvill A.G., Eberhard S., York W.S., O’Neill M.A. (2008). Moss and liverwort xyloglucans contain galacturonic acid and are structurally distinct from the xyloglucans synthesized by hornworts and vascular plants. Glycobiology.

[49] Leitch I.J., Bennett M.D. (2004). Genome downsizing in polyploid plants. Biol. J. Linn. Soc..

[50] Sierro N., Battey J.N., Ouadi S., Bovet L., Goepfert S., Bakaher N., Peitsch M.C., Ivanov N.V. (2013). Reference genomes and transcriptomes of *Nicotiana sylvestris* and *Nicotiana tomentosiformis*. Genome Biol..

[51] Okazawa K., Sato Y., Nakagawa T., Asada K., Kato I., Tomita E., Nishitani K. (1993). Molecular cloning and cDNA sequencing of endoxyloglucan transferase, a novel class of glycosyltransferase that mediates molecular grafting between matrix polysaccharides in plant cell walls. J. Biol. Chem..

[52] Campbell P., Braam J. (1999). Xyloglucan endotransglycosylases: Diversity of genes, enzymes and potential wall-modifying functions. Trends Plant Sci..

[53] Campbell P., Braam J. (1998). Co- and/or post-translational modifications are critical for TCH4 XET activity. Plant J..

[54] Han Y., Ban Q., Li H., Hou Y., Jin M., Han S., Rao J. (2016). DkXTH8, a novel xyloglucan endotransglucosylase/hydrolase in persimmon, alters cell wall structure and promotes leaf senescence and fruit postharvest softening. Sci. Rep..

[55] Xu W., Purugganan M.M., Polisensky D.H., Antosiewicz D.M., Fry S.C., Braam J. (1995). *Arabidopsis TCH4*, regulated by hormones and the environment, encodes a xyloglucan endotransglycosylase. Plant Cell.

[56] Vissenberg K., Fry S.C., Verbelen J.P. (2001). Root hair initiation is coupled to a highly localized increase of xyloglucan endotransglycosylase action in *Arabidopsis* roots. Plant Physiol..

