# Supplementary material for: Genome-Wide Identification and Expression Profiling Analysis of the Xyloglucan Endotransglucosylase/Hydrolase Gene Family in Tobacco (Nicotiana tabacum L.)
Source: Genes (Basel). 2018 May 24;9(6):273. doi: 10.3390/genes9060273 (PMC6027287; doi:10.3390/genes9060273)
Supplement: Supplementary file 1 [file genes-09-00273-s001.zip › Supplementary File 10.docx]

**Supplementary File 10: Sequences of *NtXTH* fragments obtained from PCR amplification.**

目录

[>NtXTH1(SGN database sequence) 1](#_Toc502824525)

[>NtXTH1 amplification (same with SGN database sequence) 1](#_Toc502824526)

[>NtXTH2 (SGN database sequence) 2](#_Toc502824527)

[>NtXTH2.1 amplification (Not NtXTH2 sequence) 2](#_Toc502824528)

[>NtXTH2.2 amplification (Not NtXTH2 sequence) 2](#_Toc502824529)

[>NtXTH2.3 amplification (Not NtXTH2 sequence) 3](#_Toc502824530)

[>NtXTH2.4 amplification (Not NtXTH2 sequence) 4](#_Toc502824531)

[>NtXTH2.5 amplification (Not NtXTH2 sequence) 4](#_Toc502824532)

[>NtXTH3 (SGN database sequence) 5](#_Toc502824533)

[>NtXTH3 amplification (6 bp deletion) 5](#_Toc502824534)

[>NtXTH4 (SGN database sequence) 5](#_Toc502824535)

[>NtXTH4 amplification (63 bp insertion) 6](#_Toc502824536)

[>NtXTH5 (SGN database sequence) 6](#_Toc502824537)

[>NtXTH5 amplification (same with SGN database sequence) 7](#_Toc502824538)

[>NtXTH6(SGN database sequence) 7](#_Toc502824539)

[>NtXTH6 amplification (same with SGN database sequence) 8](#_Toc502824540)

[>NtXTH7 (SGN database sequence) 8](#_Toc502824541)

[>NtXTH7 amplification (same with SGN database sequence) 9](#_Toc502824542)

[>NtXTH8(SGN database sequence) 9](#_Toc502824543)

[>NtXTH8 amplification (same with SGN database sequence) 9](#_Toc502824544)

[>NtXTH9 (SGN database sequence) 10](#_Toc502824545)

[>NtXTH9 amplification (same with SGN database sequence) 10](#_Toc502824546)

[>NtXTH10 (SGN database sequence) 11](#_Toc502824547)

[>NtXTH10 amplification (same with SGN database sequence) 11](#_Toc502824548)

[>NtXTH11(SGN database sequence) 11](#_Toc502824549)

[>NtXTH11 amplification (same with SGN database sequence) 12](#_Toc502824550)

[>NtXTH12 (SGN database sequence) 12](#_Toc502824551)

[>NtXTH12 amplification (same with SGN database sequence) 13](#_Toc502824552)

[>NtXTH13 (SGN database sequence) 13](#_Toc502824553)

[>NtXTH13 amplification (same with SGN database sequence) 14](#_Toc502824554)

[>NtXTH14(SGN database sequence) 14](#_Toc502824555)

[>NtXTH14.1 amplification (same with SGN database sequence) 14](#_Toc502824556)

[>NtXTH14.2 amplification (3 fragments insertion) 15](#_Toc502824557)

[>NtXTH15(SGN database sequence) 15](#_Toc502824558)

[>NtXTH15.1 amplification (same with SGN database sequence) 16](#_Toc502824559)

[>NtXTH15.2 amplification (1 fragment insertion) 16](#_Toc502824560)

[>NtXTH15.3 amplification (4 fragments insertion) 17](#_Toc502824561)

[>NtXTH16(SGN database sequence) 18](#_Toc502824562)

[>NtXTH16 amplification (same with SGN database sequence) 18](#_Toc502824563)

[>NtXTH17 (SGN database sequence) 18](#_Toc502824564)

[>NtXTH17 amplification (same with SGN database sequence) 19](#_Toc502824565)

[>NtXTH18 (SGN database sequence) 19](#_Toc502824566)

[>NtXTH18 amplification (same with SGN database sequence) 20](#_Toc502824567)

[>NtXTH19 (SGN database sequence) 20](#_Toc502824568)

[>NtXTH19 amplification (same with SGN database sequence) 21](#_Toc502824569)

[>NtXTH20 (SGN database sequence) 21](#_Toc502824570)

[>NtXTH20.1 amplification (same with SGN database sequence) 21](#_Toc502824571)

[>NtXTH20.2 amplification (1 fragment insertion) 22](#_Toc502824572)

[>NtXTH21 (SGN database sequence) 22](#_Toc502824573)

[>NtXTH21 amplification (same with SGN database sequence) 23](#_Toc502824574)

[>NtXTH22 (SGN database sequence) 23](#_Toc502824575)

[>NtXTH22 amplification (same with SGN database sequence) 23](#_Toc502824576)

[>NtXTH23 (SGN database sequence) 24](#_Toc502824577)

[>NtXTH23.1 amplification (same with SGN database sequence) 24](#_Toc502824578)

[>NtXTH23.2 amplification (2 fragments insertion) 25](#_Toc502824579)

[>NtXTH24 (SGN database sequence) 25](#_Toc502824580)

[>NtXTH24.1 amplification (same with SGN database sequence) 25](#_Toc502824581)

[>NtXTH24.2 amplification (2 fragments insertion) 26](#_Toc502824582)

[>NtXTH25 (SGN database sequence) 27](#_Toc502824583)

[>NtXTH25.1 amplification (same with SGN database sequence) 27](#_Toc502824584)

[>NtXTH25.2 amplification (2 fragments insertion) 27](#_Toc502824585)

[>NtXTH26 (SGN database sequence) 28](#_Toc502824586)

[>NtXTH26 amplification (same with SGN database sequence) 28](#_Toc502824587)

[>NtXTH27 (SGN database sequence) 29](#_Toc502824588)

[>NtXTH27 amplification (same with SGN database sequence) 29](#_Toc502824589)

[>NtXTH28 (SGN database sequence) 30](#_Toc502824590)

[>NtXTH28 amplification (64 bp insertion and 10 bp deletion) 30](#_Toc502824591)

[>NtXTH29 (SGN database sequence) 31](#_Toc502824592)

[>NtXTH29 amplification (same with SGN database sequence) 31](#_Toc502824593)

[>NtXTH30 (SGN database sequence) 32](#_Toc502824594)

[>NtXTH30 amplification (same with SGN database sequence) 32](#_Toc502824595)

[>NtXTH31 (SGN database sequence) 33](#_Toc502824596)

[>NtXTH31 amplification (same with SGN database sequence) 33](#_Toc502824597)

[>NtXTH32 (SGN database sequence) 33](#_Toc502824598)

[>NtXTH32 amplification (same with SGN database sequence) 34](#_Toc502824599)

[>NtXTH33 (SGN database sequence) 34](#_Toc502824600)

[>NtXTH33 amplification (same with SGN database sequence) 35](#_Toc502824601)

[>NtXTH34 (SGN database sequence) 35](#_Toc502824602)

[>NtXTH34 amplification (same with SGN database sequence) 35](#_Toc502824603)

[>NtXTH35 (SGN database sequence) 36](#_Toc502824604)

[>Amplification failed 36](#_Toc502824605)

[>NtXTH36 (SGN database sequence) 36](#_Toc502824606)

[>NtXTH36 amplification (Not NtXTH36 sequence) 37](#_Toc502824607)

[>NtXTH37 (SGN database sequence) 37](#_Toc502824608)

[>Amplification failed 38](#_Toc502824609)

[>NtXTH38 (SGN database sequence) 38](#_Toc502824610)

[>Amplification failed 38](#_Toc502824611)

[>NtXTH39 (SGN database sequence) 38](#_Toc502824612)

[>Amplification failed 38](#_Toc502824613)

[>NtXTH40 (SGN database sequence) 38](#_Toc502824614)

[>NtXTH40 amplification (same with SGN database sequence) 39](#_Toc502824615)

[>NtXTH41 (SGN database sequence) 39](#_Toc502824616)

[>NtXTH41 amplification (1 SNP with SGN database sequence) 40](#_Toc502824617)

[>NtXTH42 (SGN database sequence) 40](#_Toc502824618)

[>NtXTH42 amplification (same with SGN database sequence) 41](#_Toc502824619)

[>NtXTH43(SGN database sequence) 41](#_Toc502824620)

[>NtXTH43 amplification (same with SGN database sequence) 41](#_Toc502824621)

[>NtXTH44 (SGN database sequence) 42](#_Toc502824622)

[>NtXTH44 amplification (1 SNP with SGN database sequence) 42](#_Toc502824623)

[>NtXTH45(SGN database sequence) 43](#_Toc502824624)

[>NtXTH45 amplification (same with SGN database sequence) 43](#_Toc502824625)

[>NtXTH46 (SGN database sequence) 44](#_Toc502824626)

[>NtXTH46 amplification (same with SGN database sequence) 44](#_Toc502824627)

[>NtXTH47 (SGN database sequence) 44](#_Toc502824628)

[>NtXTH47 amplification (same with SGN database sequence) 45](#_Toc502824629)

[>NtXTH48 (SGN database sequence) 45](#_Toc502824630)

[>NtXTH48 amplification (same with SGN database sequence) 46](#_Toc502824631)

[>NtXTH49 (SGN database sequence) 46](#_Toc502824632)

[>NtXTH49.1 amplification (same with SGN database sequence) 47](#_Toc502824633)

[>NtXTH49.2 amplification (3 fragments insertion) 47](#_Toc502824634)

[>NtXTH50(SGN database sequence) 48](#_Toc502824635)

[>NtXTH50 amplification (same with SGN database sequence) 48](#_Toc502824636)

[>NtXTH51(SGN database sequence) 49](#_Toc502824637)

[>NtXTH51 amplification (same with SGN database sequence) 49](#_Toc502824638)

[>NtXTH52(SGN database sequence) 49](#_Toc502824639)

[>NtXTH52 amplification (same with SGN database sequence) 50](#_Toc502824640)

[>NtXTH53 (SGN database sequence) 50](#_Toc502824641)

[>NtXTH53 amplification (same with SGN database sequence) 51](#_Toc502824642)

[>NtXTH54 (SGN database sequence) 51](#_Toc502824643)

[>NtXTH54 amplification (same with SGN database sequence) 52](#_Toc502824644)

[>NtXTH55 (SGN database sequence) 52](#_Toc502824645)

[>NtXTH55 amplification (same with SGN database sequence) 53](#_Toc502824646)

[>NtXTH56 (SGN database sequence) 53](#_Toc502824647)

[>NtXTH56 amplification (same with SGN database sequence) 54](#_Toc502824648)

# >NtXTH1(SGN database sequence)

ATGAATAACTTCTCTACACTTATTTTCTTTGTCACTGCTTTTATTTATTTGTTTCATATTACATTAGCTTCCATAGTTTCAACAGGAGATTTCAATAAGGATTTTATAGTGCCTTGGTCCCCTAACCATGTAAATACTTCTGCCGATGGCCATACAAGAAGCTTGATATTTGATAAGGAATCTGGTTCAGGGATTGCTTCAAATGATACGTACTTGTTTGGTCAATTCGACATGAAAATTAAGTTGATACCAGGAAATTCCGCAGGCACGGTCGTGGCATTTTATTTAACTTCGTATCAACCAAATCGT***GACGAGGTAGATTTTGAGTTTCTGGGA***AATGTTCCTGGGAAACCTTATACTCTTCAAACGAATGTTTATGTCGATGGGTTGGACGATAGAGAACAGAGAATCAACTTGTGGTTTGATCCAACACAAGACTTCCACACTTATTCTATTCTGTGGAACCTTCACCAAATTGTGTTTATGGTTGATCGGGTACCTATTAGAACGTACAGAAACCATGCAGATAAAGGAGCTAAATATCCTCGTTGGCAACCAATGGCACTCCAAATTAGCATATGGAATGGAGAAAGTTGGGCAACAGATGGTGGAAAAACAAAAATTGATTGGTCAAAAGCACCATTTGTAGCCTCTTTAGGAAATTACACAATTGATGCTTGCGTTTGGAAAGGAAATGCAAGATTTTGCAGAGGAGAAAGTGAAAATAATTGGTGGAATAAGGAGAAATTCAGCACTTTGACATGGACTCAAAGAAGGTTGTTCAAATGGGTCAGAAAATATCATTTGACATATGATTATTGCATGGATAATCAACGGTTTCAAAATAATCTTCCCATAGAGTGCTCTCTCCCAAAGTATTAA

## >NtXTH1 amplification (same with SGN database sequence)

AATTACACATATTCCCTCATCCTCCATTCTTGATATTTTTCCAAGGAGAAAAT**ATG**AATAACTTCTCTACACTTATTTTCTTTGTCACTGCTTTTATTTATTTGTTTCATATTACATTAGCTTCCATAGTTTCAACAGGAGATTTCAATAAGGATTTTATAGTGCCTTGGTCCCCTAACCATGTAAATACTTCTGCCGATGGCCATACAAGAAGCTTGATATTTGATAAGGAATCTGGTTCAGGGATTGCTTCAAATGATACGTACTTGTTTGGTCAATTCGACATGAAAATTAAGTTGATACCAGGAAATTCCGCAGGCACGGTCGTGGCATTTTATTTAACTTCGTATCAACCAAATCGTGACGAGGTAGATTTTGAGTTTCTGGGAAATGTTCCTGGGAAACCTTATACTCTTCAAACGAATGTTTATGTCGATGGGTTGGACGATAGAGAACAGAGAATCAACTTGTGGTTTGATCCAACACAAGACTTCCACACTTATTCTATTCTGTGGAACCTTCACCAAATTGTGTTTATGGTTGATCGGGTACCTATTAGAACGTACAGAAACCATGCAGATAAAGGAGCTAAATATCCTCGTTGGCAACCAATGGCACTCCAAATTAGCATATGGAATGGAGAAAGTTGGGCAACAGATGGTGGAAAAACAAAAATTGATTGGTCAAAAGCACCATTTGTAGCCTCTTTAGGAAATTACACAATTGATGCTTGCGTTTGGAAAGGAAATGCAAGATTTTGCAGAGGAGAAAGTGAAAATAATTGGTGGAATAAGGAGAAATTCAGCACTTTGACATGGACTCAAAGAAGGTTGTTCAAATGGGTCAGAAAATATCATTTGACATATGATTATTGCATGGATAATCAACGGTTTCAAAATAATCTTCCCATAGAGTGCTCTCTCCCAAAGTAT**TAA**TCATCAACATCGATTAAAGGTGTCTAATCGACGAGTTGGACTAAATTCAAAAGGT

# >NtXTH2 (SGN database sequence)

ATGAAGTTGAAATTGGTAGGAGGTGACTCTGCTGGTGTTGTCACAGCTTATTATATGTGCACAGAAGATGGGGCAGGGCCAACTAGA***GATGAGGTAGACTTTGAGTTTTTGGGA***AATAGGACAGGGGAACCCTATCTTATTCAGACCAATGTGTACAAAAATGGCACTGGTGGGCGTGAGATGAGGCACGTTCTCTGGTTTGACCCCACTGAGGACTTCCATTCCTATTCCCTTCTTTGGAACTCTCACCAGCTCGTGTTTTTCGTGGATGAGGTTCCGATAAGGGTATACAAAAACGCGAATTATACGAACAATTTCTTTCCTAATGAGAAACCAATGTACTTGTTTTCAAGCATATGGAATGCAGATGACTGGGCTACTAGGGGTGGTTTGGAGAAAACAGATTGGAAAAATGCACCATTTGTTTCAACATATAAAGATTTCAGTGTAGATGGTTGCCAATGGGAAGATCCTTTTCCTACTTGTGTTTCAACAACCACTAAAAACTGGTGGGATCAGTACAATTCTTGGCACTTATCAAGTGACCAGAAATTGAATTATGCTTGGGTACAACGAAACCTTGTGATTTATGATTATTGCCAGGATACAAAGAGATATCCAGAAAAGCCTGAGGAATGTTGGTTAAGTCCCTGGGATTAA

## >NtXTH2.1 amplification (Not NtXTH2 sequence)

ATAACCGTATTACCGCCTTTGAGTGAGCTGATACCGCTCGCCGCAGCCGAACGACCGAGCGCAGCGAGTCAGTGAGCGAGGAAGCGGAAGAGCGCCCAATACGCAAACCGCCTCTCCCCGCGCGTTGGCCGATTCATTAATGCAGCTGGCACGACAGGTTTCCCGACTGGAAAGCGGGCAGTGAGCGCAACGCAATTAATGTGAGTTAGCTCACTCATTAGGCACCCCAGGCTTTACACTTTATGCTTCCGGCTCGTATGTTGTGTGGAATTGTGAGCGGATAACAATTTCACACAGGAAACAGCTATGACCATGATTACGCCAAGCTTGGTACCGAGCTCGGATCCACTAGTAACGGCCGCCAGTGTGCTGGAATTGCCCTTCGGCCGAAACTATTTAGCTACACTTCAATTAGAGGCAACATTTTCAAAAAACTCTCCTAACTCATCATGCCAAGAATATTTAAAACAGATTAAGCACCTTGCCATAACACCCTAACCTTAAGACTCAACTCAAAAGCACCAATTTTTCAAGACCTGCCACTTACTCTAACACAGAAGCGAAAGACATTACATTTCCCTCGTAAGTCATGTTTCCCTTACAACAAATAATGGAGAGTAGTCCCACAAACATAAAATTCAAGAACAAACAAAGACTCAGAATAGAATGAATTAACTCACTCTCAAAACTAAAATTTATTTGGCATAAAAGATGTACCATATGCTTGCCCGTGGTGTAATATTCTATTAATTCAAGCTCACTCAGTCAAAGATCAAGTAAGACTTTATTTTTGGGTCGAAATGTAAAGGGCAATTCTGCAGATATCCATCACACTGGCGGCCGCTCGAGCATGCATCTAGAGGGCCCAATTCGCCCTATAGTGAGTCGTATTACAATTCACTGGCCGTCGTTTTACAACGTCGTGACTGGGAAAACCCTGGCGTTACCCAACTTAATCGCCTTGCAGCACATCCCCCTTTCGCCAGCTGGCGTAATAGCGAAGAGGCCCGCACCGATCGCCCTTCCCAACAGTTGCGCAGCCTGAATGGCGAATGGACGCGCCCTGTAGCGGCGCATTAAGCGCGGCGGGTGTGGTGGTTACGCGCAGCGTGACCGCTACACTTGCCAGCGCCCTAGCGCCCGCTCCTTTCGCTTTCTTCCCTTCCTTTCTCGCCACGTTCGCCGGCTTTCCCCGTCAAGCTCTAAATCGGGGGCTCCCTTTAGGGTTC

## >NtXTH2.2 amplification (Not NtXTH2 sequence)

GCCTACATCCCTCGTTTTGCTAATCCTGTTACCAGTGGCTGCTCCCAGTGGCGATAAGTCGTGTCTTACCGGGTTGGACTCAAGACGATAGTTACCGGATAAGGCGCAGCGGTCGGGCTGAACGGGGGGTTCGTGCACACAGCCCAGCTTGGAGCGAACGACCTACACCGAACTGAGATACCTACAGCGTGAGCTATGAGAAAGCGCCACGCTTCCCGAAGGGAGAAAGGCGGACAGGTATCCGGTAAGCGGCAGGGTCGGAACAGGAGAGCGCACGAGGGAGCTTCCAGGGGGAAACGCCTGGTATCTTTATAGTCCTGTCGGGTTTCGCCACCTCTGACTTGAGCGTCGATTTTTGTGATGCTCGTCAGGGGGGCGGAGCCTATGGAAAAACGCCAGCAACCGGCCTTTTTACGGTTCCTGGCCTTTTGCTGGCCTTTTGCTCACATGTTCTTTCCTGCGTTATCCCCTGATTCTGTGGATAACCGTATTACCGCCTTTGAGTGAGCTGATACCGCTCGCCGCAGCCGAACGACCGAGCGCAGCGAGTCAGTGAGCGAGGAAGCGGAAGAGCGCCCAATACGCAAACCGCCTCTCCCCGCGCGTTGGCCGATTCATTAATGCAGCTGGCACGACAGGTTTCCCGACTGGAAAGCGGGCAGTGAGCGCAACGCAATTAATGTGAGTTAGCTCACTCATTAGGCACCCCAGGCTTTACACTTTATGCTTCCGGCTCGTATGTTGTGTGGAATTGTGAGCGGATAACAATTTCACACAGGAAACAGCTATGACCATGATTACGCCAAGCTTGGTACCGAGCTCGGATCCACTAGTAACGGCCGCCAGTGTGCTGGAATTGCCCTTTACATTTCGACCCAAAAATAAAGGCTAAGAAGTAGTGACGAGCTAAATAGTTTCGGCCGAAGGGCAATTCTGCAGATATCCATCACACTGGCGGCCGCTCGAGCATGCATCTAGAGGGCCCAATTCGCCCTATAGTGAGTCGTATTACAATTCACTGGCCGTCGTTTTACAACGTCGTGACTGGGAAAACCCTGGCGTTACCCAACTTAATCGCCTTGCAGCACATCCCCCTTTCGCCAGCTGGCGTAATAGCGAAGAGGCCCGCACCGATCGCCCTTCCCAACAGTTGCGCAGCCTGAATGGCGAATGGACGCGCCCTGTAGCGGCGCATTAAGCGCGGCGGGTGTGGTGGTTACGCGCAGCGTGACCGCTACACTTGCCAGCGCCCTAGCGCCCGCTCCTTTCGCTTTCTTCCCTTCCTTTCTCGCCACGTTCGCCGGCTTTCCCCGTCAAGCTCTAAATCGGGGGCTCCCTTTAGGGTTCCGATTTAGTGCTTTACGGCACCTCGACCCCAAAAAACTTGATTAGGGTGATGGTTCACGTAGTGGGCCATCGCCCTGATAGACGGTTTTTCGCCCTTTGACGTTGGAGTCCACGTTCTTTAATAGTGGACTCTTGTTCCAAACTGGAACAACACTCAACCCTATCTCGGTCTATTCTTTTGATTTATAAGGGATTTTGCCGATTTCGGCCTATTGGTTAAAAAATGAGCTGATTTAACAAAAATTTAACGCGAATTTTAACAAAATTCAGGGCGCAAGGGCTGCTAAAGGAAGCGGAACACGTAGAAAGCCAGTCCGCAGAAACGGTGCTGACCCCGGATGAATGTCAGCTACTGGGCTATCTGGACAAGGGAAAACGCAAGCGCAAAGAGAAAGCAGGTAGCTTGCAGTGGG

## >NtXTH2.3 amplification (Not NtXTH2 sequence)

TTGGCTAATCCTGTTACCAGTGGCTGCTGCCAGTGGCGATAAGTCGTTTCTTACCGGGTTGGACTCAAGACGATAGTTACCGGATAAGGCGCAGCGGTCGGGCTGAACGGGGGGTTCGTGCACACAGCCCAGCTTGGAGCGAACGACCTACACCGAACTGAGATACCTACAGCGTGAGCTATGAGAAAGCGCCACGCTTCCCGAAGGGAGAAAGGCGGACAGGTATCCGGTAAGCGGCAGGGTCGGAACAGGAGAGCGCACGAGGGAGCTTCCAGGGGGAAACGCCTGGTATCTTTATAGTCCTGTCGGGTTTCGCCACCTCTGACTTGAGCGTCGATTTTTGTGATGCTCGTCAGGGGGGCGGAGCCTATGGAAAAACGCCAGCCAACgCGGCCTTTTTACGGTTCCTGGCCTTTTGCTGGCCTTTTGCTCACATGTTCTTTCCTGCGTTATCCCCTGATTCTGTGGATAACCGTATTACCGCCTTTGAGTGAGCTGATACCGCTCGCCGCAGCCGAACGACCGAGCGCAGCGAGTCAGTGAGCGAGGAAGCGGAAGAGCGCCCAATACGCAAACCGCCTCTCCCCGCGCGTTGGCCGATTCATTAATGCAGCTGGCACGACAGGTTTCCCGACTGGAAAGCGGGCAGTGAGCGCAACGCAATTAATGTGAGTTAGCTCACTCATTAGGCACCCCAGGCTTTACACTTTATGCTTCCGGCTCGTATGTTGTGTGGAATTGTGAGCGGATAACAATTTCACACAGGAAACAGCTATGACCATGATTACGCCAAGCTTGGTACCGAGCTCGGATcCACTAGTAACGGCCGCCAGTGTGCTGGAATTGCCCTTTACATTTCGACCCAAAAATAAAGGAAAAATCGCTATCTACTTTCTGGAGTTCAAACTTGACCTAAATTTGACCAAAAAACTATTTATACACAGCTAAATAGTTTCGGCGAAGGGCAATTCTGCAGATATCCATCACACTGGCGGCCGCTCGAGCATGCATCTAGAGGGCCCAATTCGCCCTATAGTGAGTCGTATTACAATTCACTGGCCGTCGTTTTACAACGTCGTGACTGGGAAAACCCTGGCGTTACCCAACTTAATCGCCTTGCAGCACATCCCCCTTTCGCCAGCTGGCGTAATAGCGAAGAGGCCCGCACCGATCGCCCTTCCCAACAGTTGCGCAGCCTGAATGGCGAATGGACGCGCCCTGTAGCGGCGCATTAAGCGCGGCGGGTGTGGTGGTTACGCGCAGCGTGACCGCTACACTTGCCAGCGCCCTAGCGCCCGCTCCTTTCGCTTTCTTCCCTTCCTTTCTCGCCACGTTCGCCGGCTTTCCCCGTCAAGCTCTAAATCGGGGGCTCCCTTTAGGGTTCCGATTTAGTGCTTTACGGCACCTCGACCCCAAAAAACTTGATTAGGGTGATGGTTCACGTAGTGGGCCATCGCCCTGATAGACGGTTTTTCGCCCTTTGACGTTGGAGTCCACGTTCTTTAATAGTGGACTCTTGTTCCAAACTGGAACAACACTCAACCCTATCTCGGTCTATTCTTTTGATTTATAAGGGATTTTGCCGATTTCGGCCTATTGGTTAAAAAATGAGCTGATTTAACAAAAATTTAACGCGAATTTTAACAAAATTCAGGGCGCAAGGGCTGCTAAAGGAAGCGGAACACGTAGAAAGCCAGTCCGCAGAAACGGTGCTGACCCCGGA

## >NtXTH2.4 amplification (Not NtXTH2 sequence)

ATGAAGTTGAAATTGGTAGGAGGTGACTCTGCTGGTGTTGTCACAGCTTATTATATGTGCACAGAAGATGGGGCAGGGCCAACTAGAGATGAGGTAGACTTTGAGTTTTTGGGAAATAGGACAGGGGAACCCTATCTTATTCAGACCAATGTGTACAAAAATGGCACTGGTGGGCGTGAGATGAGGCACGTTCTCTGGTTTGACCCCACTGAGGACTTCCATTCCTATTCCCTTCTTTGGAACTCTCACCAGCTCGTGTTTTTCGTGGATGAGGTTCCGATAAGGGTATACAAAAACGCGAATTATACGAACAATTTCTTTCCTAATGAGAAACCAATGTACTTGTTTTCAAGCATATGGAATGCAGATGACTGGGCTACTAGGGGTGGTTTGGAGAAAACAGATTGGAAAAATGCACCATTTGTTTCAACATATAAAGATTTCAGTGTAGATGGTTGCCAATGGGAAGATCCTTTTCCTACTTGTGTTTCAACAACCACTAAAAACTGGTGGGATCAGTACAATTCTTGGCACTTATCAAGTGACCAGAAATTGAATTATGCTTGGGTACAACGAAACCTTGTGATTTATGATTATTGCCAGGATACAAAGAGATATCCAGAAAAGCCTGAGGAATGTTGGTTAAGTCCCTGGGATTAA

## >NtXTH2.5 amplification (Not NtXTH2 sequence)

AAGGGCGAAAAACCGTCTATCAGGGCGATGGCCCACTACGTGAACCATCACCCTAATCAAGTTTTTTGGGGTCGAGGTGCCGTAAAGCACTAAATCGGAACCCTAAAGGGAGCCCCCGATTTAGAGCTTGACGGGGAAAGCCGGCGAACGTGGCGAGAAAGGAAGGGAAGAAAGCGAAAGGAGCGGGCGCTAGGGCGCTGGCAAGTGTAGCGGTCACGCTGCGCGTAACCACCACACCCGCCGCGCTTAATGCGCCGCTACAGGGCGCGTCCATTCGCCATTCAGGCTGCGCAACTGTTGGGAAGGGCGATCGGTGCGGGCCTCTTCGCTATTACGCCAGCTGGCGAAAGGGGGATGTGCTGCAAGGCGATTAAGTTGGGTAACGCCAGGGTTTTCCCAGTCACGACGTTGTAAAACGACGGCCAGTGAATTGTAATACGACTCACTATAGGGCGAATTGGGCCCTCTAGATGCATGCTCGAGCGGCCGCCAGTGTGATGGATATCTGCAGAATTGCCCTTCGGCCGAAACTATTTAGCTACAACAAGTAAACATACAAGGAAAAGACCAACCTCAGGATTTGTGTTAAACTCTTTGACAGCCCTGTCTCTAGCAGCAAGAGACATTGTACCATCAAGCCTCTCATACTTAAAACAAAACTGGTTCAAAGCACGCTCAACCAAGTTTAACATGCCTGTCCACTGAGAGAAGATAATGGCCTTTATTGGCCCCTTGTCTTGCGATTCTGAATCTGCTTTTCCCAAATTTGACGAATCGCCATTGCATTGCACCAAGCTATCTGATTCTAAATAAGGATCCTTTGACTTAGAACATGAGTGAAGAATTTCAAGAGCAGCCTTTATTTTTGGGTCGAAATGTAAAGGGCAATTCCAGCACACTGGCGGCCGTTACTAGTGGATCCGAGCTCGGTACCAAGCTTGGCGTAATCATGGTCATAGCTGTTTCCTGTGTGAAATTGTTATCCGCTCACAATTCCACACAACATACGAGCCGGAAGCATAAAGTGTAAAGCCTGGGGTGCCTAATGAGTGAGCTAACTCACATTAATTGCGTTGCGCTCACTGCCCGCTTTCCAGTCGGGAAACCTGTCGTGCCAGCTGCATTAATGAATCGGCCAACGCGCGGGGAGAGGCGGTTTGCGTATTGGGCGCTCTTCCGCTTCCTCGCTCACTGACTCGCTGCGCTCGGTCGTTCGGCTGCGGCGAGCGGTATCAGCTCACTCAAAGGCGGTAATACGGTTATCCACAGAATCAGGGGATAACGCAGGAAAGAACATGTGAGCAAAAGGCCAGCAAAAGGCCAGGAACCGTAAAAGGCGCCGTTTGCTGGCGTTTTTCCATAGGCTCC

# >NtXTH3 (SGN database sequence)

ATGGCTAATCTTCTCTTAATTGCAGTTTTAATTGCTATTTATTGTTCACTATCTCAAGCTGAAGTTAAAGGTTCATTTGATGACAACTTTAGTAAAAGTTGTCCTGAATCTCACTTCAAGACTTCTGAAGATGGACAGATCTGGTATCTCTCCTTAGACCACAAAGCAGGATGTGGATTTATGACAAGGCAGAAATACAGATTTGGTTGGTTTAGCATGAAGTTGAAATTGGTAGGAGGTGACTCTGCTGGTGTTGTCACGGCTTATTATATGTGCACAGAAGATGGGGCAGGGCCAACTAGA***GATGAGGTAGACTTTGAGTTTTTGGGA***AATAGAACAGGGGAACCCTATCTTATTCAGACCAATGTGTACAAAAATGGCACTGGTGGGCGTGAGATGAGGCACGTTCTCTGGTTTGACCCTACTGAGGACTTCCATTCCTATTCTCTTCTTTGGAACTCTCACCAACTCGTGTTTTTCGTGGATGAGGTTCCGATAAGGGTATACAAAAACACGAATTATACGAACAATTTCTTCCCTAATGAGAAGCCAATGTACTTGTTTTCGAGCATATGGAATGCAGATGATTGGGCTACTAGGGGTGGTTTGGAGAAAACAGATTGGAAAAATGCACCATTTGTTTCAACATATAAAGATTTTAGTGTAGATGGTTGCCAATGGGAAGATCCTTTTCCTTCTTGTGTTTCAACCACCACTGAAAACTGGTGGGATCAATACAATTCTTGGCATTTATCAAGTGACCAGAAATTGGATTATGCTTGGGTACAAAGAAACCTTGTGATTTATGATTATTGTCAGGATACAGAGAGATATCCAGAAAAGCCTGAGGAGTGTTGGTTAAGTCCCTGGGATTAA

## >NtXTH3 amplification (6 bp deletion)

TTATCAGCTGAGAGCCTCACTCAGTTCCGATTACTCTCCCAAGAGCTTAATTCAAGAAACATTAAGGGAGAGAAAGGCTTCTTCATTA**ATG**GCTAATCTTCTCTTAATTGCAGTTTTAATTGCTATTTATTGTTCACTATCTCAAGCTGAAGTTAAAGGTTCATTTGATGACAACTTTAGTAAAAGTTGTCCTGAATCTCACTTCAAGACTTCTGAAGATGGACAGATCTGGTATCTCTCCTTAGACCACAAAGCAGGATGTGGATTTATGACAAGGCAGAAATACAGATTTGGTTGGTTTAGCATGAAGTTGAAATTGGTAGGAGGTGACTCTGCTGGTGTTGTCACGGCTTATTATATGTGCACAGAAGATGGGGCAGGGCCAACTAGAGATGAGGTAGACTTTGAGTTTTTGGGAAATAGAACAGGGGAACCCTATCTTATTCAGACCAATGTGTACAAAAATGGCACTGGTGGGCGTGAGATGAGGCACGTTCTCTGGTTTGACCCTACTGAGGACTTCCATTCCTATTCTCTTCTTTGGAACTCTCACCAACTCGTGTTTTTCGTGGATGAGGTTCCGATAAGGGTATACAAAAACACGAATTATACGAACAATTTCTTCCCTAATGAGAAGCCAATGTACTTGTTTTCGAGCATATGGAATGCAGATGATTGGGCTACTAGGGGTGGTTTGGAGAAAACAGATTGGAAAAATGCACCATTTGTTTCAACATATAAAGATTTTAGTGTAGATGGTTGCCAATGGGAAGATCCTTTTCCTTCTTGTGTTTCAACCACCACTGAAAACTGGTGGGATCAATACAATTCTTGGCATTTATCAAGTGACCAGAAATTGGATTATGCTTGGGTACAAAGAAACCTTGTGATTTATGATTATTGTCAGGATACAGAGAGATATCCAGAAAAGCCTGAGGAGTGTTGGTTAAGTCCC~~TGGGAT~~**TAA**A

# >NtXTH4 (SGN database sequence)

ATGGAGAGAATGTCTTCTTCAATACCTAAATTCCTTCTAATTATAGCACTAATTACTGTTCTTTTTACATTAACACAAGCTGAAGTACAAGGTTCATTTGATGACAATTTTAGTAAAAGTTGTCCTGAAACACATTTCAAGACTTCTGAAGATGGACAGATCTGGTATCTTTCATTAGATAAGAAAGCAGGATGTGGATTTATGACCAGGCAAAAATATAGATTTGGGTGGTTTAGTATGAAGTTGAAATTGGTGGGAGGTGACTCTGCCGGTGTTGTCACAGCTTACTATATGTGTACAGAAGATGGAGCAGGGCCAACAAGA***GATGAATTAGACTTTGAGTTCTTGGGA***AATAGAACAGGGGAACCTTATACTATTCAAACCAATGTGTACAAAAATGGGACTGGTAATCGTGAGATGAGACACATTCTTTGGTTTGACCCCACCGAGGACTTTCATACTTATTCCATTCTTTGGAACACCCACCAAATTGTGTTTTTCGTGGATAGAGTACCAATAAGGGTATACAAAAATGCGAATTATACGAATAATTTCTTCCCAAATGAGAAGCCAATGTACTTGTTTTCAAGCATATGGAATGCTGACGATTGGGCTACAAGAGGTGGTTTGGAGAAAACAAATTGGAAAAATCAACCATTTGTTTCAAGTTATAAGGATTTTAGTGTAGATGGTTGTCAATGGAAAGATCCATTTCCTGCTTGTGTTTCTACTACCACTAAAAATTGGTGGGATCAATATAATTCTTGGCATTTATCAAGTGACCAAAAAATGGATTATGCTTGGGTACAGAGAAATCTTGTGACTTATGATTATTGCCAAGATACTGAGAGATTTCCTAAAAAGCCTGAGGAATGTTGGTTAAATCCATGGGATTAA

## >NtXTH4 amplification (63 bp insertion)

GGGCGATTGGGCCCTCTAGATGCATGCTCGAGCGGCCGCCAGTGTGATGGATATCTGCAGAATTGCCCTTGTGATCAGTGTACAGCCTTTATTATATTTGTAGTCTTCTCCAAAAATTTCAACTTAAGAAGCTTTCACTTCTCAAGCTCCTCAAGAAAGAGA**ATG**GAGAGAATGTCTTCTTCAATACCTAAATTCCTTCTAATTATAGCACTAATTACTGTTCTTTTTACATTAACACAAGCTGAAGTACAAGGTTCATTTGATGACAATTTTAGTAAAAGTTGTCCTGAAACACATTTCAAGACTTCTGAAGATGGACAGATCTGGTATCTTTCATTAGATAAGAAAGCAGGATGTGGATTTATGACCAGGCAAAAATATAGATTTGGGTGGTTTAGTATGAAGTTGAAATTGGTGGGAGGTGACTCTGCCGGTGTTGTCACAGCTTACTATATGTGTACAGAAGATGGAGCAGGGCCAACAAGAGATGAATTAGACTTTGAGTTCTTGGGAAATAGAACAGGGGAACCTTATACTATTCAAACCAATGTGTACAAAAATGGGACTGGTAATCGTGAGATGAGACACATTCTTTGGTTTGACCCCACCGAGGACTTTCATACTTATTCCATTCTTTGGAACACCCACCAAATTGTGTTTTTCGTGGATAGAGTACCAATAAGGGTATACAAAAATGCGAATTATACGAATAATTTCTTCCCAAATGAGAAGCCAATGTACTTGTTTTCAAGCATATGGAATGCTGACGATTGGGCTACAAGAGGTGGTTTGGAGAAAACAAATTGGAAAAATCAACCATTTGTTTCAAGTTATAAGGATTTTAGTGTAGATGGTTGTCAATGGAAAGATCCATTTCCTGCTTGTGTTTCTACTACCACTAAAAATTGGTGGGATCAATATAATTCTTGGCATTTATCAAGTGACCAAAAAATGGATTATGCTTGGGTACAGAGAAATCTTGTGACTTATGATTATTGCCAAGATACTGAGAGATTTCCTAAAAAGCCTGAGGAATGTTGGTTAAATCCATGGGAT**AGGGCAATTCCAGCACACTGGCGGCCGTTACTAGTGGATCCGAGCTCGGTACCAAGCTTGGCG**TAGCTGTTTCCTGTGTGAAATTGTTATCCGCTCACAATTCCACACAACATACGAGCCGGAAGCATAAAGTGTAAAGCCTGGGGTGCCTAATGAGTGAGCTAACTCACATTAATTGCGTTGCGCTCACT

# >NtXTH5 (SGN database sequence)

ATGGAGAAAATGGCTTCTTCAATACCTAAAATCCTTCTAATTATAGCACTAATTACTGTTCTTTTTTCATTAACACAAGCTGAAGTACAAGGTTCATTTGATGATAATTTTAGTAAAAGTTGTCCTGAAACACATTTCAAGACTTCTGAAGATGGACAGATCTGGTATCTTTCATTAGATAAAAAAGCAGGATGTGGATTTATGACTAAGCAGAAATATAGATTTGGGTGGTTTAGTATGAAGTTGAAATTGGTGGGAGGTGACTCTGCTGGTGTTGTCACAGCTTATTATATGTGCACAGAAGATGGAGCAGGACCAACAAGA***GATGAATTAGACTTTGAGTTCTTGGGA***AATAGAACAGGGGAACCCTATACTATTCAAACCAATGTGTATAAAAATGGGACTGGTAACCGTGAAATGAGACACATTCTATGGTTTGACCCCACTGAGGATTTCCACACTTATTCCATTCTTTGGAACACTCACCAAATTGTGTTTTTCGTGGATAGAGTACCGATAAGGGTATACAAAAATGCGAACTATACGAATAATTTCTTCCCAAATGAGAAGCCAATGTACTTATTTTCAAGCATATGGAATGCTGATGATTGGGCAACAAGAGGTGGTTTGGAGAAAACAAATTGGAAAAATCAACCATTTGTTTCAAGTTATAAAGATTTTAGTGTAGATGGTTGTCAATGGAAAGATCCATTTCCTGCTTGTGTTTCGACAACCACTAAAAATTGGTGGGATCAATATAATTCTTGGCATTTATCAAGTGACCAAAAAATGGATTATGCTTGGGTTCAAAGGAACCTTGTGACTTATGATTATTGCCAAGATACAGAGAGATTTCCTAAAAAGCCTGAGGAATGTTGGTTAAATCCATGGGAATAA

## >NtXTH5 amplification (same with SGN database sequence)

TATTTATAGTTTCTCCAAATATTTCAACTTAAGAAGCTTCACTTCTCAAGCTCCTCAAGAAAAAAGA**ATG**GAGAAAATGGCTTCTTCAATACCTAAAATCCTTCTAATTATAGCACTAATTACTGTTCTTTTTTCATTAACACAAGCTGAAGTACAAGGTTCATTTGATGATAATTTTAGTAAAAGTTGTCCTGAAACACATTTCAAGACTTCTGAAGATGGACAGATCTGGTATCTTTCATTAGATAAAAAAGCAGGATGTGGATTTATGACTAAGCAGAAATATAGATTTGGGTGGTTTAGTATGAAGTTGAAATTGGTGGGAGGTGACTCTGCTGGTGTTGTCACAGCTTATTATATGTGCACAGAAGATGGAGCAGGACCAACAAGAGATGAATTAGACTTTGAGTTCTTGGGAAATAGAACAGGGGAACCCTATACTATTCAAACCAATGTGTATAAAAATGGGACTGGTAACCGTGAAATGAGACACATTCTATGGTTTGACCCCACTGAGGATTTCCACACTTATTCCATTCTTTGGAACACTCACCAAATTGTGTTTTTCGTGGATAGAGTACCGATAAGGGTATACAAAAATGCGAACTATACGAATAATTTCTTCCCAAATGAGAAGCCAATGTACTTATTTTCAAGCATATGGAATGCTGATGATTGGGCAACAAGAGGTGGTTTGGAGAAAACAAATTGGAAAAATCAACCATTTGTTTCAAGTTATAAAGATTTTAGTGTAGATGGTTGTCAATGGAAAGATCCATTTCCTGCTTGTGTTTCGACAACCACTAAAAATTGGTGGGATCAATATAATTCTTGGCATTTATCAAGTGACCAAAAAATGGATTATGCTTGGGTTCAAAGGAACCTTGTGACTTATGATTATTGCCAAGATACAGAGAGATTTCCTAAAAAGCCTGAGGAATGTTGGTTAAATCCATGGGAA**TAA**TAAATTTGAAGATATAGGCAAAaTTTTAATGAGAGGAAGGTATTAATTTTTGAATGAGGAGG

# >NtXTH6(SGN database sequence)

ATGGAGAGAAATGCTTCTTCAATGGCTGATCTTTTCTTCACTGCAGCACTAATGGCTGCACTCTTTTCATCCTCACATGCTGAACTCATCAAAGGTGCATTTGAAAACAACTTTAGTAAAAGTTGTCCTGGTACACATTTCAAGACTTCTCAAGATGGACAGATCTGGTATCTCACCTTAGACCAAATATCAGATTGTGGGTTTATTACTAAGCAGAGTTATAGATTTGGTTGGTTTAGCACAAAGTTGAAATTAGTAGGAGGTGACTCTGCTGGTGTTGTGACAGCCTTTTATATGTGTTCGGAAGTGGAGGCAGGGCCATTGAGA***GATGAGATAGATTTTGAGTTCTTGGGA***AACAGAACAGGACAACCTTATCTTATTCAAACCAATGTGTACAATAATGGCAGTGGTGGACGTGAGATGAGGCATCTTCTTTGGTTTGATCCCACTCAAGACTTCCATACCTATTCCATTCTTTGGAACTCTCACCAAATTGTGTTTTTTGTTGATAAGGTTCCGATAAGGGTATACAAGAACGCGAATCACACGAACAATTTTTTTCCAGCTGAGAGGCCAATGTACGTGTTTTCTAGCATATGGAATGCAGATAATTGGGCTACTAGAGGAGGATTGGACAAGATAAACTGGACAAGTGCACCATTTATAGCAAGTTATAAGGATTTTATTTTAGATGCTTGTCAATGGAAAGATCCTTTCCCTGCTTGTGTTTCCACCACTACACAGCATTGGTGGGATCAATATAATGCTTGGCACCTATCAAGTAAACAGAAGATTGATTATGCTTGGGTGCAGAGAAACTTTGTAGTTTATGATTATTGCCAGGATAGTGTGAGAAACCGTTATAAGCCCCAAGAGTGTTGGTTAAGTGCATTGGACTAA

## >NtXTH6 amplification (same with SGN database sequence)

GAGCTCGGATCcACTAGTAACGGCCGCCAGTGTGCTGGAATTGCCCTTATAAAACGTCGTGTTTCCTAAAGGATCCAGCTGAGAAGCCTTTCTTTTCTCACTTGTAAGAAGAGCTTCATTCGAAAAACA**ATG**GAGAGAAATGCTTCTTCAATGGCTGATCTTTTCTTCACTGCAGCACTAATGGCTGCACTCTTTTCATCCTCACATGCTGAACTCATCAAAGGTGCATTTGAAAACAACTTTAGTAAAAGTTGTCCTGGTACACATTTCAAGACTTCTCAAGATGGACAGATCTGGTATCTCACCTTAGACCAAATATCAGATTGTGGGTTTATTACTAAGCAGAGTTATAGATTTGGTTGGTTTAGCACAAAGTTGAAATTAGTAGGAGGTGACTCTGCTGGTGTTGTGACAGCCTTTTATATGTGTTCGGAAGTGGAGGCAGGGCCATTGAGAGATGAGATAGATTTTGAGTTCTTGGGAAACAGAACAGGACAACCTTATCTTATTCAAACCAATGTGTACAATAATGGCAGTGGTGGACGTGAGATGAGGCATCTTCTTTGGTTTGATCCCACTCAAGACTTCCATACCTATTCCATTCTTTGGAACTCTCACCAAATTGTGTTTTTTGTTGATAAGGTTCCGATAAGGGTATACAAGAACGCGAATCACACGAACAATTTTTTTCCAGCTGAGAGGCCAATGTACGTGTTTTCTAGCATATGGAATGCAGATAATTGGGCTACTAGAGGAGGATTGGACAAGATAAACTGGACAAGTGCACCATTTATAGCAAGTTATAAGGATTTTATTTTAGATGCTTGTCAATGGAAAGATCCTTTCCCTGCTTGTGTTTCCACCACTACACAGCATTGGTGGGATCAATATAATGCTTGGCACCTATCAAGTAAACAGAAGATTGATTATGCTTGGGTGCAGAGAAACTTTGTAGTTTATGATTATTGCCAGGATAGTGTGAGAAACCGTTATAAGCCCCAAGAGTGTTGGTTAAGTGCATTGGAC**TAA**TATTAAAGATAGATAAGATTCTTTTGAGAGAGAAGAAAATTATAGTATTGTAATCTTGATTTTGAGTGTATATATTGGACTCTCTCAAGGGCAATTCTGCAGATATCCATCACACTGGCGGCCGCTCGAGCATGCATCTAGA

# >NtXTH7 (SGN database sequence)

ATGGAGAGAAATATGGGTGATCTTCTCTTATTTGCAGCACTAGTGGCTACCCTTTTTTCATCATCACATGCTCAACTTATCAAAGGTGCATTTGAAAACACCTTCAGTAAAAGCTGTCCGGGTACTCATTTCAAGACTTCTCAAGATGGACAGATCTGGTATCTCACCTTAGACCAAGTATCAGATTGTGGGTTTATCACCAAGCAGAGCTATAGATTTGGTTGGTTTAGCACAAAGTTGAAATTGGTAGGAGGTGACTCTGCTGGTGTTGTGACAGCCTTTTATATGTGCTCGGAAGTAGAGGCAGGACC***ATTGAGAGATGAGATAGATTTTGAGTT***CTTGGGAAACAGAACAGGGCAGCCTTATCTTATTCAGACCAATGTGTACAATAATGGCAGTGGTGGACGTGAGATGAGGCATCTTCTCTGGTTTGACCCTACTCAGGACTTTCATACCTATTCCATTCTTTGGAACTCCCACCAAATTGTGTTTTTCGTTGATAAGGTTCCAATAAGGGTATACAAGAACGCGAATCACACTAACAATTTTTTTCCAGCTGAGAGGCCAATGTACGTGTTTTCTAGCATATGGAATGCAGATAATTGGGCTACTAGAGGAGGGTTGGACAAGATAAACTGGACAAGTGCACCATTCGTAGCAAGTTATAAGGAGTTTACTTTAGATGCTTGTCAATGGAAAGATCCTTTCCCAGCTTGTGTTTCCACCACTACACAGCACTGGTGGGATCAGTATAATGCTTGGCACCTATCAAGTAAACAGAAGATTGATTATACTTGGGTGCAGAGAAACTTTGTAGTTTATGATTATTGCCAGGATAGTGTAAGAAACCGTTACAAGCCTCAAGAGTGTTGGTTAAGTCCATTGGACTAA

## >NtXTH7 amplification (same with SGN database sequence)

AGCTCGGATCACTAGTAACGGCCGCCAGTGTGCTGGAATTGCCCTTAACGTCGTGTTTCCTAAAGGATCCAGCTGAGAAGCCTTTCTTTTCTCACTTGTAAGAAGAGCTTCATTCGAAAAACA**ATG**GAGAGAAATATGGGTGATCTTCTCTTATTTGCAGCACTAGTGGCTACCCTTTTTTCATCATCACATGCTCAACTTATCAAAGGTGCATTTGAAAACACCTTCAGTAAAAGCTGTCCGGGTACTCATTTCAAGACTTCTCAAGATGGACAGATCTGGTATCTCACCTTAGACCAAGTATCAGATTGTGGGTTTATCACCAAGCAGAGCTATAGATTTGGTTGGTTTAGCACAAAGTTGAAATTGGTAGGAGGTGACTCTGCTGGTGTTGTGACAGCCTTTTATATGTGCTCGGAAGTAGAGGCAGGACCATTGAGAGATGAGATAGATTTTGAGTTCTTGGGAAACAGAACAGGGCAGCCTTATCTTATTCAGACCAATGTGTACAATAATGGCAGTGGTGGACGTGAGATGAGGCATCTTCTCTGGTTTGACCCTACTCAGGACTTTCATACCTATTCCATTCTTTGGAACTCCCACCAAATTGTGTTTTTCGTTGATAAGGTTCCAATAAGGGTATACAAGAACGCGAATCACACTAACAATTTTTTTCCAGCTGAGAGGCCAATGTACGTGTTTTCTAGCATATGGAATGCAGATAATTGGGCTACTAGAGGAGGGTTGGACAAGATAAACTGGACAAGTGCACCATTCGTAGCAAGTTATAAGGAGTTTACTTTAGATGCTTGTCAATGGAAAGATCCTTTCCCAGCTTGTGTTTCCACCACTACACAGCACTGGTGGGATCAGTATAATGCTTGGCACCTATCAAGTAAACAGAAGATTGATTATACTTGGGTGCAGAGAAACTTTGTAGTTTATGATTATTGCCAGGATAGTGTAAGAAACCGTTACAAGCCTCAAGAGTGTTGGTTAAGTCCATTGGAC**TAA**TATTAAAGATAGATAAGATTCTTTTGAGAGAGAAGAAAATTATAGTATTGTAATCTTGATTTTGAGTGTATATATTGAAGGGCAATTCTGCAGATATCCATCACACTGGCGGCCGCTCGAGCATGCATCT

# >NtXTH8(SGN database sequence)

ATGAAGCAAGTAATTGAATATCGTTGCCTTCTGATTTTAGGATGTGGGTTTGCTTCCAAAAGCAAATACCTCTTTGGACGTGTTAGCATGAAGATCAAGCTCGTTCCTGGTGACTCTGCTGGAACTGTCACCGCCTTTTACATGAACTCGGACACAGATAACGTAAGG***GACGAGCTAGACTTCGAGTTCTTGGGA***AACAGGTCAGGCCAGCCGTACACTGTCCAAACGAATGTTTATGTCCATGGAAAGGGTGACAAGGAACAAAGGATCAACCTTTGGTTCGATCCATCCGCTGATTTTCATACCTACACCATTCTTTGGAACCACCATCACACTGTATTCTACGTGGACGCAGTACCCATTAGAGTGTACAAGAATAACGAAGCAAAAGGAATCCCATTCCCTAAATTCCAACCCATGGGAGTGTACTCAACATTGTGGGAAGCCGACGACTGGGCAACAAGAGGTGGATTAGAGAAAATAAATTGGAGCAAATCCCCATTTTACGCATACTACAAGGACTTTGACATAGAGGGATGTGCAATGCCAGGACCAGCAAACTGTGCCTCAAATCCACGCAATTGGTGGGAAGGTGCTAATTACCAACAGCTCAGTGCTGTGGAAGCAAGGCAATATCGCTGGGTTAGAACGAACCACATGATCTATGATTATTGCACTGACAAATCCAGAAATCCAGTTCCCCCACCAGAATGTGTGGCCGGAATATGA

## >NtXTH8 amplification (same with SGN database sequence)

**ATG**AAGCAAGTAATTGAATATCGTTGCCTTCTGATTTTAGTGACCAAAACTCAGGATGTGGGTTTGCTTCCAAAAGCAAATACCTCTTTGGACGTGTTAGCATGAAGATCAAGCTCGTTCCTGGTGACTCTGCTGGAACTGTCACCGCCTTTTACATGAACTCGGACACAGATAACGTAAGGGACGAGCTAGACTTCGAGTTCTTGGGAAACAGGTCAGGCCAGCCGTACACTGTCCAAACGAATGTTTATGTCCATGGAAAGGGTGACAAGGAACAAAGGATCAACCTTTGGTTCGATCCATCCGCTGATTTTCATACCTACACCATTCTTTGGAACCACCATCACACTGTATTCTACGTGGACGCAGTACCCATTAGAGTGTACAAGAATAACGAAGCAAAAGGAATCCCATTCCCTAAATTCCAACCCATGGGAGTGTACTCAACATTGTGGGAAGCCGACGACTGGGCAACAAGAGGTGGATTAGAGAAAATAAATTGGAGCAAATCCCCATTTTACGCATACTACAAGGACTTTGACATAGAGGGATGTGCAATGCCAGGACCAGCAAACTGTGCCTCAAATCCACGCAATTGGTGGGAAGGTGCTAATTACCAACAGCTCAGTGCTGTGGAAGCAAGGCAATATCGCTGGGTTAGAACGAACCACATGATCTATGATTATTGCACTGACAAATCCAGAAATCCAGTTCCCCCACCAGAATGTGTGGCCGGAATA**TGA**AACTTTACATCAGCTGTCCATTATTTGTATACATATTAAATGGTGCATATAATTGGAGCGT

# >NtXTH9 (SGN database sequence)

ATGATTTCCTCTTCTTTAAAATATTCAACTGTCATTCCAATCTTGCTATATGCCTTGACCTTTTCTTCCTCAGTAAGTGCACGACCCGCCACTTTTTTACAGGACTTTAAAGTGGCATGGGCTGACTCTCACATCAAGCAAATCGATGGCGGCAAGGCTATTCAGCTTATACTCGACCAAAACTCAGGATGTGGGTTTGCTTCCAAAAGCAAATACCTCTTTGGACGTGTTAGCATGAAGATCAAGCTCGTTCCTGGTGACTCTGCTGGAACTGTCACTGCCTTTTACATGAACTCGGACACAGATAATGTAAGG***GACGAGCTAGACTTCGAGTTCTTGGGA***AACAGGTCAGGCCAGCCGTACACTGTCCAAACGAATGTTTATGTCCATGGAAAGGGTGACAAGGAACAAAGGATCAACCTTTGGTTCGATCCATCCGCTGATTTTCATACCTACACCATACTTTGGAACCACCATCACACTGTATTCTACGTAGACGCAGTACCAATTAGAGTGTACAAGAACAACGAAGCAAAAGGAATCCCATTCCCCAAATTCCAACCCATGGGAGTTTATTCAACATTATGGGAAGCCGACGACTGGGCAACGAGAGGTGGATTAGAGAAAATAAATTGGAGCAGATCCCCATTTTACGCATATTACAAGGACTTTGACATAGAGGGATGTGCAATGCCAGGACCAGCAAACTGTGCCTCAAATCCTCGCAATTGGTGGGAAGGAGCTAATTACCAACAACTCAGTGCTGTGGAAGCAAAGCAATATCGCTGGGTTAGAATGAACCACATGATCTATGATTATTGCACTGACAAATCCAGAAATCCAGTAACCCCACCAGAATGTGTGGCCGGAATATGA

## >NtXTH9 amplification (same with SGN database sequence)

AATTGTATAATCATATATACAATTCTGATTGGTTTCTTTAATAGATAAA**ATG**ATTTCCTCTTCTTTAAAATATTCAACTGTCATTCCAATCTTGCTATATGCCTTGACCTTTTCTTCCTCAGTAAGTGCACGACCCGCCACTTTTTTACAGGACTTTAAAGTGGCATGGGCTGACTCTCACATCAAGCAAATCGATGGCGGCAAGGCTATTCAGCTTATACTCGACCAAAACTCAGGATGTGGGTTTGCTTCCAAAAGCAAATACCTCTTTGGACGTGTTAGCATGAAGATCAAGCTCGTTCCTGGTGACTCTGCTGGAACTGTCACTGCCTTTTACATGAACTCGGACACAGATAATGTAAGGGACGAGCTAGACTTCGAGTTCTTGGGAAACAGGTCAGGCCAGCCGTACACTGTCCAAACGAATGTTTATGTCCATGGAAAGGGTGACAAGGAACAAAGGATCAACCTTTGGTTCGATCCATCCGCTGATTTTCATACCTACACCATACTTTGGAACCACCATCACACTGTATTCTACGTAGACGCAGTACCAATTAGAGTGTACAAGAACAACGAAGCAAAAGGAATCCCATTCCCCAAATTCCAACCCATGGGAGTTTATTCAACATTATGGGAAGCCGACGACTGGGCAACGAGAGGTGGATTAGAGAAAATAAATTGGAGCAGATCCCCATTTTACGCATATTACAAGGACTTTGACATAGAGGGATGTGCAATGCCAGGACCAGCAAACTGTGCCTCAAATCCTCGCAATTGGTGGGAAGGAGCTAATTACCAACAACTCAGTGCTGTGGAAGCAAAGCAATATCGCTGGGTTAGAATGAACCACATGATCTATGATTATTGCACTGACAAATCCAGAAATCCAGTAACCCCACCAGAATGTGTGGCCGGAATA**TGA**AACTTTACATCAACTGTCCATTATTTGTATACATAT

# >NtXTH10 (SGN database sequence)

ATGGGCAAATTGACGTCCTTAAAATATTCAGCTGCAATTCTAATATTGCTATATGCCTTGACCTTTTCCTTCTCAGTGAGTGCACGACCCGCCACTTTTCTACAGGACTTTAAGGTCTCTTGGGCCTACTCTCACATCAAACAAATCGATGGCGGCAGGGCCATTCAGCTTATTCTCGACCAAAACTCAGGATGTGGGTTTGCTTCCAAAAGCAAATACCTCTTTGGACGTGTTAGCATGAAGATCAAGCTCGTGCCTGGTGACTCTGCTGGAACCGTCACCGCCTTTTACATGAATTCGGACACAGACAACGTAAGG***GACGAGCTAGACTTCGAGTTCTTGGGA***AACAGGTCAGGCCAGCCGTACACTGTCCAGACGAATGTTTATGTTCATGGAAAAGGTGACAAGGAACAAAGGGTCAACCTTTGGTTCGATCCATCCGCTGATTTTCACACTTATACCATTCTTTGGAACCACCACCACGCCGTATTCTACGTGGACGCAGTACCCATTAGAGTGTACAAGAACAACGAAGCAAAAGGAATCCCATTCCCCAAATTCCAACCCATGGGAGTGTATTCCACATTGTGGGAAGCCGATGACTGGGCAACGAGAGGTGGATTAGAGAAAATAAATTGGAGCAAATCCCCATTTTACGCATACTACAAGGACTTTGACATAGAGGGATGTGCAATGCCAGGACCAGCAAACTGTGCCTCAAATCCACGCAATTGGTGGGAAGGAGCTAATTACCAACAACTCAGTGCTGTGGAAGCAAGGCAATATCGCTGGGTTAGAATGAACCACATGATCTATGATTATTGCACTGACAAATCCAGAAATCCAGTCACCCCACCAGAATGTGTGGCCGGAATATGA

## >NtXTH10 amplification (same with SGN database sequence)

TCCAATTGTATAATATCATACAATTAACAGAAAAACA**ATG**GGCAAATTGACGTCCTTAAAATATTCAGCTGCAATTCTAATATTGCTATATGCCTTGACCTTTTCCTTCTCAGTGAGTGCACGACCCGCCACTTTTCTACAGGACTTTAAGGTCTCTTGGGCCTACTCTCACATCAAACAAATCGATGGCGGCAGGGCCATTCAGCTTATTCTCGACCAAAACTCAGGATGTGGGTTTGCTTCCAAAAGCAAATACCTCTTTGGACGTGTTAGCATGAAGATCAAGCTCGTGCCTGGTGACTCTGCTGGAACCGTCACCGCCTTTTACATGAATTCGGACACAGACAACGTAAGGGACGAGCTAGACTTCGAGTTCTTGGGAAACAGGTCAGGCCAGCCGTACACTGTCCAGACGAATGTTTATGTTCATGGAAAAGGTGACAAGGAACAAAGGGTCAACCTTTGGTTCGATCCATCCGCTGATTTTCACACTTATACCATTCTTTGGAACCACCACCACGCCGTATTCTACGTGGACGCAGTACCCATTAGAGTGTACAAGAACAACGAAGCAAAAGGAATCCCATTCCCCAAATTCCAACCCATGGGAGTGTATTCCACATTGTGGGAAGCCGATGACTGGGCAACGAGAGGTGGATTAGAGAAAATAAATTGGAGCAAATCCCCATTTTACGCATACTACAAGGACTTTGACATAGAGGGATGTGCAATGCCAGGACCAGCAAACTGTGCCTCAAATCCACGCAATTGGTGGGAAGGAGCTAATTACCAACAACTCAGTGCTGTGGAAGCAAGGCAATATCGCTGGGTTAGAATGAACCACATGATCTATGATTATTGCACTGACAAATCCAGAAATCCAGTCACCCCACCAGAATGTGTGGCCGGAATA**TGA**AACTTTACATCAACTGTTAATTATTTATACAT

# >NtXTH11(SGN database sequence)

ATGGCCAGATTGACTTCCTTAAAATATTCAGCTGCAATTCTAATATTGCTATATGCCTTGACCTTTTCATTCTCAGTGAGTGCACGACCCGCCACTTTTTTACAGGACTTTAAGGTCTCTTGGTCCGACTCTCACATCAAACAAATTGATGGTGGCAGGGCCATTCAGCTTATTCTCGACCAAAACTCAGGATGTGGGTTTGCTTCCAAAAGCAAATACCTCTTTGGACGTGTTAGCATGAAGATCAAGCTCGTACCTGGTGACTCTGCTGGAACCGTCACTGCCTTTTACATGAACTCGGACACAGACAACGTAAGG***GACGAACTAGACTTCGAGTTCTTGGGA***AACCGGTCAGGCCAGCCGTATACTGTCCAGACGAATGTTTATGTTCATGGAAAAGGTGACAAGGAACAAAGAGTCAACCTTTGGTTCGATCCATCCGCTGATTTTCACACTTATACCATTCTTTGGAACCACCACCACGCCGTATTCTACGTGGATGCGGTACCCATTAGAGTCTACAAGAACAACGAAGCAAAAGGAATTCCATTCCCCAAATTCCAACCCATGGGAGTGTACTCAACATTGTGGGAAGCCGACGACTGGGCAACGAGAGGTGGATTAGAGAAAATAAATTGGAGCAAATCCCCATTTTACGCATATTACAAGGACTTTGACATAGAGGGATGTGCAATGCCAGGACCAGCAAACTGTGCCTCAAATCCTCGCAATTGGTGGGAAGGAGCTAATTACCAACAACTCAGTGCTGCGGAAGCAAGGCAATATCGCTGGGTTAGAATGAACCACATGATCTATGATTATTGCACCGACAAATCCAGAAATCCAGTCACCCCACCAGAATGTGTGGCTGGAATATGA

## >NtXTH11 amplification (same with SGN database sequence)

TTCTTTTAAGCAAAAGCAATTTTCTGAAAATTTCACCATTTACGAACGATAGCCATGGTACCA**ATG**GCCAGATTGACTTCCTTAAAATATTCAGCTGCAATTCTAATATTGCTATATGCCTTGACCTTTTCATTCTCAGTGAGTGCACGACCCGCCACTTTTTTACAGGACTTTAAGGTCTCTTGGTCCGACTCTCACATCAAACAAATTGATGGTGGCAGGGCCATTCAGCTTATTCTCGACCAAAACTCAGGATGTGGGTTTGCTTCCAAAAGCAAATACCTCTTTGGACGTGTTAGCATGAAGATCAAGCTCGTACCTGGTGACTCTGCTGGAACCGTCACTGCCTTTTACATGAACTCGGACACAGACAACGTAAGGGACGAACTAGACTTCGAGTTCTTGGGAAACCGGTCAGGCCAGCCGTATACTGTCCAGACGAATGTTTATGTTCATGGAAAAGGTGACAAGGAACAAAGAGTCAACCTTTGGTTCGATCCATCCGCTGATTTTCACACTTATACCATTCTTTGGAACCACCACCACGCCGTATTCTACGTGGATGCGGTACCCATTAGAGTCTACAAGAACAACGAAGCAAAAGGAATTCCATTCCCCAAATTCCAACCCATGGGAGTGTACTCAACATTGTGGGAAGCCGACGACTGGGCAACGAGAGGTGGATTAGAGAAAATAAATTGGAGCAAATCCCCATTTTACGCATATTACAAGGACTTTGACATAGAGGGATGTGCAATGCCAGGACCAGCAAACTGTGCCTCAAATCCTCGCAATTGGTGGGAAGGAGCTAATTACCAACAACTCAGTGCTGCGGAAGCAAGGCAATATCGCTGGGTTAGAATGAACCACATGATCTATGATTATTGCACCGACAAATCCAGAAATCCAGTCACCCCACCAGAATGTGTGGCTGGAATAGGATCCGGCGCGGCCGCAGGCATGGTGAGCAAGGGC

# >NtXTH12 (SGN database sequence)

ATGGTGTCTTTTCCTATGGAATTTAAGTGGGTTTTCTTGGGTATTTCTCTAATGTTGGTTGGTTTGGTTAGCTCCTCAAGATTTGAGGAACTATATCAGCCCAGCTGGGCAACAGACCATTTGACAAATGAAGGAGAAATTCTCAGGATGAAATTGGACAACCTTTCTGGTGCTGGATTTTCATCAAAGAACAAGTATATGTTTGGGAAAGTTACTGTTCAGATTAAGCTTGTAGAGGGTGACTCTGCTGGAACTGTCACTGCTTTCTACATGTCATCAGAGGGACCAACCCAC***AATGAGTTTGATTTTGAGTTTCTAGGC***AACACTACTGGTGAACCATACTCTGTACAAACCAATGTGTACGTAAATGGCGTGGGTAACAGAGAACAAAGATTAAACCTTTGGTTCGACCCATCCAATGAATTCCACTCCTATTCCATCTTGTGGAACCAACACCGAGTTGTATTTTTAGTAGATGAAACACCAGTTCGTGTGCATTCGAATTTGGAGCACAAGGGAATCCCATTTCCAAAGGACCAAGCCATGGGTGTGTACAGTTCAATATGGAATGCAGATGATTGGGCTACACAAGGCGGAAGGGTCAAGACTGATTGGTCACATGCACCCTTTATTGCATCCTACAGAGGATTTGAGATTGATGGCTGTGAATGTCCAGCAACTGTTGCAGCTGCTGAGAATTCTAAGCGGTGCAGCAGCAGTGCTGAGAAAAGGTATTGGTGGGACGAACCAACAATGTCTGAGCTGAGTCTGCACCAGAGCCATCAGTTGATTTGGGTCAGGGCTAACCATATGGTCTATGATTATTGCACAGACACTGCTAGGTTCCCTGTTGCTCCGGTTGAGTGCCAGCACCACCAGCACAAGACTCGCAACTAG

## >NtXTH12 amplification (same with SGN database sequence)

GAGCTCGGATCACTAGTAACGGCCGCCAGTGTGCTGGAATTGCCCTTATAAAAGCCACTCCACTCCCTTTGGCCCTCTTCAGATTACGTCTGAGCACTCTTAATCTAAGCTTCTTTATCTTTTCTTTGAAATTCTTCTGAAAA**ATG**GTGTCTTTTCCTATGGAATTTAAGTGGGTTTTCTTGGGTATTTCTCTAATGTTGGTTGGTTTGGTTAGCTCCTCAAGATTTGAGGAACTATATCAGCCCAGCTGGGCAACAGACCATTTGACAAATGAAGGAGAAATTCTCAGGATGAAATTGGACAACCTTTCTGGTGCTGGATTTTCATCAAAGAACAAGTATATGTTTGGGAAAGTTACTGTTCAGATTAAGCTTGTAGAGGGTGACTCTGCTGGAACTGTCACTGCTTTCTACATGTCATCAGAGGGACCAACCCACAATGAGTTTGATTTTGAGTTTCTAGGCAACACTACTGGTGAACCATACTCTGTACAAACCAATGTGTACGTAAATGGCGTGGGTAACAGAGAACAAAGATTAAACCTTTGGTTCGACCCATCCAATGAATTCCACTCCTATTCCATCTTGTGGAACCAACACCGAGTTGTATTTTTAGTAGATGAAACACCAGTTCGTGTGCATTCGAATTTGGAGCACAAGGGAATCCCATTTCCAAAGGACCAAGCCATGGGTGTGTACAGTTCAATATGGAATGCAGATGATTGGGCTACACAAGGCGGAAGGGTCAAGACTGATTGGTCACATGCACCCTTTATTGCATCCTACAGAGGATTTGAGATTGATGGCTGTGAATGTCCAGCAACTGTTGCAGCTGCTGAGAATTCTAAGCGGTGCAGCAGCAGTGCTGAGAAAAGGTATTGGTGGGACGAACCAACAATGTCTGAGCTGAGTCTGCACCAGAGCCATCAGTTGATTTGGGTCAGGGCTAACCATATGGTCTATGATTATTGCACAGACACTGCTAGGTTCCCTGTTGCTCCGGTTGAGTGCCAGCACCACCAGCACAAGACTCGCAAC**TAG**GCCACATCTGTTGCTCTGCTTGGGAATTGTGGTTCAGTCTTGCACTGTATAAAAGATTAAAAAAAAAGAAAGACATGAACTCCATGTTGTTACATAATTAAGGGCAATTCTGCAGATATCCATCACACTGGCGGCCGCTCGAGCATGCATCTAGA

# >NtXTH13 (SGN database sequence)

ATGGTGTCTTTTCCTATGGAATTTAAGTGTGTTTTCTTGGGTATTTCTCTAATTATGGTGGGTTTGGTTAGCTCCTCAAGATTTGAGGAGCTATATCAGCCCAGCTGGGCGACAGACCATTTGACAAATGAAGGAGAAATCCTCAGGATGAAACTTGACAACCTTTCTGGCGCTGGATTTTCATCAAAGAACAAGTATATGTTTGGGAAAGTTACTGTTCAGATTAAGCTTGTAGAGGGTGACTCTGCTGGAACTGTCACTGCTTTCTACATGTCATCAGAGGGACCAACCCAC***AATGAGTTTGATTTTGAGTTTCTCGGT***AACACTACTGGTGAACCCTACTCTGTGCAGACCAATGTGTACGTAAATGGTGTGGGTAACAGAGAGCAACGACTGAACCTTTGGTTCGACCCATCCAAGGAATTCCACTCATATTCCATCTTGTGGAACCAACGCCGAGTTGTATTTTTAGTAGACGACACACCAATTCGTGTGCACTCAAATTTGGAGCACAAGGGAATACCATTTCCCAAGGACCAAGCCATGGGTGTGTACAGTTCAATATGGAATGCTGATGATTGGGCTACACAAGGTGGAAGGGTTAAGACTGATTGGTCACATGCACCCTTTATTGCATCCTACAGAGGATTTGAGATTGATGGCTGTGAATGCCCAGCAACTGTTGCAGCTGCTGAAAATTCTAAGCGGTGCAGCAGCAGTGCGGTGAAAAGGTATTGGTGGGACGAACCCGTTATGTCCGAACTGAGTCTGCACCAGAGCCACCAGCTGATTTGGGTTAGGGCTAACCATATGGTCTATGATTACTGCACAGACACTGCTCGGTTCCCTGTTGCACCGGTTGAGTGCCAGCACCACCAGCACAAGTTTCATAACTAG

## >NtXTH13 amplification (same with SGN database sequence)

CTTAGCACTTAATCTAAGCTGCTCTCTCTTTTCTTTGAAATTCTTGTGAAAA**ATG**GTGTCTTTTCCTATGGAATTTAAGTGTGTTTTCTTGGGTATTTCTCTAATTATGGTGGGTTTGGTTAGCTCCTCAAGATTTGAGGAGCTATATCAGCCCAGCTGGGCGACAGACCATTTGACAAATGAAGGAGAAATCCTCAGGATGAAACTTGACAACCTTTCTGGCGCTGGATTTTCATCAAAGAACAAGTATATGTTTGGGAAAGTTACTGTTCAGATTAAGCTTGTAGAGGGTGACTCTGCTGGAACTGTCACTGCTTTCTACATGTCATCAGAGGGACCAACCCACAATGAGTTTGATTTTGAGTTTCTCGGTAACACTACTGGTGAACCCTACTCTGTGCAGACCAATGTGTACGTAAATGGTGTGGGTAACAGAGAGCAACGACTGAACCTTTGGTTCGACCCATCCAAGGAATTCCACTCATATTCCATCTTGTGGAACCAACGCCGAGTTGTATTTTTAGTAGACGACACACCAATTCGTGTGCACTCAAATTTGGAGCACAAGGGAATACCATTTCCCAAGGACCAAGCCATGGGTGTGTACAGTTCAATATGGAATGCTGATGATTGGGCTACACAAGGTGGAAGGGTTAAGACTGATTGGTCACATGCACCCTTTATTGCATCCTACAGAGGATTTGAGATTGATGGCTGTGAATGCCCAGCAACTGTTGCAGCTGCTGAAAATTCTAAGCGGTGCAGCAGCAGTGCGGTGAAAAGGTATTGGTGGGACGAACCCGTTATGTCCGAACTGAGTCTGCACCAGAGCCACCAGCTGATTTGGGTTAGGGCTAACCATATGGTCTATGATTACTGCACAGACACTGCTCGGTTCCCTGTTGCACCGGTTGAGTGCCAGCACCACCAGCACAAGTTTCATAAC**TAG**TCAACAT

# >NtXTH14(SGN database sequence)

ATGCCATCCTCTATGATTGTCTTTTTGATCCTAGCTATGCTACTAAACACAGGAGTTGGTGTCAACTTCGCCGAAGTTTTCGAGTCGAGTTGGGCACCTGACCATATTACTGTGGTAGGAGACCAAGTTATGCTCACCCTTGACAATGCTTCTGGCTGCGGGTTTCAGTCGAAGAACAAATATTTGTTCGGGAAAGCCAGCGTGCAGATCAAACTAGTTGGAGGAGATTCAGCTGGAACAGTCATTGCTTTTTATATGTCTTCGGAGGGAGCTAATCACGACGAATTGGACTTTGAGTTTCTTGG***GAATGTTTCAGGAGAACCATACCTAGT***ACAAACAAATGTGTACGCGAATGGCACCGGAGACAGAGAGCAGAGGCATAGTCTCTGGTTCGATCCAACAACGGACTTTCACACTTACTCTTTCTTCTGGAATCATCATACCATTATCTTTTCAGTTGATGACATTCCTATTAGAGTGTTCCAAAACAAGGAGAACAAAGGCGTGGCATACCCGAAAAATCAAGGCATGGGAATTTATGGATCATTGTGGAATGCAGATGATTGGGCTACACAAGGAGGGAGAGTGAAGACCAACTGGAGCCACTCTCCATTTGTTGCAACATTTCGAGCGTTCGAGATCGACGCTTGTGATTTGTCTGGTGAGGACACAGTTGCTGCCGGTGCAAAATGCGGCAAGTTAGCAGAATGCTGGTGGGATAAGCCAGCTGTGAAGCAGCTGAACAAGAGCAAAAAGCGCCAATTCAAAATGGTTCAATCTAAGCACTTGGTCTATGATTATTGTAAGGATACTGCAAGATTCACTCAAATGCCTAAAGAATGCTTGGACTAG

## >NtXTH14.1 amplification (same with SGN database sequence)

CTAGATGCATGCTCGAGCGGCCGCCAGTGTGATGGATATCTGCAGAATTGCCCTTTAAAGTGCCTCCAAGTTCTTGGTTGGCGCTTTTCCTAATAGACCAATGGACTTGAACCTTCTACTTCTTGACCCTAAAATTAAATAGCAATGCCATCCTCTATGATTGTCTTTTTGATCCTAGCTATGCTACTAAACACAGGAGTTGGTGTCAACTTCGCCGAAGTTTTCGAGTCGAGTTGGGCACCTGACCATATTACTGTGGTAGGAGACCAAGTTATGCTCACCCTTGACAATGCTTCTGGCTGCGGGTTTCAGTCGAAGAACAAATATTTGTTCGGGAAAGCCAGCGTGCAGATCAAACTAGTTGGAGGAGATTCAGCTGGAACAGTCATTGCTTTTTATATGTCTTCGGAGGGAGCTAATCACGACGAATTGGACTTTGAGTTTCTTGGGAATGTTTCAGGAGAACCATACCTAGTACAAACAAATGTGTACGCGAATGGCACCGGAGACAGAGAGCAGAGGCATAGTCTCTGGTTCGATCCAACAACGGACTTTCACACTTACTCTTTCTTCTGGAATCATCATACCATTATCTTTTCAGTTGATGACATTCCTATTAGAGTGTTCCAAAACAAGGAGAACAAAGGCGTGGCATACCCGAAAAATCAAGGCATGGGAATTTATGGATCATTGTGGAATGCAGATGATTGGGCTACACAAGGAGGGAGAGTGAAGACCAACTGGAGCCACTCTCCATTTGTTGCAACATTTCGAGCGTTCGAGATCGACGCTTGTGATTTGTCTGGTGAGGACACAGTTGCTGCCGGTGCAAAATGCGGCAAGTTAGCAGAATGCTGGTGGGATAAGCCAGCTGTGAAGCAGCTGAACAAGAGCAAAAAGCGCCAATTCAAAATGGTTCAATCTAAGCACTTGGTCTATGATTATTGTAAGGATACTGCAAGATTCACTCAAATGCCTAAAGAATGCTTGGACTAGACCAACAATTCAGACAGTTCGGCAAGCAACATGTTAATCAACGTCTAAATATGTTTATGTCTCGCACAATTTTAGTAAGGGCAATTCCAGCACACTGGCGGCCGTTACTAGTGATCCGAGCTCG

## >NtXTH14.2 amplification (3 fragments insertion)

GAGCTCGGATCcACTAGTAACGGCCGCCAGTGTGCTGGAATTGCCCTTTAAAGTGCCTCCAAGTTCTTGGTTGGCGCTTTTCCTAATAGACCAATGGACTTGAACCTTCTACTTCTTGACCCTAAAATTAAATAGCAATGCCATCCTCTATGATTGTCTTTTTGATCCTAGCTATGCTACTAAACACAGGAGTTGGTGTCAACTTCGCCGAAGTTTTCGAGTCGAGTTGGGCACCTGACCATATTACTGTGGTAGGAGACCAAGTTATGCTCACCCTTGACAATGCTTCTGG**TGGTTACCAATATGTCTCACTTCATTTCTACTTACTCTCTCTTCCTAGTCTTTTATTATATTTTCTGTGTTTTCAAGGTAATATAATTATATAACCATTTGATATATTATGTTGATATTTACAAAGG**CTGCGGGTTTCAGTCGAAGAACAAATATTTGTTCGGGAAAGCCAGCGTGCAGATCAAACTAGTTGGAGGAGATTCAGCTGGAACAGTCATTGCTTTTTAT**GTACGTAACCAAACCCAAACAAAATATTCATCAAGAGTACTCTTACATTGTTTCTTCAAACTATAACCAACATTATTGGAATTAATATGTGGTTGCAG**ATGTCTTCGGAGGGAGCTAATCACGACGAATTGGACTTTGAGTTTCTTGGGAATGTTTCAGGAGAACCATACCTAGTACAAACAAATGTGTACGCGAATGGCACCGGAGACAGAGAGCAGAGGCATAGTCTCTGGTTCGATCCAACAACGGACTTTCACACTTACTCTTTCTTCTGGAATCATCATACCATTAT**GTAAGTAGTTCTTACTTCAACCATCTCTAAATGTGTTTGATCTTTCCTTTAAATAATTTTAGAGGGTTGCAACTTTGACAATCAAGTAAATTTCTATAACGAGTTGGTAACCTGCATTGTCGGTGTATATAAGTTAAACCTCAACTAATAAAGAAGAATTTTGAATTTTTGTAG**CTTTTCAGTTGATGACATTCCTATTAGAGTGTTCCAAAACAAGGAGAACAAAGGCGTGGCATACCCGAAAAATCAAGGCATGGGAATTTATGGATCATTGTGGAATGCAGATGATTGGGCTACACAAGGAGGGAGAGTGAAGACCAACTGGAGCCACTCTCCATTTGTTGCAACATTTCGAGCGTTCGAGATCGACGCTTGTGATTTGTCTGGTGAGGACACAGTTGCTGCCGGTGCAAAATGCGGCAAGTTAGCAGAATGCTGGTGGGATAAGCCAGCTGTGAAGCAGCTGAACAAGAGCAAAAAGCGCCAATTCAAAATGGTTCAATCTAAGCACTTGGTCTATGATTATTGTAAGGATACTGCAAGATTCACTCAAATGCCTAAAGAATGCTTGGACTAGACCAACAATTCAGACAGTTCGGCAAGCAACATGTTAATCAACGTCTAAATATGTTTATGTCTCGCACAATTTTAGTAAGGGCAATTCTGCAGATATCCATCACACTGGCGGCCGCTCGAGCATGCA

>XTH15-5 pinjie

# >NtXTH15(SGN database sequence)

ATGAGAAGAAAAAGCTGCATGCTGACGACAGTGCCATGGCTGCCACTAAAACATTCTCTTGCTCGCTGGGTTGGTGTCAACTTCACCGAAGTTTTCGAGTCGAGTTGGTCACCTGACCATATTACTGTGGTAGGAGACCAAGTTATGCTCACCCTTGACAATGCTTCTGGCTGCGGGTTTCAGTCGAAGAACAAATATATGTTTGGGAAAGCCAGCGCGCAGATCAAACTAGTTGATGGAGATTCAGCTGGAACAGTCATTGCTTTTTATATGTCATCAGAGGGAGCTAATCAC***GACGAACTGGACTTTGAGTTTCTAGGG***AATGTTTCAGGAGAACCATACCTAGTACAAACAAATGTGTACGCGAATGGCACCGGAGACAGAGAGCAGAGGCATAGTCTTTGGTTCGATCCAACTGCGGATTTCCACACTTACTCTTTCTTTTGGAATCATCATACCATTATCTTTTCGGTTGATGACATTCCTATTAGAGTGTTCAAAAACACAGAGAAAAAAGGCGTGGCATACCCGAAAAATCAAGGCATGGGAGTTTATGGATCGTTGTGGAATGCAGATGACTGGGCTACACAAGGAGGGAGAGTGAAGACCAACTGGAGCCACTCTCCATTTGTTGCAACATTTCGAGCGTTCGAGATTGATGCTTGTGATTTGTCTGGTGAGGACACAGTTGCTGCAGGCGCAAAATGTGGCAAGTTAGCACAATGCTGGTGGGATAAGCCAGCCATGAGGGAGCTGAACAAGAGCAAAAAGCGCCAATTCAAAATGGTTCAATCTAAGCACTTGGTCTATGATTATTGTAAGGATACTGCAAGATTCACTCAAATGCCTAAAGAATGCTTGGACTAG

## >NtXTH15.1 amplification (same with SGN database sequence)

GAGCTCGGATCCACTAGTAACGGCCGCCAGTGTGCTGGAATTGCCCTTATGTTTGAACAAAAGAATCTGTTATCAAAACTGGTAGTTAGTGGAGTGCAGCAACGAATCAGTTCATCTGGTAAAAAAGAATATAGTAAACAAAACATGAGAAGAAAAAGCTGCATGCTGACGACAGTGCCATGGCTGCCACTAAAACATTCTCTTGCTCGCTGGGTTGGTGTCAACTTCACCGAAGTTTTCGAGTCGAGTTGGTCACCTGACCATATTACTGTGGTAGGAGACCAAGTTATGCTCACCCTTGACAATGCTTCTGGCTGCGGGTTTCAGTCGAAGAACAAATATATGTTTGGGAAAGCCAGCGCGCAGATCAAACTAGTTGATGGAGATTCAGCTGGAACAGTCATTGCTTTTTATATGTCATCAGAGGGAGCTAATCACGACGAACTGGACTTTGAGTTTCTAGGGAATGTTTCAGGAGAACCATACCTAGTACAAACAAATGTGTACGCGAATGGCACCGGAGACAGAGAGCAGAGGCATAGTCTTTGGTTCGATCCAACTGCGGATTTCCACACTTACTCTTTCTTTTGGAATCATCATACCATTATCTTTTCGGTTGATGACATTCCTATTAGAGTGTTCAAAAACACAGAGAAAAAAGGCGTGGCATACCCGAAAAATCAAGGCATGGGAGTTTATGGATCGTTGTGGAATGCAGATGACTGGGCTACACAAGGAGGGAGAGTGAAGACCAACTGGAGCCACTCTCCATTTGTTGCAACATTTCGAGCGTTCGAGATTGATGCTTGTGATTTGTCTGGTGAGGACACAGTTGCTGCAGGCGCAAAATGTGGCAAGTTAGCACAATGCTGGTGGGATAAGCCAGCCATGAGGGAGCTGAACAAGAGCAAAAAGCGCCAATTCAAAATGGTTCAATCTAAGCACTTGGTCTATGATTATTGTAAGGATACTGCAAGATTCACTCAAATGCCTAAAGAATGCTTGGACTAGAGTTCGGCAAGCAACGTGTTGTTCAATGTCTAAATATGTTTATGTCTCGCACAATTTTTGTTTCCCAAATTTGTTGGTCCAATTTGCTGATCTTATTTAAGGGCAATTCTGCAGATATCCATCACACTGGCGGCCGCTCGAGCATGCATCT

## >NtXTH15.2 amplification (1 fragment insertion)

GAGCTCGGATCCACTAGTAACGGCCGCCAGTGTGCTGGAATTGCCCTTATGTTTGAACAAAAGAATCTGTTATCAAAACTGGTAGTTAGTGGAGTGCAGCAACGAATCAGTTCATCTGGTAAAAAAGAATATAGTAAACAAAACATGAGAAGAAAAAGCTGCATGCTGACGACAGTGCCATGGCTGCCACTAAAACATTCTCTTGCTCGCT**GTAATCCATGATCCTAATTTAAACAATCCAGTCCATAAATATATGTTAATACTAACTTATATATAAAACTAAAGTGCCTCCAAGTTCTTGATTGGCGTTTTTCCTAATAGACCAATATAGACTTGAACCTTTTACTTCTAGACCCTAAAATTAATTGGTTTCAAGTTCCAGACAGCAATGTTATCCTCTATGATTGTCTTTTTGATCCTAGCTATGTTACTAAACACAG**GGGTTGGTGTCAACTTCACCGAAGTTTTCGAGTCGAGTTGGTCACCTGACCATATTACTGTGGTAGGAGACCAAGTTATGCTCACCCTTGACAATGCTTCTGGCTGCGGGTTTCAGTCGAAGAACAAATATATGTTTGGGAAAGCCAGCGCGCAGATCAAACTAGTTGATGGAGATTCAGCTGGAACAGTCATTGCTTTTTATATGTCATCAGAGGGAGCTAATCACGACGAACTGGACTTTGAGTTTCTAGGGAATGTTTCAGGAGAACCATACCTAGTACAAACAAATGTGTACGCGAATGGCACCGGAGACAGAGAGCAGAGGCATAGTCTTTGGTTCGATCCAACTGCGGATTTCCACACTTACTCTTTCTTTTGGAATCATCATACCATTATCTTTTCGGTTGATGACATTCCTATTAGAGTGTTCAAAAACACAGAGAAAAAAGGCGTGGCATACCCGAAAAATCAAGGCATGGGAGTTTATGGATCGTTGTGGAATGCAGATGACTGGGCTACACAAGGAGGGAGAGTGAAGACCAACTGGAGCCACTCTCCATTTGTTGCAACATTTCGAGCGTTCGAGATTGATGCTTGTGATTTGTCTGGTGAGGACACAGTTGCTGCAGGCGCAAAATGTGGCAAGTTAGCACAATGCTGGTGGGATAAGCCAGCCATGAGGGAGCTGAACAAGAGCAAAAAGCGCCAATTCAAAATGGTTCAATCTAAGCACTTGGTCTATGATTATTGTAAGGATACTGCAAGATTCACTCAAATGCCTAAAGAATGCTTGGACTAGAGTTCGGCAAGCAACGTGTTGTTCAATGTCTAAATATGTTTATGTCTCGCACAATTTTTGTTTCCCAAATTTGTTGGTCCAATTTGCTGATCTTATTTAAGGGCAATTCTGCAGATATCCATCACACTGGCGGCCGCTCGAGCATGCATCT

## >NtXTH15.3 amplification (4 fragments insertion)

GGGCGATTGGGCCCTCTAGATGCATGCTCGAGCGGCCGCCAGTGTGATGGATATCTGCAGAATTGCCCTTATGTTTGAACAAAAGAATCTGTTATCAAAACTGGTAGTTAGTGGAGTGCAGCAACGAATCAGTTCATCTGGTAAAAAAGAATATAGTAAACAAAAC**ATG**AGAAGAAAAAGCTGCATGCTGACGACAGTGCCATGGCTGCCACTAAAACATTCTCTTGCTCGCT**GTAATCCATGATCCTAATTTAAACAATCCAGTCCATAAATATATGTTAATACTAACTTATATATAAAACTAAAGTGCCTCCAAGTTCTTGATTGGCGTTTTTCCTAATAGACCAATATAGACTTGAACCTTTTACTTCTAGACCCTAAAATTAATTGGTTTCAAGTTCCAGACAGCAATGTTATCCTCTATGATTGTCTTTTTGATCCTAGCTATGTTACTAAACACAG**GGGTTGGTGTCAACTTCACCGAAGTTTTCGAGTCGAGTTGGTCACCTGACCATATTACTGTGGTAGGAGACCAAGTTATGCTCACCCTTGACAATGCTTCTG**GTAGTTACCAATATGTCTCACTTCATTTCTACTTACTTTCTCTTCCTAGTCTTTTATTATATTTTCTGTTTTTCCAGGCAATATAATTAAATAACCATTGATTTATTATGTTGATATTTTGCAAAG**GCTGCGGGTTTCAGTCGAAGAACAAATATATGTTTGGGAAAGCCAGCGCGCAGATCAAACTAGTTGATGGAGATTCAGCTGGAACAGTCATTGCTTTTTAT**GTACGTAACGAAAACCAAACAAATATTCATCAAGAGTACTCTTATATTGTTTCTTTAAACTATTACCAACATTATTGGAATTAATATGTGGTTGCAG**ATGTCATCAGAGGGAGCTAATCACGACGAACTGGACTTTGAGTTTCTAGGGAATGTTTCAGGAGAACCATACCTAGTACAAACAAATGTGTACGCGAATGGCACCGGAGACAGAGAGCAGAGGCATAGTCTTTGGTTCGATCCAACTGCGGATTTCCACACTTACTCTTTCTTTTGGAATCATCATACCATTAT**GTATGTAGTTCTTACTTCGACCATCGCTAAATGTGTTTGATCTTTCCTTTAAATAATTTTAGAGGCTTGCAACTTTGGCAATTTAAAGTAAATTTTTATAACGAGTTGATAACATATATAACGAGTTAAATTCACATCTAATAATGAAGAATTTTACTTGTCATATGAATTCTTGCAG**CTTTTCGGTTGATGACATTCCTATTAGAGTGTTCAAAAACACAGAGAAAAAAGGCGTGGCATACCCGAAAAATCAAGGCATGGGAGTTTATGGATCGTTGTGGAATGCAGATGACTGGGCTACACAAGGAGGGAGAGTGAAGACCAACTGGAGCCACTCTCCATTTGTTGCAACATTTCGAGCGTTCGAGATTGATGCTTGTGATTTGTCTGGTGAGGACACAGTTGCTGCAGGCGCAAAATGTGGCAAGTTAGCACAATGCTGGTGGGATAAGCCAGCCATGAGGGAGCTGAACAAGAGCAAAAAGCGCCAATTCAAAATGGTTCAATCTAAGCACTTGGTCTATGATTATTGTAAGGATACTGCAAGATTCACTCAAATGCCTAAAGAATGCTTGGACTAGAGTTCGGCAAGCAACGTGTTGTTCAATGTCTAAATATGTTTATGTCTCGCACAATTTTTGTTTCCCAAATTTGTTGGTCCAATTTGCTGATCTTATTTAAGGGCAATTCCAGCACACTGGCGGCCGT

# >NtXTH16(SGN database sequence)

ATGGGGATGAATATGTTGTTGGTGTGTGTGTTATTTGTCGTAGGAGCAATGGCTGCTGCGCCAAAGAAGCCAATGGATGTACCATTTGGAAGAAACTATGAGAATACTTGGGCTCCTGATCATGTCAAATACTTTAATGGTGGCAGTGAGATCCAGCTCTTCCTTGACAACCGCACTGGTACTGGATTCCAGTCAAAAGGATCTTACCTATTTGGGCACTTTGCTATGCACATAAAGATGGTTGCTGGTGATTCTGCAGGCACTGTCACTGCTTTCTATCTGTCTTCACAAAATAATGAGCATGATGAAATAGACTTTGAGTTTTTGGG***GAACAAAACAGGAGAACCATATGTGGT***ACAAACAAATATATACACAGGAGGGAAAGGTGACAAAGAGCAGAGGATTTACTTATGGTTTGATCCAACCAAAGATTACCACACCTATTCTGTTTTGTGGAATCTCCACCAGATTGTGTTTTTTGTAGATGAGTACCCAATCAGAACATTCAAAAACAGCAAAGATTTAGGTGTCAAATTCCCATTTGATCAACCAATGAAGATATACTCAAGTCTATGGGAAGCAGATGATTGGGCAACAAGAGGTGGACTTGAAAAAATAGATTGGTCAAATGCACCTTTTGTTGCTTCTTACAAAGGATTTCACATAGATGGATGTGAAGCTTCAGTAAATGCAAAATTATGTGCAAATCAAGGCAAAAAATGGTGGGATCAAAAAGAATTTCAAGATTTGGATAAACAACAATGGAGACTTTTACGTAGAGTAAGGGATAAATACACTATTTATAACTATTGCACTGATAAAAAGAGGTTTGCAACTCTGCCAAAAGAGTGCAGGAGGAATAGAGATGTGCCAAGAAAATCATCAAAGAAGTCTCCTTAG

## >NtXTH16 amplification (same with SGN database sequence)

TTAAATTAGAGTAGAAACATT**ATG**GGGATGAATATGTTGTTGGTGTGTGTGTTATTTGTCGTAGGAGCAATGGCTGCTGCGCCAAAGAAGCCAATGGATGTACCATTTGGAAGAAACTATGAGAATACTTGGGCTCCTGATCATGTCAAATACTTTAATGGTGGCAGTGAGATCCAGCTCTTCCTTGACAACCGCACTGGTACTGGATTCCAGTCAAAAGGATCTTACCTATTTGGGCACTTTGCTATGCACATAAAGATGGTTGCTGGTGATTCTGCAGGCACTGTCACTGCTTTCTATCTGTCTTCACAAAATAATGAGCATGATGAAATAGACTTTGAGTTTTTGGGGAACAAAACAGGAGAACCATATGTGGTACAAACAAATATATACACAGGAGGGAAAGGTGACAAAGAGCAGAGGATTTACTTATGGTTTGATCCAACCAAAGATTACCACACCTATTCTGTTTTGTGGAATCTCCACCAGATTGTGTTTTTTGTAGATGAGTACCCAATCAGAACATTCAAAAACAGCAAAGATTTAGGTGTCAAATTCCCATTTGATCAACCAATGAAGATATACTCAAGTCTATGGGAAGCAGATGATTGGGCAACAAGAGGTGGACTTGAAAAAATAGATTGGTCAAATGCACCTTTTGTTGCTTCTTACAAAGGATTTCACATAGATGGATGTGAAGCTTCAGTAAATGCAAAATTATGTGCAAATCAAGGCAAAAAATGGTGGGATCAAAAAGAATTTCAAGATTTGGATAAACAACAATGGAGACTTTTACGTAGAGTAAGGGATAAATACACTATTTATAACTATTGCACTGATAAAAAGAGGTTTGCAACTCTGCCAAAAGAGTGCAGGAGGAATAGAGATGTGCCAAGAAAATCATCAAAGAAGTCTCCT**TAG**GTTGCGACGGTCGTGCTCGTCTTAAAC

# >NtXTH17 (SGN database sequence)

ATGGGGTTCAAATGGATGAATATGTTGTTGTTTTGTGCGTTATTTGTCGTAGGAGCAATGGCTGCTGCACCAAAGAAGCCAATGGATGTACCATTTGGAAGAAACTATGAGAATAGCTGGGCTCCTGATCATGTCAAATACTTTAATGGTGGCAGTGAGATCCAGCTCTTCCTTGACAACCGCACTGGAACTGGCTTCCAATCAAAAGGATCTTACCTATTTGGGCACTTTGCTATGCACATAAAGATGGTTGCTGGTGATTCTGCAGGCACTGTGACTGCTTTCTATTTGTCTTCACAAAATAATGAGCATGATGAAATAGATTTTGAGTTTTTGGG***GAACAAAACAGGAGAGCCATATGTTGT***ACAGACAAATGTATACACAGGAGGGAAAGGTGACAAAGAGCAGAGGATTTATTTATGGTTTGATCCAACCAAAGATTACCACACCTATTCTGTTTTGTGGAATCTCCACCAGATTGTGTTTTTTGTAGATGAGTACCCAATCAGAACGTTCAAGAACAGCAAAGATTTAGGAGTCAAATTTCCATTTGACCAACCAATGAAGATATACTCAAGTCTATGGGAAGCAGATGATTGGGCAACAAGAGGTGGACTTGAAAAAATAGATTGGTCAAATGCACCTTTTGTTGCATCTTACAAAGGATTTCACATAGATGGATGTGAAGCCTCTGTAAATGCAAAATATTGTTCAAATCAAGGCAAGAAATGGTGGGATCAAAAAGAATTTCAAGATTTGGATAAACAACAATGGAGACTTTTACGTAGAGTAAGGGATAAATACACTATTTATAACTATTGCACTGATAAAAAGAGGTTTGCAACTATGCCAAAAGAGTGCAGGAGGAATAGAGATGTGCCTAGAAAATCATCAAAAAAGTCTCCTTAG

## >NtXTH17 amplification (same with SGN database sequence)

GTGCAGAGCAAATTAGAGTAGAAACATT**ATG**GGGTTCAAATGGATGAATATGTTGTTGTTTTGTGCGTTATTTGTCGTAGGAGCAATGGCTGCTGCACCAAAGAAGCCAATGGATGTACCATTTGGAAGAAACTATGAGAATAGCTGGGCTCCTGATCATGTCAAATACTTTAATGGTGGCAGTGAGATCCAGCTCTTCCTTGACAACCGCACTGGAACTGGCTTCCAATCAAAAGGATCTTACCTATTTGGGCACTTTGCTATGCACATAAAGATGGTTGCTGGTGATTCTGCAGGCACTGTGACTGCTTTCTATTTGTCTTCACAAAATAATGAGCATGATGAAATAGATTTTGAGTTTTTGGGGAACAAAACAGGAGAGCCATATGTTGTACAGACAAATGTATACACAGGAGGGAAAGGTGACAAAGAGCAGAGGATTTATTTATGGTTTGATCCAACCAAAGATTACCACACCTATTCTGTTTTGTGGAATCTCCACCAGATTGTGTTTTTTGTAGATGAGTACCCAATCAGAACGTTCAAGAACAGCAAAGATTTAGGAGTCAAATTTCCATTTGACCAACCAATGAAGATATACTCAAGTCTATGGGAAGCAGATGATTGGGCAACAAGAGGTGGACTTGAAAAAATAGATTGGTCAAATGCACCTTTTGTTGCATCTTACAAAGGATTTCACATAGATGGATGTGAAGCCTCTGTAAATGCAAAATATTGTTCAAATCAAGGCAAGAAATGGTGGGATCAAAAAGAATTTCAAGATTTGGATAAACAACAATGGAGACTTTTACGTAGAGTAAGGGATAAATACACTATTTATAACTATTGCACTGATAAAAAGAGGTTTGCAACTATGCCAAAAGAGTGCAGGAGGAATAGAGATGTGCCTAGAAAATCATCAAAAAAGTCTCCT**TAG**GTTACGACGGTCGTGCTCG

# >NtXTH18 (SGN database sequence)

ATGGGTCTAAAAGGACTTTTGTTTAGTATTGTTTTGATTAATTTGTCATTACTAGGACTTTGTGGGTATCCCAGAAAACCTGTGGATGTACCCTTTTGGAAAAACTATGAGCCCAGTTGGGCTAGTCACCACATCAAGTACCTCAATGGTGGTTCCACTGCTGATCTTGTTCTTGACAGGTCTTCAGGAGCTGGATTTCAGTCAAAGAAATCATATCTATTTGGGCACTTTAGCATGAAACTGAGGCTTGTTGGTGGAGACTCCGCTGGTGTTGTTACTGCATTTTACCTGTCATCGAATAATGCAGAGCAC***GATGAGATAGATTTTGAATTCTTAGGG***AACAGGACTGGGCAACCATACATTTTGCAGACGAATGTGTTTACGGGAGGAAAAGGAGACAGAGAGCAGAGAATCTATCTTTGGTTTGACCCAACCAAGGGTTACCATTCTTATTCCGTTCTTTGGAATACCTTCCAGATTGTGATCTTTGTGGATGACGTCCCAATACGAGCATTCAAGAACTCGAAAGACCTAGGTGTGAAATTCCCATTCAATCAACCCATGAAAATATACTCAAGCCTTTGGGATGCAGATGATTGGGCCACAAGAGGTGGATTGGAGAAAACAGACTGGTCAAATGCCCCATTTACTGCCTCCTACACATCATTCCACGTGGACGGCTGTGAAGCTGCCACCCCACAAGAAGTCCAAGTTTGTAACACCAAAGGCATGAGATGGTGGGATCAAAAGGCTTTCCAAGATTTAGATGCTTTGCAATATAGGAGACTTCGTTGGGTTCGTCAAAAATACACTATCTATAACTATTGCACTGATAGGAAGAGATACCCTACTCTTCCACCAGAATGCACTAAGGACAGAGATATTTAA

## >NtXTH18 amplification (same with SGN database sequence)

ATAGTGTTCTCTGATTGGCTTTGCTTTAGAACATTCACC**ATG**GGTCTAAAAGGACTTTTGTTTAGTATTGTTTTGATTAATTTGTCATTACTAGGACTTTGTGGGTATCCCAGAAAACCTGTGGATGTACCCTTTTGGAAAAACTATGAGCCCAGTTGGGCTAGTCACCACATCAAGTACCTCAATGGTGGTTCCACTGCTGATCTTGTTCTTGACAGGTCTTCAGGAGCTGGATTTCAGTCAAAGAAATCATATCTATTTGGGCACTTTAGCATGAAACTGAGGCTTGTTGGTGGAGACTCCGCTGGTGTTGTTACTGCATTTTACCTGTCATCGAATAATGCAGAGCACGATGAGATAGATTTTGAATTCTTAGGGAACAGGACTGGGCAACCATACATTTTGCAGACGAATGTGTTTACGGGAGGAAAAGGAGACAGAGAGCAGAGAATCTATCTTTGGTTTGACCCAACCAAGGGTTACCATTCTTATTCCGTTCTTTGGAATACCTTCCAGATTGTGATCTTTGTGGATGACGTCCCAATACGAGCATTCAAGAACTCGAAAGACCTAGGTGTGAAATTCCCATTCAATCAACCCATGAAAATATACTCAAGCCTTTGGGATGCAGATGATTGGGCCACAAGAGGTGGATTGGAGAAAACAGACTGGTCAAATGCCCCATTTACTGCCTCCTACACATCATTCCACGTGGACGGCTGTGAAGCTGCCACCCCACAAGAAGTCCAAGTTTGTAACACCAAAGGCATGAGATGGTGGGATCAAAAGGCTTTCCAAGATTTAGATGCTTTGCAATATAGGAGACTTCGTTGGGTTCGTCAAAAATACACTATCTATAACTATTGCACTGATAGGAAGAGATACCCTACTCTTCCACCAGAATGCACTAAGGACAGAGATATT**TAA**GTAATTTAATTAAGAGGGGGCTTTTTAAGCACTTAATATTATTT

# >NtXTH19 (SGN database sequence)

ATGGGTGTAAAAGGACTTTTGTTTAGTATTGTTTTGATTAATTTGTCATTACTAGGACTTTGTGGGTATCCCAGAAAACCAGTGGATGTACCCTTTTGGAAAAACTATGAGCCCAGTTGGGCTAGTCACCACATCAAGTACCTCAGTGGTGGTTCCACTGTTGATCTTGTTCTTGACAGGTCTTCAGGTGCTGGATTTCAGTCAAAGAAATCATATTTGTTTGGGCACTTTAGCATGAAACTGAAGCTTGTTGGTGGAGACTCAGCTGGCGTTGTCACTGCATTTTACCTGTCATCGAATAATGCAGAGCAC***GATGAGATAGATTTTGAATTCTTAGGG***AACAGGACTGGGCAACCATACATTTTGCAGACAAATGTGTTCACGGGAGGAAAAGGAGACAGAGAGCAGAGAATCTATCTCTGGTTTGACCCAACCAAGGGTTACCATTCTTATTCTGTTCTTTGGAATACCTTCCAGATTGTGATCTTTGTGGATGACGTCCCAATTAGAGCATTCAAGAACTCAAAAGACCTAGGTGTGAAATTTCCATTCAATCAGCCCATGAAAATATACTCAAGCCTTTGGGATGCAGATGATTGGGCCACAAGAGGTGGATTGGAGAAAACAGACTGGTCCAATGCCCCATTTACTGCCTCCTACACATCATTCCACGTGGACGGCTGTGAAGCTGCCACCCCACAAGAAGTCCAAGTTTGTAACACCAAAGGCATGAGATGGTGGGATCAAAAGGCTTTCCAAGATTTAGATGCTTTACAATACAGGAGACTTCGTTGGGTTCGCCAAAAATACACTATTTATAATTATTGTACTGATAGGAAGAGGTACCCTACACTTCCCCCAGAGTGCACTAAGGACAGAGATATTTAA

## >NtXTH19 amplification (same with SGN database sequence)

GCAATTTTCTGAAAATTTTCACCATTTACGAACGATAGCCATGGTACCA**ATG**GGTGTAAAAGGACTTTTGTTTAGTATTGTTTTGATTAATTTGTCATTACTAGGACTTTGTGGGTATCCCAGAAAACCAGTGGATGTACCCTTTTGGAAAAACTATGAGCCCAGTTGGGCTAGTCACCACATCAAGTACCTCAGTGGTGGTTCCACTGTTGATCTTGTTCTTGACAGGTCTTCAGGTGCTGGATTTCAGTCAAAGAAATCATATTTGTTTGGGCACTTTAGCATGAAACTGAAGCTTGTTGGTGGAGACTCAGCTGGCGTTGTCACTGCATTTTACCTGTCATCGAATAATGCAGAGCACGATGAGATAGATTTTGAATTCTTAGGGAACAGGACTGGGCAACCATACATTTTGCAGACAAATGTGTTCACGGGAGGAAAAGGAGACAGAGAGCAGAGAATCTATCTCTGGTTTGACCCAACCAAGGGTTACCATTCTTATTCTGTTCTTTGGAATACCTTCCAGATTGTGATCTTTGTGGATGACGTCCCAATTAGAGCATTCAAGAACTCAAAAGACCTAGGTGTGAAATTTCCATTCAATCAGCCCATGAAAATATACTCAAGCCTTTGGGATGCAGATGATTGGGCCACAAGAGGTGGATTGGAGAAAACAGACTGGTCCAATGCCCCATTTACTGCCTCCTACACATCATTCCACGTGGACGGCTGTGAAGCTGCCACCCCACAAGAAGTCCAAGTTTGTAACACCAAAGGCATGAGATGGTGGGATCAAAAGGCTTTCCAAGATTTAGATGCTTTACAATACAGGAGACTTCGTTGGGTTCGCCAAAAATACACTATTTATAATTATTGTACTGATAGGAAGAGGTACCCTACACTTCCCCCAGAGTGCACTAAGGACAGAGATATTGGACGGGCGGCCGCAGGCATGGTGAGCAAGGGCGAGGAGCTGTTCACCGGGGTGGTGCCCATCCTGGT

# >NtXTH20 (SGN database sequence)

ATGCAACTCAAACTTGTCCCTGGAAATTCTGCTGGCACTGTCACCACCTTCTTCTTATCTTCACAAGGAGCTGGACAT***GATGAGATTGATTTCGAGTTCTTAGGC***AATGTTTCTGGCCAACCTTACACAGTTCATACCAATGTTTACTCGCAAGGCAAAGGCAACAAAGAACAACAATTCCATTTGTGGTTCGACCCAACTGCTGCATTTCACACTTACTCCATTATCTGGAATGCTCAGAAGATCATTTTCTTGGTAGATAATAGTCCAATCAGAGTATACAACAACCACGAAAGCGCTGGCATTCCATTCCCAAAAAGCCAACCAATGAAAGTGTACTGCAGCTTATGGAATGCAGATGAGTGGGCTACACAAGGAGGTAGAGTCAAGACAGATTGGACACATGCTCCTTTCACTGCATATTACAGAAATTTCAATATTGATGGCTGCGCAGTCACATCCGGCGCCTCTTCGTGTAAGTCCACTGATTCAGCAAACAATGCTAGGCCATGGCAAAATCAAGAACTTGATGCTAAGGGCAGGAATAGGCTACGATGGGTGCAGAGCAGACACATGGTTTACAACTATTGTGCTGATTCTAAGAGGTTTCCTCAAGGCTTTTCTCATGAGTGCAAGCGTTCGAGGTTCCTCTAA

## >NtXTH20.1 amplification (same with SGN database sequence)

AATAACAGGAGGAGGCAGAGGCCTTTCTCTGTCCCTTGATAAATTTTCTGGTTCGGGTTTTCAATCGAAGAATGAGTATCTCTTTGGAAGATTTGAC**ATG**CAACTCAAACTTGTCCCTGGAAATTCTGCTGGCACTGTCACCACCTTCTTCTTATCTTCACAAGGAGCTGGACATGATGAGATTGATTTCGAGTTCTTAGGCAATGTTTCTGGCCAACCTTACACAGTTCATACCAATGTTTACTCGCAAGGCAAAGGCAACAAAGAACAACAATTCCATTTGTGGTTCGACCCAACTGCTGCATTTCACACTTACTCCATTATCTGGAATGCTCAGAAGATCATTTTCTTGGTAGATAATAGTCCAATCAGAGTATACAACAACCACGAAAGCGCTGGCATTCCATTCCCAAAAAGCCAACCAATGAAAGTGTACTGCAGCTTATGGAATGCAGATGAGTGGGCTACACAAGGAGGTAGAGTCAAGACAGATTGGACACATGCTCCTTTCACTGCATATTACAGAAATTTCAATATTGATGGCTGCGCAGTCACATCCGGCGCCTCTTCGTGTAAGTCCACTGATTCAGCAAACAATGCTAGGCCATGGCAAAATCAAGAACTTGATGCTAAGGGCAGGAATAGGCTACGATGGGTGCAGAGCAGACACATGGTTTACAACTATTGTGCTGATTCTAAGAGGTTTCCTCAAGGCTTTTCTCATGAGTGCAAGCGTTCGAGGTTCCTCTAATTAAGTTGCTGGAGCAAGGAAATTGTGGCACTGCCTCTGCT

## >NtXTH20.2 amplification (1 fragment insertion)

GAGCTCGGATCcACTAGTAACGGCCGCCAGTGTGCTGGAATTGCCCTTAAGGACGTGGTAAAATAACAGGAGGAGGCAGAGGCCTTTCTCTGTCCCTTGATAAATTTTCTGGTTCGGGTTTTCAATCGAAGAATGAGTATCTCTTTGGAAGATTTGAC**ATG**CAACTCAAACTTGTCCCTGGAAATTCTGCTGGCACTGTCACCACCTTCTTC**GTAAGTCATTCAACTTTTCCCTTACAAAATTGTATTAGTTCTATTTCATGTCCAAGTTCTGATGATTTAATATTTTTTCTTGTTCTTTGTTTCTTTCATCAGTTATCTTCACAAGGAGCTGGACATGATGAGATTGATTTCGAGTTCTTAGGCAATGTTTCTGGCCAACCTTACACAG**TTCATACCAATGTTTACTCGCAAGGCAAAGGCAACAAAGAACAACAATTCCATTTGTGGTTCGACCCAACTGCTGCATTTCACACTTACTCCATTATCTGGAATGCTCAGAAGATCAT**GTAAGTTCCTTATATTAATCGCACTGTAAGAATTAATGATGTTTGCTTGCAATTTCTTCTTATTTTGGTACTGATGATTTCCATATGAATTCCCATCTTCAG**TTTCTTGGTAGATAATAGTCCAATCAGAGTATACAACAACCACGAAAGCGCTGGCATTCCATTCCCAAAAAGCCAACCAATGAAAGTGTACTGCAGCTTATGGAATGCAGATGAGTGGGCTACACAAGGAGGTAGAGTCAAGACAGATTGGACACATGCTCCTTTCACTGCATATTACAGAAATTTCAATATTGATGGCTGCGCAGTCACATCCGGCGCCTCTTCGTGTAAGTCCACTGATTCAGCAAACAATGCTAGGCCATGGCAAAATCAAGAACTTGATGCTAAGGGCAGGAATAGGCTACGATGGGTGCAGAGCAGACACATGGTTTACAACTATTGTGCTGATTCTAAGAGGTTTCCTCAAGGCTTTTCTCATGAGTGCAAGCGTTCGAGGTTCCTCTAATTAAGTTGCTGGAGCAAGGAAATTGTGGCACTGCCTCTGCTATTTATTAAGATTAAGGGCAATTCTGCAGATATCCATCACACTGGCGGCCGCTCGAGCATGCATCTAGAGGGCCCAATCGCCCTA

# >NtXTH21 (SGN database sequence)

ATGTCGCCTCGTTTCTCTTTCAAAATGTTAATCCTTCCTATAGTCATGGCAAGTCTATGGGCAGCCGCCTCAGCTGGTAATTTTTATAATCTTGCAGATATCACTTGGGGCGAAGGACGTGGTAAAATAACAGAAGGAGGCAGAGGCCTCTCTCTGTCCCTTGACAAATTATCTGGTTCAGGTTTTCAATCCAAGAATGAGTATTTATTCGGAAGATTTGACATGCAACTCAAACTTGTCCCTGGAAATTCTGCTGGCACTGTCACCACCTTCTTTTTATCTTCACAAGGAGCAGGACAT***GATGAGATTGACTTCGAGTTCTTAGGC***AATGTTTCTGGTCAACCTTACACAGTCCACACCAATGTTTACTCGCAAGGCAAAGGCAACAAAGAACAACAATTCCATTTGTGGTTCGACCCAACTGCTGCATTTCACACTTACTCCATCATCTGGAACGCTCAGAAAATCATTTTCTTGGTGGATAATAGTCCAATCAGAGTATACAACAACCACGAAAGCAATGGCATTCCATTCCCAAAAATCCAACCAATGAAAGTGTACTGCAGCTTATGGAATGCAGATGAGTGGGCAACACAAGGAGGTAGAGTCAAGACAGATTGGACACATGTTCCTTTCACTGCTTACTACAGAAACTTCAATATTGATGGCTGCGCAGTTACATCCGGCACCTCTTCGTGTAAGTCCACTGATTCAGCCAACAATGCTAGGCCATGGCAAAATCAAGAACTTGATGCTAAGGGCAGGAATAGGCTACGATGGGTTCAAAGCAGACACATGGTTTACAACTATTGTGCTGATTCTAAGAGGTTTCCTCAAGGCTTTTCTCATGAGTGCAAGCGTTCGAGGTTCCTGTAA

## >NtXTH21 amplification (same with SGN database sequence)

CAACTTGAAAAAAACGTTGAGAAATCATTTTGAAAA**ATG**TCGCCTCGTTTCTCTTTCAAAATGTTAATCCTTCCTATAGTCATGGCAAGTCTATGGGCAGCCGCCTCAGCTGGTAATTTTTATAATCTTGCAGATATCACTTGGGGCGAAGGACGTGGTAAAATAACAGAAGGAGGCAGAGGCCTCTCTCTGTCCCTTGACAAATTATCTGGTTCAGGTTTTCAATCCAAGAATGAGTATTTATTCGGAAGATTTGACATGCAACTCAAACTTGTCCCTGGAAATTCTGCTGGCACTGTCACCACCTTCTTTTTATCTTCACAAGGAGCAGGACATGATGAGATTGACTTCGAGTTCTTAGGCAATGTTTCTGGTCAACCTTACACAGTCCACACCAATGTTTACTCGCAAGGCAAAGGCAACAAAGAACAACAATTCCATTTGTGGTTCGACCCAACTGCTGCATTTCACACTTACTCCATCATCTGGAACGCTCAGAAAATCATTTTCTTGGTGGATAATAGTCCAATCAGAGTATACAACAACCACGAAAGCAATGGCATTCCATTCCCAAAAATCCAACCAATGAAAGTGTACTGCAGCTTATGGAATGCAGATGAGTGGGCAACACAAGGAGGTAGAGTCAAGACAGATTGGACACATGTTCCTTTCACTGCTTACTACAGAAACTTCAATATTGATGGCTGCGCAGTTACATCCGGCACCTCTTCGTGTAAGTCCACTGATTCAGCCAACAATGCTAGGCCATGGCAAAATCAAGAACTTGATGCTAAGGGCAGGAATAGGCTACGATGGGTTCAAAGCAGACACATGGTTTACAACTATTGTGCTGATTCTAAGAGGTTTCCTCAAGGCTTTTCTCATGAGTGCAAGCGTTCGAGGTTCCTG**TAA**TTAAGTtGCTGgAACAAGGAAATTGTGGCACTGCCCCGCATTTATTTT

# >NtXTH22 (SGN database sequence)

ATGGCTTCTCATTTGTTTCTAATTTCCATTCTAATGGGCAGCCTAGTTGCTGCCTCAGCTAATTTTAATAATCTTGCAGAGATCACTTGGGGCGAAGGACGTGGTAAAATAACAGAAGGAGGCAAGGGCCTCTCCCTGTCCCTTGACAAACTTTCTGGTTCAGGTTTTCAATCCAAGAATGAATATCTCTTTGGAAGATTTGACATGCAACTCAAACTCGTTCCTGGAAACTCTGCTGGCACTGTCACCACCTTCTTTTTATCTTCACAAGGAGAAGGACAT***GATGAGATCGATTTCGAGTTCTTGGGT***AATACGACGGGCGAGCCCTACACTGTCCATACCAACGTCTATTCTCAAGGAAAGGGAAACAAAGAACAACAATTCCACCTTTGGTTCGATCCAACTGCAGCATTTCACACTTACACCATTGTGTGGAATTCTAACCGCATAGTGTTCTTGGTGGATAACATTCCAATTAGAGTATACAACAACCATGAAAACAATGGCATTCCATTCCCAAAGAGCCAACCAATGAAAGTGTACTGCAGCTTATGGAATGCAGATGAGTGGGCTACACAAGGAGGCAGAGTCAAGACTGATTGGACACATGCTCCTTTCACAGCTTACTACAGAAACTTCAAAATAGATGGCTGCGCAGTCACATCCGGCGCCTCTTCATGTAAGTCCACTGATTCTGCAGGCAATGCTAAGGCATGGCAAAATCAAGAACTTGATGCTAAGGGCAGGAATAGAGTCCGATGGGTGCAAAGTAGACACATGGTTTACAACTACTGCGCTGATAAAAAGAGGTTTCCTCAAGGCTATTCTCATGAATGCAAGAGCTCAAGGTTTTAA

## >NtXTH22 amplification (same with SGN database sequence)

TCCTTTAGCAACCTAAAGAATCACCATTCTTACTACTCTTATCATCTCTTCAGCATTTCA**ATG**GCTTCTCATTTGTTTCTAATTTCCATTCTAATGGGCAGCCTAGTTGCTGCCTCAGCTAATTTTAATAATCTTGCAGAGATCACTTGGGGCGAAGGACGTGGTAAAATAACAGAAGGAGGCAAGGGCCTCTCCCTGTCCCTTGACAAACTTTCTGGTTCAGGTTTTCAATCCAAGAATGAATATCTCTTTGGAAGATTTGACATGCAACTCAAACTCGTTCCTGGAAACTCTGCTGGCACTGTCACCACCTTCTTTTTATCTTCACAAGGAGAAGGACATGATGAGATCGATTTCGAGTTCTTGGGTAATACGACGGGCGAGCCCTACACTGTCCATACCAACGTCTATTCTCAAGGAAAGGGAAACAAAGAACAACAATTCCACCTTTGGTTCGATCCAACTGCAGCATTTCACACTTACACCATTGTGTGGAATTCTAACCGCATAGTGTTCTTGGTGGATAACATTCCAATTAGAGTATACAACAACCATGAAAACAATGGCATTCCATTCCCAAAGAGCCAACCAATGAAAGTGTACTGCAGCTTATGGAATGCAGATGAGTGGGCTACACAAGGAGGCAGAGTCAAGACTGATTGGACACATGCTCCTTTCACAGCTTACTACAGAAACTTCAAAATAGATGGCTGCGCAGTCACATCCGGCGCCTCTTCATGTAAGTCCACTGATTCTGCAGGCAATGCTAAGGCATGGCAAAATCAAGAACTTGATGCTAAGGGCAGGAATAGAGTCCGATGGGTGCAAAGTAGACACATGGTTTACAACTACTGCGCTGATAAAAAGAGGTTTCCTCAAGGCTATTCTCATGAATGCAAGAGCTCAAGGTTT**TAA**TTAGGAGATGACACTTCATTATATTAATTCCAAAGGTTTGAAGATTGATGCATAGCCTGTGAA

# >NtXTH23 (SGN database sequence)

ATGGCTTCTCATTTTCTTCTGATTTCCATTCTAATGGGCAGCCTAGTCGTTGCATCAGCTAATTTTAATAATCTTGCAGAGATTACTTGGGGCGAAGGACGTGGTAAAATAACAGAAGGAGGCAAAGGTCTCTCTCTGTCCCTTGACAAACTTTCTGGCTCAGGTTTTCAATCCAAGAATGAGTATTTATTCGGGAGATTTGACATGCAACTCAAACTTGTACCTGGAAACTCTGCTGGCACTGTCACCACCTTCTTTTTATCTTCACAAGGAAAAGGACAT***GATGAGATTGATTTCGAGTTCTTGGGT***AATACGACTGGCGAGCCCTACACTGTCCACACCAACGTGTATTCTCAAGGAAAGGGAAACAAAGAACAACAATTCCACCTTTGGTTCGACCCAACTGCAGCATTTCACACCTACACCATTGTGTGGAACGCTAACCGCATACTGTTCTTGGTAGATAACATCCCAATTAGAGTGTACAACAACCATGAAAGCAATGGCATTCCATTCCCAAAGAGCCAACCAATGAAAGTGTACTGCAGCTTATGGAATGCAGATGAGTGGGCTACACAAGGAGGCAGAGTCAAGACTGACTGGACACATGCTCCTTTCACTGCTTACTACAGAAACTTCAAAATTGATGGTTGCGCAGTCACATCGGGGGCCTCTTCATGTAAGTCCACTGATTCTGCAGGCAATGCTAAGGCATGGCAAAATCATGAACTTGATGCTAAGGGCAGGAATAGGGTCCGATGGGTGCAGAGCAGACACATGGTTTACAACTACTGTGCTGATAAAAAGAGGTTTCCTCAAGGCTATTCTCATGAGTGCAAGAGCTCAAGGTTTTAA

## >NtXTH23.1 amplification (same with SGN database sequence)

TTACTCTTATCTTCTCTTCAGCATTTCA**ATG**GCTTCTCATTTTCTTCTGATTTCCATTCTAATGGGCAGCCTAGTCGTTGCATCAGCTAATTTTAATAATCTTGCAGAGATTACTTGGGGCGAAGGACGTGGTAAAATAACAGAAGGAGGCAAAGGTCTCTCTCTGTCCCTTGACAAACTTTCTGGCTCAGGTTTTCAATCCAAGAATGAGTATTTATTCGGGAGATTTGACATGCAACTCAAACTTGTACCTGGAAACTCTGCTGGCACTGTCACCACCTTCTTTTTATCTTCACAAGGAAAAGGACATGATGAGATTGATTTCGAGTTCTTGGGTAATACGACTGGCGAGCCCTACACTGTCCACACCAACGTGTATTCTCAAGGAAAGGGAAACAAAGAACAACAATTCCACCTTTGGTTCGACCCAACTGCAGCATTTCACACCTACACCATTGTGTGGAACGCTAACCGCATACTGTTCTTGGTAGATAACATCCCAATTAGAGTGTACAACAACCATGAAAGCAATGGCATTCCATTCCCAAAGAGCCAACCAATGAAAGTGTACTGCAGCTTATGGAATGCAGATGAGTGGGCTACACAAGGAGGCAGAGTCAAGACTGACTGGACACATGCTCCTTTCACTGCTTACTACAGAAACTTCAAAATTGATGGTTGCGCAGTCACATCGGGGGCCTCTTCATGTAAGTCCACTGATTCTGCAGGCAATGCTAAGGCATGGCAAAATCATGAACTTGATGCTAAGGGCAGGAATAGGGTCCGATGGGTGCAGAGCAGACACATGGTTTACAACTACTGTGCTGATAAAAAGAGGTTTCCTCAAGGCTATTCTCATGAGTGCAAGAGCTCAAGGTTT**TAA**TTAGGAAGTGGCACTTCATtATATCAACTCCAAAGGTTTGAAATTGATGCAACCCCGGGTGGTTAAAAT

## >NtXTH23.2 amplification (2 fragments insertion)

ACTCTTATCTTCTCTTCAGCATTTCA**ATG**GCTTCTCATTTTCTTCTGATTTCCATTCTAATGGGCAGCCTAGTCGTTGCATCAGCTAATTTTAATAATCTTGCAGAGATTACTTGGGGCGAAGGACGTGGTAAAATAACAGAAGGAGGCAAAGGTCTCTCTCTGTCCCTTGACAAACTTTCTGGCTCAGGTTTTCAATCCAAGAATGAGTATTTATTCGGGAGATTTGACATGCAACTCAAACTTGTACCTGGAAACTCTGCTGGCACTGTCACCACCTTCTTT**GTAAGCTATTCAATTTTCGATTTTTATAAATATAAGTCTATTTTTATTTCCATGTTACGGTGATTTGATTTTTATTTTATGTTCGTTATTTCTTGATCAG**TTATCTTCACAAGGAAAAGGACATGATGAGATTGATTTCGAGTTCTTGGG**TAA**TACGACTGGCGAGCCCTACACTGTCCACACCAACGTGTATTCTCAAGGAAAGGGAAACAAAGAACAACAATTCCACCTTTGGTTCGACCCAACTGCAGCATTTCACACCTACACCATTGTGTGGAACGCTAACCGCATACTGT**AAGCTATTTTATAATTATGTTATATACTGACAGTATGTTATCTGATGGTTTAAGTATTTTTAGTTAAATTCTATTTTTGTTACTCCACAACGTACATTTTTTTATTTTGCTTACATTGTTTTCAATATGATGGATCAGGT**TCTTGGTAGATAACATCCCAATTAGAGTGTACAACAACCATGAAAGCAATGGCATTCCATTCCCAAAGAGCCAACCAATGAAAGTGTACTGCAGCTTATGGAATGCAGATGAGTGGGCTACACAAGGAGGCAGAGTCAAGACTGACTGGACACATGCTCCTTTCACTGCTTACTACAGAAACTTCAAAATTGATGGTTGCGCAGTCACATCGGGGGCCTCTTCATGTAAGTCCACTGATTCTGCAGGCAATGCTAAGGCATGGCAAAATCATGAACTTGATGCTAAGGGCAGGAATAGGGTCCGATGGGTGCAGAGCAGACACATGGTTTACAACTACTGTGCTGATAAAAAGAGGTTTCCTCAAGGCTATTCTCATGAGTGCAAGAGCTCAAGGTTTTAATTAGGAAGTGGCACTTCATTATATCAA

# >NtXTH24 (SGN database sequence)

ATGGCTTCTAAATTTTCATCAGTAATGCTTCTGCTTTGCATAATAATGAGCATACAATTATTAGCAGCCTCAGCTGGTAACTTCTACAGAGATGCTGTAATTACTTGGGGTGAAGGACGTGGCAAAATACAAGAAGGTGGCAGAGGTCTTGCCCTCACTCTTGACAAATTATCAGGCTCTGGTTTTCAGTCCAAGAATGAATATTTATTTGGAAGATTTGACATGCAACTCAAGCTTGTACCTGGAAATTCCGCTGGCACTGTCACCACTTTCTTTTTATCTTCACAAGGAGAAGGACAT***GATGAGATTGACTTTGAGTTCTTGGGC***AATGTTTCTGGACAGCCTTACACTGTCCATACCAATGTTTATACACAAGGAAAAGGAAACAAAGAACAACAATTCCACCTTTGGTTCGACCCAACTGCCGCATTTCACACTTACACCATTGTCTGGAACCCTCACCGCATAGTGTTTTTAGTGGACAACAGCCCCATTAGAGTATACAACAACCATGAAAGCATAGGCATTCCATTCCCAAAGAGCCAAGCAATGAGAGTATACTGCAGCTTATGGAATGCAGATGAGTGGGCAACACAAGGAGGCAGAGTCAAAACAGATTGGACACTTGCTCCTTTCACTGCTTACTACAGAAACATCAATATCGATGGTTGTGCAGTGTTATCCGGTACCTCGTCATGTAAATCCAGCAATTCAGCAAACAATGCTAAGCCATGGCAAACTCATGAACTTGATGGAAAGGGAAGGAATAGACTAAGATGGGTGCAAAGCAGACACATGGTTTATAATTATTGTGCTGATTCTAAGAGGTTTCCTCAAGGTTTTTCAGCTGAGTGCAAGAGTTCAAGATTTTAG

## >NtXTH24.1 amplification (same with SGN database sequence)

AGATGCATGCTCGAGCGGCCGCCAGTGTGATGGATATCTGCAGAATTGCCCTTTACTATGTTTCAGCTTCGTAATTCCACCTATAAATAGCCTCCTTTAAAAAATCACAATATCATCATCTCACACTCTTCCCTTTTCTCTTGAACAATATTTCAGTTCATAAA**ATG**GCTTCTAAATTTTCATCAGTAATGCTTCTGCTTTGCATAATAATGAGCATACAATTATTAGCAGCCTCAGCTGGTAACTTCTACAGAGATGCTGTAATTACTTGGGGTGAAGGACGTGGCAAAATACAAGAAGGTGGCAGAGGTCTTGCCCTCACTCTTGACAAATTATCAGGCTCTGGTTTTCAGTCCAAGAATGAATATTTATTTGGAAGATTTGACATGCAACTCAAGCTTGTACCTGGAAATTCCGCTGGCACTGTCACCACTTTCTTTTTATCTTCACAAGGAGAAGGACATGATGAGATTGACTTTGAGTTCTTGGGCAATGTTTCTGGACAGCCTTACACTGTCCATACCAATGTTTATACACAAGGAAAAGGAAACAAAGAACAACAATTCCACCTTTGGTTCGACCCAACTGCCGCATTTCACACTTACACCATTGTCTGGAACCCTCACCGCATAGTGTTTTTAGTGGACAACAGCCCCATTAGAGTATACAACAACCATGAAAGCATAGGCATTCCATTCCCAAAGAGCCAAGCAATGAGAGTATACTGCAGCTTATGGAATGCAGATGAGTGGGCAACACAAGGAGGCAGAGTCAAAACAGATTGGACACTTGCTCCTTTCACTGCTTACTACAGAAACATCAATATCGATGGTTGTGCAGTGTTATCCGGTACCTCGTCATGTAAATCCAGCAATTCAGCAAACAATGCTAAGCCATGGCAAACTCATGAACTTGATGGAAAGGGAAGGAATAGACTAAGATGGGTGCAAAGCAGACACATGGTTTATAATTATTGTGCTGATTCTAAGAGGTTTCCTCAAGGTTTTTCAGCTGAGTGCAAGAGTTCAAGATTT**TAG**GATATGAGAGTGTGAAATTCTAAGGGCAATTCCAGCACACTGGCGGCCGTTACTAGTgGATCCGAG

## >NtXTH24.2 amplification (2 fragments insertion)

ATGCATGCTCGAGCGGCCGCCAGTGTGATGGATATCTGCAGAATTGCCCTTTACTATGTTTCAGCTTCGTAATTCCACCTATAAATAGCCTCCTTTAAAAAATCACAATATCATCATCTCACACTCTTCCCTTTTCTCTTGAACAATATTTCAGTTCATAAA**ATG**GCTTCTAAATTTTCATCAGTAATGCTTCTGCTTTGCATAATAATGAGCATACAATTATTAGCAGCCTCAGCTGGTAACTTCTACAGAGATGCTGTAATTACTTGGGGTGAAGGACGTGGCAAAATACAAGAAGGTGGCAGAGGTCTTGCCCTCACTCTTGACAAATTATCAGGCTCTGGTTTTCAGTCCAAGAATGAATATTTATTTGGAAGATTTGACATGCAACTCAAGCTTGTACCTGGAAATTCCGCTGGCACTGTCACCACTTTCTTT**GTAAGTTGCTCTGCTTAAATTGAACACTAGTATCCTATAATTCTATTTCGCCATATATGTGCTCATGGATTGTTCTTTATTTTCTTTGTTTTTCATCAG**TTATCTTCACAAGGAGAAGGACATGATGAGATTGACTTTGAGTTCTTGGGCAATGTTTCTGGACAGCCTTACACTGTCCATACCAATGTTTATACACAAGGAAAAGGAAACAAAGAACAACAATTCCACCTTTGGTTCGACCCAACTGCCGCATTTCACACTTACACCATTGTCTGGAACCCTCACCGCATAGTGT**AAGTTAGACAATCACCTTACATATATCATTCTTATATATCCGGAGTTTAACTTCTAAACACTGAAAGGGTAAAAGAAATTTTTACACTATCAAGTCATCTAACAGATATCTATAATAAGTCAAACTAGTTACCTGAAAAATAAAGCATATTCTATGGCTTTCTATAACATATTAAATTTTACACTGATAGTGTAACAAGAAAAACTTACACTGTTAATGTATATAAGTTTAGATCTAAGTTCTTGTATTTCTGTT**AGTTTTACATTTTATCTTACCTATGCATTCTTTGATATAATCAGGTTTTTAGTGGACAACAGCCCCATTAGAGTATACAACAACCATGAAAGCATAGGCATTCCATTCCCAAAGAGCCAAGCAATGAGAGTATACTGCAGCTTATGGAATGCAGATGAGTGGGCAACACAAGGAGGCAGAGTCAAAACAGATTGGACACTTGCTCCTTTCACTGCTTACTACAGAAACATCAATATCGATGGTTGTGCAGTGTTATCCGGTACCTCGTCATGTAAATCCAGCAATTCAGCAAACAATGCTAAGCCATGGCAAACTCATGAACTTGATGGAAAGGGAAGGAATAGACTAAGATGGGTGCAAAGCAGACACATGGTTTATAATTATTGTGCTGATTCTAAGAGGTTTCCTCAAGGTTTTTCAGCTGAGTGCAAGAGTTCAAGATTT**TAG**GATATGAGAGTGTGAAATTCTAAGGGCAATTCCAGCACACTGGCGGCCGTTACTAGTGGATCCGAG

# >NtXTH25 (SGN database sequence)

ATGGCTTCTAAATTTTCATCAGCAATGCTTCTGCTTTGTATACTAATGAGCATCCAATTATTAGCAGCCTCAGCTGGTAACTTCTACAGAGATACTGTGATTACTTGGGGCGAAGGACGTGGTAAAATACAAGAAGGTGGCAGAGGTCTCGCTCTCACTCTTGATAAACTTTCAGGCTCTGGTTTCCAGTCCAAGAATGAATACTTATTCGGAAGATTTGATATGCAACTCAAGCTTGTGCCTGGAAACTCTGCTGGCACTGTCACCACTTTCTTTTTATCTTCGCAAGGAGAAGGACAT***GATGAGATTGATTTTGAGTTCTTGGGT***AATGTTTCTGGCCAGCCTTACACTGTCCATACCAATGTTTATACACAAGGAAAAGGAAACAAAGAACAACAATTCCACCTTTGGTTCGATCCTACTGCTGCATTTCACACTTACACCATTGTCTGGAACCCTCACCGCATAGTGTTCTTAGTGGATAACAGCCCCATTAGAGTATACAACAACCATGAAAACATTGGCATTCCATTCCCAAAGAGCCAAGCAATGAGAGTATACTGCAGCTTATGGAATGCAGATGAGTGGGCTACACAAGGAGGCAGAGTCAAGACAGATTGGACACTTGCTCCTTTCACTGCCTATTACCGAAACATCAATATTGATGGTTGTGCAGTGTTATCCGGTACCTCGTCGTGTAAGTCTAGCAATTCAGCAAACAATGCTAAGCCATGGCAAACTCATGAACTTGATGGTAAGGGAAGGAATAGGCTAAGATGGGTACAAAGCAGACACATGGTTTATAACTATTGTGCTGATTCTAAGAGGTTTCCTCAAGGTTTTTCTGAAGAGTGCAAGCGTTCAAGGTTTTAG

## >NtXTH25.1 amplification (same with SGN database sequence)

GCATGCTCGAGCGGCCGCCAGTGTGATGGATATCTGCAGAATTGCCCTTTCATAATTCCACCTATAAATAGCCCTCCTTTAAAAACCAGTACATCATCATCTCACATTCTTCCCTTTTCTCTTGAACAATATTAATATTTCAGTTCAGAAA**ATG**GCTTCTAAATTTTCATCAGCAATGCTTCTGCTTTGTATACTAATGAGCATCCAATTATTAGCAGCCTCAGCTGGTAACTTCTACAGAGATACTGTGATTACTTGGGGCGAAGGACGTGGTAAAATACAAGAAGGTGGCAGAGGTCTCGCTCTCACTCTTGATAAACTTTCAGGCTCTGGTTTCCAGTCCAAGAATGAATACTTATTCGGAAGATTTGATATGCAACTCAAGCTTGTGCCTGGAAACTCTGCTGGCACTGTCACCACTTTCTTTTTATCTTCGCAAGGAGAAGGACATGATGAGATTGATTTTGAGTTCTTGGGTAATGTTTCTGGCCAGCCTTACACTGTCCATACCAATGTTTATACACAAGGAAAAGGAAACAAAGAACAACAATTCCACCTTTGGTTCGATCCTACTGCTGCATTTCACACTTACACCATTGTCTGGAACCCTCACCGCATAGTGTTCTTAGTGGATAACAGCCCCATTAGAGTATACAACAACCATGAAAACATTGGCATTCCATTCCCAAAGAGCCAAGCAATGAGAGTATACTGCAGCTTATGGAATGCAGATGAGTGGGCTACACAAGGAGGCAGAGTCAAGACAGATTGGACACTTGCTCCTTTCACTGCCTATTACCGAAACATCAATATTGATGGTTGTGCAGTGTTATCCGGTACCTCGTCGTGTAAGTCTAGCAATTCAGCAAACAATGCTAAGCCATGGCAAACTCATGAACTTGATGGTAAGGGAAGGAATAGGCTAAGATGGGTACAAAGCAGACACATGGTTTATAACTATTGTGCTGATTCTAAGAGGTTTCCTCAAGGTTTTTCTGAAGAGTGCAAGCGTTCAAGGTTT**TAG**ATGATGGGTTCACTTGATCTACTTCCTAATGTTCAATGCATATTCCATTGGTTTTTGGGATTGGTTGTAAGGGCAATTCCAGCACACTGGCGGCCGTTACTAGTGGATCCGAGCTC

## >NtXTH25.2 amplification (2 fragments insertion)

GAGCTCGGATCcACTAGTAACGGCCGCCAGTGTGCTGGAATTGCCCTTCATAATTCCACCTATAAATAGCCCTCCTTTAAAAACCAGTACATCATCATCTCACATTCTTCCCTTTTCTCTTGAACAATATTAATATTTCAGTTCAGAAA**ATG**GCTTCTAAATTTTCATCAGCAATGCTTCTGCTTTGTATACTAATGAGCATCCAATTATTAGCAGCCTCAGCTGGTAACTTCTACAGAGATACTGTGATTACTTGGGGCGAAGGACGTGGTAAAATACAAGAAGGTGGCAGAGGTCTCGCTCTCACTCTTGATAAACTTTCAGGCTCTGGTTTCCAGTCCAAGAATGAATACTTATTCGGAAGATTTGATATGCAACTCAAGCTTGTGCCTGGAAACTCTGCTGGCACTGTCACCACTTTCTTT**GTAAGTTGCTCTGCTTAAATTGAAACCTACTATCTTAATTCTTGTAATATGTCATATATGCTAATGGATTGTTATTGTTCTGTTCTTTTCTTTGTTCATCAATAG**TTATCTTCGCAAGGAGAAGGACATGATGAGATTGATTTTGAGTTCTTGGGTAATGTTTCTGGCCAGCCTTACACTGTCCATACCAATGTTTATACACAAGGAAAAGGAAACAAAGAACAACAATTCCACCTTTGGTTCGATCCTACTGCTGCATTTCACACTTACACCATTGTCTGGAACCCTCACCGCATAGT**GTAAGTTAGACAATCACCTTACATATATCTTTCTTATATATCCAGAGTTTAACTTCTAAACACAGAAAGTATAAAAGATGTTTTTACACTATCAAGTCGTCTAGCACTGACTGTCTGTAATAAGTCAAACTAGTTACCTGAAAAATAAAGCATATCTACGACTTTCTATAATAGATTAAATTACACTGTTATACTGTTAATGTATTTAAGTTTAAATCTAAATTCTTATATTTCTGTTAGTTATACATTTTATCTTACCAATTCTTTGATATAATCAG**GTTCTTAGTGGATAACAGCCCCATTAGAGTATACAACAACCATGAAAACATTGGCATTCCATTCCCAAAGAGCCAAGCAATGAGAGTATACTGCAGCTTATGGAATGCAGATGAGTGGGCTACACAAGGAGGCAGAGTCAAGACAGATTGGACACTTGCTCCTTTCACTGCCTATTACCGAAACATCAATATTGATGGTTGTGCAGTGTTATCCGGTACCTCGTCGTGTAAGTCTAGCAATTCAGCAAACAATGCTAAGCCATGGCAAACTCATGAACTTGATGGTAAGGGAAGGAATAGGCTAAGATGGGTACAAAGCAGACACATGGTTTATAACTATTGTGCTGATTCTAAGAGGTTTCCTCAAGGTTTTTCTGAAGAGTGCAAGCGTTCAAGGTTTTAGATGATGGGTTCACTTGATCTACTTCCTAATGTTCAATGCATATTCCATTGGTTTTTGGGATTGGTTGTAAGGGCAATTCTGCAGATATCCATCACACTGGCGGCCGCTCGAGCATG

# >NtXTH26 (SGN database sequence)

ATGTCATTATCCTCTGCTTCCTCCAGAATTCCAAAAATGTTCCTTCAGCTCTCTGTTCTTGCAGTTTTCCTCCTATGCACTGCTTGTGCTGATAATTTCTACCAAGACGCGACTGTCACCTGGGGTGACCAGCGGGCTCACATACAAGAAGGTGGCCGTCTTCTAACCTTGTCTCTCGATAAAATTTCAGGCTCTGGCTTTCAATCCAAGAGTGAGTTTTTATTCGGAAGGTTCGACATGCAGCTCAAGTTAATACCTGGAAATTCTGCTGGCACTGTCACCACTTTCTACTTGTCGTCTCAAGGAGCAGGGCAC***GACGAAATTGATTTTGAATTTCTGGGA***AATTCATCAGGCCAGCCTTACACAGTTCACACCAACGTTTATTCTCAGGGAAAAGGCAACAAAGAACAACAATTTCACCTCTGGTTCGATCCCACCACATCGTTTCACACCTACTCTATCATTTGGAACGCTCAACGCATCATATTTTTGGTGGATAACATACCAATAAGAGTGTACAACAATCACGAAGCACTTGGGGTTGCATTTCCAAAGAATCAAGCAATGAGAGTGTACGCTAGCCTATGGAATGCTGATGACTGGGCAACACAAGGCGGGCGAGTGAAAACGGACTGGTCCATGGCTCCATTCACAGCTTCTTACAGGAATTTCAATACAAATGCTTGTGTTTGGTCAGCGGCATCATCTACTTCATCTTGTGGAGGCTCTAAATCCACTGATTCAGCGAATAATGATCAGACATGGCAAACTCAAGAACTGGACGCTAATGGCAGAAATAGGCTTAGATGGGTGCAGCAGAAATACATGACATACAATTACTGTACAGATGCTCAAAGGTTCAATCAAGTCATTCCTCCTGAATGCAAGCGTTCAAGGTTTTAA

## >NtXTH26 amplification (same with SGN database sequence)

TCACAAACATCTCTTGCTCCATTTCTTCATATTAAAGTACAACA**ATG**TCATTATCCTCTGCTTCCTCCAGAATTCCAAAAATGTTCCTTCAGCTCTCTGTTCTTGCAGTTTTCCTCCTATGCACTGCTTGTGCTGATAATTTCTACCAAGACGCGACTGTCACCTGGGGTGACCAGCGGGCTCACATACAAGAAGGTGGCCGTCTTCTAACCTTGTCTCTCGATAAAATTTCAGGCTCTGGCTTTCAATCCAAGAGTGAGTTTTTATTCGGAAGGTTCGACATGCAGCTCAAGTTAATACCTGGAAATTCTGCTGGCACTGTCACCACTTTCTACTTGTCGTCTCAAGGAGCAGGGCACGACGAAATTGATTTTGAATTTCTGGGAAATTCATCAGGCCAGCCTTACACAGTTCACACCAACGTTTATTCTCAGGGAAAAGGCAACAAAGAACAACAATTTCACCTCTGGTTCGATCCCACCACATCGTTTCACACCTACTCTATCATTTGGAACGCTCAACGCATCATATTTTTGGTGGATAACATACCAATAAGAGTGTACAACAATCACGAAGCACTTGGGGTTGCATTTCCAAAGAATCAAGCAATGAGAGTGTACGCTAGCCTATGGAATGCTGATGACTGGGCAACACAAGGCGGGCGAGTGAAAACGGACTGGTCCATGGCTCCATTCACAGCTTCTTACAGGAATTTCAATACAAATGCTTGTGTTTGGTCAGCGGCATCATCTACTTCATCTTGTGGAGGCTCTAAATCCACTGATTCAGCGAATAATGATCAGACATGGCAAACTCAAGAACTGGACGCTAATGGCAGAAATAGGCTTAGATGGGTGCAGCAGAAATACATGACATACAATTACTGTACAGATGCTCAAAGGTTCAATCAAGTCATTCCTCCTGAATGCAAGCGTTCAAGGTTTTAAGACCATCATGTGTTGCTG**TAA**

# >NtXTH27 (SGN database sequence)

ATGGGGTCAAGAATTTTCTTGGTTCTAGCACTTGTGTTTAGTTCTTGCATGGTTTCTTATGGTGGAAATTTCTTTCAAGAATTTGACTTTACTTGGGGTGGAAATAGGGCTAAGATTTTCAATGGAGGTCAGCTTATGTCTTTGTCTTTGGACAAAGTTTCTGGCTCTGGTTTTCAATCTAAGAAAGAGTATCTCTTTGGGAGAATTGATATGCAAATCAAACTTGTTGCTGGAAATTCTGCTGGAACTGTCACTACATACTATTTATCTTCTCAGGGACCCACACAT***GATGAAATTGACTTTGAATTCTTGGGA***AATGTTACTGGTGAACCTTATATTCTCCACACAAACATTTATGCCCAAGGCAAAGGAAACAAAGAGCAGCAATTTTACCTTTGGTTTGATCCTACCAAGAACTTCCACACCTACTCAATCATATGGAAACCCCAACATATCATTTTCTTGGTCGACAACACACCAATAAGAGTTTACAAGAATGCTGAATCCATTGGTGTGCCATTTCCCAAGAACCAGCCCATGAGAATTTACTCTAGCCTTTGGAATGCTGATGATTGGGCAACAAGAGGAGGCCTAGTGAAAACTGATTGGTCTAAAGCACCATTTACAGCCTACTATAGAAATTTCAATTCTCAAACTTTTAGCAGTTCACAATTTTCAAATGAAAAATGGCAAAATCAAGAACTTGATGCCAATGGCAGAAGAAGACTCAGATGGGTGCAGAGGAATTTCATGATTTATAATTATTGTACTGATTTTAAGAGGTTTCCTCAGGGTTTTCCTCCAGAATGCAAAAGATTTTGA

## >NtXTH27 amplification (same with SGN database sequence)

CACAAAAACAAACATCACACATTCTAATTAGTACAAGTAAATTTTTATATAAGTTAAGAGCTAAA**ATG**GGGTCAAGAATTTTCTTGGTTCTAGCACTTGTGTTTAGTTCTTGCATGGTTTCTTATGGTGGAAATTTCTTTCAAGAATTTGACTTTACTTGGGGTGGAAATAGGGCTAAGATTTTCAATGGAGGTCAGCTTATGTCTTTGTCTTTGGACAAAGTTTCTGGCTCTGGTTTTCAATCTAAGAAAGAGTATCTCTTTGGGAGAATTGATATGCAAATCAAACTTGTTGCTGGAAATTCTGCTGGAACTGTCACTACATACTATTTATCTTCTCAGGGACCCACACATGATGAAATTGACTTTGAATTCTTGGGAAATGTTACTGGTGAACCTTATATTCTCCACACAAACATTTATGCCCAAGGCAAAGGAAACAAAGAGCAGCAATTTTACCTTTGGTTTGATCCTACCAAGAACTTCCACACCTACTCAATCATATGGAAACCCCAACATATCATTTTCTTGGTCGACAACACACCAATAAGAGTTTACAAGAATGCTGAATCCATTGGTGTGCCATTTCCCAAGAACCAGCCCATGAGAATTTACTCTAGCCTTTGGAATGCTGATGATTGGGCAACAAGAGGAGGCCTAGTGAAAACTGATTGGTCTAAAGCACCATTTACAGCCTACTATAGAAATTTCAATTCTCAAACTTTTAGCAGTTCACAATTTTCAAATGAAAAATGGCAAAATCAAGAACTTGATGCCAATGGCAGAAGAAGACTCAGATGGGTGCAGAGGAATTTCATGATTTATAATTATTGTACTGATTTTAAGAGGTTTCCTCAGGGTTTTCCTCCAGAATGCAAAAGATTT**TGA**GTATA

# >NtXTH28 (SGN database sequence)

ATGGCAAGGTTTTCGTCTTCTTCATCTAGGTCCAGGTCTTCTCTTCCATACATTGTATTGCTCTTCGTTGCTGCCCTTTTTGTCTTTAAGATAGATGTTATCATATCTCAGACATTTAGTTCAGCCCGTCGCAACCTGGAGAACACTCCTAACCGTATCTTAGTGAAGTCTAAATCCCAAGAAACTGATGACAGTATACCTGTAGTATTAGTAAATGGTACATTTCACCGGCATTTTATATTATCATGGGGAGACGATAGAGGAAAGATACATGAAAATGGAGAACTTTTAACACTCTCCTTAGATAAGCAATCTGGATCAGGATTTCAGTCCAAGAAAGAGTATCTCTTTGCCAAAATTGATATGCAAATTAAGCTCGTCCCTGGAAATTCAGCTGGCACTGTTACTACTTTTTACCTGTCATCACAAGGCAACAAGCAT***GATGAAATAGATTTTGAATTCTTGGGA***AATTCCACAGGAAATCCTTATACTCTTCATACTAATATTTTCAGTCTAGGCCAAGGCAATAGAGAACAACAATTTTTCTTGTGGTTCGATCCTACTGCAGATTACCATACCTATTCAATCCTTTGGAATCCAAAATGTATTATATTCTATGTTGATGGTACACCAATTAGGGAGTTCAAAAATGCAGAAAAAATTGGTGTTCCATTTCTAAAATACCAACCAATGAGACTATACTCAAGTCTATGGAATGCAGATGATTGGGCTACACAAGGTGGTCGTGTTAAAACTAACTGGAAATTAGCACCTTTTATTGCTTCTTACAAAAATTTTACTTATGAAGCCTGCATTTATTCAAGATTAACTAGTTCGTCTTCGTGCAATATCAACTCTCCTCCTTTTGGTAACAACGCGTGGCTAACACACGAATTGGATCGAAGAAGTCGAGCAAAAATGAAAATTTTGCAGAAAAAACATATGATTTATGATTATTGTAAGGATAAATGGAGGTTTCCTAAAGGACCTGCTCCTGAATGCAAGCTTCAATAA

## >NtXTH28 amplification (64 bp insertion and 10 bp deletion)

GGGCGATTGGGCCCTCTAGATGCATGCTCGAGCGGCCGCCAGTGTGATGGATATCTGCAGAATTGCCCTTATGAACACCGAAGTTCCATTTGTATACAATAATTATGAGTAATACAAAAAGAATGCAATATGAGGTTTCAAAGAATCATATTATCAAATTATTTCA**ATG**GCAAGGTTTTCGTCTTCTTCATCTAGGTCCAGGTCTTCTCTTCCATACATTGTATTGCTCTTCGTTGCTGCCCTTTTTGTCTTTAAGATAGATGTTATCATATCTCAGACATTTAGTTCAGCCCGTCGCAACCTGGAGAACACTCCTAACCGTATCTTAGTGAAGTCTAAATCCCAAGAAACTGATGACAGTATACCTGTAGTATTAGTAAATGGTACATTTCACCGGCATTTTATATTATCATGGGGAGACGATAGAGGAAAGATACATGAAAATGGAGAACTTTTAACACTCTCCTTAGATAAGCAATCTGGATCAGGATTTCAGTCCAAGAAAGAGTATCTCTTTGCCAAAATTGATATGCAAATTAAGCTCGTCCCTGGAAATTCAGCTGGCACTGTTACTACTTTTTACCTGTCATCACAAGGCAACAAGCATGATGAAATAGATTTTGAATTCTTGGGAAATTCCACAGGAAATCCTTATACTCTTCATACTAATATTTTCAGTCTAGGCCAAGGCAATAGAGAACAACAATTTTTCTTGTGGTTCGATCCTACTGCAGATTACCATACCTATTCAATCCTTTGGAATCCAAAATGTATTATATTCTATGTTGATGGTACACCAATTAGGGAGTTCAAAAATGCAGAAAAAATTGGTGTTCCATTTCTAAAATACCAACCAATGAGACTATACTCAAGTCTATGGAATGCAGATGATTGGGCTACACAAGGTGGTCGTGTTAAAACTAACTGGAAATTAGCACCTTTTATTGCTTCTTACAAAAATTTTACTTATGAAGCCTGCATTTATTCAAGATTAACTAGTTCGTCTTCGTGCAATATCAACTCTCCTCCTTTTGGTAACAACGCGTGGCTAACACACGAATTGGATCGAAGAAGTCGAGCAAAAATGAAAATTTTGCAGAAAAAACATATGATTTATGATTATTGTAAGGATAAATGGAGGTTTCCTAAAGGACCTGCTCCTGAATGCAA**AAGGGCAATTCCAGCACACTGGCGGCCGTTACTAGTGGATCCGAGCTCGGTACCAAGCTTGGCG**~~GCTTCAATAA~~**TAA**TCATGGTCATAGCTGTTTCCTGTGTGAAATTGTTATCCGCTCACAATTCCACACAACATACGAGCCGGAAGCATAAAGTGTAAAGCCTGGGGTGCCTAATGAGTGAGCTAACTCACATTAATTGCGTTGCGCTCACTGCCCGCTTTCCAGTCGGGAAACCTGTCGTGCCAGCTGCATTAATGAATCGGCCAACGCGCGGGGAGAGGCGGTTTGCGTATTGGGCGCTCTTCCGCTTCCTCGCTCACTGACTCGCTGCGCTCGGTCGTTCGGCTGCGGCGAGCGGTATCAGCTCACTCAAAGGCGGTAATACGGTTATCCACAGAATCAGGGGATAACGCAGGAAAGAACATGTGAGCAAAAGGCCAGCAAAAGGCCAGGAACCGTAAAAAGGCCGCGTTGCTGGCGTTTTTCCATAGGCTCCGCCCCCCTGACGAGCATCACAAAAATCGACGCTCAAGTCAGAGGTGGCGAAACCCGACAGGACTATAAAGATACCAGGCGTTTCCCCCTGGAAGCTCCCTCGTGCGCTCTCCTGTTCCGACCCTGCCGCTTACCGGATACCTGTCCGCCTTTCTCCCTTCGGGAAGCGTGGCGCTTTCTCATAGCTCACGCTGTAGGTATCTCAGTTCGGTGTAGGTCGTTCGCTCCAAGCTGGGCTGTGTGCACGAAC

# >NtXTH29 (SGN database sequence)

ATGGCAAGGTTTTCATCTTCTTCATCTAGGTCTAGGTCTTCTCTTCCATACATTATATTGCTCTTCGTTGCTGCCCTTTTTGTCTTTAAGATAGATGTTATCATATCTCAGTCGTTTAGTTCAGCCCGTCGCAACCTGGAGAACACCCCTAATCATATCTTGGTGAAGTCTAAATCCCAAGAAACTGATGACAGTATACCTGTAGTATTAGTAAATGGTACATTTCACCGGCATTTTATATTATCATGGGGAGACGATAGAGGAAAGATACATGAAAATGGAGAACTTTTAACACTTTCCTTAGACAAGCTATCTGGATCAGGATTTCAGTCCAAGAAAGAGTATCTCTTTGCCAAAATTGATATGCAAATTAAGCTCGTTCCTGGAAATTCAGCTGGCACTGTTACTACTTTTTACCTATCATCACAAGGAAACAAGCAT***GATGAAATAGACTTTGAATTCTTGGGA***AATTCAACAGGAAATCCTTATACTCTTCATACAAATATTTTCAGTTTAGGCCAAGGCAATAGAGAACAACAATTTTTCTTGTGGTTTGATCCTACTGCAGATTACCATACCTATTCAATCCTTTGGAATCCAAAATGTATTATATTCTATGTTGATGGTACACCAATTAGGGAATACAAAAATGCAGAAAAAATTGGTGTTCCATTTCCAAAATACCAACCAATGAGACTATACTCAAGTCTATGGAATGCAGATGATTGGGCTACACAAGGTGGTCGTATTAAAACTAATTGGAAATTAGCACCTTTTATTGCTTCTTACAAAAATTTTACTTATGATGCTTGCATTTATTCAAGATTAACTAGTTCATCTTCGTGCAATATCAACTCTCCTCCTTTTGGTAATGACTCGTGGCTAACGCACGAATTGGATCGAAGAAGTCGAGCAAAAATGAAAATTTTGCAGAAAAAACATATGATTTATGATTATTGTAATGATAAATGGAGGTTTCCTAAAGGACCTGCGCCTGAATGCAAGCTTCAATAA

## >NtXTH29 amplification (same with SGN database sequence)

GCAATATGAAGTTTCAAAAATTCATATTATCAAATTAGTTCA**ATG**GCAAGGTTTTCATCTTCTTCATCTAGGTCTAGGTCTTCTCTTCCATACATTATATTGCTCTTCGTTGCTGCCCTTTTTGTCTTTAAGATAGATGTTATCATATCTCAGTCGTTTAGTTCAGCCCGTCGCAACCTGGAGAACACCCCTAATCATATCTTGGTGAAGTCTAAATCCCAAGAAACTGATGACAGTATACCTGTAGTATTAGTAAATGGTACATTTCACCGGCATTTTATATTATCATGGGGAGACGATAGAGGAAAGATACATGAAAATGGAGAACTTTTAACACTTTCCTTAGACAAGCTATCTGGATCAGGATTTCAGTCCAAGAAAGAGTATCTCTTTGCCAAAATTGATATGCAAATTAAGCTCGTTCCTGGAAATTCAGCTGGCACTGTTACTACTTTTTACCTATCATCACAAGGAAACAAGCATGATGAAATAGACTTTGAATTCTTGGGAAATTCAACAGGAAATCCTTATACTCTTCATACAAATATTTTCAGTTTAGGCCAAGGCAATAGAGAACAACAATTTTTCTTGTGGTTTGATCCTACTGCAGATTACCATACCTATTCAATCCTTTGGAATCCAAAATGTATTATATTCTATGTTGATGGTACACCAATTAGGGAATACAAAAATGCAGAAAAAATTGGTGTTCCATTTCCAAAATACCAACCAATGAGACTATACTCAAGTCTATGGAATGCAGATGATTGGGCTACACAAGGTGGTCGTATTAAAACTAATTGGAAATTAGCACCTTTTATTGCTTCTTACAAAAATTTTACTTATGATGCTTGCATTTATTCAAGATTAACTAGTTCATCTTCGTGCAATATCAACTCTCCTCCTTTTGGTAATGACTCGTGGCTAACGCACGAATTGGATCGAAGAAGTCGAGCAAAAATGAAAATTTTGCAGAAAAAACATATGATTTATGATTATTGTAATGATAAATGGAGGTTTCCTAAAGGACCTGCGCCTGAATGCAAGCTTCAA**TAA**CAATATAGGAAAATTATAACTT

# >NtXTH30 (SGN database sequence)

ATGATGAAAACTTCAAGTTGTATGTTTTCTTTCTTGTTTCTGAGTTTCTTGGTGTTGGTGGCTTTGGCAGAAAATTTCAACCAAGAATTTGATGTTACATGGGGTGATGGCAGGGTAAAAATACTTGAAAATGGGCAGCTTCTCACCCTTTCCCTTGACAAAACTTCAGGCTCTGGATTTAGGTCAAAAAGACAATATATGTTTGGAAAGATTGACATGAAGATCAAACTTGTTCCTGGCAATTCTGCAGGCACTGTTACTACATACTATTTATCTTCACTTGGACCGACTCAT***GACGAGATTGACTTTGAGTTTCTAGGC***AACCTAAGTGGAGACCCTTATATTCTTCATACAAATGTGTTCGTACAAGGCAAGGGGGAGAGAGAGCAACAGTTTTATCTTTGGTTCGACCCCACTAAGGATTTTCACACCTACTCTATTCTCTGGAATCCTCGAAGCATCATATTTTCAGTAGATGGGACGCCAATTAGGCAATTCAAGAATCTTGAAGCTTCAAGGGGAATACCTTATCCCAAAAATCAACCAATGTGGATATACTCAAGCTTATGGGATGCTGAAGATTGGGCAACAAGAGGAGGACTTGTCAAAACTGATTGGAGCAAAGCCCCTTTCATTGCTTCTTACAGAAATTTTAATGCCCAAGCATGTGTTTGGTCTTCTGGTTCTACTTCTTCTTGCTCCATAAATTCCACAGCCAATTCTTGGATAACTGAATCATTGGATAACTCTGGCCAAGCAAGGATTAAATGGGTGCAAAAGAATTACATGGTCTATAACTACTGCACTGATACTAAACGTTTCCCTCAAGGATTTCCCCTTGAATGCTCTCTAAATTAA

## >NtXTH30 amplification (same with SGN database sequence)

TATCATTATTTGAACCATATTAGTCACTGTCCCCTTTCTTTAAAAGGTGTAAAGTAAAGCCAATAAACACC**ATG**ATGAAAACTTCAAGTTGTATGTTTTCTTTCTTGTTTCTGAGTTTCTTGGTGTTGGTGGCTTTGGCAGAAAATTTCAACCAAGAATTTGATGTTACATGGGGTGATGGCAGGGTAAAAATACTTGAAAATGGGCAGCTTCTCACCCTTTCCCTTGACAAAACTTCAGGCTCTGGATTTAGGTCAAAAAGACAATATATGTTTGGAAAGATTGACATGAAGATCAAACTTGTTCCTGGCAATTCTGCAGGCACTGTTACTACATACTATTTATCTTCACTTGGACCGACTCATGACGAGATTGACTTTGAGTTTCTAGGCAACCTAAGTGGAGACCCTTATATTCTTCATACAAATGTGTTCGTACAAGGCAAGGGGGAGAGAGAGCAACAGTTTTATCTTTGGTTCGACCCCACTAAGGATTTTCACACCTACTCTATTCTCTGGAATCCTCGAAGCATCATATTTTCAGTAGATGGGACGCCAATTAGGCAATTCAAGAATCTTGAAGCTTCAAGGGGAATACCTTATCCCAAAAATCAACCAATGTGGATATACTCAAGCTTATGGGATGCTGAAGATTGGGCAACAAGAGGAGGACTTGTCAAAACTGATTGGAGCAAAGCCCCTTTCATTGCTTCTTACAGAAATTTTAATGCCCAAGCATGTGTTTGGTCTTCTGGTTCTACTTCTTCTTGCTCCATAAATTCCACAGCCAATTCTTGGATAACTGAATCATTGGATAACTCTGGCCAAGCAAGGATTAAATGGGTGCAAAAGAATTACATGGTCTATAACTACTGCACTGATACTAAACGTTTCCCTCAAGGATTTCCCCTTGAATGCTCTCTAAAT**TAA**AAA

# >NtXTH31 (SGN database sequence)

ATGATGAAAACTTCAATTAGTTGTATAATTTCTTTCTTGTTTCTGAGTTTCTTGCTGGTGGTGATGGCGGCTTTGGCTGGAGATTTCAACCAAGAATTTGATGTTACATGGGGTGATGGCAGGGTAAAAATACTTGAAAACGGGCAGCTTCTCACCCTTTCCCTTGACAAAACTTCAGGTTCGGGGTTTAGGTCAAAAAGACAGTATATGTTTGGAAAGATTGACATGAAGATCAAACTTGTTCCTGGCAATTCTGCAGGCACTGTTACTACATACTATTTATCTTCGCTGGGACCGACTCAT***GACGAGATTGACTTTGAGTTCCTTGGC***AACCTAAGTGGAGACCCTTATATTCTTCATACAAATGTGTTCACACAAGGCAAAGGAGACAGAGAGCAACAATTTTATCTTTGGTTCGACCCCACTAAGGATTTTCACACATACTCTATTCTTTGGAATCCTCGAAGCATCATATTTTCAGTAGATGGGACACCAATTAGACAATTCAAGAATCTTGAAACTTCAATGGGAATACCTTATCCAAAAAATCAACCAATGTGGATATACTCAAGCTTATGGGATGCTGAAGATTGGGCAACAAGAGGCGGACTTGTCAAAACCGATTGGAGCCAAGCCCCTTTTGTTGCTTCTTACAGAAATTTTAATGCCCAAGCATGTGTTTGGTCTTCTGGTTCTACTTCTTCCTGCTCCAGAAATTCCACAGCTAATTCTTGGATAACTGAATCATTGGATAACTCTGGCCAAGCAAGGATTAAATGGGTGCAAAAGAATTACATGGTTTATAACTACTGCACTGATATTAAACGTTTCCCTCAAGGATTTCCCCTTGAATGCTCTCTAAATTAA

## >NtXTH31 amplification (same with SGN database sequence)

ATCAAACGTACACCAATCC**ATG**ATGAAAACTTCAATTAGTTGTATAATTTCTTTCTTGTTTCTGAGTTTCTTGCTGGTGGTGATGGCGGCTTTGGCTGGAGATTTCAACCAAGAATTTGATGTTACATGGGGTGATGGCAGGGTAAAAATACTTGAAAACGGGCAGCTTCTCACCCTTTCCCTTGACAAAACTTCAGGTTCGGGGTTTAGGTCAAAAAGACAGTATATGTTTGGAAAGATTGACATGAAGATCAAACTTGTTCCTGGCAATTCTGCAGGCACTGTTACTACATACTATTTATCTTCGCTGGGACCGACTCATGACGAGATTGACTTTGAGTTCCTTGGCAACCTAAGTGGAGACCCTTATATTCTTCATACAAATGTGTTCACACAAGGCAAAGGAGACAGAGAGCAACAATTTTATCTTTGGTTCGACCCCACTAAGGATTTTCACACATACTCTATTCTTTGGAATCCTCGAAGCATCATATTTTCAGTAGATGGGACACCAATTAGACAATTCAAGAATCTTGAAACTTCAATGGGAATACCTTATCCAAAAAATCAACCAATGTGGATATACTCAAGCTTATGGGATGCTGAAGATTGGGCAACAAGAGGCGGACTTGTCAAAACCGATTGGAGCCAAGCCCCTTTTGTTGCTTCTTACAGAAATTTTAATGCCCAAGCATGTGTTTGGTCTTCTGGTTCTACTTCTTCCTGCTCCAGAAATTCCACAGCTAATTCTTGGATAACTGAATCATTGGATAACTCTGGCCAAGCAAGGATTAAATGGGTGCAAAAGAATTACATGGTTTATAACTACTGCACTGATATTAAACGTTTCCCTCAAGGATTTCCCCTTGAATGCTCTCTAAAT**TAA**TGCAAGAAAGAAAATCACAAAAATATCGT

# >NtXTH32 (SGN database sequence)

ATGATGAAATCTTTCTTGTTTCAGATGATGTTTTTGGTGGTGGCTTTTGCTGGAAATTTCAACCAAAATTTTGATATTACATGGGGTGATGGCCGAGCTAAAATACTCGAAAACGGACAACTTCTTACCCTTTCCCTTGATAAAACCTCTGGCTCTGGTTTCCGATCCAAAAATCAGTATTTGTTTGGAAAGATTGATTTGAAAATCAAACTTGTCCCTGGTAATTCTGCTGGCACCGTTACTACATATTATTTATCTTCAATAGGATCAAGTCAT***GACGAGATTGATTTCGAGTTTCTTGGG***AATCTAAGTGGAGATCCCTATATTCTTCACACAAATGTATTCACACAAGGGAAGGGAAATAGAGAGCAGCAGTTTTATCTTTGGTTCGACCCTACTAAGTACTTTCATACTTATTCTATTCTTTGGAATCCTCAGAGCATCATCTTTTCAGTAGATGGGACACCAATTAGGCAATTCAAGAATTTAGAAGCAAGTGGGATACCTTATCCAAAGAACCAACCAATGTGGATATACTCAAGCTTATGGAATGCAGATGATTGGGCAACAAGAGGAGGATTAGTTAAGACTGATTGGAGCAAAGCCCCATTTATAGCTTCTTACAGAAATTACAATGCCCAAGCTTGTGTATGGTCTTCAACTTCTTCTTCTTCCTGCAGCCCCAACAACTCCACAGAAAATTCTTGGCTAAGTGAATCCTTGGATAACACAGGCCAATCTAAGATTAAATGGGTGCAAAATAATTACATGATTTATAATTATTGCACTGATACTAAACGCTTCCCTCAAGGATTTCCTCCTGAATGTTCTCTCAATTAG

## >NtXTH32 amplification (same with SGN database sequence)

CTCGGATCACTAGTAACGGCCGCCAGTGTGCTGGAATTGCCCTTTCCTTGAAACATATTAACAAGCAACACTGGCTTTGAAATCTCGAATCAATATATTAATATTTTACACC**ATG**ATGAAATCTTTCTTGTTTCAGATGATGTTTTTGGTGGTGGCTTTTGCTGGAAATTTCAACCAAAATTTTGATATTACATGGGGTGATGGCCGAGCTAAAATACTCGAAAACGGACAACTTCTTACCCTTTCCCTTGATAAAACCTCTGGCTCTGGTTTCCGATCCAAAAATCAGTATTTGTTTGGAAAGATTGATTTGAAAATCAAACTTGTCCCTGGTAATTCTGCTGGCACCGTTACTACATATTATTTATCTTCAATAGGATCAAGTCATGACGAGATTGATTTCGAGTTTCTTGGGAATCTAAGTGGAGATCCCTATATTCTTCACACAAATGTATTCACACAAGGGAAGGGAAATAGAGAGCAGCAGTTTTATCTTTGGTTCGACCCTACTAAGTACTTTCATACTTATTCTATTCTTTGGAATCCTCAGAGCATCATCTTTTCAGTAGATGGGACACCAATTAGGCAATTCAAGAATTTAGAAGCAAGTGGGATACCTTATCCAAAGAACCAACCAATGTGGATATACTCAAGCTTATGGAATGCAGATGATTGGGCAACAAGAGGAGGATTAGTTAAGACTGATTGGAGCAAAGCCCCATTTATAGCTTCTTACAGAAATTACAATGCCCAAGCTTGTGTATGGTCTTCAACTTCTTCTTCTTCCTGCAGCCCCAACAACTCCACAGAAAATTCTTGGCTAAGTGAATCCTTGGATAACACAGGCCAATCTAAGATTAAATGGGTGCAAAATAATTACATGATTTATAATTATTGCACTGATACTAAACGCTTCCCTCAAGGATTTCCTCCTGAATGTTCTCTCAAT**TAG**TGCGAGTAAATTTATATGTTTAAGTGTTAAAACTGTTTTTTTTTTCTTTAGGTTTGCATTGTAATAAAATGTAGGGTTCCAGCTGAGTATAAAGGGCAATTCTGCAGATATCCATCACACTGGCGGCCGCTCGAGCATGCATCTAGAGGGC

# >NtXTH33 (SGN database sequence)

ATGATGAAATCTTTCTTGTTTCTGATGATATTTTTGGTGGTGGCTTTGGCTGGAAATTTCAACAAAGATTTTGATATTACATGGGGTGATGGCCGAGCTAAAATACTCGAAAACGGACAACTTCTCACCCTTTCCCTCGATAAAACCTCTGGCTCTGGTTTTCGGTCCAAAAATCAGTATTTGTTTGGAAAGATTGATTTGAAAATCAAACTTGTCCCTGGTAATTCTGCTGGCACCGTTACTACATATTATTTATCTTCAATAGGATCAAGTCAT***GACGAGATTGATTTCGAGTTTCTTGGG***AATCTAAGTGGAGACCCCTATATTCTTCACACAAATGTATTCACACAAGGGAAGGGAAATAGAGAGCAGCAGTTTTATCTTTGGTTCGATCCTACTAAGGACTTTCATACTTATACTATTCTTTGGAATCCTCAGAGCATCATCTTTTCAGTAGATGGGACACCAATTAGGCAATTCAAGAATTTAGAAGCAAGTGGGATACCTTATCCAAAGAATCAACCAATGTGGATATACTCAAGCTTATGGAATGCAGATGATTGGGCAACAAGAGGAGGACTAGTTAAGACTGATTGGAGCAAAGCCCCATTTATAGCTTCCTACAGAAATTACAATGCCCAAGCTTGTGTATGGTCTTCAAGTTCTTCTTCTTCCTGCACCTCTAACAGTTCCACAGGGAATTCTTGGCTAAGTGAATCATTGGATAGCACAGGCCAATCTAGGATTAAATGGGTGCAAAGTAATTATATGATTTATAATTATTGCACCGATACTAAACGCTTCCCGCAAGGATTTCCCCCTGAATGCTCTCTCAATTAG

## >NtXTH33 amplification (same with SGN database sequence)

CTAGATGCATGCTCGAGCGGCCGCCAGTGTGATGGATATCTGCAGAATTGCCCTTCTATATATATATAAAGGCTAATCGCGTCCATGCAATTCAAAAACACAAAATACTTCTTTGAAACATATTCACAACTTATATAAATATTTTACAAC**ATG**ATGAAATCTTTCTTGTTTCTGATGATATTTTTGGTGGTGGCTTTGGCTGGAAATTTCAACAAAGATTTTGATATTACATGGGGTGATGGCCGAGCTAAAATACTCGAAAACGGACAACTTCTCACCCTTTCCCTCGATAAAACCTCTGGCTCTGGTTTTCGGTCCAAAAATCAGTATTTGTTTGGAAAGATTGATTTGAAAATCAAACTTGTCCCTGGTAATTCTGCTGGCACCGTTACTACATATTATTTATCTTCAATAGGATCAAGTCATGACGAGATTGATTTCGAGTTTCTTGGGAATCTAAGTGGAGACCCCTATATTCTTCACACAAATGTATTCACACAAGGGAAGGGAAATAGAGAGCAGCAGTTTTATCTTTGGTTCGATCCTACTAAGGACTTTCATACTTATACTATTCTTTGGAATCCTCAGAGCATCATCTTTTCAGTAGATGGGACACCAATTAGGCAATTCAAGAATTTAGAAGCAAGTGGGATACCTTATCCAAAGAATCAACCAATGTGGATATACTCAAGCTTATGGAATGCAGATGATTGGGCAACAAGAGGAGGACTAGTTAAGACTGATTGGAGCAAAGCCCCATTTATAGCTTCCTACAGAAATTACAATGCCCAAGCTTGTGTATGGTCTTCAAGTTCTTCTTCTTCCTGCACCTCTAACAGTTCCACAGGGAATTCTTGGCTAAGTGAATCATTGGATAGCACAGGCCAATCTAGGATTAAATGGGTGCAAAGTAATTATATGATTTATAATTATTGCACCGATACTAAACGCTTCCCGCAAGGATTTCCCCCTGAATGCTCTCTCAAT**TAG**TGCTAGTAAAGGGCAATTCCAGCACACTGGCGGCCGTTACTAGTgGATCCGAGCTC

# >NtXTH34 (SGN database sequence)

ATGTCTTCTTTTTCTTCTAAATTAGTACTAGCTCTTATTGTTAGTGCTTTCGCTATTGCAATTGCGGGTACTATTGACGAAAATTTTGAAATTACATGGGGTGAAGGCAGAGCAAAGATGCTAAATAATGGAGAGCTTCTAACTCTATCACTTGACAAAATCTCAGGCTCAGGATTTCAATCCAAGAATGAATATCTCTTTGGTAAAATAGACATGCAACTCAAACTTGTCCCTGGAAATTCTGCTGGCACTGTCACTGCTTACTATTTGTCATCACAAGGACCAACACAT***GATGAAATAGATTTTGAATTCTTGGGA***AATCTAAGTGGTGATCCTTATACACTTCACACTAATGTATTTAGCCAAGGCAAAGGCAACAGAGAGCAACAATTCCATCTTTGGTTTGACCCTACTGCTGATTTCCACACTTATTCCATCCTCTGGAATCCACAACGCATCATATTTTATGTAGATGGAACACCAATTAGAGAATACAAGAATGCAGAATCAATTGGAGTATCATATCCAAAGAAGCAACCAATGAGAATATACTCAAGTCTATGGAATGCAGATGATTGGGCTACAAGAGGAGGACTTATTAAAACTGATTGGAGTAAAGCACCCTTTAGTGCTTCCTACAGAAACTTCAAATCTGCAACTTCAACCTCTGCAGCCACTAGCAATTCATGGTTGAATGAAGAGTTGGATAATACAAGTCAAGAAAGGCTGAAATGGGTGCAGAAAAATTATATGGTTTACAATTACTGCAATGATTCCAAGAGATTTCCACAGGGATTTCCTGCAGATTGTGCTATGTAA

## >NtXTH34 amplification (same with SGN database sequence)

TCTAGATGCATGCTCGAGCGGCCGCCAGTGTGATGGATATCTGCAGAATTGCCCTTTTTGAACTTTCCTCTTCACTTCCTTTAGCCTAACCCCACACGCTCTACTTCTTTGTTGGTGAGGTGGGTTGGATTTTTTAATTTTTTTTTTTTTTTGGGGGGGGGGGGGGGGTATAAACCCCTTATAAAAACCTACCTAGTTATCCATTCTAACTCATCCAAACAAACTTGAAAATTCTTGAAATATTAGGAATTAAAG**ATG**TCTTCTTTTTCTTCTAAATTAGTACTAGCTCTTATTGTTAGTGCTTTCGCTATTGCAATTGCGGGTACTATTGACGAAAATTTTGAAATTACATGGGGTGAAGGCAGAGCAAAGATGCTAAATAATGGAGAGCTTCTAACTCTATCACTTGACAAAATCTCAGGCTCAGGATTTCAATCCAAGAATGAATATCTCTTTGGTAAAATAGACATGCAACTCAAACTTGTCCCTGGAAATTCTGCTGGCACTGTCACTGCTTACTATTTGTCATCACAAGGACCAACACATGATGAAATAGATTTTGAATTCTTGGGAAATCTAAGTGGTGATCCTTATACACTTCACACTAATGTATTTAGCCAAGGCAAAGGCAACAGAGAGCAACAATTCCATCTTTGGTTTGACCCTACTGCTGATTTCCACACTTATTCCATCCTCTGGAATCCACAACGCATCATATTTTATGTAGATGGAACACCAATTAGAGAATACAAGAATGCAGAATCAATTGGAGTATCATATCCAAAGAAGCAACCAATGAGAATATACTCAAGTCTATGGAATGCAGATGATTGGGCTACAAGAGGAGGACTTATTAAAACTGATTGGAGTAAAGCACCCTTTAGTGCTTCCTACAGAAACTTCAAATCTGCAACTTCAACCTCTGCAGCCACTAGCAATTCATGGTTGAATGAAGAGTTGGATAATACAAGTCAAGAAAGGCTGAAATGGGTGCAGAAAAATTATATGGTTTACAATTACTGCAATGATTCCAAGAGATTTCCACAGGGATTTCCTGCAGATTGTGCTATG**TAA**CAGCTGAGATCTTAAGGGCAATTCCAGCACACTGGCGGCCGTTACTAGTGGATCCGAGCTCG

# >NtXTH35 (SGN database sequence)

ATGGCTTCTTTGTTAGCTCAATATTTGGTTTTTCTTGCCTTATGCTCTTTGCAATATCATAGTTTGGCTTATAATAACTTTAATCAAGATTTTGATGTTACATGGGGAGATGGTAGGGCAAAGGTTCTCAACAATGGAAAACTTCTTACTCTCTCCCTTGACAAAGCCTCTGGTTCCGGTATTCAATCCAAGAGAGAGTATTTATTTGGAAGGATTGATATGCAGTTGAAACTCGTACGTGGAAATTCAGCTGGCACTGTTACTACATATTACTTATCATCACAAGGGGCAACACAT***GATGAGATAGATTTTGAATTCTTGGGC***AATCTTAGTGGTGATCCTTATATTATTCATACAAATGTTTACACTCAAGGCAAAGGTGACAAAGAACAGCAATTCTACTTATGGTTTGATCCAACTGCTGGTTTTCATACCTACTCCATTCTTTGGAACCCACAAACAATTATATTTTATGTGGATGGCACACCTATAAGAGTGTTCAAGAACATGAAGTCAAGAGGAATACCATACCCAAACAAGCAACCAATGAGAGTATATGCAAGTCTATGGAATGCAGATGATTGGGCTACTAGGGGTGGCCTAATTAAAACAGATTGGTCCAATGCTCCATTTATAGCCTCTTTTAGAAATTTCAAAGCCAATGCTTGTGTTTGGGAATTTGGAAAATCATCATGTAATAGTAGCACAAATCCATGGTTTTTTCAAGAACTTGATTCAACAAGCCAAGCTAAGTTACAATGGGTGCAGAAAAATTATATGGTTTATAATTATTGTACTGATATTAAAAGGTTTCCTCAAGGTTTTCCTCTAGAATGTAATTTCAACTCCACAACTAGTTAA

## >Amplification failed

# >NtXTH36 (SGN database sequence)

ATGGCTTCTTTGTTAGTTCAATGTTTGAATTTTCTTGCCTTATGCTCTTTGCAATATCATATCTTGGCTTCTAGTAATTTTAATCAAGATTTTGATGTTACATGGGGAGATGGTAGGGCAAAGGTTCTCAACAATGGAAAACTTCTTACCCTCTCTCTTGACAAAGCCTCTGGTTCTGGTATTCAATCCAAGAGAGAGTATTTATTTGGAAGGATCGATATGCAGTTGAAACTCGTACGTGAAAACTCAGCCGGCACAGTTACTACATATTATTTATCATCACAAGGGGCAACACAC***GATGAGATAGATTTCGAATTCTTGGGA***AATCTTAGTGGTGATCCATATATTATTCATACAAATGTTTACACTCAAGGCAAAGGTGACAAAGAACAACAGTTCTACTTATGGTTTGATCCCACTGCTGGTTTTCATACCTACTCCATTCTTTGGAACCCCCAAACAATTATATTTTATGTGGATGGTACACCAATAAGAGTGTTCAAGAACATGAAGTCAAGTGGGGTACCCTACCCAACCAACCAACCTATGAGGGTATATGCAAGTCTATGGAATGCAGATGATTGGGCTACTAGGGGTGGCCTTATTAAAACAGATTGGTCCAAAGCTCCATTTATAGCTTCTTTTAGAAATTTCAAAGCCAATGCTTGTGTTTGGGAATTTGGAAAATCATCATGCAATAGTAGCACAAATTCCACAAAGCCATGGTTTTTTCAAGAACTTGATTCCACAAGCCAAGCTAGGTTACAATGGGTGCAGAAAAATTATATGGTTTATAATTATTGTACTGATATTAAAAGGTTTCCTCAAGGTCTTCCTCAAGAATGCAATTTCAACTCCACGACTAGTTAA

## >NtXTH36 amplification (Not NtXTH36 sequence)

CAGGCTTTCCATTTATGCTTCCGGCTCGTATGTTGTGTGGAATTGTGAGCGGATAACAATTTCACACAGGAAACAGCTATGACCATGATTACGCCAAGCTTGGTACCGAGCTCGGATCCACTAGTAACGGCCGCCAGTGTGCTGGAATTGCCCTTCCGTCAATTACAAGAGGAATATGAATGTTCTATCATGTTCCATCAATACTCTAAAGGGGTTATACGATATATCCGGTGTGGAAGTAGGCCAACATTTCTATTGGCAAATAGGGGGTTTCCAAGTACATGGCCAAGTACTTATTACTTCTTGGGTTGTAATTGCTATCTTATTAGGTTCAGCCACTATAGCTGTTCGGAACCCACAAACCATTCCGACCGGGGGTCAGAATTTCTTCGAATATGTTCTTGAATTTATTCGAGATGTGAGTAAAACTCAAATTGGCGAAGAATATGGGCCCTGGGTTCCTTTTATTGGCACTATGTTTTTATTTATTTTTGTTTCTAATTGGTCAGGAGCTCTTTTACCTTGGAAAATCATACAATTACCTCATGGGGAGTTAGCCGCACCCACGAATGATATAAATACTACTGTTGCTTTGGCTTTACTCACATCAGTGGCATATTTCTATGCGGGTCTTACAAAAAAAGGATTAGGTTATTTCGGAAAATATATTCAACCAACCCCAATCCTTTTACCCATTAACATCTTAGAAGATTTCACAAAACCTTTATCACTTAGTTTTCGACTTTTCGGGAATATCTTAGCTGATGAATTAGTAGTTGTTGTTCTTGTTTCTTTAGTACCTTTAGTAGTTCCTATACCTGTCATGCTCCTTGGATTATTTACAAGTGGTATTCAAGCTCTTATTTTTGCAACTTTAGCCGCGGCTTATATAGGTGAATCCATGGAGGGCCATTTTGGTAATAAGGGCAATTCTGCAGATATCCATCACACTGGCGGCCGCTCGAGCATGCATCTAGAGGGCCCAATTCGCCCTATAGTGAGTCGTATTACAATTCACTGGCCGTCGTTTTACAACGTCGTGACTGG

# >NtXTH37 (SGN database sequence)

ATGGCCAAATTCATAGCTTTTAATTCCTTGGTTTTGATCATTGCAACATTTGCATTTCATTGTGCTATAGTCAATGCAAAGATCTCAAGTAGCATGTATATCAATTGGGGTGCTCATCATTGTCAAATGCTTGGGGATGATCTTCAACTTGTCCTTGATAAATCTGCAGGTTCTGGTGCGCAATCAAAGAGAACATTTCTCTTTGGTAGCTTTGAAATGCTTATCAAGTTGGTACCTAACAACTCCGCTGGAACTGTTACAACATACTATCTATCTTCTACTGGCACCAAACAT***GATGAGATTGGTTTCGAGTTTTTAGGA***AATGTATCAGGACAACCTTACATTATCCACACAAACATTTACACCCAAGGTGTTGGAAACAAGGAGCAGCAATTCTATCCTTGGTTTGATCCAACTGCAGATTTTCACAACTACACCATTCATTGGAATCCTAATGCAGTCGTATGGTATATTGATGGTATTCCAATTAGGGTATTTAGAAATTATCAACTCAAAGGAATTCCATTTCCAAACCAACAAGGAATGAGAATATACTCTAGCCTTTGGAATGCAGATGAATGGGCAACAAGAGGTGGACGTGATAAAATTGATTGGACAAATGCACCATTTATTGCAACATATCGTAAGTTTAGGCCAAGAGCTTGTTATTGGAATGGACCATTGAGTATTGTTCAATGTGCTATTCCTACTAAATCCAATTGGTGGAATTTTCCTTTATACAGTAAATTGAGTGCTCCTAAAGTGGATCAAATGAACTCAATTAGGAGCAAATACATGATTTATGATTATTGCAAAGATACTACACGATTTAAGGGAGTTATGCCTACTGAGTGTACATTGCCACAAAACTAG

## >Amplification failed

# >NtXTH38 (SGN database sequence)

ATGGCCAAATTCATAGCTTTTAATTCCTTGGTTTTGATCATTGCTACAATTGCTTTTCATTGTGCTATAGTCAATGGAAAGATTTCAAGTAGCATGTATGTTAATTGGGGTGCTCATCATTGTCAAATGCTAGGGGATGATCTTCAACTTGTCCTTGATAAATCTGCAGGTTCTGGTGCGCAATCAAAAAGAACATTTCTCTTTGGTAGCTTTGAAATGCTTATCAAGTTGGTACCTAACAACTCAGCTGGAACAGTCACAACATACTATTTATCTTCTACGGGTACCAAGCAT***GATGAAATCGACTTCGAGTTTTTAGGA***AATGTATCAGGACAACCTTACATTCTCCACACAAATATTTATACCCAAGGTGTTGGAAATAGGGAGCAACAGTTTTATCCTTGGTTTGATCCAACTGCTGATTTTCACAACTACACCATTCATTGGAACCCTAATGCTGTCGTATGGTATGTTGATGGTATTCCAATTAGGGTATTTAGAAATTATCAATTCAAAGGAATTCCATATCCAAACCAACAAGGAATGAGAATATACTCTAGCCTTTGGAATGCAGATGAATGGGCAACAAGAGGTGGACGTGACAAAATTGATTGGACAAATGCACCATTTATTGCAACATATCGTAAGTTTAGGCCAAGAGCTTGTTATTGGAATGGACCATTGAGTATTGTTCAATGTGCTATTCCTACTAAATCCAATTGGTGGAATTCTCCTTTATACAGTAAATTGAGTGCTCCTAAAGTGGATCAAATGAACTCAATTAGGAGCAAATACATGATTTATGACTATTGCAAAGATACTACACGATTCAAGGGAGTTATGCCTATTGAGTGTTCATTGCCACAATACTAG

## >Amplification failed

# >NtXTH39 (SGN database sequence)

ATGGCCAAATTTGTAGCTTTTAATTCCTTGGTTTTGATCATTGCAACAATTGCATTTCATTGTGCTATAGTCAATGGAAAGATCTCAAGTAGCATGTATGTCAATTGGGGTGCTCATCATTGTCAAATGCTAGGGGAAGATCTTCAACTTGTCCTTGATAAATCTGCAGGTTCTGGTGCGCAATCAAAAAGAACATTTCTTTTTGGTAGCTTTGAAATGCTTATCAAGTTGGTACCTAACAACTCTGCTGGAACTGTTACAACATACTATTTATCTTCTACTGGTACCAAGCAT***GATGAAATCGACTTTGAGTTTTTAGGA***AATGTATCGGGACAACCTTACATTCTCCACACAAATATTTATACCCAAGGTGTTGGAAATAGGGAGCAACAATTCTATCCTTGGTTTGATCCAACTGCTGATTTTCACAACTACACCATTCATTGGAACCCCAATGCTGTAGTATGGTATGTAGATAGTATTCCAATTAGGGTATTTAGAAATTATCAACTCAAAGGAATTCCATTTCCAAACCAACAAGGAATGAGAATCTACTCTAGTCTTTGGAATGCTGATGAATGGGCAACAAGAGGTGGCCGTGACAAAATTGATTGGACAAATGCACCATTTATTGCAAAATATCGTAAGTTTAGGCCAAGAGCTTGTTATTGGAATGGACCATTAAGTATTGTCCAATGTGCAATTCCAACAAAATCCAATTGGTGGAATTCTCCTTTATACAGTAAATTGAGTGCTCCTAAAGTGGACCAAATGAACTCAATTAGGAGCAAATACATGATTTATGACTATTGCAAAGATACTACACGATTCAAGGGAGTTACGCCTACTGAATGTTCATTGCCACAAAACTAG

## >Amplification failed

# >NtXTH40 (SGN database sequence)

ATGGCCAAATTCATAACTTTTTCCTTGGTTTTGATCATTGCAACATTTGCATTTCGTTGTACTCTAGTCAATGGAAAGATCTCAAGTAGCATGTATATCAATTGGGGTGCTCATCATTGTAAAATGCAAGGGGATGATCTTCAACTTGTCCTTGATAAATCTGCAGGTTCTGGTGCGCAATCAAAAAGAACATTTCTCTTTGGTAGCTTTGAAATGCTTATCAAGTTGGTACCTAACAACTCCGCTGGAACTGTTACAACATACTATCTATCTTCTACTGGCACCAAACAT***GATGAAATCGACTTCGAGTTTTTAGGA***AATGTATCAGGACAACCTTACATTATCCACACAAATATTTACACCCAAGGTGTTGGAAACAAGGAGCAACAATTCTATCCTTGGTTTGATCCAACTGCAGATTTTCACAACTACACCATTCACTGGAATCTCAATGCTGTCGTATGGTACGTAGATGGTATTCCAATTAGGGTATTTAGAAATTATGAGCTCAAAGGAATTCCATTCCCAAACCAACAAGGAATGAGAATCTACTCTAGCCTTTGGAATGCTGATGAATGGGCAACAAGAGGTGGCCGTGATAAAATTGATTGGACAAATGCACCATTTATTGCAACATATCGTAACTTTAGGCCAAGAGCTTGTTATTGGAATGGACCATTGAGTATTGGTCAATGTGCAATTCCCACAAAATCCAATTGGTGGAATTCACCTTTATACAATAAATTGAGTGCTCCTAAAGTGGATCAAATGAACTCAATTAGAAGCAAATACATGATTTATGACTATTGCAAAGATACTAAACGATTCAAGGGAGTTACGCCTACTGAATGTTCATTGCCACAAAACTAG

## >NtXTH40 amplification (same with SGN database sequence)

TCTAGATGCATGCTCGAGCGGCCGCCAGTGTGATGGATATCTGCAGAATTGCCCTTGTAATTGAAAGGGTCTTAATTTTCAGCTATATAAGAGCCTCCACAGCCTAAGAGCAAAATGTGTCATTTCCAAGTGACTCACAAAGAACAAAATTGTGTGGCAAATTAAAG**ATG**GCCAAATTCATAACTTTTTCCTTGGTTTTGATCATTGCAACATTTGCATTTCGTTGTACTCTAGTCAATGGAAAGATCTCAAGTAGCATGTATATCAATTGGGGTGCTCATCATTGTAAAATGCAAGGGGATGATCTTCAACTTGTCCTTGATAAATCTGCAGGTTCTGGTGCGCAATCAAAAAGAACATTTCTCTTTGGTAGCTTTGAAATGCTTATCAAGTTGGTACCTAACAACTCCGCTGGAACTGTTACAACATACTATCTATCTTCTACTGGCACCAAACATGATGAAATCGACTTCGAGTTTTTAGGAAATGTATCAGGACAACCTTACATTATCCACACAAATATTTACACCCAAGGTGTTGGAAACAAGGAGCAACAATTCTATCCTTGGTTTGATCCAACTGCAGATTTTCACAACTACACCATTCACTGGAATCTCAATGCTGTCGTATGGTACGTAGATGGTATTCCAATTAGGGTATTTAGAAATTATGAGCTCAAAGGAATTCCATTCCCAAACCAACAAGGAATGAGAATCTACTCTAGCCTTTGGAATGCTGATGAATGGGCAACAAGAGGTGGCCGTGATAAAATTGATTGGACAAATGCACCATTTATTGCAACATATCGTAACTTTAGGCCAAGAGCTTGTTATTGGAATGGACCATTGAGTATTGGTCAATGTGCAATTCCCACAAAATCCAATTGGTGGAATTCACCTTTATACAATAAATTGAGTGCTCCTAAAGTGGATCAAATGAACTCAATTAGAAGCAAATACATGATTTATGACTATTGCAAAGATACTAAACGATTCAAGGGAGTTACGCCTACTGAATGTTCATTGCCACAAAACTAAGGCGTCAAAATTAGAACAAGGGCAATTCCAGCACACTGGCGGCCGTTACTAGTgGATCCGAG

# >NtXTH41 (SGN database sequence)

ATGTTCAAAATTATGGCCAGCTCTCGACTTCTTTCTTTGGCTAATTTGTTCATTTTGGCAATTGCATTTCATTTGGTTTCAGTCAATGGTATGTTCTCAGATAACATGTATATTGGCTGGGGTGCCCATCATTCTTGGATGCAAGGAAATGATCTTCAGCTTGTTCTTGATCAATCCTCAGGTTCAGGTGTACAATCAAAAGGGGCATTTCTTTTTGGAAGCATACAAATGCAAATCAAATTGGTGCCTGGAAACTCTGCTGGAACAGTTACTGCATACTATTTATCCTCTACTGGTGACAAACAC***GACGAGATCGACTTCGAGTTTTTAGGG***AATGTATCAGGGCATCCATATATTATACACACAAATATTTTTACTCAAGGTGCAGGAGGCAGGGAACAACAATTCTATCCATGGTTTGATCCAACTGCTGATTATCATAACTACACCATTCATTGGAACCCCAGTGCAGTTGTATGGTACGTTGACGATATACCAATCAGAGTATACAAGAATTATCAAAGCCAGGGAATTCTCTATCCGAACGCACAAGGAATGGGGGTTTACTCTAGCCTTTGGAACGCCGATAACTGGGCAACTAGAGGCGGCCTTGACAAGATTGACTGGACCAATGCTCCATTTATAGCCAAGTACAGAAATTTCGCGCCACGAGCTTGTCCCTGGTATGGACCAGGTAGCATTAGCCATTGTGCTGCTCCAACTCCAAATAATTGGTATACTTCTCCTGAGTATAGTCAATTGAGCTATGCTAAGCAAGGGCAAATGAATTGGGTAAGGAACAATTACATGATCTATGATTATTGTAAAGATACGACGCGATTCAATGGACAGATTCCTGGAGAATGTTTTAAGCCTCAATTCTAA

## >NtXTH41 amplification (1 SNP with SGN database sequence)

TAGATGCATGCTCGAGCGGCCGCCAGTGTGATGGATATCTGCAGAATTGCCCTTGTCATATTCCTAAGAACAAACACACACCATTAATTTGCAAAACTGAGAGAAGCAAGAAATCTATTACAGTTCC**ATG**TTCAAAATTATGGCCAGCTCTCGACTTCTTTCTTTGGCTAATTTGTTCATTTTGGCAATTGCATTTCATTTGGTTTCAGTCAATGGTATGTTCTCAGATAACATGTATATTGGCTGGGGTGCCCATCATTCTTGGATGCAAGGAAATGATCTTCAGCTTGTTCTTGATCAATCCTCAGGTTCAGGTGTACAATCAAAAGGGGCATTTCTTTTTGGAAGCATACAAATGCAAATCAAATTGGTGCCTGGAAACTCTGCTGGAACAGTTACTGCATACTATTTATCCTCTACTGGTGACAAACACGACGAGATCGACTTCGAGTTTTTAGGGAATGTATCAGGGCATCCATATATTATACACACAAATATTTTTACTCAAGGTGCAGGAGGCAGGGAACAACAATTCTATCCATGGTTTGATCCAACTGCTGATTATCATAACTACACCATTCATTGGAACCCCAGTGCAGTTGTATGGTACGTTGACGATATACCAATCAGAGTATACAAGAATTATCAAAGCCAGGGAATTCTCTATCCGAACGCACAAGGAATGGGGGTTTACTCTAGCCTTTGGAACGCCGATAACTGGGCAACTAGAGGCGGCCTTGACAAGATTGACTGGACCAATGCTCCATTTATAGCCAAGTACAGAAATTTCGCGCCACGAGCCTGTCCCTGGTATGGACCAGGTAGCATTAGCCATTGTGCTGCTCCAACTCCAAATAATTGGTATACTTCTCCTGAGTATAGTCAATTGAGCTATGCTAAGCAAGGGCAAATGAATTGGGTAAGGAACAATTACATGATCTATGATTATTGTAAAGATACGACGCGATTCAATGGACAGATTCCTGGAGAATGTTTTAAGCCTCAATTC**TAA**CAAGTGAATTGAGTCGATTGGAATATCCACTTATGGGAATATACTGAGTTTGTTATTGTGTTGTTGAATTGAGGCCATTGTTATACTTTGTAAGGGCAATTCCAGCACACTGGCGGCCGTTACTAGTGGATCCGAGCTCG

# >NtXTH42 (SGN database sequence)

ATGTTCAAAATTATGGCCAGCTCTCGACTTCTTTCTTTGTCTAATTTGTTCATTTTGGCAATTGCATTTCATTTGGTTTCAGTCAATGGAATGTTCTCAGATAACATGTATATTAACTGGGGTGCCCATCATTCTTGGATGCAAGGAAATGATCTTCAGCTTGTCCTTGATCAATCCGCAGGTTCGGGTGTACAATCAAAAGGAGCATTTCTTTTTGGAAGCATAGAAATGCAAATAAAATTAGTACCTGGAAATTCTGCTGGAACAGTCACAGCATACTATTTGTCATCTACTGGTGACAAGCAC***GACGAGATCGACTTCGAGTTTTTGGGA***AATGTATCAGGGCAACCATATATTATACACACAAATATTTTTACTCAAGGTGCAGGAGGCAGGGAACAACAATTCTATCCGTGGTTTGATCCAACTGCTGATTACCATAACTATACCATTCATTGGAACCCCAGTGCAGTTGTATGGTACGTTGACGGTATACCAATCAGAGTATACAAGAATTATCAGAGCCAGGGAATTCTCTATCCGAACGCACAAGGAATGAAGGTTTACTCTAGCCTTTGGAACGCCGATAACTGGGCAACCAGAGGCGGCCTTGACAAGATTGACTGGACCAATGCTCCATTTATAGCCAAGTACAGAAATTTCGCGCCGCGAGCTTGTCCCTGGTATGGACCAGGTAGCATTCGCCAATGTGCTGCTCCAACTCCAAATAATTGGTATACTTCTTATGAGTATAGTCAATTGAGCTATGCTAAGCAAGGGCAAATGAATTGGGTAAGGAACAATTACATGATCTATGATTATTGTAAAGATAAGACGCGATTCAATGGACAGATTCCAGGAGAATGTTTTAAGCCTCAAATCTAA

## >NtXTH42 amplification (same with SGN database sequence)

AGAAGCAAGAAATCTATTACAGTTCC**ATG**TTCAAAATTATGGCCAGCTCTCGACTTCTTTCTTTGTCTAATTTGTTCATTTTGGCAATTGCATTTCATTTGGTTTCAGTCAATGGAATGTTCTCAGATAACATGTATATTAACTGGGGTGCCCATCATTCTTGGATGCAAGGAAATGATCTTCAGCTTGTCCTTGATCAATCCGCAGGTTCGGGTGTACAATCAAAAGGAGCATTTCTTTTTGGAAGCATAGAAATGCAAATAAAATTAGTACCTGGAAATTCTGCTGGAACAGTCACAGCATACTATTTGTCATCTACTGGTGACAAGCACGACGAGATCGACTTCGAGTTTTTGGGAAATGTATCAGGGCAACCATATATTATACACACAAATATTTTTACTCAAGGTGCAGGAGGCAGGGAACAACAATTCTATCCGTGGTTTGATCCAACTGCTGATTACCATAACTATACCATTCATTGGAACCCCAGTGCAGTTGTATGGTACGTTGACGGTATACCAATCAGAGTATACAAGAATTATCAGAGCCAGGGAATTCTCTATCCGAACGCACAAGGAATGAAGGTTTACTCTAGCCTTTGGAACGCCGATAACTGGGCAACCAGAGGCGGCCTTGACAAGATTGACTGGACCAATGCTCCATTTATAGCCAAGTACAGAAATTTCGCGCCGCGAGCTTGTCCCTGGTATGGACCAGGTAGCATTCGCCAATGTGCTGCTCCAACTCCAAATAATTGGTATACTTCTTATGAGTATAGTCAATTGAGCTATGCTAAGCAAGGGCAAATGAATTGGGTAAGGAACAATTACATGATCTATGATTATTGTAAAGATAAGACGCGATTCAATGGACAGATTCCAGGAGAATGTTTTAAGCCTCAAATC**TAA**

# >NtXTH43(SGN database sequence)

ATGGCAATCTTTTTTCTCCATTTTCTTCTCTTGCTCATTGTTGTCCCTTCTACAAATGCTGGTTATTGGCCACCTTCTCCTGGCTATTATCCAAGTTCCAAGTTTAGGTCTATGAGCTTTTATCAAGGATTTAGAAACCTTTGGGGCCCTAATCATCAAAATGTAGATAATAATGGCATTAATATTTGGCTTGATAGAAATTCAGGAAGTGGATTCAAGTCAATTAAACCATTTCGATCAGGGTATTTTGGTGCTTCCATTAAACTCCAACCTGGTTATACTGCTGGTGTTATTACAGCTTTTTACCTTTCAAATAATGAAGCTCATCCAGGGTACCAT***GATGAAGTGGACATAGAATTTCTTGGA***ACAACATTTGGGAAGCCTTATACATTGCAAACCAATGTTTATATTAGAGGAAGCGGAGATGGGAAAATTGTAGGAAGAGAAATGAAGTTTCATTTGTGGTTTGATCCAACAAAGGAATTTCATCACTATGCTATTTTGTGGAGTCCTAGAGAGATCATATTTCTTGTGGATGATGTGCCAATAAGGAGGTATGCTAGGAAGAGTATTGCAACATTTCCACTAAGGCCAATGTGGTTATATGGATCAATATGGGATGCATCTTCTTGGGCAACTGAGGATGGAAAATACAAAGCCGATTATAGGTACCAACCATTCTACGGGAAATTCACGAACTTTAAGGCAAGCGGTTGCACCGCCTATTCATCGCGATGGTGCCACCCCGTGTCCGCTTCACCATCCAGGTCCGGAGGCCTTACCAGGCAACAACGTCAAGCCATGAATTGGGTTCATAGTCACTACTTGGCTTATGACTATTGTCGAGACTCCAAAAGAGACCATTCCCTAACACCGGAATGCTGGCGTTAA

## >NtXTH43 amplification (same with SGN database sequence)

GAGCTCGGATCcACTAGTAACGGCCGCCAGTGTGCTGGAATTGCCCTTTCTATATACACCTTCCTTTCAACACTCTCTTGTCTTTAACCTGCATTCATATTCATATATATATGTATAATAGCTAAGCTCCATTTACTAAATCTTCAATTTTTCTCTATCATTTTCCTTCTTCAATTCAATA**ATG**GCAATCTTTTTTCTCCATTTTCTTCTCTTGCTCATTGTTGTCCCTTCTACAAATGCTGGTTATTGGCCACCTTCTCCTGGCTATTATCCAAGTTCCAAGTTTAGGTCTATGAGCTTTTATCAAGGATTTAGAAACCTTTGGGGCCCTAATCATCAAAATGTAGATAATAATGGCATTAATATTTGGCTTGATAGAAATTCAGGAAGTGGATTCAAGTCAATTAAACCATTTCGATCAGGGTATTTTGGTGCTTCCATTAAACTCCAACCTGGTTATACTGCTGGTGTTATTACAGCTTTTTACCTTTCAAATAATGAAGCTCATCCAGGGTACCATGATGAAGTGGACATAGAATTTCTTGGAACAACATTTGGGAAGCCTTATACATTGCAAACCAATGTTTATATTAGAGGAAGCGGAGATGGGAAAATTGTAGGAAGAGAAATGAAGTTTCATTTGTGGTTTGATCCAACAAAGGAATTTCATCACTATGCTATTTTGTGGAGTCCTAGAGAGATCATATTTCTTGTGGATGATGTGCCAATAAGGAGGTATGCTAGGAAGAGTATTGCAACATTTCCACTAAGGCCAATGTGGTTATATGGATCAATATGGGATGCATCTTCTTGGGCAACTGAGGATGGAAAATACAAAGCCGATTATAGGTACCAACCATTCTACGGGAAATTCACGAACTTTAAGGCAAGCGGTTGCACCGCCTATTCATCGCGATGGTGCCACCCCGTGTCCGCTTCACCATCCAGGTCCGGAGGCCTTACCAGGCAACAACGTCAAGCCATGAATTGGGTTCATAGTCACTACTTGGCTTATGACTATTGTCGAGACTCCAAAAGAGACCATTCCCTAACACCGGAATGCTGGCGT**TAA**GCGAACTGCAAAAGCTGTGAGATGTAAAAGTACATGGGTGGAAGTGTCAATCTTTCATAGAAGGGCAATTCTGCAGATATCCATCACACTGGCGGCCGCTCGAGCATGCATCTAGA

# >NtXTH44 (SGN database sequence)

ATGTCAATCTTTTTCCTCCCTTTTCTTCTCTTCCTCATTGTTCTCCCTTCTACAAATGCTGGTTATTGGCCACCTTCTCCTGGCTATTATCCAAGTTCCAAGTTTAAGTCCATGAGTTTCTATCAAGGTTTTAAAAACCTTTGGGGTCCTAATCATCAAAATGTAGATAATAATGGCATTAATATTTGGCTTGATAGAAATTCAGGAAGTGGATTCAAGTCAATTAAACCATTTCGATCCGGGTATTTTGGTGCTTCTATTAAACTTCAACCTGGTTACACTGCTGGTGTTATTACAGCTTTCTACCTTTCAAATAATGAAGCCCATCCAGGGTACCAT***GATGAAGTGGACATAGAATTTCTTGGA***ACAACATTTGGGAAGCCTTACACATTGCAAACCAATGTTTATATTAGAGGAAGTGGAGATGGTAAAATTATAGGAAGAGAAATGAAGTTTCATTTGTGGTTTGATCCAACAAAGGATTTTCATCACTATGCTATTTTGTGGAGTCCTAGAGAGATCATATTTCTTGTGGATGATGTGCCAATAAGGAGGTATGCTAGGAAGAGTATTGCAACATTTCCACTAAGGCCAATGTGGTTATATGGATCAATATGGGATGCATCTTCTTGGGCAACTGAGGATGGAAAATACAAAGCCGATTATAGGTACCAACCATTCTACGGGAAATTCACGAATTTTAAGGCAAGCGGTTGCACCGCCTATTCATCGCGATGGTGCCACCCTGTGTCCGCTTCGCCATCCCGGTCCGGAGGCCTTACTAGGCAACAACGTCAAGCCATGAATTGGGTTCATAGTCACTACTTGGCCTATGACTATTGTCGAGACTCCAAAAGAGACCATTCCCTTACACCGGAATGTTGGCGTTAA

## >NtXTH44 amplification (1 SNP with SGN database sequence)

TAGTCTTCAATTTTCTTCACATTTTCTCTATCATTTTCCTTCTTCAATTCAATA**ATG**TCAATCTTTTTCCTCCCTTTTCTTCTCTTCCTCATTGTTCTCCCTTCTACAAATGCTGGTTATTGGCCACCTTCTCCTGGCTATTATCCAAGTTCCAAGTTTAAGTCCATGAGTTTCTATCAAGGTTTTAAAAACCTTTGGGGTCCTAATCATCAAAATGTAGATAATAATGGCATTAATATTTGGCTTGATAGAAATTCAGGAAGTGGATTCAAGTCAATTAAACCATTTCGATCCGGGTATTTTGGTGCTTCTATTAAACTTCAACCTGGTTACACTGCTGGTGTTATTACAGCTTTCTACCTTTCAAATAATGAAGCCCATCCAGGGTACCATGATGAAGTGGACATAGAATTTCTTGGAACAACATTTGGGAAGCCTTACACATTGCAAACCAATGTTTATATTAGAGGAAGTGGAGATGGTAAAATTATAGGAAGAGAAATGAAGTTTCATTTGTGGTTTGATCCAACAAAGGATTTTCATCACTATGCTATTTTGTGGAGTCCTAGAGAGATCATATTTCTTGTGGATGATGTGCCAATAAGGAGGTATGCTAGGAAGAGTATTGCAACATTTCCACTAAGGCCAATGTGGTTATATGGATCAATATGGGATGCATCTTCTTGGGCAACTGAGGATGGAAAATACAAAGCCGATTATAGGTACCAACCATTCTACGGAAAATTCACGAATTTTAAGGCAAGCGGTTGCACCGCCTATTCATCGCGATGGTGCCACCCTGTGTCCGCTTCGCCATCCCGGTCCGGAGGCCTTACTAGGCAACAACGTCAAGCCATGAATTGGGTTCATAGTCACTACTTGGCCTATGACTATTGTCGAGACTCCAAAAGAGACCATTCCCTTACACCGGAATGTTGGCGT**TAA**G

# >NtXTH45(SGN database sequence)

ATGGCTAATTTATTCCTTCTTTCTTTACTTCTCATTTTCTTGTTCAATTCAAGCAATGCTCAGGGTCCCCTTTCTCCTGGCTACTATCCTAGTTCTAAGGTTCAATCGTTAGGGTTTAACCAGGGTTTTAGAAACCTTTGGGGTCCTCAACATCAATCTTTGGACCAAAGTGCTTTAACAATATGGCTTGATAAAACCTCAGGGGGAAGTGGCTTTAAATCTCTGGAAAATTATCGTTCCGGTTATTTTGGCACTTCTGTGAAGCTACAACCTGGTTACACTGCTGGAATTATTACTTCTTTCTATCTTTCAAACAATCAAGATTATCCAGGGAACCATG***ATGAAATTGATATTGAGTTTCTTGGAA***CAACGCCAAATAAGCCTTATACTTTACAAACAAATGTATACATCAGAGGAAGTGGAGATGGAAATATTATTGGGAGAGAAATGAAATTTCACCTTTGGTTTGATCCAACTAAAGCTTACCACAATTATGCTATCCTTTGGGATCCCAATGAGATCATATTTTTTGTCGACGATGTTCCAATCAGAAGATACCCTAGGAAAAATGATGCTACATTTCCACAAAGACCTATGTATGTGTATGGTTCAATTTGGGATGCTTCATCTTGGGCAACAGAGGAAGGAAGAATTAAAGCCGATTATCGGTACCAACCATTCGTCGGAAAATATAACAATTTTAAAATTGCTGGTTGTACAGCTAATGAGAACCCCTGGTGCGGACGCTCGCCCTCCAGCTCTCCGTCTAGAGCTGGTGGGCTGAGCCGCCAACAAATAGCGGCCATGCTATGGGTGCAGAGGAACTATAAGGTGTATGATTATTGTCGGGACCCTAGGAGAGACCATACTCACACTCCTGAGTGTTAG

## >NtXTH45 amplification (same with SGN database sequence)

TCATTTTATCCACATTAATGATTATAtTTCTTCCCTTTCTAATT**ATG**GCTAATTTATTCCTTCTTTCTTTACTTCTCATTTTCTTGTTCAATTCAAGCAATGCTCAGGGTCCCCTTTCTCCTGGCTACTATCCTAGTTCTAAGGTTCAATCGTTAGGGTTTAACCAGGGTTTTAGAAACCTTTGGGGTCCTCAACATCAATCTTTGGACCAAAGTGCTTTAACAATATGGCTTGATAAAACCTCAGGGGGAAGTGGCTTTAAATCTCTGGAAAATTATCGTTCCGGTTATTTTGGCACTTCTGTGAAGCTACAACCTGGTTACACTGCTGGAATTATTACTTCTTTCTATCTTTCAAACAATCAAGATTATCCAGGGAACCATGATGAAATTGATATTGAGTTTCTTGGAACAACGCCAAATAAGCCTTATACTTTACAAACAAATGTATACATCAGAGGAAGTGGAGATGGAAATATTATTGGGAGAGAAATGAAATTTCACCTTTGGTTTGATCCAACTAAAGCTTACCACAATTATGCTATCCTTTGGGATCCCAATGAGATCATATTTTTTGTCGACGATGTTCCAATCAGAAGATACCCTAGGAAAAATGATGCTACATTTCCACAAAGACCTATGTATGTGTATGGTTCAATTTGGGATGCTTCATCTTGGGCAACAGAGGAAGGAAGAATTAAAGCCGATTATCGGTACCAACCATTCGTCGGAAAATATAACAATTTTAAAATTGCTGGTTGTACAGCTAATGAGAACCCCTGGTGCGGACGCTCGCCCTCCAGCTCTCCGTCTAGAGCTGGTGGGCTGAGCCGCCAACAAATAGCGGCCATGCTATGGGTGCAGAGGAACTATAAGGTGTATGATTATTGTCGGGACCCTAGGAGAGACCATACTCACACTCCTGAGTGTTACGATTCGTAAAAGG

# >NtXTH46 (SGN database sequence)

ATGGCTTTATTCCTTCTTTCTTTGCTTCTCCTTTTCTTGTTCAATTCAAGCAATGCTCAGGGTCCCCCTTCTCCAGGCTACTATCCTAGTTCTAAGGTTCAATCTTTAGGGTTTAGCCAGTGTTTTAGAAACCTTTGGGGTCCTCAACATCAATCTTTGGACCAAAGTGCCTTAACTATATGGCTTGATAAAACCACAGGGGGAAGTGGCTTTAAATCTCTAAAAAATTATCGTTCCGGTTATTTTGGCACTTCTGTGAAGCTACAGCCTGGTTACACTGCTGGAATTATTACTTCTTTCTATCTTTCAAACAATCAAGATTATCCAGGGAACCAT***GATGAAATTGATATTGAGTTTCTTGGA***ACAACGCCAAATAAGCCTTATACTTTACAAACAAATGTATACATCAGAGGAAGTGGAGATGGAAATATTATTGGGAGAGAAATGAAATTTCACCTTTGGTTTGACCCAACTCAAGCTTACCACAATTATGCTATCCTTTGGAATCCCAATGAGATCATATTTTTTGTCGACGATGTTCCAATCAGAAGATACCCTAGGAAAAATGATGCTACATTTCCACAAAGACCTATGTATGTGTATGGTTCAATTTGGGATGCTTCATCTTGGGCAACAGAGGAAGGAAGAATTAAAGCCGATTATCGGTACCAACCATTCATCGGAAAATATAACAATTTTAAAATTGCTGGTTGCACAGCTAACGAGAACCCCTGGTGCGGACGCTCGCCCTCCAGCTCTTCGTCTAGAGCTGGTGGGCTGAGCCGCCAGCAGATGGCGGCCATGCTATGGGTGCAGAGGAACTATAAGGTGTATGATTATTGTCGGGACCCCAGGAGAGACCATACTCACACTCCTGAGTGTTAG

## >NtXTH46 amplification (same with SGN database sequence)

GCCTCTAAAGCACTCATTTTTATCCACATTAATTGATTATATTTCTTCCCTTTTATAATT**ATG**GCTTTATTCCTTCTTTCTTTGCTTCTCCTTTTCTTGTTCAATTCAAGCAATGCTCAGGGTCCCCCTTCTCCAGGCTACTATCCTAGTTCTAAGGTTCAATCTTTAGGGTTTAGCCAGTGTTTTAGAAACCTTTGGGGTCCTCAACATCAATCTTTGGACCAAAGTGCCTTAACTATATGGCTTGATAAAACCACAGGGGGAAGTGGCTTTAAATCTCTAAAAAATTATCGTTCCGGTTATTTTGGCACTTCTGTGAAGCTACAGCCTGGTTACACTGCTGGAATTATTACTTCTTTCTATCTTTCAAACAATCAAGATTATCCAGGGAACCATGATGAAATTGATATTGAGTTTCTTGGAACAACGCCAAATAAGCCTTATACTTTACAAACAAATGTATACATCAGAGGAAGTGGAGATGGAAATATTATTGGGAGAGAAATGAAATTTCACCTTTGGTTTGACCCAACTCAAGCTTACCACAATTATGCTATCCTTTGGAATCCCAATGAGATCATATTTTTTGTCGACGATGTTCCAATCAGAAGATACCCTAGGAAAAATGATGCTACATTTCCACAAAGACCTATGTATGTGTATGGTTCAATTTGGGATGCTTCATCTTGGGCAACAGAGGAAGGAAGAATTAAAGCCGATTATCGGTACCAACCATTCATCGGAAAATATAACAATTTTAAAATTGCTGGTTGCACAGCTAACGAGAACCCCTGGTGCGGACGCTCGCCCTCCAGCTCTTCGTCTAGAGCTGGTGGGCTGAGCCGCCAGCAGATGGCGGCCATGCTATGGGTGCAGAGGAACTATAAGGTGTATGATTATTGTCGGGACCCCAGGAGAGACCATACTCACACTCCTGAGTGT**TAG**

# >NtXTH47 (SGN database sequence)

ATGGATTTCTTTCATCATAATAAAACCTTCCTATTATCACAGTTCTTGATTTTCTGCATGATAGTTGTCGTTTCATGTCGAGGTCCAGTCTATAAGCCTCCAGAAGTAGAGAAATTAACTGATCATTTCAGCCGATTATCGGTTAATCAAGGTTATAATGTATTCTTTGGAGGTGCTAATGTTCGTATGACAAACAATGGGTCCAGTGCTGATCTTATCTTAGATAAATCTTCAGGTTCTGGACTGATCTCTAAGGAGAAATACTACTATGGTTTCTTCAATGCTGCTCTAAAACTGCCTGCTCATTTTACATCCGGAGTTGTAATTGCCTTTTATATGTCTAATTCAGATGTGTTCCCACACAACCAT***GATGAAATTGACTTTGAGTTGCTTGGG***CATGATAAGAGAAGAGATTGGGTTCTGCAGACTAATCTATATGGAAATGGAAGTGTTCACACAGGGAGAGAAGAGAAGTTTTACCTCTGGTTTGATCCAACACTGGATTTTCATGACTACACCATCCTCTGGAATAATCATCACATAGTATTTCTTGTGGACAATGTGCCAATAAGAGAGGTAGTTCATAACACAGCTATATCTTCTGTTTACCCATCAAAGCCAATGTCTGTTATAGCAACAATATGGGATGGATCAGAATGGGCAACTCATGGAGGAAAATACCCTGTAAACTACCAATATGCACCATTTGTAACATCAATGAAAGAAGTAGAATTAGAAGGATGTGTAAGACAACAAAATACTTCAGCAACTTCTACATGTTTTAGGAGAAGTACTTCAAGTTTGGATCCTGTTGATGGGGAAGAATTTATGAAATTATCACAACAGCAGATGACAGGGCTGGATTGGGTAAGGAGAAAGCACATGTTCTACTCATATTGTCAAGATACTAATAGATACAAAGTTCTACCACCAGAGTGCACTTCTAATTAA

## >NtXTH47 amplification (same with SGN database sequence)

T**ATG**GATTTCTTTCATCATAATAAAACCTTCCTATTATCACAGTTCTTGATTTTCTGCATGATAGTTGTCGTTTCATGTCGAGGTCCAGTCTATAAGCCTCCAGAAGTAGAGAAATTAACTGATCATTTCAGCCGATTATCGGTTAATCAAGGTTATAATGTATTCTTTGGAGGTGCTAATGTTCGTATGACAAACAATGGGTCCAGTGCTGATCTTATCTTAGATAAATCTTCAGGTTCTGGACTGATCTCTAAGGAGAAATACTACTATGGTTTCTTCAATGCTGCTCTAAAACTGCCTGCTCATTTTACATCCGGAGTTGTAATTGCCTTTTATATGTCTAATTCAGATGTGTTCCCACACAACCATGATGAAATTGACTTTGAGTTGCTTGGGCATGATAAGAGAAGAGATTGGGTTCTGCAGACTAATCTATATGGAAATGGAAGTGTTCACACAGGGAGAGAAGAGAAGTTTTACCTCTGGTTTGATCCAACACTGGATTTTCATGACTACACCATCCTCTGGAATAATCATCACATAGTATTTCTTGTGGACAATGTGCCAATAAGAGAGGTAGTTCATAACACAGCTATATCTTCTGTTTACCCATCAAAGCCAATGTCTGTTATAGCAACAATATGGGATGGATCAGAATGGGCAACTCATGGAGGAAAATACCCTGTAAACTACCAATATGCACCATTTGTAACATCAATGAAAGAAGTAGAATTAGAAGGATGTGTAAGACAACAAAATACTTCAGCAACTTCTACATGTTTTAGGAGAAGTACTTCAAGTTTGGATCCTGTTGATGGGGAAGAATTTATGAAATTATCACAACAGCAGATGACAGGGCTGGATTGGGTAAGGAGAAAGCACATGTTCTACTCATATTGTCAAGATACTAATAGATACAAAGTTCTACCACCAGAGTGCACTTCTAAT**TAA**TTTATTTGTAACTAATAAAT

# >NtXTH48 (SGN database sequence)

ATGGAATTCTATCATCAGCACAAAACATGCTTATTTTCAGGATTCTTGATTTTCTGCATGATAGCTGTGGCTTCATCTCTAGGTCCAATCTATACTCCTCCAGAGGCCGAGCGGTTAACTGATCGTTTCAGTAGATTATCCGTTAATCAGGGATATAATGTGTTTTTCGGAGGTGCTAATGTTCGTCTAACCAACAATGGGTCCAATGCTGATCTTATCTTAGATAAATCTTCAGGTTCAGGACTAGTCTCAAGAGACAAATACTACTATGGTTTCTTCAATGCTGCACTAAAGCTGCCTGCAAATTTTACATCAGGAGTGGTAGTTGCTTTTTATCTTTCTAATCAAAATATTTTCCCACACAACCAT***GATGAACTAGATTTTGAACTGCTTGGG***TATGATAAGAGAAGGGATTGGGTTCTACAAACCAATATTTATGGAAATGGAAGTGTCAGCACAGGGAGGGAAGAGAAGTTCTACCTCTGGTTTGATCCAACACAAGATTTCCATGACTACAGTATTCTCTGGAACAATCATCACATTCTATTTCTAGTGGACAATGTGCCAGTAAGAGAGGTTGTCAATAATACTACAATCTCTTCTGTTTACCCATCTAAGCCAATGTCTATTTATGCAACAATATGGGATGGATCACAATGGGCAACTCGTGGAGGGAAATACCCAGTAAATTATACTTACGCCCCGTTTGTAACATCAATAAAAGGAGTAGAGTTAGAAGGGTGTGTAAGCGAGCAAAACGCATCAGCAGCTAGTGCATGTGCTAGGAGAAGCACATCAAGTTTGGATCCTGTTGATGGAGAAGAGTTTGTCAAGCTGTCACAGCAGCAAATGACGGGGTTGGACTGGGCAAGGAGGAAGCATATGTTTTACTCGTATTGCCAAGATACTAGGAGATACAAAGTCCTACCACCAGAGTGCACTGCCACATAA

## >NtXTH48 amplification (same with SGN database sequence)

CaTATGCATAACATAACAAGAAATAA**ATG**GAATTCTATCATCAGCACAAAACATGCTTATTTTCAGGATTCTTGATTTTCTGCATGATAGCTGTGGCTTCATCTCTAGGTCCAATCTATACTCCTCCAGAGGCCGAGCGGTTAACTGATCGTTTCAGTAGATTATCCGTTAATCAGGGATATAATGTGTTTTTCGGAGGTGCTAATGTTCGTCTAACCAACAATGGGTCCAATGCTGATCTTATCTTAGATAAATCTTCAGGTTCAGGACTAGTCTCAAGAGACAAATACTACTATGGTTTCTTCAATGCTGCACTAAAGCTGCCTGCAAATTTTACATCAGGAGTGGTAGTTGCTTTTTATCTTTCTAATCAAAATATTTTCCCACACAACCATGATGAACTAGATTTTGAACTGCTTGGGTATGATAAGAGAAGGGATTGGGTTCTACAAACCAATATTTATGGAAATGGAAGTGTCAGCACAGGGAGGGAAGAGAAGTTCTACCTCTGGTTTGATCCAACACAAGATTTCCATGACTACAGTATTCTCTGGAACAATCATCACATTCTATTTCTAGTGGACAATGTGCCAGTAAGAGAGGTTGTCAATAATACTACAATCTCTTCTGTTTACCCATCTAAGCCAATGTCTATTTATGCAACAATATGGGATGGATCACAATGGGCAACTCGTGGAGGGAAATACCCAGTAAATTATACTTACGCCCCGTTTGTAACATCAATAAAAGGAGTAGAGTTAGAAGGGTGTGTAAGCGAGCAAAACGCATCAGCAGCTAGTGCATGTGCTAGGAGAAGCACATCAAGTTTGGATCCTGTTGATGGAGAAGAGTTTGTCAAGCTGTCACAGCAGCAAATGACGGGGTTGGACTGGGCAAGGAGGAAGCATATGTTTTACTCGTATTGCCAAGATACTAGGAGATACAAAGTCCTACCACCAGAGTGCACTGCCACA**TAA**C

# >NtXTH49 (SGN database sequence)

ATGGAATTCTTTCACCAGCACAACACACTCTTATTATCAGAGTTCTTGATTTTCTGCATGATATCTGTGGCTTCATCTCTAGGTCCAATCTATACTCCTCCAGAGGTTGAGCGGTTAACTGATCGTTTCAGTAGATTATCCGTTAATCAGGGATATAATATGTTTTTTGGAGGTGTTAATGTTCGTCTAACGAACAATGGGTCCAGTGCTGATCTTATCTTAGATAAATCTTCAGGTTCAGGACTAGTCTCAAGAGACAAATACTACTATGGTTTCTTCAATGCTGCACTAAAGCTGCCTGCAAATTTTACATCGGGAGTGGTAGTTGCTTTTTATCTTTCTAATCAAAATATTTTCCCACACGACCAT***GATGAACTAGATTTTGAATTGCTTGGG***TATGATAAGAGAAGGGATTGGGTTCTACAAACCAATAATTATGGAAATGGAAGTGTCAGCACAGGGAGGGAAGGGAAGTTCTACCTCTGGTTTGATCCAACACAAGATTTCCATGACTACACTATTCTCTGGAACAATCATCACATTCTATTTCTGGTGGACAATGTGCCAGTAAGAGAGGTTGTCCATAATACTGCAATCTCTTCTGTTTACCCATCAAAGCCGATGTCCATTTATGTGACAATATGGGATGGATCACAATGGGCAACTCGCAGAGGGAAATACCCAGTAAATTATACTTACGCCCCGTTTGTAACATCAATAAAAGGAGTAGAGTTAGAAGGGTGTGTAAGCGAGCAAAACGGATCAGCAGCTACTGCATGTGCTAGGAGAAGCACATCAAGTTTGGATCCGGTTGATGGAGAAGAATTTGTCAAGCTGTCACAGCAGCAAATGATGGGGTTGGACTGGGCAAGGAGGAAGCATATGTTTTACTCGTATTGCCAAGATACTAGGAGATACAAAGTCCTACCACCAGAGTGCACTGCCACATAA

## >NtXTH49.1 amplification (same with SGN database sequence)

CTCGGATCACTAGTAACGGCCGCCAGTGTGCTGGAATTGCCCTTTTAAGGTTTCCTCTGATAAATAGCCCTGTTTTGCCCCACATTTTCCTTGATTCATATACATAACATAACAAGAAAGAA**ATG**GAATTCTTTCACCAGCACAACACACTCTTATTATCAGAGTTCTTGATTTTCTGCATGATATCTGTGGCTTCATCTCTAGGTCCAATCTATACTCCTCCAGAGGTTGAGCGGTTAACTGATCGTTTCAGTAGATTATCCGTTAATCAGGGATATAATATGTTTTTTGGAGGTGTTAATGTTCGTCTAACGAACAATGGGTCCAGTGCTGATCTTATCTTAGATAAATCTTCAGGTTCAGGACTAGTCTCAAGAGACAAATACTACTATGGTTTCTTCAATGCTGCACTAAAGCTGCCTGCAAATTTTACATCGGGAGTGGTAGTTGCTTTTTATCTTTCTAATCAAAATATTTTCCCACACGACCATGATGAACTAGATTTTGAATTGCTTGGGTATGATAAGAGAAGGGATTGGGTTCTACAAACCAATAATTATGGAAATGGAAGTGTCAGCACAGGGAGGGAAGGGAAGTTCTACCTCTGGTTTGATCCAACACAAGATTTCCATGACTACACTATTCTCTGGAACAATCATCACATTCTATTTCTGGTGGACAATGTGCCAGTAAGAGAGGTTGTCCATAATACTGCAATCTCTTCTGTTTACCCATCAAAGCCGATGTCCATTTATGTGACAATATGGGATGGATCACAATGGGCAACTCGCAGAGGGAAATACCCAGTAAATTATACTTACGCCCCGTTTGTAACATCAATAAAAGGAGTAGAGTTAGAAGGGTGTGTAAGCGAGCAAAACGGATCAGCAGCTACTGCATGTGCTAGGAGAAGCACATCAAGTTTGGATCCGGTTGATGGAGAAGAATTTGTCAAGCTGTCACAGCAGCAAATGATGGGGTTGGACTGGGCAAGGAGGAAGCATATGTTTTACTCGTATTGCCAAGATACTAGGAGATACAAAGTCCTACCACCAGAGTGCACTGCCACA**TAA**CATAGTTCATCTCATTTTTGTCTCTAAGACTGACGTATGAAAAAGGGCAATTCTGCAGATATCCATCACACTGGCGGCCGCTCGAGCATGCATCTAGA

## >NtXTH49.2 amplification (3 fragments insertion)

GATTCaTATACATAACATAACAAGAAAGAA**ATG**GAATTCTTTCACCAGCACAACACACTCTTATTATCAGAGTTCTTGATTTTCTGCATGATATCTGTGGCTTCATCTCTAGGTCCAATCTATACTCCTCCAGAGGTTGAGCGGTTAACTGATCGTTTCAGTAGATTATCCGTTAATCAGGGATATAATATGTTTTTTGGAGGTGTTAATGTTCGTCTAACGAACAATGGGTCCAGTGCTGATCTTATCTTAGATAAATCTTCAGGT**AGACTCCCACATCATAATATGAGAAATGCTTTGCTCAAGTTTAAACTACTGAAGTAATTAACTCCTTGTCTTCTTGGTCCCTCCATAGGT**TCAGGACTAGTCTCAAGAGACAAATACTACTATGGTTTCTTCAATGCTGCACTAAAGCTGCCTGCAAATTTTACATCGGGAGTGGTAGTTGCTTTTTAT**GTAAGTTTCTTGCATATCCCACAGTATTATGAATCATAATTTCTATTTTATTTGAAGCATAATGCAAGAGCTTTTTTCCCATTTTCAG**CTTTCTAATCAAAATATTTTCCCACACGACCATGATGAACTAGATTTTGAATTGCTTGGGTATGATAAGAGAAGGGATTGGGTTCTACAAACCAATAATTATGGAAATGGAAGTGTCAGCACAGGGAGGGAAGGGAAGTTCTACCTCTGGTTTGATCCAACACAAGATTTCCATGACTACACTATTCTCTGGAACAATCATCACATTCT**GTAAGCCAGTGAAAAAAAAATTGAGTACTTTATGAGTTCCATTTACCTTGGTTCTAAATATAACAATTGTCTGTGAACTTCTGCAG**ATTTCTGGTGGACAATGTGCCAGTAAGAGAGGTTGTCCATAATACTGCAATCTCTTCTGTTTACCCATCAAAGCCGATGTCCATTTATGTGACAATATGGGATGGATCACAATGGGCAACTCGCAGAGGGAAATACCCAGTAAATTATACTTACGCCCCGTTTGTAACATCAATAAAAGGAGTAGAGTTAGAAGGGTGTGTAAGCGAGCAAAACGGATCAGCAGCTACTGCATGTGCTAGGAGAAGCACATCAAGTTTGGATCCGGTTGATGGAGAAGAATTTGTCAAGCTGTCACAGCAGCAAATGATGGGGTTGGACTGGGCAAGGAGGAAGCATATGTTTTACTCGTATTGCCAAGATACTAGGAGATACAAAGTCCTACCACCAGAGTGCACTGCCACA**TAA**

# >NtXTH50(SGN database sequence)

ATGGATTATCGAGTTCTTTCATCTCTATCAAAATCGTTGACACCCTTCTCTCTCCTTATGTTGTTATATATTTTCCCGGCGGCTGAGACGGCAACGGCGACCACCGCAAAGGCTTTTAACCTCTCCACCATCACATTCGAAGAAGGATATTCCCCTCTTTTTAGTGATTTCAATATCGAACGATCTCCTGATGATACAAGCTTTCGTCTCCTCCTTAATCGTTTCTCTGGGTCTGGTGTAATTTCGACAGAATATTACAATTATGGATTTTTTAGCGCTAGTATTAAGCTGCCAGCCATATATACTGCCGGCATCGTTGTCGCATTTTATACGTCAAATGTGGACACATTTGAGAAGAATCAT***GACGAGTTAGACATCGAGTTTCTGGGG***AATGTGAACGGGCAGCCATGGAGATTTCAGACCAACTTGTATGGAAATGGGAGCGTAAGCCGTGGGAGAGAAGAGAGGTATAGAATGTGGTTTGATCCTAGCAACGACTTCCATCACTACAGCATTCTTTGGACCCCCAAAAATATCATATTCTACGTTGATGAGACACCAATAAGAGAAGTAAATCGTAATCCAGCAATGGGAGGGGACTTTCCATCAAAACCAATGTCCTTATATGCCACAATTTGGGATGCATCTTCTTGGGCTACAAATGGCGGCAAGGCTAAAGTTGACTACAAACATGAACCTTTTGCAACTGAGTTCAAAGACTTGGTTCTTGAAGGCTGTATAGTAGATCCCATTGAGCAAATTTCATCTACAAATTGCACTGATAGAATTGCCAGACTGCTTTCTCAAAACTACTCTATCATGACACCCGAAAGGCGAAAATCAATGAAATGGTTTAGAGAAAGATACATGTATTATTCTTATTGTTATGATAATATTAGGTACCCTGTGCCACCTCCAGAATGTGTTATTGTTCAATCAGAAAGAGACTTATTTAAGGATAGTGGAAGGCTTAGGCAGAAGATGAAGTTTGGTGGCAGCCACAGCCACCGGAAACACCGCCCTGGACGGAGCTCTAGGCGGCGGAATAGGGCTGCTGGTGGTGGTTCATCAAAGTCTGGCCAAGCTGCTGCAATGTAA

## >NtXTH50 amplification (same with SGN database sequence)

AGCTCGGATCACTAGTAACGGCCGCCAGTGTGCTGGAATTGCCCTTCAAT**ATG**GATTATCGAGTTCTTTCATCTCTATCAAAATCGTTGACACCCTTCTCTCTCCTTATGTTGTTATATATTTTCCCGGCGGCTGAGACGGCAACGGCGACCACCGCAAAGGCTTTTAACCTCTCCACCATCACATTCGAAGAAGGATATTCCCCTCTTTTTAGTGATTTCAATATCGAACGATCTCCTGATGATACAAGCTTTCGTCTCCTCCTTAATCGTTTCTCTGGGTCTGGTGTAATTTCGACAGAATATTACAATTATGGATTTTTTAGCGCTAGTATTAAGCTGCCAGCCATATATACTGCCGGCATCGTTGTCGCATTTTATACGTCAAATGTGGACACATTTGAGAAGAATCATGACGAGTTAGACATCGAGTTTCTGGGGAATGTGAACGGGCAGCCATGGAGATTTCAGACCAACTTGTATGGAAATGGGAGCGTAAGCCGTGGGAGAGAAGAGAGGTATAGAATGTGGTTTGATCCTAGCAACGACTTCCATCACTACAGCATTCTTTGGACCCCCAAAAATATCATATTCTACGTTGATGAGACACCAATAAGAGAAGTAAATCGTAATCCAGCAATGGGAGGGGACTTTCCATCAAAACCAATGTCCTTATATGCCACAATTTGGGATGCATCTTCTTGGGCTACAAATGGCGGCAAGGCTAAAGTTGACTACAAACATGAACCTTTTGCAACTGAGTTCAAAGACTTGGTTCTTGAAGGCTGTATAGTAGATCCCATTGAGCAAATTTCATCTACAAATTGCACTGATAGAATTGCCAGACTGCTTTCTCAAAACTACTCTATCATGACACCCGAAAGGCGAAAATCAATGAAATGGTTTAGAGAAAGATACATGTATTATTCTTATTGTTATGATAATATTAGGTACCCTGTGCCACCTCCAGAATGTGTTATTGTTCAATCAGAAAGAGACTTATTTAAGGATAGTGGAAGGCTTAGGCAGAAGATGAAGTTTGGTGGCAGCCACAGCCACCGGAAACACCGCCCTGGACGGAGCTCTAGGCGGCGGAATAGGGCTGCTGGTGGTGGTTCATCAAAGTCTGGCCAAGCTGCTGCAATG**TAA**TAAAAGGGCAATTCTGCAGATATCCATCACACTGGCGGCCGCTCGAGCATGCATCTAGA

# >NtXTH51(SGN database sequence)

ATGGATTTCATCAGAAAGAAGATATGTCTGTCTGTCTTCTTGTTTTTCCATGTCTGGTTTAGTACAGCCCTTAATGTCTCCACCATACCTTTTAGCGATGGCTTCAGCCATCTCTTTGGCGAAGGAAACATTCTTCATGCTACTGATGATAAGAGCCTTCAACTTCACCTCAACCAACGCACAGGTTCAGGGTTTAAATCTTCTGACCTCTACAACCATGGTTTCTTCAGTGCTAAGATAAAATTGCCATCAGATTATACTGCAGGAATCGTTGTTGCCTTCTATACGACGAATGGTGATTTATTTACAAAGACACAT***GATGAACTGGATTTTGAGTTTCTGGGA***AATATAAGAGGAAAAGCTTGGAGATTTCAGACAAATATGTATGGAAATGGAAGCACAAGTAGAGGAAGAGAAGAACGATATTATCTTTGGTTCGACCCTTCTAAAGAATTTCATCGTTACAGTATCCTGTGGACCAACAAAAACATCATATTTTATATAGATGATGTTCCAATTAGAGAAATCGTACGTAATGATGCAATGGGAGGAGACTATCCATCAAAGCCAATGGGACTATATGCAACAATATGGGATGCTTCAGATTGGGCTACTTCAGGAGGCAAATATAAAACAAATTACAAATATGCACCATTTATAGCTGAATTCACTGATTTAGTACTAAATGGATGTGCAATGGATCCATTGGAACAAGTAGTAAACAACCCTAGTTGTGATGAGAAAGATGATGAACTTCAAAAGGCAGATTTTTCAAGAATTACACCAAGACAAAGAATGGCTATGAAAAGATTTAGGTCAAAATATATGTATTATTCTTATTGTTACGATTCTTTGAGATACTCAGTGCCACCACCAGAATGCGAGATAGATCCAATTGAACAACAACATTTCAAAGAGACGGGGAGGTTGAAGTTTAACAAGCACCACCATCGCCATCCAAAGAGAACAAAAAGTCAAGTTCTTGATGCTAGGAATTATGGAAATCAAGATGAAGAGTGA

## >NtXTH51 amplification (same with SGN database sequence)

**ATG**GATTTCATCAGAAAGAAGATATGTCTGTCTGTCTTCTTGTTTTTCCATGTCTGGTTTAGTACAGCCCTTAATGTCTCCACCATACCTTTTAGCGATGGCTTCAGCCATCTCTTTGGCGAAGGAAACATTCTTCATGCTACTGATGATAAGAGCCTTCAACTTCACCTCAACCAACGCACAGGTTCAGGGTTTAAATCTTCTGACCTCTACAACCATGGTTTCTTCAGTGCTAAGATAAAATTGCCATCAGATTATACTGCAGGAATCGTTGTTGCCTTCTATACGACGAATGGTGATTTATTTACAAAGACACATGATGAACTGGATTTTGAGTTTCTGGGAAATATAAGAGGAAAAGCTTGGAGATTTCAGACAAATATGTATGGAAATGGAAGCACAAGTAGAGGAAGAGAAGAACGATATTATCTTTGGTTCGACCCTTCTAAAGAATTTCATCGTTACAGTATCCTGTGGACCAACAAAAACATCATATTTTATATAGATGATGTTCCAATTAGAGAAATCGTACGTAATGATGCAATGGGAGGAGACTATCCATCAAAGCCAATGGGACTATATGCAACAATATGGGATGCTTCAGATTGGGCTACTTCAGGAGGCAAATATAAAACAAATTACAAATATGCACCATTTATAGCTGAATTCACTGATTTAGTACTAAATGGATGTGCAATGGATCCATTGGAACAAGTAGTAAACAACCCTAGTTGTGATGAGAAAGATGATGAACTTCAAAAGGCAGATTTTTCAAGAATTACACCAAGACAAAGAATGGCTATGAAAAGATTTAGGTCAAAATATATGTATTATTCTTATTGTTACGATTCTTTGAGATACTCAGTGCCACCACCAGAATGCGAGATAGATCCAATTGAACAACAACATTTCAAAGAGACGGGGAGGTTGAAGTTTAACAAGCACCACCATCGCCATCCAAAGAGAACAAAAAGTCAAGTTCTTGATGCTAGGAATTATGGAAATCAAGATGAAGAG**TGA**AA

# >NtXTH52(SGN database sequence)

ATGGATTTCATCAGAAAGAAGATATGTCTGTCTGTCTTCTTGTTTTTCCATGTCTGCTTTATTACAGCTGATGCTGCCTTAAATGTCTCTACCATACCTTTTAGCGATGGCTTCAGCCATCTCTTTGGCGAAGGAAACATTCTTCATGCTACTGATGATAAGAGCCTTCAACTTCATCTCAACCAACGCACTGGTTCAGGATTCAAGTCGTCTGACCTCTACACCCATGGTTTCTTCAGTGCTAAGATAAAATTGCCATCAGATTATACTGCAGGGATCGTTGTTGCCTTCTATACGACGAATGGTGATTTATTTACAAAAACACAT***GATGAACTGGATTTTGAGTTTCTGGGA***AATATAAGAGGAAAAGCATGGAGATTTCAGACAAATATGTATGGAAATGGAAGCACAAGTAGAGGAAGAGAAGAACGATATTATCTTTGGTTTGACCCTTCTAAAGAATTTCATCGTTACAGTATCCTGTGGACCATCAAAAACATCATATTTTATATAGATGATGTTCCAATTAGAGAAATTGTACGTAATGATGCAATGGGAGGAGACTATCCATCAAAACCAATGGGATTATATGCAACAATATGGGATGCTTCAGATTGGGCTACTTCAGGAGGCAAATATAAAACAAATTACAAGTATGCACCATTTATAGCTGAATTCACTGATTTAGTATTAAATGGATGTGCAATGGATCCATTGGAACAAGTAGTAAACAACCCTAGTTGTGACGAGAAAGATGATGAACTTCAAAAGGCAGATTTTTCAAGGATTACACCAAGACAAAGAATGGCTATGAAAAGATTTAGGTCAAAATATATGTATTATTCTTATTGTTACGATTCTTTGAGATATTCAGTGCCACCACCAGAATGCGAGATAGATCACGTTGAACAACAACATTTCAAAGAGACGGGGAGGTTGAAATTTAACAAACACGGCCACCATCGTCATGCAAAGAGAACAAGAAGTCAAGTTCTTGATGCTAGGAACCATGGAAATCAGGATGAAGAGTGA

## >NtXTH52 amplification (same with SGN database sequence)

CTCTAGATGCATGCTCGAGCGGCCGCCAGTGTGATGGATATCTGCAGAATTGCCCTTCATCAGAAAGTACAGAAAAAACAGAGAAAGCTAAGACCAAAAGGAATAGTGAGGAAACAAAAG**ATG**GATTTCATCAGAAAGAAGATATGTCTGTCTGTCTTCTTGTTTTTCCATGTCTGCTTTATTACAGCTGATGCTGCCTTAAATGTCTCTACCATACCTTTTAGCGATGGCTTCAGCCATCTCTTTGGCGAAGGAAACATTCTTCATGCTACTGATGATAAGAGCCTTCAACTTCATCTCAACCAACGCACTGGTTCAGGATTCAAGTCGTCTGACCTCTACACCCATGGTTTCTTCAGTGCTAAGATAAAATTGCCATCAGATTATACTGCAGGGATCGTTGTTGCCTTCTATACGACGAATGGTGATTTATTTACAAAAACACATGATGAACTGGATTTTGAGTTTCTGGGAAATATAAGAGGAAAAGCATGGAGATTTCAGACAAATATGTATGGAAATGGAAGCACAAGTAGAGGAAGAGAAGAACGATATTATCTTTGGTTTGACCCTTCTAAAGAATTTCATCGTTACAGTATCCTGTGGACCATCAAAAACATCATATTTTATATAGATGATGTTCCAATTAGAGAAATTGTACGTAATGATGCAATGGGAGGAGACTATCCATCAAAACCAATGGGATTATATGCAACAATATGGGATGCTTCAGATTGGGCTACTTCAGGAGGCAAATATAAAACAAATTACAAGTATGCACCATTTATAGCTGAATTCACTGATTTAGTATTAAATGGATGTGCAATGGATCCATTGGAACAAGTAGTAAACAACCCTAGTTGTGACGAGAAAGATGATGAACTTCAAAAGGCAGATTTTTCAAGGATTACACCAAGACAAAGAATGGCTATGAAAAGATTTAGGTCAAAATATATGTATTATTCTTATTGTTACGATTCTTTGAGATATTCAGTGCCACCACCAGAATGCGAGATAGATCACGTTGAACAACAACATTTCAAAGAGACGGGGAGGTTGAAATTTAACAAACACGGCCACCATCGTCATGCAAAGAGAACAAGAAGTCAAGTTCTTGATGCTAGGAACCATGGAAATCAGGATGAAGAG**TGA**TAAGGGCAATTCCAGCACACTGGCGGCCGTTACTAGTgGATCCGAGCT

# >NtXTH53 (SGN database sequence)

ATGGTGAACTATCATCTTGTTACTTTCATATTTTTCTCTGTTGTTGAATTGGTTTATGGGTCTTCAAGAAATTTGCCAATTTTAGCGTTTGATGAAGGGTACTCCCATCTCTTTGGTGATGATAACGTTATGATCCTTAAAGATGGAAAATCTGCTCATATTTCTCTAGATGAAAGAACAGGGGCTGGATTTGTGTCTCAAGACCTATATCTTCATGGATTCTTCAGTGCTTCTATTAAGCTGCCTGCTGATTACACTGCTGGTGTGGTTGTTGCATTTTATATGTCTAATGTGGACATGTTTGAGAAGAACCAT***GATGAAATTGACTTTGAGTTCTTGGGA***AATATTAGAGGTAAAGACTGGAGAATTCAGACCAATATTTATGGGAATGGTAGCACTAGTGTTGGCAGAGAAGAAAGATATGGACTCTGGTTTGACCCTTCTGAAGATTTCCATCACTACAGTATCCTTTGGACTGAGAATTTCATCATCTTTTATGTAGATAATGTCCCCATAAGAGAGATCAAGAGGACAGAAGCTATGGGTGGGGACTTCCCATCTAAGCCAATGTCTTTGTATGCTACAATATGGGATGGTTCTGGTTGGGCTACCAATGGTGGAAAATACAAAGTCAATTACAAATACGCCCCGTATATTGCCAAGTTCTCTGATTTCGTCCTCCACGGATGCGCAGTTGATCCGATTGAACTATCATCCAAATGTGACACTGCACCAAAAACTGCATCAATCCCTACCGGTATTACCCCTGATCAAAGAAGAAAAATGGAGAAGTTTAGAAAGAAGCAAATGCAGTATTCGTACTGCTATGACAAGACTCGGTACAAGGTCCCTCCACCGGAATGTGTGATCGATCCTAAGGAAGCTGAACGACTCCGAGCCTTTGACCCGGTTACATTTGGCGGATCCCGCCACCATCACGGGAAACAACACCGCCGGAGCAGATCAAGAGCTGAGGGTGATATATCCTTTCTGTAA

## >NtXTH53 amplification (same with SGN database sequence)

TCTAGATGCATGCTCGAGCGGCCGCCAGTGTGATGGATATCTGCAGAATTGCCCTTCTCCATACAAATACTCCACAAACACAACTCTTTTGAGTGTTCCTAACTGATCAAAGAATACTAAACCCCTCAATAAAGGGGCAAAA**ATG**GTGAACTATCATCTTGTTACTTTCATATTTTTCTCTGTTGTTGAATTGGTTTATGGGTCTTCAAGAAATTTGCCAATTTTAGCGTTTGATGAAGGGTACTCCCATCTCTTTGGTGATGATAACGTTATGATCCTTAAAGATGGAAAATCTGCTCATATTTCTCTAGATGAAAGAACAGGGGCTGGATTTGTGTCTCAAGACCTATATCTTCATGGATTCTTCAGTGCTTCTATTAAGCTGCCTGCTGATTACACTGCTGGTGTGGTTGTTGCATTTTATATGTCTAATGTGGACATGTTTGAGAAGAACCATGATGAAATTGACTTTGAGTTCTTGGGAAATATTAGAGGTAAAGACTGGAGAATTCAGACCAATATTTATGGGAATGGTAGCACTAGTGTTGGCAGAGAAGAAAGATATGGACTCTGGTTTGACCCTTCTGAAGATTTCCATCACTACAGTATCCTTTGGACTGAGAATTTCATCATCTTTTATGTAGATAATGTCCCCATAAGAGAGATCAAGAGGACAGAAGCTATGGGTGGGGACTTCCCATCTAAGCCAATGTCTTTGTATGCTACAATATGGGATGGTTCTGGTTGGGCTACCAATGGTGGAAAATACAAAGTCAATTACAAATACGCCCCGTATATTGCCAAGTTCTCTGATTTCGTCCTCCACGGATGCGCAGTTGATCCGATTGAACTATCATCCAAATGTGACACTGCACCAAAAACTGCATCAATCCCTACCGGTATTACCCCTGATCAAAGAAGAAAAATGGAGAAGTTTAGAAAGAAGCAAATGCAGTATTCGTACTGCTATGACAAGACTCGGTACAAGGTCCCTCCACCGGAATGTGTGATCGATCCTAAGGAAGCTGAACGACTCCGAGCCTTTGACCCGGTTACATTTGGCGGATCCCGCCACCATCACGGGAAACAACACCGCCGGAGCAGATCAAGAGCTGAGGGTGATATATCCTTTCTGTAAAAGGGCAATTCCAGCACACTGGCGGCCGTTACTAGTGATCCGAGCTC

# >NtXTH54 (SGN database sequence)

ATGGTGAACTATCATCTTGTTATTTTCATATTTTTCTCTGTTGTTGAATTGGTTTATGGGTCTTCAAGAAATTTGCCAATTTTAGCGTTTGATGAAGGCTACTCCCATCTCTTTGGTGATAATAACCTTATGATCCTTAAAGATGGAAAATCTGCTCATATTTCTCTAGATGAAAGAACAGGGGCTGGATTTGTGTCTCAAGACCTATATCTTCATGGATTCTTCAGTGCTTCTATTAAGCTTCCTGCTGATTACACTGCTGGTGTGGTTGTTGCATTTTATATGTCTAATGTGGACATGTTTGAGAAGAACCAT***GATGAAATTGACTTTGAGTTCTTGGGA***AATATTAGAGGCAAAGACTGGAGAATTCAGACCAATATTTATGGGAATGGTAGTACTAGTTTTGGCAGAGAAGAAAGATATGGACTCTGGTTTGACCCTTCTGAAGATTTCCATCACTACAGTATCCTTTGGACTGAGAATTTTATCATCTTTTATGTAGATAATGTCCCCATTAGAGAGATCAAGAGGACAGAAGCTATGGGTGGGGACTTCCCATCTAAGCCAATGTCTTTGTATGCTACAATATGGGATGGTTCTGGTTGGGCTACCAATGGTGGAAAATACAAAGTCAATTACAAATATGCCCCGTATATTGCCAAGTTCTCTGATTTCGTCCTCCACGGATGCGCGGTTGATCCGATTGAATTATCATCCAAATGTGACACTGCACCAAAAACTTCATCAATCCCTACAGGTATTACCCCTGATCAAAGAAGAAAAATGGAGAACTTCAGAAAGAAGCAAATGCAGTATTCTTACTGCTATGACAAGACTCGGTACAAGGTCCCTCCAACGGAATGTGTGATCGATCCTAAGGAAGCTGAACGACTCCGAGTCTTTGACCCCGTTACATTTGGCGGATCCCGCCACCATCATGGGAAACGACATAGCCGGAGCAGATCAAGGGCTGAGGGTGATGTATCCTTTCTGTAA

## >NtXTH54 amplification (same with SGN database sequence)

TATTAAGAAA**ATG**GTGAACTATCATCTTGTTATTTTCATATTTTTCTCTGTTGTTGAATTGGTTTATGGGTCTTCAAGAAATTTGCCAATTTTAGCGTTTGATGAAGGCTACTCCCATCTCTTTGGTGATAATAACCTTATGATCCTTAAAGATGGAAAATCTGCTCATATTTCTCTAGATGAAAGAACAGGGGCTGGATTTGTGTCTCAAGACCTATATCTTCATGGATTCTTCAGTGCTTCTATTAAGCTTCCTGCTGATTACACTGCTGGTGTGGTTGTTGCATTTTATATGTCTAATGTGGACATGTTTGAGAAGAACCATGATGAAATTGACTTTGAGTTCTTGGGAAATATTAGAGGCAAAGACTGGAGAATTCAGACCAATATTTATGGGAATGGTAGTACTAGTTTTGGCAGAGAAGAAAGATATGGACTCTGGTTTGACCCTTCTGAAGATTTCCATCACTACAGTATCCTTTGGACTGAGAATTTTATCATCTTTTATGTAGATAATGTCCCCATTAGAGAGATCAAGAGGACAGAAGCTATGGGTGGGGACTTCCCATCTAAGCCAATGTCTTTGTATGCTACAATATGGGATGGTTCTGGTTGGGCTACCAATGGTGGAAAATACAAAGTCAATTACAAATATGCCCCGTATATTGCCAAGTTCTCTGATTTCGTCCTCCACGGATGCGCGGTTGATCCGATTGAATTATCATCCAAATGTGACACTGCACCAAAAACTTCATCAATCCCTACAGGTATTACCCCTGATCAAAGAAGAAAAATGGAGAACTTCAGAAAGAAGCAAATGCAGTATTCTTACTGCTATGACAAGACTCGGTACAAGGTCCCTCCAACGGAATGTGTGATCGATCCTAAGGAAGCTGAACGACTCCGAGTCTTTGACCCCGTTACATTTGGCGGATCCCGCCACCATCATGGGAAACGACATAGCCGGAGCAGATCAAGGGCTGAGGGTGATGTATCCTTTCTG**TAA**AAGCAAGGCCACAAG

# >NtXTH55 (SGN database sequence)

ATGGTGAATTTTCGTCTGGAAATTTTCATATTATGCTCTTTTCTTGTATTAGTTTGTGGGTCTTCAAAACAGCTCCAAACTTTACCGTTTGACGAAGGGTACTCACAACTCTTTGGCCATGATAATCTTATGGTTCTTGAAGATGGAAAGTCAGTTCATCTTTCTCTAGATGAAAGAACAGGAGCAGGATTTGTGTCTCAAGATCTTTACCTTCATGGCTACTTCAGTGCTTCTATTAAGTTACCAGCAGATTACACTGCTGGAGTGGTTGTTGCATTTTATATGTCTAACGGCGACATGTTTGAGAAGAACCAT***GATGAAATTGACTTTGAGTTCTTGGGA***AATATAAGAGCAAAAAAATGGAGGATTCAAACTAATATATATGGGAATGGTAGCACAAATGTTGGCAGAGAAGAAAGATATGGACTCTGGTTTGATCCCTCTGAAGATTTTCATCAATATAGCATCTTGTGGACTGAGAGCCAGATCATCTTTTATGTAGATAATATCCCCATAAGAGAGATCAAGAGGACAAAAGCAATGGGTGGGGACTTCCCTTCTAAGCCAATGTCTTTGTATGCTACAATATGGGATGGTTCTAGTTGGGCTACCAATGGGGGCAAATACAAAGTCAATTACAAATATGCCCCTTATGTCGCCAAGTTTTCCGACTTTATCCTTCATGGATGTGCAGTTGATCCAATTGAATTGTCACCAAAATGTGACACAACCCCTAATTCTGCATCCATTCCAACTAGTATATCCCCTGATCAAAGAAGAAAAATGGAGAGCTTCCGAAAGAAGTACTTGCAATATTCATACTGCTATGACCGGACTCGATACAATGTACCTCTATCTGAATGTGTAATTGATCCTAAGGAAGCTGACCGTCTCCGAGGCTTTGACCCCGTGACCTTTGGTGGCGTCCAGCGCCATCACAGCAAACGACACCACCAGAGGCAATCGAGGAGGGAAGACACGTCTTCTGAATAG

## >NtXTH55 amplification (same with SGN database sequence)

GCTACTACTTATTTTTCTGTTCAACCAAAGGGACCAAAAACAAA**ATG**GTGAATTTTCGTCTGGAAATTTTCATATTATGCTCTTTTCTTGTATTAGTTTGTGGGTCTTCAAAACAGCTCCAAACTTTACCGTTTGACGAAGGGTACTCACAACTCTTTGGCCATGATAATCTTATGGTTCTTGAAGATGGAAAGTCAGTTCATCTTTCTCTAGATGAAAGAACAGGAGCAGGATTTGTGTCTCAAGATCTTTACCTTCATGGCTACTTCAGTGCTTCTATTAAGTTACCAGCAGATTACACTGCTGGAGTGGTTGTTGCATTTTATATGTCTAACGGCGACATGTTTGAGAAGAACCATGATGAAATTGACTTTGAGTTCTTGGGAAATATAAGAGCAAAAAAATGGAGGATTCAAACTAATATATATGGGAATGGTAGCACAAATGTTGGCAGAGAAGAAAGATATGGACTCTGGTTTGATCCCTCTGAAGATTTTCATCAATATAGCATCTTGTGGACTGAGAGCCAGATCATCTTTTATGTAGATAATATCCCCATAAGAGAGATCAAGAGGACAAAAGCAATGGGTGGGGACTTCCCTTCTAAGCCAATGTCTTTGTATGCTACAATATGGGATGGTTCTAGTTGGGCTACCAATGGGGGCAAATACAAAGTCAATTACAAATATGCCCCTTATGTCGCCAAGTTTTCCGACTTTATCCTTCATGGATGTGCAGTTGATCCAATTGAATTGTCACCAAAATGTGACACAACCCCTAATTCTGCATCCATTCCAACTAGTATATCCCCTGATCAAAGAAGAAAAATGGAGAGCTTCCGAAAGAAGTACTTGCAATATTCATACTGCTATGACCGGACTCGATACAATGTACCTCTATCTGAATGTGTAATTGATCCTAAGGAAGCTGACCGTCTCCGAGGCTTTGACCCCGTGACCTTTGGTGGCGTCCAGCGCCATCACAGCAAACGACACCACCAGAGGCAATCGAGGAGGGAAGACACGTCTTCTGAA**TAG**AAAGAA

# >NtXTH56 (SGN database sequence)

ATGGTGAATTTTCGTCTGGGAATTTTCATACTATGTTCTTTTCTTGTATTAGTTTCAGGGTCTTCAAAAAAGCTCCAAACGTTACCGTTTGATGAAGGGTACTCGCAACTCTTTGGTCATGATAATCTTATGGTTCTTGAAGATGGAAAATCAGTTCATATTTCTCTTGATGAAAGAACAGGAGCAGGATTTGTGTCTCAAGACCTCTACCTTCATGGCTACTTCAGTGCTTCTATTAAGTTACCTGCAGATTACACTGCTGGAGTGGTTGTTGCATTTTATATGTCTAATGGTGACATGTTTGAGAAGAGCCAT***GATGAAATTGACTTTGAATTCTTGGGA***AATATAAGAGCAAAAAACTGGAGGATTCAAACTAATATATATGGGAATGGTAGCACAAATGTTGGCAGAGAAGAAAGATATGGACTCTGGTTTGATCCTTCTGAAGATTTTCATCAATATACCATCCTCTGGACTGAGAGCCAGATCATCTTTTATGTAGATAATATCCCCATAAGAGAGATCAAAAGGACAAAAGCAATGGGTGGGGACTTCCCTTCTAAGCCAATGTCTTTATATGCTACAATATGGGATGGTTCTAGTTGGGCTACCAATGGGGGCAAATACAAAGTCAATTACAAATATGCCCCTTACGTCGCCAAGTTTTCCGACTTTGTCCTCCACGGATGTGCAGTTGATCCAATTGAATTGTCACCAAAATGTGACACTGCACCTAAGTCTGCATTCGTTCCAACTGGTATATCCCCTGATCAAAGAAGAAAAATGGAGAGCTTCCGAAAGAAGTACTTGCAATATTCGTATTGTTATGACCGGACTCGATACAATGTACCTCTATCTGAATGTGTTATTGATCCTAAGGAAGCGGATCGTCTCCAAGGCTTTGATCCCGTGACCTTTGGTGGCGTCCAGCGTCATCACAGCAAACGACGCCGTCAGAGGCAATCGAGGAGAGAAGACGCGTCTTCTGAATAG

## >NtXTH56 amplification (same with SGN database sequence)

TCTGTTCACAAAACAA**ATG**GTGAATTTTCGTCTGGGAATTTTCATACTATGTTCTTTTCTTGTATTAGTTTCAGGGTCTTCAAAAAAGCTCCAAACGTTACCGTTTGATGAAGGGTACTCGCAACTCTTTGGTCATGATAATCTTATGGTTCTTGAAGATGGAAAATCAGTTCATATTTCTCTTGATGAAAGAACAGGAGCAGGATTTGTGTCTCAAGACCTCTACCTTCATGGCTACTTCAGTGCTTCTATTAAGTTACCTGCAGATTACACTGCTGGAGTGGTTGTTGCATTTTATATGTCTAATGGTGACATGTTTGAGAAGAGCCATGATGAAATTGACTTTGAATTCTTGGGAAATATAAGAGCAAAAAACTGGAGGATTCAAACTAATATATATGGGAATGGTAGCACAAATGTTGGCAGAGAAGAAAGATATGGACTCTGGTTTGATCCTTCTGAAGATTTTCATCAATATACCATCCTCTGGACTGAGAGCCAGATCATCTTTTATGTAGATAATATCCCCATAAGAGAGATCAAAAGGACAAAAGCAATGGGTGGGGACTTCCCTTCTAAGCCAATGTCTTTATATGCTACAATATGGGATGGTTCTAGTTGGGCTACCAATGGGGGCAAATACAAAGTCAATTACAAATATGCCCCTTACGTCGCCAAGTTTTCCGACTTTGTCCTCCACGGATGTGCAGTTGATCCAATTGAATTGTCACCAAAATGTGACACTGCACCTAAGTCTGCATTCGTTCCAACTGGTATATCCCCTGATCAAAGAAGAAAAATGGAGAGCTTCCGAAAGAAGTACTTGCAATATTCGTATTGTTATGACCGGACTCGATACAATGTACCTCTATCTGAATGTGTTATTGATCCTAAGGAAGCGGATCGTCTCCAAGGCTTTGATCCCGTGACCTTTGGTGGCGTCCAGCGTCATCACAGCAAACGACGCCGTCAGAGGCAATCGAGGAGAGAAGACGCGTCTTCTGAA**TAG**AAAGAAGGGTATTAATTAGTCTCTACATTGGATGTTGATA
