# Supplementary material for: Genome-Wide Identification and Expression Profiling Analysis of the Xyloglucan Endotransglucosylase/Hydrolase Gene Family in Tobacco (Nicotiana tabacum L.)
Source: Genes (Basel). 2018 May 24;9(6):273. doi: 10.3390/genes9060273 (PMC6027287; doi:10.3390/genes9060273)
Supplement: Supplementary file 1 [file genes-09-00273-s001.zip › Supplementary File 3.docx]

**Supplementary File 3: Coding sequences of *N. tabacum NtXTHs*.**

# >NtXTH1

ATGAATAACTTCTCTACACTTATTTTCTTTGTCACTGCTTTTATTTATTTGTTTCATATTACATTAGCTTCCATAGTTTCAACAGGAGATTTCAATAAGGATTTTATAGTGCCTTGGTCCCCTAACCATGTAAATACTTCTGCCGATGGCCATACAAGAAGCTTGATATTTGATAAGGAATCTGGTTCAGGGATTGCTTCAAATGATACGTACTTGTTTGGTCAATTCGACATGAAAATTAAGTTGATACCAGGAAATTCCGCAGGCACGGTCGTGGCATTTTATTTAACTTCGTATCAACCAAATCGT***GACGAGGTAGATTTTGAGTTTCTGGGA***AATGTTCCTGGGAAACCTTATACTCTTCAAACGAATGTTTATGTCGATGGGTTGGACGATAGAGAACAGAGAATCAACTTGTGGTTTGATCCAACACAAGACTTCCACACTTATTCTATTCTGTGGAACCTTCACCAAATTGTGTTTATGGTTGATCGGGTACCTATTAGAACGTACAGAAACCATGCAGATAAAGGAGCTAAATATCCTCGTTGGCAACCAATGGCACTCCAAATTAGCATATGGAATGGAGAAAGTTGGGCAACAGATGGTGGAAAAACAAAAATTGATTGGTCAAAAGCACCATTTGTAGCCTCTTTAGGAAATTACACAATTGATGCTTGCGTTTGGAAAGGAAATGCAAGATTTTGCAGAGGAGAAAGTGAAAATAATTGGTGGAATAAGGAGAAATTCAGCACTTTGACATGGACTCAAAGAAGGTTGTTCAAATGGGTCAGAAAATATCATTTGACATATGATTATTGCATGGATAATCAACGGTTTCAAAATAATCTTCCCATAGAGTGCTCTCTCCCAAAGTATTAA

# >NtXTH2

ATGAAGTTGAAATTGGTAGGAGGTGACTCTGCTGGTGTTGTCACAGCTTATTATATGTGCACAGAAGATGGGGCAGGGCCAACTAGA***GATGAGGTAGACTTTGAGTTTTTGGGA***AATAGGACAGGGGAACCCTATCTTATTCAGACCAATGTGTACAAAAATGGCACTGGTGGGCGTGAGATGAGGCACGTTCTCTGGTTTGACCCCACTGAGGACTTCCATTCCTATTCCCTTCTTTGGAACTCTCACCAGCTCGTGTTTTTCGTGGATGAGGTTCCGATAAGGGTATACAAAAACGCGAATTATACGAACAATTTCTTTCCTAATGAGAAACCAATGTACTTGTTTTCAAGCATATGGAATGCAGATGACTGGGCTACTAGGGGTGGTTTGGAGAAAACAGATTGGAAAAATGCACCATTTGTTTCAACATATAAAGATTTCAGTGTAGATGGTTGCCAATGGGAAGATCCTTTTCCTACTTGTGTTTCAACAACCACTAAAAACTGGTGGGATCAGTACAATTCTTGGCACTTATCAAGTGACCAGAAATTGAATTATGCTTGGGTACAACGAAACCTTGTGATTTATGATTATTGCCAGGATACAAAGAGATATCCAGAAAAGCCTGAGGAATGTTGGTTAAGTCCCTGGGATTAA

# >NtXTH3

ATGGCTAATCTTCTCTTAATTGCAGTTTTAATTGCTATTTATTGTTCACTATCTCAAGCTGAAGTTAAAGGTTCATTTGATGACAACTTTAGTAAAAGTTGTCCTGAATCTCACTTCAAGACTTCTGAAGATGGACAGATCTGGTATCTCTCCTTAGACCACAAAGCAGGATGTGGATTTATGACAAGGCAGAAATACAGATTTGGTTGGTTTAGCATGAAGTTGAAATTGGTAGGAGGTGACTCTGCTGGTGTTGTCACGGCTTATTATATGTGCACAGAAGATGGGGCAGGGCCAACTAGA***GATGAGGTAGACTTTGAGTTTTTGGGA***AATAGAACAGGGGAACCCTATCTTATTCAGACCAATGTGTACAAAAATGGCACTGGTGGGCGTGAGATGAGGCACGTTCTCTGGTTTGACCCTACTGAGGACTTCCATTCCTATTCTCTTCTTTGGAACTCTCACCAACTCGTGTTTTTCGTGGATGAGGTTCCGATAAGGGTATACAAAAACACGAATTATACGAACAATTTCTTCCCTAATGAGAAGCCAATGTACTTGTTTTCGAGCATATGGAATGCAGATGATTGGGCTACTAGGGGTGGTTTGGAGAAAACAGATTGGAAAAATGCACCATTTGTTTCAACATATAAAGATTTTAGTGTAGATGGTTGCCAATGGGAAGATCCTTTTCCTTCTTGTGTTTCAACCACCACTGAAAACTGGTGGGATCAATACAATTCTTGGCATTTATCAAGTGACCAGAAATTGGATTATGCTTGGGTACAAAGAAACCTTGTGATTTATGATTATTGTCAGGATACAGAGAGATATCCAGAAAAGCCTGAGGAGTGTTGGTTAAGTCCCTGGGATTAA

# >NtXTH4

ATGGAGAGAATGTCTTCTTCAATACCTAAATTCCTTCTAATTATAGCACTAATTACTGTTCTTTTTACATTAACACAAGCTGAAGTACAAGGTTCATTTGATGACAATTTTAGTAAAAGTTGTCCTGAAACACATTTCAAGACTTCTGAAGATGGACAGATCTGGTATCTTTCATTAGATAAGAAAGCAGGATGTGGATTTATGACCAGGCAAAAATATAGATTTGGGTGGTTTAGTATGAAGTTGAAATTGGTGGGAGGTGACTCTGCCGGTGTTGTCACAGCTTACTATATGTGTACAGAAGATGGAGCAGGGCCAACAAGA***GATGAATTAGACTTTGAGTTCTTGGGA***AATAGAACAGGGGAACCTTATACTATTCAAACCAATGTGTACAAAAATGGGACTGGTAATCGTGAGATGAGACACATTCTTTGGTTTGACCCCACCGAGGACTTTCATACTTATTCCATTCTTTGGAACACCCACCAAATTGTGTTTTTCGTGGATAGAGTACCAATAAGGGTATACAAAAATGCGAATTATACGAATAATTTCTTCCCAAATGAGAAGCCAATGTACTTGTTTTCAAGCATATGGAATGCTGACGATTGGGCTACAAGAGGTGGTTTGGAGAAAACAAATTGGAAAAATCAACCATTTGTTTCAAGTTATAAGGATTTTAGTGTAGATGGTTGTCAATGGAAAGATCCATTTCCTGCTTGTGTTTCTACTACCACTAAAAATTGGTGGGATCAATATAATTCTTGGCATTTATCAAGTGACCAAAAAATGGATTATGCTTGGGTACAGAGAAATCTTGTGACTTATGATTATTGCCAAGATACTGAGAGATTTCCTAAAAAGCCTGAGGAATGTTGGTTAAATCCATGGGATTAA

# >NtXTH5

ATGGAGAAAATGGCTTCTTCAATACCTAAAATCCTTCTAATTATAGCACTAATTACTGTTCTTTTTTCATTAACACAAGCTGAAGTACAAGGTTCATTTGATGATAATTTTAGTAAAAGTTGTCCTGAAACACATTTCAAGACTTCTGAAGATGGACAGATCTGGTATCTTTCATTAGATAAAAAAGCAGGATGTGGATTTATGACTAAGCAGAAATATAGATTTGGGTGGTTTAGTATGAAGTTGAAATTGGTGGGAGGTGACTCTGCTGGTGTTGTCACAGCTTATTATATGTGCACAGAAGATGGAGCAGGACCAACAAGA***GATGAATTAGACTTTGAGTTCTTGGGA***AATAGAACAGGGGAACCCTATACTATTCAAACCAATGTGTATAAAAATGGGACTGGTAACCGTGAAATGAGACACATTCTATGGTTTGACCCCACTGAGGATTTCCACACTTATTCCATTCTTTGGAACACTCACCAAATTGTGTTTTTCGTGGATAGAGTACCGATAAGGGTATACAAAAATGCGAACTATACGAATAATTTCTTCCCAAATGAGAAGCCAATGTACTTATTTTCAAGCATATGGAATGCTGATGATTGGGCAACAAGAGGTGGTTTGGAGAAAACAAATTGGAAAAATCAACCATTTGTTTCAAGTTATAAAGATTTTAGTGTAGATGGTTGTCAATGGAAAGATCCATTTCCTGCTTGTGTTTCGACAACCACTAAAAATTGGTGGGATCAATATAATTCTTGGCATTTATCAAGTGACCAAAAAATGGATTATGCTTGGGTTCAAAGGAACCTTGTGACTTATGATTATTGCCAAGATACAGAGAGATTTCCTAAAAAGCCTGAGGAATGTTGGTTAAATCCATGGGAATAA

# >NtXTH6

ATGGAGAGAAATGCTTCTTCAATGGCTGATCTTTTCTTCACTGCAGCACTAATGGCTGCACTCTTTTCATCCTCACATGCTGAACTCATCAAAGGTGCATTTGAAAACAACTTTAGTAAAAGTTGTCCTGGTACACATTTCAAGACTTCTCAAGATGGACAGATCTGGTATCTCACCTTAGACCAAATATCAGATTGTGGGTTTATTACTAAGCAGAGTTATAGATTTGGTTGGTTTAGCACAAAGTTGAAATTAGTAGGAGGTGACTCTGCTGGTGTTGTGACAGCCTTTTATATGTGTTCGGAAGTGGAGGCAGGGCCATTGAGA***GATGAGATAGATTTTGAGTTCTTGGGA***AACAGAACAGGACAACCTTATCTTATTCAAACCAATGTGTACAATAATGGCAGTGGTGGACGTGAGATGAGGCATCTTCTTTGGTTTGATCCCACTCAAGACTTCCATACCTATTCCATTCTTTGGAACTCTCACCAAATTGTGTTTTTTGTTGATAAGGTTCCGATAAGGGTATACAAGAACGCGAATCACACGAACAATTTTTTTCCAGCTGAGAGGCCAATGTACGTGTTTTCTAGCATATGGAATGCAGATAATTGGGCTACTAGAGGAGGATTGGACAAGATAAACTGGACAAGTGCACCATTTATAGCAAGTTATAAGGATTTTATTTTAGATGCTTGTCAATGGAAAGATCCTTTCCCTGCTTGTGTTTCCACCACTACACAGCATTGGTGGGATCAATATAATGCTTGGCACCTATCAAGTAAACAGAAGATTGATTATGCTTGGGTGCAGAGAAACTTTGTAGTTTATGATTATTGCCAGGATAGTGTGAGAAACCGTTATAAGCCCCAAGAGTGTTGGTTAAGTGCATTGGACTAA

# >NtXTH7

ATGGAGAGAAATATGGGTGATCTTCTCTTATTTGCAGCACTAGTGGCTACCCTTTTTTCATCATCACATGCTCAACTTATCAAAGGTGCATTTGAAAACACCTTCAGTAAAAGCTGTCCGGGTACTCATTTCAAGACTTCTCAAGATGGACAGATCTGGTATCTCACCTTAGACCAAGTATCAGATTGTGGGTTTATCACCAAGCAGAGCTATAGATTTGGTTGGTTTAGCACAAAGTTGAAATTGGTAGGAGGTGACTCTGCTGGTGTTGTGACAGCCTTTTATATGTGCTCGGAAGTAGAGGCAGGACC***ATTGAGAGATGAGATAGATTTTGAGTT***CTTGGGAAACAGAACAGGGCAGCCTTATCTTATTCAGACCAATGTGTACAATAATGGCAGTGGTGGACGTGAGATGAGGCATCTTCTCTGGTTTGACCCTACTCAGGACTTTCATACCTATTCCATTCTTTGGAACTCCCACCAAATTGTGTTTTTCGTTGATAAGGTTCCAATAAGGGTATACAAGAACGCGAATCACACTAACAATTTTTTTCCAGCTGAGAGGCCAATGTACGTGTTTTCTAGCATATGGAATGCAGATAATTGGGCTACTAGAGGAGGGTTGGACAAGATAAACTGGACAAGTGCACCATTCGTAGCAAGTTATAAGGAGTTTACTTTAGATGCTTGTCAATGGAAAGATCCTTTCCCAGCTTGTGTTTCCACCACTACACAGCACTGGTGGGATCAGTATAATGCTTGGCACCTATCAAGTAAACAGAAGATTGATTATACTTGGGTGCAGAGAAACTTTGTAGTTTATGATTATTGCCAGGATAGTGTAAGAAACCGTTACAAGCCTCAAGAGTGTTGGTTAAGTCCATTGGACTAA

# >NtXTH8

ATGAAGCAAGTAATTGAATATCGTTGCCTTCTGATTTTAGGATGTGGGTTTGCTTCCAAAAGCAAATACCTCTTTGGACGTGTTAGCATGAAGATCAAGCTCGTTCCTGGTGACTCTGCTGGAACTGTCACCGCCTTTTACATGAACTCGGACACAGATAACGTAAGG***GACGAGCTAGACTTCGAGTTCTTGGGA***AACAGGTCAGGCCAGCCGTACACTGTCCAAACGAATGTTTATGTCCATGGAAAGGGTGACAAGGAACAAAGGATCAACCTTTGGTTCGATCCATCCGCTGATTTTCATACCTACACCATTCTTTGGAACCACCATCACACTGTATTCTACGTGGACGCAGTACCCATTAGAGTGTACAAGAATAACGAAGCAAAAGGAATCCCATTCCCTAAATTCCAACCCATGGGAGTGTACTCAACATTGTGGGAAGCCGACGACTGGGCAACAAGAGGTGGATTAGAGAAAATAAATTGGAGCAAATCCCCATTTTACGCATACTACAAGGACTTTGACATAGAGGGATGTGCAATGCCAGGACCAGCAAACTGTGCCTCAAATCCACGCAATTGGTGGGAAGGTGCTAATTACCAACAGCTCAGTGCTGTGGAAGCAAGGCAATATCGCTGGGTTAGAACGAACCACATGATCTATGATTATTGCACTGACAAATCCAGAAATCCAGTTCCCCCACCAGAATGTGTGGCCGGAATATGA

# >NtXTH9

ATGATTTCCTCTTCTTTAAAATATTCAACTGTCATTCCAATCTTGCTATATGCCTTGACCTTTTCTTCCTCAGTAAGTGCACGACCCGCCACTTTTTTACAGGACTTTAAAGTGGCATGGGCTGACTCTCACATCAAGCAAATCGATGGCGGCAAGGCTATTCAGCTTATACTCGACCAAAACTCAGGATGTGGGTTTGCTTCCAAAAGCAAATACCTCTTTGGACGTGTTAGCATGAAGATCAAGCTCGTTCCTGGTGACTCTGCTGGAACTGTCACTGCCTTTTACATGAACTCGGACACAGATAATGTAAGG***GACGAGCTAGACTTCGAGTTCTTGGGA***AACAGGTCAGGCCAGCCGTACACTGTCCAAACGAATGTTTATGTCCATGGAAAGGGTGACAAGGAACAAAGGATCAACCTTTGGTTCGATCCATCCGCTGATTTTCATACCTACACCATACTTTGGAACCACCATCACACTGTATTCTACGTAGACGCAGTACCAATTAGAGTGTACAAGAACAACGAAGCAAAAGGAATCCCATTCCCCAAATTCCAACCCATGGGAGTTTATTCAACATTATGGGAAGCCGACGACTGGGCAACGAGAGGTGGATTAGAGAAAATAAATTGGAGCAGATCCCCATTTTACGCATATTACAAGGACTTTGACATAGAGGGATGTGCAATGCCAGGACCAGCAAACTGTGCCTCAAATCCTCGCAATTGGTGGGAAGGAGCTAATTACCAACAACTCAGTGCTGTGGAAGCAAAGCAATATCGCTGGGTTAGAATGAACCACATGATCTATGATTATTGCACTGACAAATCCAGAAATCCAGTAACCCCACCAGAATGTGTGGCCGGAATATGA

# >NtXTH10

ATGGGCAAATTGACGTCCTTAAAATATTCAGCTGCAATTCTAATATTGCTATATGCCTTGACCTTTTCCTTCTCAGTGAGTGCACGACCCGCCACTTTTCTACAGGACTTTAAGGTCTCTTGGGCCTACTCTCACATCAAACAAATCGATGGCGGCAGGGCCATTCAGCTTATTCTCGACCAAAACTCAGGATGTGGGTTTGCTTCCAAAAGCAAATACCTCTTTGGACGTGTTAGCATGAAGATCAAGCTCGTGCCTGGTGACTCTGCTGGAACCGTCACCGCCTTTTACATGAATTCGGACACAGACAACGTAAGG***GACGAGCTAGACTTCGAGTTCTTGGGA***AACAGGTCAGGCCAGCCGTACACTGTCCAGACGAATGTTTATGTTCATGGAAAAGGTGACAAGGAACAAAGGGTCAACCTTTGGTTCGATCCATCCGCTGATTTTCACACTTATACCATTCTTTGGAACCACCACCACGCCGTATTCTACGTGGACGCAGTACCCATTAGAGTGTACAAGAACAACGAAGCAAAAGGAATCCCATTCCCCAAATTCCAACCCATGGGAGTGTATTCCACATTGTGGGAAGCCGATGACTGGGCAACGAGAGGTGGATTAGAGAAAATAAATTGGAGCAAATCCCCATTTTACGCATACTACAAGGACTTTGACATAGAGGGATGTGCAATGCCAGGACCAGCAAACTGTGCCTCAAATCCACGCAATTGGTGGGAAGGAGCTAATTACCAACAACTCAGTGCTGTGGAAGCAAGGCAATATCGCTGGGTTAGAATGAACCACATGATCTATGATTATTGCACTGACAAATCCAGAAATCCAGTCACCCCACCAGAATGTGTGGCCGGAATATGA

# >NtXTH11

ATGGCCAGATTGACTTCCTTAAAATATTCAGCTGCAATTCTAATATTGCTATATGCCTTGACCTTTTCATTCTCAGTGAGTGCACGACCCGCCACTTTTTTACAGGACTTTAAGGTCTCTTGGTCCGACTCTCACATCAAACAAATTGATGGTGGCAGGGCCATTCAGCTTATTCTCGACCAAAACTCAGGATGTGGGTTTGCTTCCAAAAGCAAATACCTCTTTGGACGTGTTAGCATGAAGATCAAGCTCGTACCTGGTGACTCTGCTGGAACCGTCACTGCCTTTTACATGAACTCGGACACAGACAACGTAAGG***GACGAACTAGACTTCGAGTTCTTGGGA***AACCGGTCAGGCCAGCCGTATACTGTCCAGACGAATGTTTATGTTCATGGAAAAGGTGACAAGGAACAAAGAGTCAACCTTTGGTTCGATCCATCCGCTGATTTTCACACTTATACCATTCTTTGGAACCACCACCACGCCGTATTCTACGTGGATGCGGTACCCATTAGAGTCTACAAGAACAACGAAGCAAAAGGAATTCCATTCCCCAAATTCCAACCCATGGGAGTGTACTCAACATTGTGGGAAGCCGACGACTGGGCAACGAGAGGTGGATTAGAGAAAATAAATTGGAGCAAATCCCCATTTTACGCATATTACAAGGACTTTGACATAGAGGGATGTGCAATGCCAGGACCAGCAAACTGTGCCTCAAATCCTCGCAATTGGTGGGAAGGAGCTAATTACCAACAACTCAGTGCTGCGGAAGCAAGGCAATATCGCTGGGTTAGAATGAACCACATGATCTATGATTATTGCACCGACAAATCCAGAAATCCAGTCACCCCACCAGAATGTGTGGCTGGAATATGA

# >NtXTH12

ATGGTGTCTTTTCCTATGGAATTTAAGTGGGTTTTCTTGGGTATTTCTCTAATGTTGGTTGGTTTGGTTAGCTCCTCAAGATTTGAGGAACTATATCAGCCCAGCTGGGCAACAGACCATTTGACAAATGAAGGAGAAATTCTCAGGATGAAATTGGACAACCTTTCTGGTGCTGGATTTTCATCAAAGAACAAGTATATGTTTGGGAAAGTTACTGTTCAGATTAAGCTTGTAGAGGGTGACTCTGCTGGAACTGTCACTGCTTTCTACATGTCATCAGAGGGACCAACCCAC***AATGAGTTTGATTTTGAGTTTCTAGGC***AACACTACTGGTGAACCATACTCTGTACAAACCAATGTGTACGTAAATGGCGTGGGTAACAGAGAACAAAGATTAAACCTTTGGTTCGACCCATCCAATGAATTCCACTCCTATTCCATCTTGTGGAACCAACACCGAGTTGTATTTTTAGTAGATGAAACACCAGTTCGTGTGCATTCGAATTTGGAGCACAAGGGAATCCCATTTCCAAAGGACCAAGCCATGGGTGTGTACAGTTCAATATGGAATGCAGATGATTGGGCTACACAAGGCGGAAGGGTCAAGACTGATTGGTCACATGCACCCTTTATTGCATCCTACAGAGGATTTGAGATTGATGGCTGTGAATGTCCAGCAACTGTTGCAGCTGCTGAGAATTCTAAGCGGTGCAGCAGCAGTGCTGAGAAAAGGTATTGGTGGGACGAACCAACAATGTCTGAGCTGAGTCTGCACCAGAGCCATCAGTTGATTTGGGTCAGGGCTAACCATATGGTCTATGATTATTGCACAGACACTGCTAGGTTCCCTGTTGCTCCGGTTGAGTGCCAGCACCACCAGCACAAGACTCGCAACTAG

# >NtXTH13

ATGGTGTCTTTTCCTATGGAATTTAAGTGTGTTTTCTTGGGTATTTCTCTAATTATGGTGGGTTTGGTTAGCTCCTCAAGATTTGAGGAGCTATATCAGCCCAGCTGGGCGACAGACCATTTGACAAATGAAGGAGAAATCCTCAGGATGAAACTTGACAACCTTTCTGGCGCTGGATTTTCATCAAAGAACAAGTATATGTTTGGGAAAGTTACTGTTCAGATTAAGCTTGTAGAGGGTGACTCTGCTGGAACTGTCACTGCTTTCTACATGTCATCAGAGGGACCAACCCAC***AATGAGTTTGATTTTGAGTTTCTCGGT***AACACTACTGGTGAACCCTACTCTGTGCAGACCAATGTGTACGTAAATGGTGTGGGTAACAGAGAGCAACGACTGAACCTTTGGTTCGACCCATCCAAGGAATTCCACTCATATTCCATCTTGTGGAACCAACGCCGAGTTGTATTTTTAGTAGACGACACACCAATTCGTGTGCACTCAAATTTGGAGCACAAGGGAATACCATTTCCCAAGGACCAAGCCATGGGTGTGTACAGTTCAATATGGAATGCTGATGATTGGGCTACACAAGGTGGAAGGGTTAAGACTGATTGGTCACATGCACCCTTTATTGCATCCTACAGAGGATTTGAGATTGATGGCTGTGAATGCCCAGCAACTGTTGCAGCTGCTGAAAATTCTAAGCGGTGCAGCAGCAGTGCGGTGAAAAGGTATTGGTGGGACGAACCCGTTATGTCCGAACTGAGTCTGCACCAGAGCCACCAGCTGATTTGGGTTAGGGCTAACCATATGGTCTATGATTACTGCACAGACACTGCTCGGTTCCCTGTTGCACCGGTTGAGTGCCAGCACCACCAGCACAAGTTTCATAACTAG

# >NtXTH14

ATGCCATCCTCTATGATTGTCTTTTTGATCCTAGCTATGCTACTAAACACAGGAGTTGGTGTCAACTTCGCCGAAGTTTTCGAGTCGAGTTGGGCACCTGACCATATTACTGTGGTAGGAGACCAAGTTATGCTCACCCTTGACAATGCTTCTGGCTGCGGGTTTCAGTCGAAGAACAAATATTTGTTCGGGAAAGCCAGCGTGCAGATCAAACTAGTTGGAGGAGATTCAGCTGGAACAGTCATTGCTTTTTATATGTCTTCGGAGGGAGCTAATCACGACGAATTGGACTTTGAGTTTCTTGG***GAATGTTTCAGGAGAACCATACCTAGT***ACAAACAAATGTGTACGCGAATGGCACCGGAGACAGAGAGCAGAGGCATAGTCTCTGGTTCGATCCAACAACGGACTTTCACACTTACTCTTTCTTCTGGAATCATCATACCATTATCTTTTCAGTTGATGACATTCCTATTAGAGTGTTCCAAAACAAGGAGAACAAAGGCGTGGCATACCCGAAAAATCAAGGCATGGGAATTTATGGATCATTGTGGAATGCAGATGATTGGGCTACACAAGGAGGGAGAGTGAAGACCAACTGGAGCCACTCTCCATTTGTTGCAACATTTCGAGCGTTCGAGATCGACGCTTGTGATTTGTCTGGTGAGGACACAGTTGCTGCCGGTGCAAAATGCGGCAAGTTAGCAGAATGCTGGTGGGATAAGCCAGCTGTGAAGCAGCTGAACAAGAGCAAAAAGCGCCAATTCAAAATGGTTCAATCTAAGCACTTGGTCTATGATTATTGTAAGGATACTGCAAGATTCACTCAAATGCCTAAAGAATGCTTGGACTAG

# >NtXTH15

ATGAGAAGAAAAAGCTGCATGCTGACGACAGTGCCATGGCTGCCACTAAAACATTCTCTTGCTCGCTGGGTTGGTGTCAACTTCACCGAAGTTTTCGAGTCGAGTTGGTCACCTGACCATATTACTGTGGTAGGAGACCAAGTTATGCTCACCCTTGACAATGCTTCTGGCTGCGGGTTTCAGTCGAAGAACAAATATATGTTTGGGAAAGCCAGCGCGCAGATCAAACTAGTTGATGGAGATTCAGCTGGAACAGTCATTGCTTTTTATATGTCATCAGAGGGAGCTAATCAC***GACGAACTGGACTTTGAGTTTCTAGGG***AATGTTTCAGGAGAACCATACCTAGTACAAACAAATGTGTACGCGAATGGCACCGGAGACAGAGAGCAGAGGCATAGTCTTTGGTTCGATCCAACTGCGGATTTCCACACTTACTCTTTCTTTTGGAATCATCATACCATTATCTTTTCGGTTGATGACATTCCTATTAGAGTGTTCAAAAACACAGAGAAAAAAGGCGTGGCATACCCGAAAAATCAAGGCATGGGAGTTTATGGATCGTTGTGGAATGCAGATGACTGGGCTACACAAGGAGGGAGAGTGAAGACCAACTGGAGCCACTCTCCATTTGTTGCAACATTTCGAGCGTTCGAGATTGATGCTTGTGATTTGTCTGGTGAGGACACAGTTGCTGCAGGCGCAAAATGTGGCAAGTTAGCACAATGCTGGTGGGATAAGCCAGCCATGAGGGAGCTGAACAAGAGCAAAAAGCGCCAATTCAAAATGGTTCAATCTAAGCACTTGGTCTATGATTATTGTAAGGATACTGCAAGATTCACTCAAATGCCTAAAGAATGCTTGGACTAG

# >NtXTH16

ATGGGGATGAATATGTTGTTGGTGTGTGTGTTATTTGTCGTAGGAGCAATGGCTGCTGCGCCAAAGAAGCCAATGGATGTACCATTTGGAAGAAACTATGAGAATACTTGGGCTCCTGATCATGTCAAATACTTTAATGGTGGCAGTGAGATCCAGCTCTTCCTTGACAACCGCACTGGTACTGGATTCCAGTCAAAAGGATCTTACCTATTTGGGCACTTTGCTATGCACATAAAGATGGTTGCTGGTGATTCTGCAGGCACTGTCACTGCTTTCTATCTGTCTTCACAAAATAATGAGCATGATGAAATAGACTTTGAGTTTTTGGG***GAACAAAACAGGAGAACCATATGTGGT***ACAAACAAATATATACACAGGAGGGAAAGGTGACAAAGAGCAGAGGATTTACTTATGGTTTGATCCAACCAAAGATTACCACACCTATTCTGTTTTGTGGAATCTCCACCAGATTGTGTTTTTTGTAGATGAGTACCCAATCAGAACATTCAAAAACAGCAAAGATTTAGGTGTCAAATTCCCATTTGATCAACCAATGAAGATATACTCAAGTCTATGGGAAGCAGATGATTGGGCAACAAGAGGTGGACTTGAAAAAATAGATTGGTCAAATGCACCTTTTGTTGCTTCTTACAAAGGATTTCACATAGATGGATGTGAAGCTTCAGTAAATGCAAAATTATGTGCAAATCAAGGCAAAAAATGGTGGGATCAAAAAGAATTTCAAGATTTGGATAAACAACAATGGAGACTTTTACGTAGAGTAAGGGATAAATACACTATTTATAACTATTGCACTGATAAAAAGAGGTTTGCAACTCTGCCAAAAGAGTGCAGGAGGAATAGAGATGTGCCAAGAAAATCATCAAAGAAGTCTCCTTAG

# >NtXTH17

ATGGGGTTCAAATGGATGAATATGTTGTTGTTTTGTGCGTTATTTGTCGTAGGAGCAATGGCTGCTGCACCAAAGAAGCCAATGGATGTACCATTTGGAAGAAACTATGAGAATAGCTGGGCTCCTGATCATGTCAAATACTTTAATGGTGGCAGTGAGATCCAGCTCTTCCTTGACAACCGCACTGGAACTGGCTTCCAATCAAAAGGATCTTACCTATTTGGGCACTTTGCTATGCACATAAAGATGGTTGCTGGTGATTCTGCAGGCACTGTGACTGCTTTCTATTTGTCTTCACAAAATAATGAGCATGATGAAATAGATTTTGAGTTTTTGGG***GAACAAAACAGGAGAGCCATATGTTGT***ACAGACAAATGTATACACAGGAGGGAAAGGTGACAAAGAGCAGAGGATTTATTTATGGTTTGATCCAACCAAAGATTACCACACCTATTCTGTTTTGTGGAATCTCCACCAGATTGTGTTTTTTGTAGATGAGTACCCAATCAGAACGTTCAAGAACAGCAAAGATTTAGGAGTCAAATTTCCATTTGACCAACCAATGAAGATATACTCAAGTCTATGGGAAGCAGATGATTGGGCAACAAGAGGTGGACTTGAAAAAATAGATTGGTCAAATGCACCTTTTGTTGCATCTTACAAAGGATTTCACATAGATGGATGTGAAGCCTCTGTAAATGCAAAATATTGTTCAAATCAAGGCAAGAAATGGTGGGATCAAAAAGAATTTCAAGATTTGGATAAACAACAATGGAGACTTTTACGTAGAGTAAGGGATAAATACACTATTTATAACTATTGCACTGATAAAAAGAGGTTTGCAACTATGCCAAAAGAGTGCAGGAGGAATAGAGATGTGCCTAGAAAATCATCAAAAAAGTCTCCTTAG

# >NtXTH18

ATGGGTCTAAAAGGACTTTTGTTTAGTATTGTTTTGATTAATTTGTCATTACTAGGACTTTGTGGGTATCCCAGAAAACCTGTGGATGTACCCTTTTGGAAAAACTATGAGCCCAGTTGGGCTAGTCACCACATCAAGTACCTCAATGGTGGTTCCACTGCTGATCTTGTTCTTGACAGGTCTTCAGGAGCTGGATTTCAGTCAAAGAAATCATATCTATTTGGGCACTTTAGCATGAAACTGAGGCTTGTTGGTGGAGACTCCGCTGGTGTTGTTACTGCATTTTACCTGTCATCGAATAATGCAGAGCAC***GATGAGATAGATTTTGAATTCTTAGGG***AACAGGACTGGGCAACCATACATTTTGCAGACGAATGTGTTTACGGGAGGAAAAGGAGACAGAGAGCAGAGAATCTATCTTTGGTTTGACCCAACCAAGGGTTACCATTCTTATTCCGTTCTTTGGAATACCTTCCAGATTGTGATCTTTGTGGATGACGTCCCAATACGAGCATTCAAGAACTCGAAAGACCTAGGTGTGAAATTCCCATTCAATCAACCCATGAAAATATACTCAAGCCTTTGGGATGCAGATGATTGGGCCACAAGAGGTGGATTGGAGAAAACAGACTGGTCAAATGCCCCATTTACTGCCTCCTACACATCATTCCACGTGGACGGCTGTGAAGCTGCCACCCCACAAGAAGTCCAAGTTTGTAACACCAAAGGCATGAGATGGTGGGATCAAAAGGCTTTCCAAGATTTAGATGCTTTGCAATATAGGAGACTTCGTTGGGTTCGTCAAAAATACACTATCTATAACTATTGCACTGATAGGAAGAGATACCCTACTCTTCCACCAGAATGCACTAAGGACAGAGATATTTAA

# >NtXTH19

ATGGGTGTAAAAGGACTTTTGTTTAGTATTGTTTTGATTAATTTGTCATTACTAGGACTTTGTGGGTATCCCAGAAAACCAGTGGATGTACCCTTTTGGAAAAACTATGAGCCCAGTTGGGCTAGTCACCACATCAAGTACCTCAGTGGTGGTTCCACTGTTGATCTTGTTCTTGACAGGTCTTCAGGTGCTGGATTTCAGTCAAAGAAATCATATTTGTTTGGGCACTTTAGCATGAAACTGAAGCTTGTTGGTGGAGACTCAGCTGGCGTTGTCACTGCATTTTACCTGTCATCGAATAATGCAGAGCAC***GATGAGATAGATTTTGAATTCTTAGGG***AACAGGACTGGGCAACCATACATTTTGCAGACAAATGTGTTCACGGGAGGAAAAGGAGACAGAGAGCAGAGAATCTATCTCTGGTTTGACCCAACCAAGGGTTACCATTCTTATTCTGTTCTTTGGAATACCTTCCAGATTGTGATCTTTGTGGATGACGTCCCAATTAGAGCATTCAAGAACTCAAAAGACCTAGGTGTGAAATTTCCATTCAATCAGCCCATGAAAATATACTCAAGCCTTTGGGATGCAGATGATTGGGCCACAAGAGGTGGATTGGAGAAAACAGACTGGTCCAATGCCCCATTTACTGCCTCCTACACATCATTCCACGTGGACGGCTGTGAAGCTGCCACCCCACAAGAAGTCCAAGTTTGTAACACCAAAGGCATGAGATGGTGGGATCAAAAGGCTTTCCAAGATTTAGATGCTTTACAATACAGGAGACTTCGTTGGGTTCGCCAAAAATACACTATTTATAATTATTGTACTGATAGGAAGAGGTACCCTACACTTCCCCCAGAGTGCACTAAGGACAGAGATATTTAA

# >NtXTH20

ATGCAACTCAAACTTGTCCCTGGAAATTCTGCTGGCACTGTCACCACCTTCTTCTTATCTTCACAAGGAGCTGGACAT***GATGAGATTGATTTCGAGTTCTTAGGC***AATGTTTCTGGCCAACCTTACACAGTTCATACCAATGTTTACTCGCAAGGCAAAGGCAACAAAGAACAACAATTCCATTTGTGGTTCGACCCAACTGCTGCATTTCACACTTACTCCATTATCTGGAATGCTCAGAAGATCATTTTCTTGGTAGATAATAGTCCAATCAGAGTATACAACAACCACGAAAGCGCTGGCATTCCATTCCCAAAAAGCCAACCAATGAAAGTGTACTGCAGCTTATGGAATGCAGATGAGTGGGCTACACAAGGAGGTAGAGTCAAGACAGATTGGACACATGCTCCTTTCACTGCATATTACAGAAATTTCAATATTGATGGCTGCGCAGTCACATCCGGCGCCTCTTCGTGTAAGTCCACTGATTCAGCAAACAATGCTAGGCCATGGCAAAATCAAGAACTTGATGCTAAGGGCAGGAATAGGCTACGATGGGTGCAGAGCAGACACATGGTTTACAACTATTGTGCTGATTCTAAGAGGTTTCCTCAAGGCTTTTCTCATGAGTGCAAGCGTTCGAGGTTCCTCTAA

# >NtXTH21

ATGTCGCCTCGTTTCTCTTTCAAAATGTTAATCCTTCCTATAGTCATGGCAAGTCTATGGGCAGCCGCCTCAGCTGGTAATTTTTATAATCTTGCAGATATCACTTGGGGCGAAGGACGTGGTAAAATAACAGAAGGAGGCAGAGGCCTCTCTCTGTCCCTTGACAAATTATCTGGTTCAGGTTTTCAATCCAAGAATGAGTATTTATTCGGAAGATTTGACATGCAACTCAAACTTGTCCCTGGAAATTCTGCTGGCACTGTCACCACCTTCTTTTTATCTTCACAAGGAGCAGGACAT***GATGAGATTGACTTCGAGTTCTTAGGC***AATGTTTCTGGTCAACCTTACACAGTCCACACCAATGTTTACTCGCAAGGCAAAGGCAACAAAGAACAACAATTCCATTTGTGGTTCGACCCAACTGCTGCATTTCACACTTACTCCATCATCTGGAACGCTCAGAAAATCATTTTCTTGGTGGATAATAGTCCAATCAGAGTATACAACAACCACGAAAGCAATGGCATTCCATTCCCAAAAATCCAACCAATGAAAGTGTACTGCAGCTTATGGAATGCAGATGAGTGGGCAACACAAGGAGGTAGAGTCAAGACAGATTGGACACATGTTCCTTTCACTGCTTACTACAGAAACTTCAATATTGATGGCTGCGCAGTTACATCCGGCACCTCTTCGTGTAAGTCCACTGATTCAGCCAACAATGCTAGGCCATGGCAAAATCAAGAACTTGATGCTAAGGGCAGGAATAGGCTACGATGGGTTCAAAGCAGACACATGGTTTACAACTATTGTGCTGATTCTAAGAGGTTTCCTCAAGGCTTTTCTCATGAGTGCAAGCGTTCGAGGTTCCTGTAA

# >NtXTH22

ATGGCTTCTCATTTGTTTCTAATTTCCATTCTAATGGGCAGCCTAGTTGCTGCCTCAGCTAATTTTAATAATCTTGCAGAGATCACTTGGGGCGAAGGACGTGGTAAAATAACAGAAGGAGGCAAGGGCCTCTCCCTGTCCCTTGACAAACTTTCTGGTTCAGGTTTTCAATCCAAGAATGAATATCTCTTTGGAAGATTTGACATGCAACTCAAACTCGTTCCTGGAAACTCTGCTGGCACTGTCACCACCTTCTTTTTATCTTCACAAGGAGAAGGACAT***GATGAGATCGATTTCGAGTTCTTGGGT***AATACGACGGGCGAGCCCTACACTGTCCATACCAACGTCTATTCTCAAGGAAAGGGAAACAAAGAACAACAATTCCACCTTTGGTTCGATCCAACTGCAGCATTTCACACTTACACCATTGTGTGGAATTCTAACCGCATAGTGTTCTTGGTGGATAACATTCCAATTAGAGTATACAACAACCATGAAAACAATGGCATTCCATTCCCAAAGAGCCAACCAATGAAAGTGTACTGCAGCTTATGGAATGCAGATGAGTGGGCTACACAAGGAGGCAGAGTCAAGACTGATTGGACACATGCTCCTTTCACAGCTTACTACAGAAACTTCAAAATAGATGGCTGCGCAGTCACATCCGGCGCCTCTTCATGTAAGTCCACTGATTCTGCAGGCAATGCTAAGGCATGGCAAAATCAAGAACTTGATGCTAAGGGCAGGAATAGAGTCCGATGGGTGCAAAGTAGACACATGGTTTACAACTACTGCGCTGATAAAAAGAGGTTTCCTCAAGGCTATTCTCATGAATGCAAGAGCTCAAGGTTTTAA

# >NtXTH23

ATGGCTTCTCATTTTCTTCTGATTTCCATTCTAATGGGCAGCCTAGTCGTTGCATCAGCTAATTTTAATAATCTTGCAGAGATTACTTGGGGCGAAGGACGTGGTAAAATAACAGAAGGAGGCAAAGGTCTCTCTCTGTCCCTTGACAAACTTTCTGGCTCAGGTTTTCAATCCAAGAATGAGTATTTATTCGGGAGATTTGACATGCAACTCAAACTTGTACCTGGAAACTCTGCTGGCACTGTCACCACCTTCTTTTTATCTTCACAAGGAAAAGGACAT***GATGAGATTGATTTCGAGTTCTTGGGT***AATACGACTGGCGAGCCCTACACTGTCCACACCAACGTGTATTCTCAAGGAAAGGGAAACAAAGAACAACAATTCCACCTTTGGTTCGACCCAACTGCAGCATTTCACACCTACACCATTGTGTGGAACGCTAACCGCATACTGTTCTTGGTAGATAACATCCCAATTAGAGTGTACAACAACCATGAAAGCAATGGCATTCCATTCCCAAAGAGCCAACCAATGAAAGTGTACTGCAGCTTATGGAATGCAGATGAGTGGGCTACACAAGGAGGCAGAGTCAAGACTGACTGGACACATGCTCCTTTCACTGCTTACTACAGAAACTTCAAAATTGATGGTTGCGCAGTCACATCGGGGGCCTCTTCATGTAAGTCCACTGATTCTGCAGGCAATGCTAAGGCATGGCAAAATCATGAACTTGATGCTAAGGGCAGGAATAGGGTCCGATGGGTGCAGAGCAGACACATGGTTTACAACTACTGTGCTGATAAAAAGAGGTTTCCTCAAGGCTATTCTCATGAGTGCAAGAGCTCAAGGTTTTAA

# >NtXTH24

ATGGCTTCTAAATTTTCATCAGTAATGCTTCTGCTTTGCATAATAATGAGCATACAATTATTAGCAGCCTCAGCTGGTAACTTCTACAGAGATGCTGTAATTACTTGGGGTGAAGGACGTGGCAAAATACAAGAAGGTGGCAGAGGTCTTGCCCTCACTCTTGACAAATTATCAGGCTCTGGTTTTCAGTCCAAGAATGAATATTTATTTGGAAGATTTGACATGCAACTCAAGCTTGTACCTGGAAATTCCGCTGGCACTGTCACCACTTTCTTTTTATCTTCACAAGGAGAAGGACAT***GATGAGATTGACTTTGAGTTCTTGGGC***AATGTTTCTGGACAGCCTTACACTGTCCATACCAATGTTTATACACAAGGAAAAGGAAACAAAGAACAACAATTCCACCTTTGGTTCGACCCAACTGCCGCATTTCACACTTACACCATTGTCTGGAACCCTCACCGCATAGTGTTTTTAGTGGACAACAGCCCCATTAGAGTATACAACAACCATGAAAGCATAGGCATTCCATTCCCAAAGAGCCAAGCAATGAGAGTATACTGCAGCTTATGGAATGCAGATGAGTGGGCAACACAAGGAGGCAGAGTCAAAACAGATTGGACACTTGCTCCTTTCACTGCTTACTACAGAAACATCAATATCGATGGTTGTGCAGTGTTATCCGGTACCTCGTCATGTAAATCCAGCAATTCAGCAAACAATGCTAAGCCATGGCAAACTCATGAACTTGATGGAAAGGGAAGGAATAGACTAAGATGGGTGCAAAGCAGACACATGGTTTATAATTATTGTGCTGATTCTAAGAGGTTTCCTCAAGGTTTTTCAGCTGAGTGCAAGAGTTCAAGATTTTAG

# >NtXTH25

ATGGCTTCTAAATTTTCATCAGCAATGCTTCTGCTTTGTATACTAATGAGCATCCAATTATTAGCAGCCTCAGCTGGTAACTTCTACAGAGATACTGTGATTACTTGGGGCGAAGGACGTGGTAAAATACAAGAAGGTGGCAGAGGTCTCGCTCTCACTCTTGATAAACTTTCAGGCTCTGGTTTCCAGTCCAAGAATGAATACTTATTCGGAAGATTTGATATGCAACTCAAGCTTGTGCCTGGAAACTCTGCTGGCACTGTCACCACTTTCTTTTTATCTTCGCAAGGAGAAGGACAT***GATGAGATTGATTTTGAGTTCTTGGGT***AATGTTTCTGGCCAGCCTTACACTGTCCATACCAATGTTTATACACAAGGAAAAGGAAACAAAGAACAACAATTCCACCTTTGGTTCGATCCTACTGCTGCATTTCACACTTACACCATTGTCTGGAACCCTCACCGCATAGTGTTCTTAGTGGATAACAGCCCCATTAGAGTATACAACAACCATGAAAACATTGGCATTCCATTCCCAAAGAGCCAAGCAATGAGAGTATACTGCAGCTTATGGAATGCAGATGAGTGGGCTACACAAGGAGGCAGAGTCAAGACAGATTGGACACTTGCTCCTTTCACTGCCTATTACCGAAACATCAATATTGATGGTTGTGCAGTGTTATCCGGTACCTCGTCGTGTAAGTCTAGCAATTCAGCAAACAATGCTAAGCCATGGCAAACTCATGAACTTGATGGTAAGGGAAGGAATAGGCTAAGATGGGTACAAAGCAGACACATGGTTTATAACTATTGTGCTGATTCTAAGAGGTTTCCTCAAGGTTTTTCTGAAGAGTGCAAGCGTTCAAGGTTTTAG

# >NtXTH26

ATGTCATTATCCTCTGCTTCCTCCAGAATTCCAAAAATGTTCCTTCAGCTCTCTGTTCTTGCAGTTTTCCTCCTATGCACTGCTTGTGCTGATAATTTCTACCAAGACGCGACTGTCACCTGGGGTGACCAGCGGGCTCACATACAAGAAGGTGGCCGTCTTCTAACCTTGTCTCTCGATAAAATTTCAGGCTCTGGCTTTCAATCCAAGAGTGAGTTTTTATTCGGAAGGTTCGACATGCAGCTCAAGTTAATACCTGGAAATTCTGCTGGCACTGTCACCACTTTCTACTTGTCGTCTCAAGGAGCAGGGCAC***GACGAAATTGATTTTGAATTTCTGGGA***AATTCATCAGGCCAGCCTTACACAGTTCACACCAACGTTTATTCTCAGGGAAAAGGCAACAAAGAACAACAATTTCACCTCTGGTTCGATCCCACCACATCGTTTCACACCTACTCTATCATTTGGAACGCTCAACGCATCATATTTTTGGTGGATAACATACCAATAAGAGTGTACAACAATCACGAAGCACTTGGGGTTGCATTTCCAAAGAATCAAGCAATGAGAGTGTACGCTAGCCTATGGAATGCTGATGACTGGGCAACACAAGGCGGGCGAGTGAAAACGGACTGGTCCATGGCTCCATTCACAGCTTCTTACAGGAATTTCAATACAAATGCTTGTGTTTGGTCAGCGGCATCATCTACTTCATCTTGTGGAGGCTCTAAATCCACTGATTCAGCGAATAATGATCAGACATGGCAAACTCAAGAACTGGACGCTAATGGCAGAAATAGGCTTAGATGGGTGCAGCAGAAATACATGACATACAATTACTGTACAGATGCTCAAAGGTTCAATCAAGTCATTCCTCCTGAATGCAAGCGTTCAAGGTTTTAA

# >NtXTH27

ATGGGGTCAAGAATTTTCTTGGTTCTAGCACTTGTGTTTAGTTCTTGCATGGTTTCTTATGGTGGAAATTTCTTTCAAGAATTTGACTTTACTTGGGGTGGAAATAGGGCTAAGATTTTCAATGGAGGTCAGCTTATGTCTTTGTCTTTGGACAAAGTTTCTGGCTCTGGTTTTCAATCTAAGAAAGAGTATCTCTTTGGGAGAATTGATATGCAAATCAAACTTGTTGCTGGAAATTCTGCTGGAACTGTCACTACATACTATTTATCTTCTCAGGGACCCACACAT***GATGAAATTGACTTTGAATTCTTGGGA***AATGTTACTGGTGAACCTTATATTCTCCACACAAACATTTATGCCCAAGGCAAAGGAAACAAAGAGCAGCAATTTTACCTTTGGTTTGATCCTACCAAGAACTTCCACACCTACTCAATCATATGGAAACCCCAACATATCATTTTCTTGGTCGACAACACACCAATAAGAGTTTACAAGAATGCTGAATCCATTGGTGTGCCATTTCCCAAGAACCAGCCCATGAGAATTTACTCTAGCCTTTGGAATGCTGATGATTGGGCAACAAGAGGAGGCCTAGTGAAAACTGATTGGTCTAAAGCACCATTTACAGCCTACTATAGAAATTTCAATTCTCAAACTTTTAGCAGTTCACAATTTTCAAATGAAAAATGGCAAAATCAAGAACTTGATGCCAATGGCAGAAGAAGACTCAGATGGGTGCAGAGGAATTTCATGATTTATAATTATTGTACTGATTTTAAGAGGTTTCCTCAGGGTTTTCCTCCAGAATGCAAAAGATTTTGA

# >NtXTH28

ATGGCAAGGTTTTCGTCTTCTTCATCTAGGTCCAGGTCTTCTCTTCCATACATTGTATTGCTCTTCGTTGCTGCCCTTTTTGTCTTTAAGATAGATGTTATCATATCTCAGACATTTAGTTCAGCCCGTCGCAACCTGGAGAACACTCCTAACCGTATCTTAGTGAAGTCTAAATCCCAAGAAACTGATGACAGTATACCTGTAGTATTAGTAAATGGTACATTTCACCGGCATTTTATATTATCATGGGGAGACGATAGAGGAAAGATACATGAAAATGGAGAACTTTTAACACTCTCCTTAGATAAGCAATCTGGATCAGGATTTCAGTCCAAGAAAGAGTATCTCTTTGCCAAAATTGATATGCAAATTAAGCTCGTCCCTGGAAATTCAGCTGGCACTGTTACTACTTTTTACCTGTCATCACAAGGCAACAAGCAT***GATGAAATAGATTTTGAATTCTTGGGA***AATTCCACAGGAAATCCTTATACTCTTCATACTAATATTTTCAGTCTAGGCCAAGGCAATAGAGAACAACAATTTTTCTTGTGGTTCGATCCTACTGCAGATTACCATACCTATTCAATCCTTTGGAATCCAAAATGTATTATATTCTATGTTGATGGTACACCAATTAGGGAGTTCAAAAATGCAGAAAAAATTGGTGTTCCATTTCTAAAATACCAACCAATGAGACTATACTCAAGTCTATGGAATGCAGATGATTGGGCTACACAAGGTGGTCGTGTTAAAACTAACTGGAAATTAGCACCTTTTATTGCTTCTTACAAAAATTTTACTTATGAAGCCTGCATTTATTCAAGATTAACTAGTTCGTCTTCGTGCAATATCAACTCTCCTCCTTTTGGTAACAACGCGTGGCTAACACACGAATTGGATCGAAGAAGTCGAGCAAAAATGAAAATTTTGCAGAAAAAACATATGATTTATGATTATTGTAAGGATAAATGGAGGTTTCCTAAAGGACCTGCTCCTGAATGCAAGCTTCAATAA

# >NtXTH29

ATGGCAAGGTTTTCATCTTCTTCATCTAGGTCTAGGTCTTCTCTTCCATACATTATATTGCTCTTCGTTGCTGCCCTTTTTGTCTTTAAGATAGATGTTATCATATCTCAGTCGTTTAGTTCAGCCCGTCGCAACCTGGAGAACACCCCTAATCATATCTTGGTGAAGTCTAAATCCCAAGAAACTGATGACAGTATACCTGTAGTATTAGTAAATGGTACATTTCACCGGCATTTTATATTATCATGGGGAGACGATAGAGGAAAGATACATGAAAATGGAGAACTTTTAACACTTTCCTTAGACAAGCTATCTGGATCAGGATTTCAGTCCAAGAAAGAGTATCTCTTTGCCAAAATTGATATGCAAATTAAGCTCGTTCCTGGAAATTCAGCTGGCACTGTTACTACTTTTTACCTATCATCACAAGGAAACAAGCAT***GATGAAATAGACTTTGAATTCTTGGGA***AATTCAACAGGAAATCCTTATACTCTTCATACAAATATTTTCAGTTTAGGCCAAGGCAATAGAGAACAACAATTTTTCTTGTGGTTTGATCCTACTGCAGATTACCATACCTATTCAATCCTTTGGAATCCAAAATGTATTATATTCTATGTTGATGGTACACCAATTAGGGAATACAAAAATGCAGAAAAAATTGGTGTTCCATTTCCAAAATACCAACCAATGAGACTATACTCAAGTCTATGGAATGCAGATGATTGGGCTACACAAGGTGGTCGTATTAAAACTAATTGGAAATTAGCACCTTTTATTGCTTCTTACAAAAATTTTACTTATGATGCTTGCATTTATTCAAGATTAACTAGTTCATCTTCGTGCAATATCAACTCTCCTCCTTTTGGTAATGACTCGTGGCTAACGCACGAATTGGATCGAAGAAGTCGAGCAAAAATGAAAATTTTGCAGAAAAAACATATGATTTATGATTATTGTAATGATAAATGGAGGTTTCCTAAAGGACCTGCGCCTGAATGCAAGCTTCAATAA

# >NtXTH30

ATGATGAAAACTTCAAGTTGTATGTTTTCTTTCTTGTTTCTGAGTTTCTTGGTGTTGGTGGCTTTGGCAGAAAATTTCAACCAAGAATTTGATGTTACATGGGGTGATGGCAGGGTAAAAATACTTGAAAATGGGCAGCTTCTCACCCTTTCCCTTGACAAAACTTCAGGCTCTGGATTTAGGTCAAAAAGACAATATATGTTTGGAAAGATTGACATGAAGATCAAACTTGTTCCTGGCAATTCTGCAGGCACTGTTACTACATACTATTTATCTTCACTTGGACCGACTCAT***GACGAGATTGACTTTGAGTTTCTAGGC***AACCTAAGTGGAGACCCTTATATTCTTCATACAAATGTGTTCGTACAAGGCAAGGGGGAGAGAGAGCAACAGTTTTATCTTTGGTTCGACCCCACTAAGGATTTTCACACCTACTCTATTCTCTGGAATCCTCGAAGCATCATATTTTCAGTAGATGGGACGCCAATTAGGCAATTCAAGAATCTTGAAGCTTCAAGGGGAATACCTTATCCCAAAAATCAACCAATGTGGATATACTCAAGCTTATGGGATGCTGAAGATTGGGCAACAAGAGGAGGACTTGTCAAAACTGATTGGAGCAAAGCCCCTTTCATTGCTTCTTACAGAAATTTTAATGCCCAAGCATGTGTTTGGTCTTCTGGTTCTACTTCTTCTTGCTCCATAAATTCCACAGCCAATTCTTGGATAACTGAATCATTGGATAACTCTGGCCAAGCAAGGATTAAATGGGTGCAAAAGAATTACATGGTCTATAACTACTGCACTGATACTAAACGTTTCCCTCAAGGATTTCCCCTTGAATGCTCTCTAAATTAA

# >NtXTH31

ATGATGAAAACTTCAATTAGTTGTATAATTTCTTTCTTGTTTCTGAGTTTCTTGCTGGTGGTGATGGCGGCTTTGGCTGGAGATTTCAACCAAGAATTTGATGTTACATGGGGTGATGGCAGGGTAAAAATACTTGAAAACGGGCAGCTTCTCACCCTTTCCCTTGACAAAACTTCAGGTTCGGGGTTTAGGTCAAAAAGACAGTATATGTTTGGAAAGATTGACATGAAGATCAAACTTGTTCCTGGCAATTCTGCAGGCACTGTTACTACATACTATTTATCTTCGCTGGGACCGACTCAT***GACGAGATTGACTTTGAGTTCCTTGGC***AACCTAAGTGGAGACCCTTATATTCTTCATACAAATGTGTTCACACAAGGCAAAGGAGACAGAGAGCAACAATTTTATCTTTGGTTCGACCCCACTAAGGATTTTCACACATACTCTATTCTTTGGAATCCTCGAAGCATCATATTTTCAGTAGATGGGACACCAATTAGACAATTCAAGAATCTTGAAACTTCAATGGGAATACCTTATCCAAAAAATCAACCAATGTGGATATACTCAAGCTTATGGGATGCTGAAGATTGGGCAACAAGAGGCGGACTTGTCAAAACCGATTGGAGCCAAGCCCCTTTTGTTGCTTCTTACAGAAATTTTAATGCCCAAGCATGTGTTTGGTCTTCTGGTTCTACTTCTTCCTGCTCCAGAAATTCCACAGCTAATTCTTGGATAACTGAATCATTGGATAACTCTGGCCAAGCAAGGATTAAATGGGTGCAAAAGAATTACATGGTTTATAACTACTGCACTGATATTAAACGTTTCCCTCAAGGATTTCCCCTTGAATGCTCTCTAAATTAA

# >NtXTH32

ATGATGAAATCTTTCTTGTTTCAGATGATGTTTTTGGTGGTGGCTTTTGCTGGAAATTTCAACCAAAATTTTGATATTACATGGGGTGATGGCCGAGCTAAAATACTCGAAAACGGACAACTTCTTACCCTTTCCCTTGATAAAACCTCTGGCTCTGGTTTCCGATCCAAAAATCAGTATTTGTTTGGAAAGATTGATTTGAAAATCAAACTTGTCCCTGGTAATTCTGCTGGCACCGTTACTACATATTATTTATCTTCAATAGGATCAAGTCAT***GACGAGATTGATTTCGAGTTTCTTGGG***AATCTAAGTGGAGATCCCTATATTCTTCACACAAATGTATTCACACAAGGGAAGGGAAATAGAGAGCAGCAGTTTTATCTTTGGTTCGACCCTACTAAGTACTTTCATACTTATTCTATTCTTTGGAATCCTCAGAGCATCATCTTTTCAGTAGATGGGACACCAATTAGGCAATTCAAGAATTTAGAAGCAAGTGGGATACCTTATCCAAAGAACCAACCAATGTGGATATACTCAAGCTTATGGAATGCAGATGATTGGGCAACAAGAGGAGGATTAGTTAAGACTGATTGGAGCAAAGCCCCATTTATAGCTTCTTACAGAAATTACAATGCCCAAGCTTGTGTATGGTCTTCAACTTCTTCTTCTTCCTGCAGCCCCAACAACTCCACAGAAAATTCTTGGCTAAGTGAATCCTTGGATAACACAGGCCAATCTAAGATTAAATGGGTGCAAAATAATTACATGATTTATAATTATTGCACTGATACTAAACGCTTCCCTCAAGGATTTCCTCCTGAATGTTCTCTCAATTAG

# >NtXTH33

ATGATGAAATCTTTCTTGTTTCTGATGATATTTTTGGTGGTGGCTTTGGCTGGAAATTTCAACAAAGATTTTGATATTACATGGGGTGATGGCCGAGCTAAAATACTCGAAAACGGACAACTTCTCACCCTTTCCCTCGATAAAACCTCTGGCTCTGGTTTTCGGTCCAAAAATCAGTATTTGTTTGGAAAGATTGATTTGAAAATCAAACTTGTCCCTGGTAATTCTGCTGGCACCGTTACTACATATTATTTATCTTCAATAGGATCAAGTCAT***GACGAGATTGATTTCGAGTTTCTTGGG***AATCTAAGTGGAGACCCCTATATTCTTCACACAAATGTATTCACACAAGGGAAGGGAAATAGAGAGCAGCAGTTTTATCTTTGGTTCGATCCTACTAAGGACTTTCATACTTATACTATTCTTTGGAATCCTCAGAGCATCATCTTTTCAGTAGATGGGACACCAATTAGGCAATTCAAGAATTTAGAAGCAAGTGGGATACCTTATCCAAAGAATCAACCAATGTGGATATACTCAAGCTTATGGAATGCAGATGATTGGGCAACAAGAGGAGGACTAGTTAAGACTGATTGGAGCAAAGCCCCATTTATAGCTTCCTACAGAAATTACAATGCCCAAGCTTGTGTATGGTCTTCAAGTTCTTCTTCTTCCTGCACCTCTAACAGTTCCACAGGGAATTCTTGGCTAAGTGAATCATTGGATAGCACAGGCCAATCTAGGATTAAATGGGTGCAAAGTAATTATATGATTTATAATTATTGCACCGATACTAAACGCTTCCCGCAAGGATTTCCCCCTGAATGCTCTCTCAATTAG

# >NtXTH34

ATGTCTTCTTTTTCTTCTAAATTAGTACTAGCTCTTATTGTTAGTGCTTTCGCTATTGCAATTGCGGGTACTATTGACGAAAATTTTGAAATTACATGGGGTGAAGGCAGAGCAAAGATGCTAAATAATGGAGAGCTTCTAACTCTATCACTTGACAAAATCTCAGGCTCAGGATTTCAATCCAAGAATGAATATCTCTTTGGTAAAATAGACATGCAACTCAAACTTGTCCCTGGAAATTCTGCTGGCACTGTCACTGCTTACTATTTGTCATCACAAGGACCAACACAT***GATGAAATAGATTTTGAATTCTTGGGA***AATCTAAGTGGTGATCCTTATACACTTCACACTAATGTATTTAGCCAAGGCAAAGGCAACAGAGAGCAACAATTCCATCTTTGGTTTGACCCTACTGCTGATTTCCACACTTATTCCATCCTCTGGAATCCACAACGCATCATATTTTATGTAGATGGAACACCAATTAGAGAATACAAGAATGCAGAATCAATTGGAGTATCATATCCAAAGAAGCAACCAATGAGAATATACTCAAGTCTATGGAATGCAGATGATTGGGCTACAAGAGGAGGACTTATTAAAACTGATTGGAGTAAAGCACCCTTTAGTGCTTCCTACAGAAACTTCAAATCTGCAACTTCAACCTCTGCAGCCACTAGCAATTCATGGTTGAATGAAGAGTTGGATAATACAAGTCAAGAAAGGCTGAAATGGGTGCAGAAAAATTATATGGTTTACAATTACTGCAATGATTCCAAGAGATTTCCACAGGGATTTCCTGCAGATTGTGCTATGTAA

# >NtXTH35

ATGGCTTCTTTGTTAGCTCAATATTTGGTTTTTCTTGCCTTATGCTCTTTGCAATATCATAGTTTGGCTTATAATAACTTTAATCAAGATTTTGATGTTACATGGGGAGATGGTAGGGCAAAGGTTCTCAACAATGGAAAACTTCTTACTCTCTCCCTTGACAAAGCCTCTGGTTCCGGTATTCAATCCAAGAGAGAGTATTTATTTGGAAGGATTGATATGCAGTTGAAACTCGTACGTGGAAATTCAGCTGGCACTGTTACTACATATTACTTATCATCACAAGGGGCAACACAT***GATGAGATAGATTTTGAATTCTTGGGC***AATCTTAGTGGTGATCCTTATATTATTCATACAAATGTTTACACTCAAGGCAAAGGTGACAAAGAACAGCAATTCTACTTATGGTTTGATCCAACTGCTGGTTTTCATACCTACTCCATTCTTTGGAACCCACAAACAATTATATTTTATGTGGATGGCACACCTATAAGAGTGTTCAAGAACATGAAGTCAAGAGGAATACCATACCCAAACAAGCAACCAATGAGAGTATATGCAAGTCTATGGAATGCAGATGATTGGGCTACTAGGGGTGGCCTAATTAAAACAGATTGGTCCAATGCTCCATTTATAGCCTCTTTTAGAAATTTCAAAGCCAATGCTTGTGTTTGGGAATTTGGAAAATCATCATGTAATAGTAGCACAAATCCATGGTTTTTTCAAGAACTTGATTCAACAAGCCAAGCTAAGTTACAATGGGTGCAGAAAAATTATATGGTTTATAATTATTGTACTGATATTAAAAGGTTTCCTCAAGGTTTTCCTCTAGAATGTAATTTCAACTCCACAACTAGTTAA

# >NtXTH36

ATGGCTTCTTTGTTAGTTCAATGTTTGAATTTTCTTGCCTTATGCTCTTTGCAATATCATATCTTGGCTTCTAGTAATTTTAATCAAGATTTTGATGTTACATGGGGAGATGGTAGGGCAAAGGTTCTCAACAATGGAAAACTTCTTACCCTCTCTCTTGACAAAGCCTCTGGTTCTGGTATTCAATCCAAGAGAGAGTATTTATTTGGAAGGATCGATATGCAGTTGAAACTCGTACGTGAAAACTCAGCCGGCACAGTTACTACATATTATTTATCATCACAAGGGGCAACACAC***GATGAGATAGATTTCGAATTCTTGGGA***AATCTTAGTGGTGATCCATATATTATTCATACAAATGTTTACACTCAAGGCAAAGGTGACAAAGAACAACAGTTCTACTTATGGTTTGATCCCACTGCTGGTTTTCATACCTACTCCATTCTTTGGAACCCCCAAACAATTATATTTTATGTGGATGGTACACCAATAAGAGTGTTCAAGAACATGAAGTCAAGTGGGGTACCCTACCCAACCAACCAACCTATGAGGGTATATGCAAGTCTATGGAATGCAGATGATTGGGCTACTAGGGGTGGCCTTATTAAAACAGATTGGTCCAAAGCTCCATTTATAGCTTCTTTTAGAAATTTCAAAGCCAATGCTTGTGTTTGGGAATTTGGAAAATCATCATGCAATAGTAGCACAAATTCCACAAAGCCATGGTTTTTTCAAGAACTTGATTCCACAAGCCAAGCTAGGTTACAATGGGTGCAGAAAAATTATATGGTTTATAATTATTGTACTGATATTAAAAGGTTTCCTCAAGGTCTTCCTCAAGAATGCAATTTCAACTCCACGACTAGTTAA

# >NtXTH37

ATGGCCAAATTCATAGCTTTTAATTCCTTGGTTTTGATCATTGCAACATTTGCATTTCATTGTGCTATAGTCAATGCAAAGATCTCAAGTAGCATGTATATCAATTGGGGTGCTCATCATTGTCAAATGCTTGGGGATGATCTTCAACTTGTCCTTGATAAATCTGCAGGTTCTGGTGCGCAATCAAAGAGAACATTTCTCTTTGGTAGCTTTGAAATGCTTATCAAGTTGGTACCTAACAACTCCGCTGGAACTGTTACAACATACTATCTATCTTCTACTGGCACCAAACAT***GATGAGATTGGTTTCGAGTTTTTAGGA***AATGTATCAGGACAACCTTACATTATCCACACAAACATTTACACCCAAGGTGTTGGAAACAAGGAGCAGCAATTCTATCCTTGGTTTGATCCAACTGCAGATTTTCACAACTACACCATTCATTGGAATCCTAATGCAGTCGTATGGTATATTGATGGTATTCCAATTAGGGTATTTAGAAATTATCAACTCAAAGGAATTCCATTTCCAAACCAACAAGGAATGAGAATATACTCTAGCCTTTGGAATGCAGATGAATGGGCAACAAGAGGTGGACGTGATAAAATTGATTGGACAAATGCACCATTTATTGCAACATATCGTAAGTTTAGGCCAAGAGCTTGTTATTGGAATGGACCATTGAGTATTGTTCAATGTGCTATTCCTACTAAATCCAATTGGTGGAATTTTCCTTTATACAGTAAATTGAGTGCTCCTAAAGTGGATCAAATGAACTCAATTAGGAGCAAATACATGATTTATGATTATTGCAAAGATACTACACGATTTAAGGGAGTTATGCCTACTGAGTGTACATTGCCACAAAACTAG

# >NtXTH38

ATGGCCAAATTCATAGCTTTTAATTCCTTGGTTTTGATCATTGCTACAATTGCTTTTCATTGTGCTATAGTCAATGGAAAGATTTCAAGTAGCATGTATGTTAATTGGGGTGCTCATCATTGTCAAATGCTAGGGGATGATCTTCAACTTGTCCTTGATAAATCTGCAGGTTCTGGTGCGCAATCAAAAAGAACATTTCTCTTTGGTAGCTTTGAAATGCTTATCAAGTTGGTACCTAACAACTCAGCTGGAACAGTCACAACATACTATTTATCTTCTACGGGTACCAAGCAT***GATGAAATCGACTTCGAGTTTTTAGGA***AATGTATCAGGACAACCTTACATTCTCCACACAAATATTTATACCCAAGGTGTTGGAAATAGGGAGCAACAGTTTTATCCTTGGTTTGATCCAACTGCTGATTTTCACAACTACACCATTCATTGGAACCCTAATGCTGTCGTATGGTATGTTGATGGTATTCCAATTAGGGTATTTAGAAATTATCAATTCAAAGGAATTCCATATCCAAACCAACAAGGAATGAGAATATACTCTAGCCTTTGGAATGCAGATGAATGGGCAACAAGAGGTGGACGTGACAAAATTGATTGGACAAATGCACCATTTATTGCAACATATCGTAAGTTTAGGCCAAGAGCTTGTTATTGGAATGGACCATTGAGTATTGTTCAATGTGCTATTCCTACTAAATCCAATTGGTGGAATTCTCCTTTATACAGTAAATTGAGTGCTCCTAAAGTGGATCAAATGAACTCAATTAGGAGCAAATACATGATTTATGACTATTGCAAAGATACTACACGATTCAAGGGAGTTATGCCTATTGAGTGTTCATTGCCACAATACTAG

# >NtXTH39

ATGGCCAAATTTGTAGCTTTTAATTCCTTGGTTTTGATCATTGCAACAATTGCATTTCATTGTGCTATAGTCAATGGAAAGATCTCAAGTAGCATGTATGTCAATTGGGGTGCTCATCATTGTCAAATGCTAGGGGAAGATCTTCAACTTGTCCTTGATAAATCTGCAGGTTCTGGTGCGCAATCAAAAAGAACATTTCTTTTTGGTAGCTTTGAAATGCTTATCAAGTTGGTACCTAACAACTCTGCTGGAACTGTTACAACATACTATTTATCTTCTACTGGTACCAAGCAT***GATGAAATCGACTTTGAGTTTTTAGGA***AATGTATCGGGACAACCTTACATTCTCCACACAAATATTTATACCCAAGGTGTTGGAAATAGGGAGCAACAATTCTATCCTTGGTTTGATCCAACTGCTGATTTTCACAACTACACCATTCATTGGAACCCCAATGCTGTAGTATGGTATGTAGATAGTATTCCAATTAGGGTATTTAGAAATTATCAACTCAAAGGAATTCCATTTCCAAACCAACAAGGAATGAGAATCTACTCTAGTCTTTGGAATGCTGATGAATGGGCAACAAGAGGTGGCCGTGACAAAATTGATTGGACAAATGCACCATTTATTGCAAAATATCGTAAGTTTAGGCCAAGAGCTTGTTATTGGAATGGACCATTAAGTATTGTCCAATGTGCAATTCCAACAAAATCCAATTGGTGGAATTCTCCTTTATACAGTAAATTGAGTGCTCCTAAAGTGGACCAAATGAACTCAATTAGGAGCAAATACATGATTTATGACTATTGCAAAGATACTACACGATTCAAGGGAGTTACGCCTACTGAATGTTCATTGCCACAAAACTAG

# >NtXTH40

ATGGCCAAATTCATAACTTTTTCCTTGGTTTTGATCATTGCAACATTTGCATTTCGTTGTACTCTAGTCAATGGAAAGATCTCAAGTAGCATGTATATCAATTGGGGTGCTCATCATTGTAAAATGCAAGGGGATGATCTTCAACTTGTCCTTGATAAATCTGCAGGTTCTGGTGCGCAATCAAAAAGAACATTTCTCTTTGGTAGCTTTGAAATGCTTATCAAGTTGGTACCTAACAACTCCGCTGGAACTGTTACAACATACTATCTATCTTCTACTGGCACCAAACAT***GATGAAATCGACTTCGAGTTTTTAGGA***AATGTATCAGGACAACCTTACATTATCCACACAAATATTTACACCCAAGGTGTTGGAAACAAGGAGCAACAATTCTATCCTTGGTTTGATCCAACTGCAGATTTTCACAACTACACCATTCACTGGAATCTCAATGCTGTCGTATGGTACGTAGATGGTATTCCAATTAGGGTATTTAGAAATTATGAGCTCAAAGGAATTCCATTCCCAAACCAACAAGGAATGAGAATCTACTCTAGCCTTTGGAATGCTGATGAATGGGCAACAAGAGGTGGCCGTGATAAAATTGATTGGACAAATGCACCATTTATTGCAACATATCGTAACTTTAGGCCAAGAGCTTGTTATTGGAATGGACCATTGAGTATTGGTCAATGTGCAATTCCCACAAAATCCAATTGGTGGAATTCACCTTTATACAATAAATTGAGTGCTCCTAAAGTGGATCAAATGAACTCAATTAGAAGCAAATACATGATTTATGACTATTGCAAAGATACTAAACGATTCAAGGGAGTTACGCCTACTGAATGTTCATTGCCACAAAACTAG

# >NtXTH41

ATGTTCAAAATTATGGCCAGCTCTCGACTTCTTTCTTTGGCTAATTTGTTCATTTTGGCAATTGCATTTCATTTGGTTTCAGTCAATGGTATGTTCTCAGATAACATGTATATTGGCTGGGGTGCCCATCATTCTTGGATGCAAGGAAATGATCTTCAGCTTGTTCTTGATCAATCCTCAGGTTCAGGTGTACAATCAAAAGGGGCATTTCTTTTTGGAAGCATACAAATGCAAATCAAATTGGTGCCTGGAAACTCTGCTGGAACAGTTACTGCATACTATTTATCCTCTACTGGTGACAAACAC***GACGAGATCGACTTCGAGTTTTTAGGG***AATGTATCAGGGCATCCATATATTATACACACAAATATTTTTACTCAAGGTGCAGGAGGCAGGGAACAACAATTCTATCCATGGTTTGATCCAACTGCTGATTATCATAACTACACCATTCATTGGAACCCCAGTGCAGTTGTATGGTACGTTGACGATATACCAATCAGAGTATACAAGAATTATCAAAGCCAGGGAATTCTCTATCCGAACGCACAAGGAATGGGGGTTTACTCTAGCCTTTGGAACGCCGATAACTGGGCAACTAGAGGCGGCCTTGACAAGATTGACTGGACCAATGCTCCATTTATAGCCAAGTACAGAAATTTCGCGCCACGAGCTTGTCCCTGGTATGGACCAGGTAGCATTAGCCATTGTGCTGCTCCAACTCCAAATAATTGGTATACTTCTCCTGAGTATAGTCAATTGAGCTATGCTAAGCAAGGGCAAATGAATTGGGTAAGGAACAATTACATGATCTATGATTATTGTAAAGATACGACGCGATTCAATGGACAGATTCCTGGAGAATGTTTTAAGCCTCAATTCTAA

# >NtXTH42

ATGTTCAAAATTATGGCCAGCTCTCGACTTCTTTCTTTGTCTAATTTGTTCATTTTGGCAATTGCATTTCATTTGGTTTCAGTCAATGGAATGTTCTCAGATAACATGTATATTAACTGGGGTGCCCATCATTCTTGGATGCAAGGAAATGATCTTCAGCTTGTCCTTGATCAATCCGCAGGTTCGGGTGTACAATCAAAAGGAGCATTTCTTTTTGGAAGCATAGAAATGCAAATAAAATTAGTACCTGGAAATTCTGCTGGAACAGTCACAGCATACTATTTGTCATCTACTGGTGACAAGCAC***GACGAGATCGACTTCGAGTTTTTGGGA***AATGTATCAGGGCAACCATATATTATACACACAAATATTTTTACTCAAGGTGCAGGAGGCAGGGAACAACAATTCTATCCGTGGTTTGATCCAACTGCTGATTACCATAACTATACCATTCATTGGAACCCCAGTGCAGTTGTATGGTACGTTGACGGTATACCAATCAGAGTATACAAGAATTATCAGAGCCAGGGAATTCTCTATCCGAACGCACAAGGAATGAAGGTTTACTCTAGCCTTTGGAACGCCGATAACTGGGCAACCAGAGGCGGCCTTGACAAGATTGACTGGACCAATGCTCCATTTATAGCCAAGTACAGAAATTTCGCGCCGCGAGCTTGTCCCTGGTATGGACCAGGTAGCATTCGCCAATGTGCTGCTCCAACTCCAAATAATTGGTATACTTCTTATGAGTATAGTCAATTGAGCTATGCTAAGCAAGGGCAAATGAATTGGGTAAGGAACAATTACATGATCTATGATTATTGTAAAGATAAGACGCGATTCAATGGACAGATTCCAGGAGAATGTTTTAAGCCTCAAATCTAA

# >NtXTH43

ATGGCAATCTTTTTTCTCCATTTTCTTCTCTTGCTCATTGTTGTCCCTTCTACAAATGCTGGTTATTGGCCACCTTCTCCTGGCTATTATCCAAGTTCCAAGTTTAGGTCTATGAGCTTTTATCAAGGATTTAGAAACCTTTGGGGCCCTAATCATCAAAATGTAGATAATAATGGCATTAATATTTGGCTTGATAGAAATTCAGGAAGTGGATTCAAGTCAATTAAACCATTTCGATCAGGGTATTTTGGTGCTTCCATTAAACTCCAACCTGGTTATACTGCTGGTGTTATTACAGCTTTTTACCTTTCAAATAATGAAGCTCATCCAGGGTACCAT***GATGAAGTGGACATAGAATTTCTTGGA***ACAACATTTGGGAAGCCTTATACATTGCAAACCAATGTTTATATTAGAGGAAGCGGAGATGGGAAAATTGTAGGAAGAGAAATGAAGTTTCATTTGTGGTTTGATCCAACAAAGGAATTTCATCACTATGCTATTTTGTGGAGTCCTAGAGAGATCATATTTCTTGTGGATGATGTGCCAATAAGGAGGTATGCTAGGAAGAGTATTGCAACATTTCCACTAAGGCCAATGTGGTTATATGGATCAATATGGGATGCATCTTCTTGGGCAACTGAGGATGGAAAATACAAAGCCGATTATAGGTACCAACCATTCTACGGGAAATTCACGAACTTTAAGGCAAGCGGTTGCACCGCCTATTCATCGCGATGGTGCCACCCCGTGTCCGCTTCACCATCCAGGTCCGGAGGCCTTACCAGGCAACAACGTCAAGCCATGAATTGGGTTCATAGTCACTACTTGGCTTATGACTATTGTCGAGACTCCAAAAGAGACCATTCCCTAACACCGGAATGCTGGCGTTAA

# >NtXTH44

ATGTCAATCTTTTTCCTCCCTTTTCTTCTCTTCCTCATTGTTCTCCCTTCTACAAATGCTGGTTATTGGCCACCTTCTCCTGGCTATTATCCAAGTTCCAAGTTTAAGTCCATGAGTTTCTATCAAGGTTTTAAAAACCTTTGGGGTCCTAATCATCAAAATGTAGATAATAATGGCATTAATATTTGGCTTGATAGAAATTCAGGAAGTGGATTCAAGTCAATTAAACCATTTCGATCCGGGTATTTTGGTGCTTCTATTAAACTTCAACCTGGTTACACTGCTGGTGTTATTACAGCTTTCTACCTTTCAAATAATGAAGCCCATCCAGGGTACCAT***GATGAAGTGGACATAGAATTTCTTGGA***ACAACATTTGGGAAGCCTTACACATTGCAAACCAATGTTTATATTAGAGGAAGTGGAGATGGTAAAATTATAGGAAGAGAAATGAAGTTTCATTTGTGGTTTGATCCAACAAAGGATTTTCATCACTATGCTATTTTGTGGAGTCCTAGAGAGATCATATTTCTTGTGGATGATGTGCCAATAAGGAGGTATGCTAGGAAGAGTATTGCAACATTTCCACTAAGGCCAATGTGGTTATATGGATCAATATGGGATGCATCTTCTTGGGCAACTGAGGATGGAAAATACAAAGCCGATTATAGGTACCAACCATTCTACGGGAAATTCACGAATTTTAAGGCAAGCGGTTGCACCGCCTATTCATCGCGATGGTGCCACCCTGTGTCCGCTTCGCCATCCCGGTCCGGAGGCCTTACTAGGCAACAACGTCAAGCCATGAATTGGGTTCATAGTCACTACTTGGCCTATGACTATTGTCGAGACTCCAAAAGAGACCATTCCCTTACACCGGAATGTTGGCGTTAA

# >NtXTH45

ATGGCTAATTTATTCCTTCTTTCTTTACTTCTCATTTTCTTGTTCAATTCAAGCAATGCTCAGGGTCCCCTTTCTCCTGGCTACTATCCTAGTTCTAAGGTTCAATCGTTAGGGTTTAACCAGGGTTTTAGAAACCTTTGGGGTCCTCAACATCAATCTTTGGACCAAAGTGCTTTAACAATATGGCTTGATAAAACCTCAGGGGGAAGTGGCTTTAAATCTCTGGAAAATTATCGTTCCGGTTATTTTGGCACTTCTGTGAAGCTACAACCTGGTTACACTGCTGGAATTATTACTTCTTTCTATCTTTCAAACAATCAAGATTATCCAGGGAACCATG***ATGAAATTGATATTGAGTTTCTTGGAA***CAACGCCAAATAAGCCTTATACTTTACAAACAAATGTATACATCAGAGGAAGTGGAGATGGAAATATTATTGGGAGAGAAATGAAATTTCACCTTTGGTTTGATCCAACTAAAGCTTACCACAATTATGCTATCCTTTGGGATCCCAATGAGATCATATTTTTTGTCGACGATGTTCCAATCAGAAGATACCCTAGGAAAAATGATGCTACATTTCCACAAAGACCTATGTATGTGTATGGTTCAATTTGGGATGCTTCATCTTGGGCAACAGAGGAAGGAAGAATTAAAGCCGATTATCGGTACCAACCATTCGTCGGAAAATATAACAATTTTAAAATTGCTGGTTGTACAGCTAATGAGAACCCCTGGTGCGGACGCTCGCCCTCCAGCTCTCCGTCTAGAGCTGGTGGGCTGAGCCGCCAACAAATAGCGGCCATGCTATGGGTGCAGAGGAACTATAAGGTGTATGATTATTGTCGGGACCCTAGGAGAGACCATACTCACACTCCTGAGTGTTAG

# >NtXTH46

ATGGCTTTATTCCTTCTTTCTTTGCTTCTCCTTTTCTTGTTCAATTCAAGCAATGCTCAGGGTCCCCCTTCTCCAGGCTACTATCCTAGTTCTAAGGTTCAATCTTTAGGGTTTAGCCAGTGTTTTAGAAACCTTTGGGGTCCTCAACATCAATCTTTGGACCAAAGTGCCTTAACTATATGGCTTGATAAAACCACAGGGGGAAGTGGCTTTAAATCTCTAAAAAATTATCGTTCCGGTTATTTTGGCACTTCTGTGAAGCTACAGCCTGGTTACACTGCTGGAATTATTACTTCTTTCTATCTTTCAAACAATCAAGATTATCCAGGGAACCAT***GATGAAATTGATATTGAGTTTCTTGGA***ACAACGCCAAATAAGCCTTATACTTTACAAACAAATGTATACATCAGAGGAAGTGGAGATGGAAATATTATTGGGAGAGAAATGAAATTTCACCTTTGGTTTGACCCAACTCAAGCTTACCACAATTATGCTATCCTTTGGAATCCCAATGAGATCATATTTTTTGTCGACGATGTTCCAATCAGAAGATACCCTAGGAAAAATGATGCTACATTTCCACAAAGACCTATGTATGTGTATGGTTCAATTTGGGATGCTTCATCTTGGGCAACAGAGGAAGGAAGAATTAAAGCCGATTATCGGTACCAACCATTCATCGGAAAATATAACAATTTTAAAATTGCTGGTTGCACAGCTAACGAGAACCCCTGGTGCGGACGCTCGCCCTCCAGCTCTTCGTCTAGAGCTGGTGGGCTGAGCCGCCAGCAGATGGCGGCCATGCTATGGGTGCAGAGGAACTATAAGGTGTATGATTATTGTCGGGACCCCAGGAGAGACCATACTCACACTCCTGAGTGTTAG

# >NtXTH47

ATGGATTTCTTTCATCATAATAAAACCTTCCTATTATCACAGTTCTTGATTTTCTGCATGATAGTTGTCGTTTCATGTCGAGGTCCAGTCTATAAGCCTCCAGAAGTAGAGAAATTAACTGATCATTTCAGCCGATTATCGGTTAATCAAGGTTATAATGTATTCTTTGGAGGTGCTAATGTTCGTATGACAAACAATGGGTCCAGTGCTGATCTTATCTTAGATAAATCTTCAGGTTCTGGACTGATCTCTAAGGAGAAATACTACTATGGTTTCTTCAATGCTGCTCTAAAACTGCCTGCTCATTTTACATCCGGAGTTGTAATTGCCTTTTATATGTCTAATTCAGATGTGTTCCCACACAACCAT***GATGAAATTGACTTTGAGTTGCTTGGG***CATGATAAGAGAAGAGATTGGGTTCTGCAGACTAATCTATATGGAAATGGAAGTGTTCACACAGGGAGAGAAGAGAAGTTTTACCTCTGGTTTGATCCAACACTGGATTTTCATGACTACACCATCCTCTGGAATAATCATCACATAGTATTTCTTGTGGACAATGTGCCAATAAGAGAGGTAGTTCATAACACAGCTATATCTTCTGTTTACCCATCAAAGCCAATGTCTGTTATAGCAACAATATGGGATGGATCAGAATGGGCAACTCATGGAGGAAAATACCCTGTAAACTACCAATATGCACCATTTGTAACATCAATGAAAGAAGTAGAATTAGAAGGATGTGTAAGACAACAAAATACTTCAGCAACTTCTACATGTTTTAGGAGAAGTACTTCAAGTTTGGATCCTGTTGATGGGGAAGAATTTATGAAATTATCACAACAGCAGATGACAGGGCTGGATTGGGTAAGGAGAAAGCACATGTTCTACTCATATTGTCAAGATACTAATAGATACAAAGTTCTACCACCAGAGTGCACTTCTAATTAA

# >NtXTH48

ATGGAATTCTATCATCAGCACAAAACATGCTTATTTTCAGGATTCTTGATTTTCTGCATGATAGCTGTGGCTTCATCTCTAGGTCCAATCTATACTCCTCCAGAGGCCGAGCGGTTAACTGATCGTTTCAGTAGATTATCCGTTAATCAGGGATATAATGTGTTTTTCGGAGGTGCTAATGTTCGTCTAACCAACAATGGGTCCAATGCTGATCTTATCTTAGATAAATCTTCAGGTTCAGGACTAGTCTCAAGAGACAAATACTACTATGGTTTCTTCAATGCTGCACTAAAGCTGCCTGCAAATTTTACATCAGGAGTGGTAGTTGCTTTTTATCTTTCTAATCAAAATATTTTCCCACACAACCAT***GATGAACTAGATTTTGAACTGCTTGGG***TATGATAAGAGAAGGGATTGGGTTCTACAAACCAATATTTATGGAAATGGAAGTGTCAGCACAGGGAGGGAAGAGAAGTTCTACCTCTGGTTTGATCCAACACAAGATTTCCATGACTACAGTATTCTCTGGAACAATCATCACATTCTATTTCTAGTGGACAATGTGCCAGTAAGAGAGGTTGTCAATAATACTACAATCTCTTCTGTTTACCCATCTAAGCCAATGTCTATTTATGCAACAATATGGGATGGATCACAATGGGCAACTCGTGGAGGGAAATACCCAGTAAATTATACTTACGCCCCGTTTGTAACATCAATAAAAGGAGTAGAGTTAGAAGGGTGTGTAAGCGAGCAAAACGCATCAGCAGCTAGTGCATGTGCTAGGAGAAGCACATCAAGTTTGGATCCTGTTGATGGAGAAGAGTTTGTCAAGCTGTCACAGCAGCAAATGACGGGGTTGGACTGGGCAAGGAGGAAGCATATGTTTTACTCGTATTGCCAAGATACTAGGAGATACAAAGTCCTACCACCAGAGTGCACTGCCACATAA

# >NtXTH49

ATGGAATTCTTTCACCAGCACAACACACTCTTATTATCAGAGTTCTTGATTTTCTGCATGATATCTGTGGCTTCATCTCTAGGTCCAATCTATACTCCTCCAGAGGTTGAGCGGTTAACTGATCGTTTCAGTAGATTATCCGTTAATCAGGGATATAATATGTTTTTTGGAGGTGTTAATGTTCGTCTAACGAACAATGGGTCCAGTGCTGATCTTATCTTAGATAAATCTTCAGGTTCAGGACTAGTCTCAAGAGACAAATACTACTATGGTTTCTTCAATGCTGCACTAAAGCTGCCTGCAAATTTTACATCGGGAGTGGTAGTTGCTTTTTATCTTTCTAATCAAAATATTTTCCCACACGACCAT***GATGAACTAGATTTTGAATTGCTTGGG***TATGATAAGAGAAGGGATTGGGTTCTACAAACCAATAATTATGGAAATGGAAGTGTCAGCACAGGGAGGGAAGGGAAGTTCTACCTCTGGTTTGATCCAACACAAGATTTCCATGACTACACTATTCTCTGGAACAATCATCACATTCTATTTCTGGTGGACAATGTGCCAGTAAGAGAGGTTGTCCATAATACTGCAATCTCTTCTGTTTACCCATCAAAGCCGATGTCCATTTATGTGACAATATGGGATGGATCACAATGGGCAACTCGCAGAGGGAAATACCCAGTAAATTATACTTACGCCCCGTTTGTAACATCAATAAAAGGAGTAGAGTTAGAAGGGTGTGTAAGCGAGCAAAACGGATCAGCAGCTACTGCATGTGCTAGGAGAAGCACATCAAGTTTGGATCCGGTTGATGGAGAAGAATTTGTCAAGCTGTCACAGCAGCAAATGATGGGGTTGGACTGGGCAAGGAGGAAGCATATGTTTTACTCGTATTGCCAAGATACTAGGAGATACAAAGTCCTACCACCAGAGTGCACTGCCACATAA

# >NtXTH50

ATGGATTATCGAGTTCTTTCATCTCTATCAAAATCGTTGACACCCTTCTCTCTCCTTATGTTGTTATATATTTTCCCGGCGGCTGAGACGGCAACGGCGACCACCGCAAAGGCTTTTAACCTCTCCACCATCACATTCGAAGAAGGATATTCCCCTCTTTTTAGTGATTTCAATATCGAACGATCTCCTGATGATACAAGCTTTCGTCTCCTCCTTAATCGTTTCTCTGGGTCTGGTGTAATTTCGACAGAATATTACAATTATGGATTTTTTAGCGCTAGTATTAAGCTGCCAGCCATATATACTGCCGGCATCGTTGTCGCATTTTATACGTCAAATGTGGACACATTTGAGAAGAATCAT***GACGAGTTAGACATCGAGTTTCTGGGG***AATGTGAACGGGCAGCCATGGAGATTTCAGACCAACTTGTATGGAAATGGGAGCGTAAGCCGTGGGAGAGAAGAGAGGTATAGAATGTGGTTTGATCCTAGCAACGACTTCCATCACTACAGCATTCTTTGGACCCCCAAAAATATCATATTCTACGTTGATGAGACACCAATAAGAGAAGTAAATCGTAATCCAGCAATGGGAGGGGACTTTCCATCAAAACCAATGTCCTTATATGCCACAATTTGGGATGCATCTTCTTGGGCTACAAATGGCGGCAAGGCTAAAGTTGACTACAAACATGAACCTTTTGCAACTGAGTTCAAAGACTTGGTTCTTGAAGGCTGTATAGTAGATCCCATTGAGCAAATTTCATCTACAAATTGCACTGATAGAATTGCCAGACTGCTTTCTCAAAACTACTCTATCATGACACCCGAAAGGCGAAAATCAATGAAATGGTTTAGAGAAAGATACATGTATTATTCTTATTGTTATGATAATATTAGGTACCCTGTGCCACCTCCAGAATGTGTTATTGTTCAATCAGAAAGAGACTTATTTAAGGATAGTGGAAGGCTTAGGCAGAAGATGAAGTTTGGTGGCAGCCACAGCCACCGGAAACACCGCCCTGGACGGAGCTCTAGGCGGCGGAATAGGGCTGCTGGTGGTGGTTCATCAAAGTCTGGCCAAGCTGCTGCAATGTAA

# >NtXTH51

ATGGATTTCATCAGAAAGAAGATATGTCTGTCTGTCTTCTTGTTTTTCCATGTCTGGTTTAGTACAGCCCTTAATGTCTCCACCATACCTTTTAGCGATGGCTTCAGCCATCTCTTTGGCGAAGGAAACATTCTTCATGCTACTGATGATAAGAGCCTTCAACTTCACCTCAACCAACGCACAGGTTCAGGGTTTAAATCTTCTGACCTCTACAACCATGGTTTCTTCAGTGCTAAGATAAAATTGCCATCAGATTATACTGCAGGAATCGTTGTTGCCTTCTATACGACGAATGGTGATTTATTTACAAAGACACAT***GATGAACTGGATTTTGAGTTTCTGGGA***AATATAAGAGGAAAAGCTTGGAGATTTCAGACAAATATGTATGGAAATGGAAGCACAAGTAGAGGAAGAGAAGAACGATATTATCTTTGGTTCGACCCTTCTAAAGAATTTCATCGTTACAGTATCCTGTGGACCAACAAAAACATCATATTTTATATAGATGATGTTCCAATTAGAGAAATCGTACGTAATGATGCAATGGGAGGAGACTATCCATCAAAGCCAATGGGACTATATGCAACAATATGGGATGCTTCAGATTGGGCTACTTCAGGAGGCAAATATAAAACAAATTACAAATATGCACCATTTATAGCTGAATTCACTGATTTAGTACTAAATGGATGTGCAATGGATCCATTGGAACAAGTAGTAAACAACCCTAGTTGTGATGAGAAAGATGATGAACTTCAAAAGGCAGATTTTTCAAGAATTACACCAAGACAAAGAATGGCTATGAAAAGATTTAGGTCAAAATATATGTATTATTCTTATTGTTACGATTCTTTGAGATACTCAGTGCCACCACCAGAATGCGAGATAGATCCAATTGAACAACAACATTTCAAAGAGACGGGGAGGTTGAAGTTTAACAAGCACCACCATCGCCATCCAAAGAGAACAAAAAGTCAAGTTCTTGATGCTAGGAATTATGGAAATCAAGATGAAGAGTGA

# >NtXTH52

ATGGATTTCATCAGAAAGAAGATATGTCTGTCTGTCTTCTTGTTTTTCCATGTCTGCTTTATTACAGCTGATGCTGCCTTAAATGTCTCTACCATACCTTTTAGCGATGGCTTCAGCCATCTCTTTGGCGAAGGAAACATTCTTCATGCTACTGATGATAAGAGCCTTCAACTTCATCTCAACCAACGCACTGGTTCAGGATTCAAGTCGTCTGACCTCTACACCCATGGTTTCTTCAGTGCTAAGATAAAATTGCCATCAGATTATACTGCAGGGATCGTTGTTGCCTTCTATACGACGAATGGTGATTTATTTACAAAAACACAT***GATGAACTGGATTTTGAGTTTCTGGGA***AATATAAGAGGAAAAGCATGGAGATTTCAGACAAATATGTATGGAAATGGAAGCACAAGTAGAGGAAGAGAAGAACGATATTATCTTTGGTTTGACCCTTCTAAAGAATTTCATCGTTACAGTATCCTGTGGACCATCAAAAACATCATATTTTATATAGATGATGTTCCAATTAGAGAAATTGTACGTAATGATGCAATGGGAGGAGACTATCCATCAAAACCAATGGGATTATATGCAACAATATGGGATGCTTCAGATTGGGCTACTTCAGGAGGCAAATATAAAACAAATTACAAGTATGCACCATTTATAGCTGAATTCACTGATTTAGTATTAAATGGATGTGCAATGGATCCATTGGAACAAGTAGTAAACAACCCTAGTTGTGACGAGAAAGATGATGAACTTCAAAAGGCAGATTTTTCAAGGATTACACCAAGACAAAGAATGGCTATGAAAAGATTTAGGTCAAAATATATGTATTATTCTTATTGTTACGATTCTTTGAGATATTCAGTGCCACCACCAGAATGCGAGATAGATCACGTTGAACAACAACATTTCAAAGAGACGGGGAGGTTGAAATTTAACAAACACGGCCACCATCGTCATGCAAAGAGAACAAGAAGTCAAGTTCTTGATGCTAGGAACCATGGAAATCAGGATGAAGAGTGA

# >NtXTH53

ATGGTGAACTATCATCTTGTTACTTTCATATTTTTCTCTGTTGTTGAATTGGTTTATGGGTCTTCAAGAAATTTGCCAATTTTAGCGTTTGATGAAGGGTACTCCCATCTCTTTGGTGATGATAACGTTATGATCCTTAAAGATGGAAAATCTGCTCATATTTCTCTAGATGAAAGAACAGGGGCTGGATTTGTGTCTCAAGACCTATATCTTCATGGATTCTTCAGTGCTTCTATTAAGCTGCCTGCTGATTACACTGCTGGTGTGGTTGTTGCATTTTATATGTCTAATGTGGACATGTTTGAGAAGAACCAT***GATGAAATTGACTTTGAGTTCTTGGGA***AATATTAGAGGTAAAGACTGGAGAATTCAGACCAATATTTATGGGAATGGTAGCACTAGTGTTGGCAGAGAAGAAAGATATGGACTCTGGTTTGACCCTTCTGAAGATTTCCATCACTACAGTATCCTTTGGACTGAGAATTTCATCATCTTTTATGTAGATAATGTCCCCATAAGAGAGATCAAGAGGACAGAAGCTATGGGTGGGGACTTCCCATCTAAGCCAATGTCTTTGTATGCTACAATATGGGATGGTTCTGGTTGGGCTACCAATGGTGGAAAATACAAAGTCAATTACAAATACGCCCCGTATATTGCCAAGTTCTCTGATTTCGTCCTCCACGGATGCGCAGTTGATCCGATTGAACTATCATCCAAATGTGACACTGCACCAAAAACTGCATCAATCCCTACCGGTATTACCCCTGATCAAAGAAGAAAAATGGAGAAGTTTAGAAAGAAGCAAATGCAGTATTCGTACTGCTATGACAAGACTCGGTACAAGGTCCCTCCACCGGAATGTGTGATCGATCCTAAGGAAGCTGAACGACTCCGAGCCTTTGACCCGGTTACATTTGGCGGATCCCGCCACCATCACGGGAAACAACACCGCCGGAGCAGATCAAGAGCTGAGGGTGATATATCCTTTCTGTAA

# >NtXTH54

ATGGTGAACTATCATCTTGTTATTTTCATATTTTTCTCTGTTGTTGAATTGGTTTATGGGTCTTCAAGAAATTTGCCAATTTTAGCGTTTGATGAAGGCTACTCCCATCTCTTTGGTGATAATAACCTTATGATCCTTAAAGATGGAAAATCTGCTCATATTTCTCTAGATGAAAGAACAGGGGCTGGATTTGTGTCTCAAGACCTATATCTTCATGGATTCTTCAGTGCTTCTATTAAGCTTCCTGCTGATTACACTGCTGGTGTGGTTGTTGCATTTTATATGTCTAATGTGGACATGTTTGAGAAGAACCAT***GATGAAATTGACTTTGAGTTCTTGGGA***AATATTAGAGGCAAAGACTGGAGAATTCAGACCAATATTTATGGGAATGGTAGTACTAGTTTTGGCAGAGAAGAAAGATATGGACTCTGGTTTGACCCTTCTGAAGATTTCCATCACTACAGTATCCTTTGGACTGAGAATTTTATCATCTTTTATGTAGATAATGTCCCCATTAGAGAGATCAAGAGGACAGAAGCTATGGGTGGGGACTTCCCATCTAAGCCAATGTCTTTGTATGCTACAATATGGGATGGTTCTGGTTGGGCTACCAATGGTGGAAAATACAAAGTCAATTACAAATATGCCCCGTATATTGCCAAGTTCTCTGATTTCGTCCTCCACGGATGCGCGGTTGATCCGATTGAATTATCATCCAAATGTGACACTGCACCAAAAACTTCATCAATCCCTACAGGTATTACCCCTGATCAAAGAAGAAAAATGGAGAACTTCAGAAAGAAGCAAATGCAGTATTCTTACTGCTATGACAAGACTCGGTACAAGGTCCCTCCAACGGAATGTGTGATCGATCCTAAGGAAGCTGAACGACTCCGAGTCTTTGACCCCGTTACATTTGGCGGATCCCGCCACCATCATGGGAAACGACATAGCCGGAGCAGATCAAGGGCTGAGGGTGATGTATCCTTTCTGTAA

# >NtXTH55

ATGGTGAATTTTCGTCTGGAAATTTTCATATTATGCTCTTTTCTTGTATTAGTTTGTGGGTCTTCAAAACAGCTCCAAACTTTACCGTTTGACGAAGGGTACTCACAACTCTTTGGCCATGATAATCTTATGGTTCTTGAAGATGGAAAGTCAGTTCATCTTTCTCTAGATGAAAGAACAGGAGCAGGATTTGTGTCTCAAGATCTTTACCTTCATGGCTACTTCAGTGCTTCTATTAAGTTACCAGCAGATTACACTGCTGGAGTGGTTGTTGCATTTTATATGTCTAACGGCGACATGTTTGAGAAGAACCAT***GATGAAATTGACTTTGAGTTCTTGGGA***AATATAAGAGCAAAAAAATGGAGGATTCAAACTAATATATATGGGAATGGTAGCACAAATGTTGGCAGAGAAGAAAGATATGGACTCTGGTTTGATCCCTCTGAAGATTTTCATCAATATAGCATCTTGTGGACTGAGAGCCAGATCATCTTTTATGTAGATAATATCCCCATAAGAGAGATCAAGAGGACAAAAGCAATGGGTGGGGACTTCCCTTCTAAGCCAATGTCTTTGTATGCTACAATATGGGATGGTTCTAGTTGGGCTACCAATGGGGGCAAATACAAAGTCAATTACAAATATGCCCCTTATGTCGCCAAGTTTTCCGACTTTATCCTTCATGGATGTGCAGTTGATCCAATTGAATTGTCACCAAAATGTGACACAACCCCTAATTCTGCATCCATTCCAACTAGTATATCCCCTGATCAAAGAAGAAAAATGGAGAGCTTCCGAAAGAAGTACTTGCAATATTCATACTGCTATGACCGGACTCGATACAATGTACCTCTATCTGAATGTGTAATTGATCCTAAGGAAGCTGACCGTCTCCGAGGCTTTGACCCCGTGACCTTTGGTGGCGTCCAGCGCCATCACAGCAAACGACACCACCAGAGGCAATCGAGGAGGGAAGACACGTCTTCTGAATAG

# >NtXTH56

ATGGTGAATTTTCGTCTGGGAATTTTCATACTATGTTCTTTTCTTGTATTAGTTTCAGGGTCTTCAAAAAAGCTCCAAACGTTACCGTTTGATGAAGGGTACTCGCAACTCTTTGGTCATGATAATCTTATGGTTCTTGAAGATGGAAAATCAGTTCATATTTCTCTTGATGAAAGAACAGGAGCAGGATTTGTGTCTCAAGACCTCTACCTTCATGGCTACTTCAGTGCTTCTATTAAGTTACCTGCAGATTACACTGCTGGAGTGGTTGTTGCATTTTATATGTCTAATGGTGACATGTTTGAGAAGAGCCAT***GATGAAATTGACTTTGAATTCTTGGGA***AATATAAGAGCAAAAAACTGGAGGATTCAAACTAATATATATGGGAATGGTAGCACAAATGTTGGCAGAGAAGAAAGATATGGACTCTGGTTTGATCCTTCTGAAGATTTTCATCAATATACCATCCTCTGGACTGAGAGCCAGATCATCTTTTATGTAGATAATATCCCCATAAGAGAGATCAAAAGGACAAAAGCAATGGGTGGGGACTTCCCTTCTAAGCCAATGTCTTTATATGCTACAATATGGGATGGTTCTAGTTGGGCTACCAATGGGGGCAAATACAAAGTCAATTACAAATATGCCCCTTACGTCGCCAAGTTTTCCGACTTTGTCCTCCACGGATGTGCAGTTGATCCAATTGAATTGTCACCAAAATGTGACACTGCACCTAAGTCTGCATTCGTTCCAACTGGTATATCCCCTGATCAAAGAAGAAAAATGGAGAGCTTCCGAAAGAAGTACTTGCAATATTCGTATTGTTATGACCGGACTCGATACAATGTACCTCTATCTGAATGTGTTATTGATCCTAAGGAAGCGGATCGTCTCCAAGGCTTTGATCCCGTGACCTTTGGTGGCGTCCAGCGTCATCACAGCAAACGACGCCGTCAGAGGCAATCGAGGAGAGAAGACGCGTCTTCTGAATAG
