# Supplementary material for: Genome-Wide Identification and Expression Profiling Analysis of the Xyloglucan Endotransglucosylase/Hydrolase Gene Family in Tobacco (Nicotiana tabacum L.)
Source: Genes (Basel). 2018 May 24;9(6):273. doi: 10.3390/genes9060273 (PMC6027287; doi:10.3390/genes9060273)
Supplement: Supplementary file 1 [file genes-09-00273-s001.zip › Supplementary File 4.docx]

**Supplementary File 4: Genomic sequences of *N. tabacum NtXTHs*.**

# >NtXTH1

ATGAATAACTTCTCTACACTTATTTTCTTTGTCACTGCTTTTATTTATTTGTTTCATATTACATTAGCTTCCATAGTTTCAACAGGAGATTTCAATAAGGATTTTATAGTGCCTTGGTCCCCTAACCATGTAAATACTTCTGCCGATGGCCATACAAGAAGCTTGATATTTGATAAGGAATCTGGTAATTGAAATACAAAATCTCTACATCTCTAACTTTTCTTTTTGTGGCTAAGAACATAATGTTTGATAATATATTTTTTTATATTTCATTTCATTATCTTCAGGTTCAGGGATTGCTTCAAATGATACGTACTTGTTTGGTCAATTCGACATGAAAATTAAGTTGATACCAGGAAATTCCGCAGGCACGGTCGTGGCATTTTATGTATGTATGCACAACCTAATTAAATTCTCGTTCTTTTTAAATTTTAACTTTTAATTCCTCTAAAATGTTGATTAATTTGGAACTATATGCAGTTAACTTCGTATCAACCAAATCGTGACGAGGTAGATTTTGAGTTTCTGGGAAATGTTCCTGGGAAACCTTATACTCTTCAAACGAATGTTTATGTCGATGGGTTGGACGATAGAGAACAGAGAATCAACTTGTGGTTTGATCCAACACAAGACTTCCACACTTATTCTATTCTGTGGAACCTTCACCAAATTGTGTAAGCCTTTAATTTTCTTCTTACAGTTTTGCATCAAGGTAAATCTAGGATTTAAATTCAATCAATTCAACCTTTAGGTTCTAATTCAAAATTATGAGTTCAGAATTTAATATTTATTGAAATTTTAGTAATATAAGTTTAACCGATTCATTATGTACTTCATCCATGATCCATCTCAACCTTATTCTTTTTTCTTAATATCACACACAAACGCATATAAATAACGTTTCAGTCTCAAACTAGTCTCAAACTAGCTGGAGTTGACTGACTATATATGAATAATCTGTGTATAGATTTCGCTCTATTTTGGATTCATTTCATTCTAATATTAAAGGATAAGACTTCTAATAAGGTGATAAAACTCTGTGATTTTTGGGCAGGTTTATGGTTGATCGGGTACCTATTAGAACGTACAGAAACCATGCAGATAAAGGAGCTAAATATCCTCGTTGGCAACCAATGGCACTCCAAATTAGCATATGGAATGGAGAAAGTTGGGCAACAGATGGTGGAAAAACAAAAATTGATTGGTCAAAAGCACCATTTGTAGCCTCTTTAGGAAATTACACAATTGATGCTTGCGTTTGGAAAGGAAATGCAAGATTTTGCAGAGGAGAAAGTGAAAATAATTGGTGGAATAAGGAGAAATTCAGCACTTTGACATGGACTCAAAGAAGGTTGTTCAAATGGGTCAGAAAATATCATTTGACATATGATTATTGCATGGATAATCAACGGTTTCAAAATAATCTTCCCATAGAGTGCTCTCTCCCAAAGTATTAA

# >NtXTH2

ATGAAGTTGAAATTGGTAGGAGGTGACTCTGCTGGTGTTGTCACAGCTTATTATGTAAGTTGCAAGATTTTGAAGTTTTATGCATGAATAAATGTCAGCTCTAACAATGCAAAATTTAAATCTTGTAAGAATGTAAAAAGGATTTAACTTATATATAGTGACTTTATAAAAAATATTTATACGATCAGTAAGTTTTCTGGTTGCAAGAAGTGATCTGTTCTTAATTTTCAGGTTATTAATTCTACTTTTTTTATGGATGATTACCCGTAGTTATATTTTAGTTGACGTGAAAGTTTAAGAATTTTGTTACATTTACTGTTTACAAGTTAAACTCATGTAAAAATGTGTATAATGGGGAATTTTATGTGAGTGCAGATGTGCACAGAAGATGGGGCAGGGCCAACTAGAGATGAGGTAGACTTTGAGTTTTTGGGAAATAGGACAGGGGAACCCTATCTTATTCAGACCAATGTGTACAAAAATGGCACTGGTGGGCGTGAGATGAGGCACGTTCTCTGGTTTGACCCCACTGAGGACTTCCATTCCTATTCCCTTCTTTGGAACTCTCACCAGCTCGTGTAAGTAACTCTTTCTAACTTTTAGGTGTTGCTCGGTGTCTCCTAAAATATAGTTGAGTGTGTCGGATTCTTCACAAATAATGTATTTTTGAAGAATCTAATACGGGTGCGGTAATATTTTTAGAAAGACCGATATTAAAAGATGAAAAGTGTGGTGGTGTACACACTATTTCTACCTGATTCAATTACTTGAGGATTCGTAAGGTTTAACCAGATTCTCCTTGAAACATTTCAACCTACTCGGTATTGACCAAAACATGGTTGCATTTGCTTAGGGCAGAGAGTAATTCAGAATTTTTAATCAATGAATGTGGATTACTATTAGTCAATTACACCTACTAATCCTTTATAGCAATTGGTACGAAGTGCAAACTTAGTAGAGATATTTGCTCTAAAATTACTCAAACATTCATATTTATGTTCAAGCAGCAGGAGTAACATTTTCTATCTAATTGGGCTAGATGAAAATAATTTACAATCAACAGGTTTGACGTACTATTGCACTCACTGTTCATATATATTTATTTCAAACATATTATAAATATATAAAAATTGGGATTACTATATTTTTGGGCCGCCAAAAATGTCTATGTTTTGTATATTAAATATTGAATATACATATAATATACATAAATATACATAATATGCATATCATATATAATCGGTGTATATTTGGAATATCGCCTCCAACTATTTTGAGCCGTCAGGACGAAAACGGAAAAGCCCTAAATAATTTTACAGTTGTTCAACCATATAATATAAATACAATTCGACAACACGCGTTATCAACAAAGTGTGTCTGCCCTTGTTCACAATCAGATTTAATTTACATCATAATTCTGATATTTGCAGGTTTTTCGTGGATGAGGTTCCGATAAGGGTATACAAAAACGCGAATTATACGAACAATTTCTTTCCTAATGAGAAACCAATGTACTTGTTTTCAAGCATATGGAATGCAGATGACTGGGCTACTAGGGGTGGTTTGGAGAAAACAGATTGGAAAAATGCACCATTTGTTTCAACATATAAAGATTTCAGTGTAGATGGTTGCCAATGGGAAGATCCTTTTCCTACTTGTGTTTCAACAACCACTAAAAACTGGTGGGATCAGTACAATTCTTGGCACTTATCAAGTGACCAGAAATTGAATTATGCTTGGGTACAACGAAACCTTGTGATTTATGATTATTGCCAGGATACAAAGAGATATCCAGAAAAGCCTGAGGAATGTTGGTTAAGTCCCTGGGATTAA

# >NtXTH3

ATGGCTAATCTTCTCTTAATTGCAGTTTTAATTGCTATTTATTGTTCACTATCTCAAGCTGAAGTTAAAGGTTCATTTGATGACAACTTTAGTAAAAGTTGTCCTGAATCTCACTTCAAGACTTCTGAAGATGGACAGATCTGGTATCTCTCCTTAGACCACAAAGCAGGTACTTAAAACACAAGAATTCAGTGCTTTTCATTTAGTACACATTTTCATTTCTTTGTGGCATATGGGTAAAAAAAAATATTTGTAGCCTTCGGCCAAAACTATTTATATTCGATAGCCACAAACTTGTATAAAATTTGTATATTTATTGTATATAACATACAGAAATATAGATATATAAAAAAAATATACATTTTTCGGCTATTATTTTGAGAGTGGTTGTACAGTGTTATTTTCTCATTTTGAATCTTCAAGTTCTTGATAGTTAACTTTGATGTATTTCTTCTATACAGGATGTGGATTTATGACAAGGCAGAAATACAGATTTGGTTGGTTTAGCATGAAGTTGAAATTGGTAGGAGGTGACTCTGCTGGTGTTGTCACGGCTTATTATGTAAGTTGCAAGATTCTTGAAGTTGTATGCATGAATAAATGTCAGCTCTAGCAATGCAAAATTTAAATCTCGTAAGAATGTAAAAAGGAATTAACTTATATATATTAACTGTATAAAAAATATTTATACGACGAGTAAGTTTTCTGAAAAGAAATTGAATGGAATTATTACTTTCAAAAAATATTGCTTCAAAACAAAGAAAGAAGGTTTGTTTTCCTACATAATTCTCCGGTTAAACCAATGATTCTCTTCTCCAAGTAATAGAAAGAGTTTTCTCGTTAGACTTCTATCAATGAAATAAAGAACCAGAGTACTTTTAACATGTTGCCACTAGTTTTATATATTATATTTTGAATCTCCTTGACATGACCTAAAAGTGTAGCTAAGTGGTTAAGAGGGTTCAAAACCTTTGTGAGATTGCTGGTTCTATTATTCCTGGCTATAGTCTCTTTTAATTTTTAATTTTTTTTTTAACTTCCTTAGCAGAAATCGCCACTGATTGCAACAAGTGATGTGTTCTTATTTTTCCGGTTACAAATTCTAATTTTTATGGATGATTACCCATAGTTATGTTTTAGTTGACGTGAAAGTTTATGAATTTTGTTACATTTTATTGTTTACAAGTTAAACACGTGTGAAAATGTGTATAATGGGGGATTGTATGTGAGTGCAGATGTGCACAGAAGATGGGGCAGGGCCAACTAGAGATGAGGTAGACTTTGAGTTTTTGGGAAATAGAACAGGGGAACCCTATCTTATTCAGACCAATGTGTACAAAAATGGCACTGGTGGGCGTGAGATGAGGCACGTTCTCTGGTTTGACCCTACTGAGGACTTCCATTCCTATTCTCTTCTTTGGAACTCTCACCAACTCGTGTAAGTATCTCTTTCAACTTTCAAATTATTCTCAACTATATTGTTCGGCCTCGTAAAAAATATCAGTAACATTGTGTGTTAGATCATCTAAAAGTAATACAATTTTGGAGCACACGATATAATACAATAATATTTTTGAAGAATCCGAATAACATAGATTCGTCTTAATAGAAATCACAAACATTAGAAGATGAAAAATATGGAGGGCAAATGTATATGTTTTGTATATTAAATATAAATATACATACAACACGCATACAATATACATAATATACATATCATATATATAATCAGTGCATATATTTCGTATATTTGGGCGGCTGGGCCAAAAATAAAAAAAATCCCTATAAAACATTACAGTCGTTCAACCATATAATATAAATACAATTCGATAGCACGTGTTACCAACAAAGTGTGTCTGCCCTTGTTCACAATTAGACTTAATTAATTAACATCATAATTTTCGTGTGTGCAGGTTTTTCGTGGATGAGGTTCCGATAAGGGTATACAAAAACACGAATTATACGAACAATTTCTTCCCTAATGAGAAGCCAATGTACTTGTTTTCGAGCATATGGAATGCAGATGATTGGGCTACTAGGGGTGGTTTGGAGAAAACAGATTGGAAAAATGCACCATTTGTTTCAACATATAAAGATTTTAGTGTAGATGGTTGCCAATGGGAAGATCCTTTTCCTTCTTGTGTTTCAACCACCACTGAAAACTGGTGGGATCAATACAATTCTTGGCATTTATCAAGTGACCAGAAATTGGATTATGCTTGGGTACAAAGAAACCTTGTGATTTATGATTATTGTCAGGATACAGAGAGATATCCAGAAAAGCCTGAGGAGTGTTGGTTAAGTCCCTGGGATTAA

# >NtXTH4

ATGGAGAGAATGTCTTCTTCAATACCTAAATTCCTTCTAATTATAGCACTAATTACTGTTCTTTTTACATTAACACAAGCTGAAGTACAAGGTTCATTTGATGACAATTTTAGTAAAAGTTGTCCTGAAACACATTTCAAGACTTCTGAAGATGGACAGATCTGGTATCTTTCATTAGATAAGAAAGCAGGTGATTATAAAGCCAAGAATTCAATGTTTTTCAATACCATTTTCATTTATTTTAACTTTTAATCTTCATGTTCTTGCTAATATTGATGAGTTTCTATATATAGGATGTGGATTTATGACCAGGCAAAAATATAGATTTGGGTGGTTTAGTATGAAGTTGAAATTGGTGGGAGGTGACTCTGCCGGTGTTGTCACAGCTTACTATGTAAGTTGCAAGAATTTTGAACTTTTTTATACATGGATTGTCAACTCTACTAATGCAAAAATTAACATGGATATAACTTATATAGAATTTTTTTTACCTCGGTAGTCAAGCATTTCCTTTTGTCTTCATATGTTTGATAGCATTAGTGTTGGTTTGATATCTTGTTAATCTTAGATTGCTATTATTACCTATCGTTTAGTTGTTATATTGGCTTCAGACTTCTTTTTATCATGTCGTTGTTTTTACTTGTTGCAATTACTTCTTTTTATCTTTTCTTTAGCCGATGGTTTATCGGAAACAACTTCCCTGTCCTTCCAGGGTACGGATAAGACTGCATACATATTAGCCTTACCAGACTCCACTTATAGAAATTTACCGGGTTTGTTATTATTGGTAGCAAGTAACATGCTTTATTTTTCAAAGTTACTAATTTTACCTTTTATGGACTGCCAAATGCAACTTTAAAATTCTATGGATCATGAACTCTAGCAATACAACTTAATATGGATATACTAACTCATATGCACTACCAACATAAAGAATGTTTATATTAACCATTTTTTAACCTGTTGTAATATGTAATATACTTTATTTTTGAGGTTACTAATCTAGCGCTCTTTTATGGACGATTATAGTTATCGTTTATGTAGTCTTATAGTATATTGTATTTGTATGGAAATTAAACTCTTTTAATATTGTGGGAATATCTAGCAATGGGTTTAATTGGGTTTTTTTTAACATGATTGTAGATGTGTACAGAAGATGGAGCAGGGCCAACAAGAGATGAATTAGACTTTGAGTTCTTGGGAAATAGAACAGGGGAACCTTATACTATTCAAACCAATGTGTACAAAAATGGGACTGGTAATCGTGAGATGAGACACATTCTTTGGTTTGACCCCACCGAGGACTTTCATACTTATTCCATTCTTTGGAACACCCACCAAATTGTGTAAGTCCCTCTTTCACTTTCTATCAAACTAACCCTCGTATGCATCGGACCACCTTTTTTGTCACAATTTATTTTTACACAATTAATATGTACCGTCAGACACTCTATAACAATATACCTATATAACAGTTATTCATTATAAAAATTATATTTTTTTCGGAATCGATTTTTATATTATATTATAATATATGTCATCTGTAACAACACTTCACTATAACATCAAAAAAAAAATATCGAAACAAACGAAACTGTTATAGAAAGGTTTGAGTGAACCTATCAATGATTAGCTTGTTGAGTTAGATTCAATCCAGCAACTTGCAGACTTTGGACGATGCTATAGTATGTACTTTTATTAGGGCTAGAATATAGAGGAGATCTGACGGAAGTACTAAATTCTGATTGTTCAAGTTAAGGATTAGAATTAATATTGATATCGATACTTATGTGTATGCATGTCAATTCACTTGCTACCTTTGGATAACACTATAATATATATAATTAGGGCTAGATAGAGAAGTTCTTACAGAAGTACAAAATTCTGATTGTTCAAGTTAAGGATTAGAATTTTATTGATATCAATCCTTATGTGTATGCAGGTTTTTCGTGGATAGAGTACCAATAAGGGTATACAAAAATGCGAATTATACGAATAATTTCTTCCCAAATGAGAAGCCAATGTACTTGTTTTCAAGCATATGGAATGCTGACGATTGGGCTACAAGAGGTGGTTTGGAGAAAACAAATTGGAAAAATCAACCATTTGTTTCAAGTTATAAGGATTTTAGTGTAGATGGTTGTCAATGGAAAGATCCATTTCCTGCTTGTGTTTCTACTACCACTAAAAATTGGTGGGATCAATATAATTCTTGGCATTTATCAAGTGACCAAAAAATGGATTATGCTTGGGTACAGAGAAATCTTGTGACTTATGATTATTGCCAAGATACTGAGAGATTTCCTAAAAAGCCTGAGGAATGTTGGTTAAATCCATGGGATTAA

# >NtXTH5

ATGGAGAAAATGGCTTCTTCAATACCTAAAATCCTTCTAATTATAGCACTAATTACTGTTCTTTTTTCATTAACACAAGCTGAAGTACAAGGTTCATTTGATGATAATTTTAGTAAAAGTTGTCCTGAAACACATTTCAAGACTTCTGAAGATGGACAGATCTGGTATCTTTCATTAGATAAAAAAGCAGGTAAATTATAAAGCCAAGAATTCAATGTTTTTCAATACCATTTTCATTTATTTGAACTTTTAATCTTCATTTTCTTGCTAATATTGATGAGTTTCTTTATATAGGATGTGGATTTATGACTAAGCAGAAATATAGATTTGGGTGGTTTAGTATGAAGTTGAAATTGGTGGGAGGTGACTCTGCTGGTGTTGTCACAGCTTATTATGTAAGTTGCAAGAATTTTGAACCTTTTTCACATGGATTGTCAACTCTACTAATGCAAAAATTAACATAGATATAACATATAGAATTTTTTTTACCTCGGTAATCGAGCGTTTTCTTTTTTGTCTTTATTTGTTCAATAGTATTAGTGTTAGTGTGATGTCTTGTTAATCTTAGATTGCTATTATTACCTACCGTTTGGTTGTTATATTGGCTTCGGTCTTCTTTGTATTTTGTTGTTGATTTTACTCATTGCTATTACTTCTTTTCATTTTTTCTTTAGCCAAATATCTATCGGAAATAACTTCTCTATCCTTCTTGGGTAGGGATAAGGCTGCGTATATTTTACCCTCTGCAGACTTCACTTGTAGAAATTTACTAGGTTTGTTGCTTGTTGGTAGCAAGTAACATACTTTATTATCCACCGAGTTTCGAAGGCTGCGGTTGGTCCAAAGAATACGCCCCAGACGGATTTCTCGATCATCAAATTTTTTTTTTTACTAATATCACCTTTTATGGACGGTCATATGCAACTTTAAAATTCTTCTTTGTAAATGGATCATCAACTATAGCAATACAAATTAATATGGATATACTAACTCATATACACTAACAACGTAAAAAATATTTATATTAATCGTTTTTTGACCTGATGTAACATGTAATCTATTTTATTTTCGAGATTACTAATCGTGCTTTTATGGCCGACGGTTATTGTTATAGTTTATGCAGTCTTATAGTAATATATTATCATTGTATAGAAATTAAACTCTTTTAATATTGTGTTAATATCTAGCAATGAGTTTAATTAGGGTTTTTACATGACTGCAGATGTGCACAGAAGATGGAGCAGGACCAACAAGAGATGAATTAGACTTTGAGTTCTTGGGAAATAGAACAGGGGAACCCTATACTATTCAAACCAATGTGTATAAAAATGGGACTGGTAACCGTGAAATGAGACACATTCTATGGTTTGACCCCACTGAGGATTTCCACACTTATTCCATTCTTTGGAACACTCACCAAATTGTGTAAGTCCCTCTTTCACTTTCTATTAAACTATAAACTAAGCGTGCCGACGTGGACTTTTAGTGATTTTTTACATGCATAATATGGAAATTGTCGCAAATTTTTTACACAATCAGTATTATATCGTCAGAATTCTCTTTTAACAGGATTTCTATATAATATTCATTCACTATAAAAATTGTATTTTTCTTAGAATCGATTTTTATATTATGTTATAATATGTCATCTATAACAACATTCCACTATTGTAACCAAACAATATTGGAACAAACAAATAAGTTATAGAGAGGTTTGGCTGAACACGTTGATGATTAGCTTGTTGAGTTAGATTCAAGCTTGCGACATGTTAGTTAACTTGCTGACTTTGGAGTTGGGACAACACAATAATATGTATTTTATTAGGGCTAGAACATAGAGGAGATCTGACAGAAGTACAAAATTCTGCTTGTTCAAGTTAAGGATTAGAATTAACATTGATATCGATCCTTATGTCAATTCACTTGCTAACTTTGGATAACGCTACAATATATACTTAATTAGGGCTAGATAAAAAATTCTGACAGAAGTACAAAATTCTGATTGTTCAAGTTAAGGATTAGAATTATATTAATATCGATCTTTATGTGTATGCAGGTTTTTCGTGGATAGAGTACCGATAAGGGTATACAAAAATGCGAACTATACGAATAATTTCTTCCCAAATGAGAAGCCAATGTACTTATTTTCAAGCATATGGAATGCTGATGATTGGGCAACAAGAGGTGGTTTGGAGAAAACAAATTGGAAAAATCAACCATTTGTTTCAAGTTATAAAGATTTTAGTGTAGATGGTTGTCAATGGAAAGATCCATTTCCTGCTTGTGTTTCGACAACCACTAAAAATTGGTGGGATCAATATAATTCTTGGCATTTATCAAGTGACCAAAAAATGGATTATGCTTGGGTTCAAAGGAACCTTGTGACTTATGATTATTGCCAAGATACAGAGAGATTTCCTAAAAAGCCTGAGGAATGTTGGTTAAATCCATGGGAATAA

# >NtXTH6

ATGGAGAGAAATGCTTCTTCAATGGCTGATCTTTTCTTCACTGCAGCACTAATGGCTGCACTCTTTTCATCCTCACATGCTGAACTCATCAAAGGTGCATTTGAAAACAACTTTAGTAAAAGTTGTCCTGGTACACATTTCAAGACTTCTCAAGATGGACAGATCTGGTATCTCACCTTAGACCAAATATCAGGTGATTAAAAAAAACCCAAGAATTCTAATGCATATCATTTCTTACATTCTTTTTTCTTTTCTTAACATATGAATATTCATGTTCTTGGTATACAGATTGTGGGTTTATTACTAAGCAGAGTTATAGATTTGGTTGGTTTAGCACAAAGTTGAAATTAGTAGGAGGTGACTCTGCTGGTGTTGTGACAGCCTTTTATGTAAGTTCAGAAAATTTGTGCATGAAATGACAAATCAATAGTACTATATACAAATGGGTGTAATGGGGATTTTAATTTATATTGGGTGCAGATGTGTTCGGAAGTGGAGGCAGGGCCATTGAGAGATGAGATAGATTTTGAGTTCTTGGGAAACAGAACAGGACAACCTTATCTTATTCAAACCAATGTGTACAATAATGGCAGTGGTGGACGTGAGATGAGGCATCTTCTTTGGTTTGATCCCACTCAAGACTTCCATACCTATTCCATTCTTTGGAACTCTCACCAAATTGTGTAAGTCCTTTTCTCACTTCTAGGTAGTTTTTGTTTTTTTCTCTCTATTTAACCTTATATTGTCTGTTTTTCACCAATCCCGCAAAGTTTTCCTCAAAAGTCCTCACACTATTAAGAAATCCCTATAGAGTATCTTAATTCTGATGACATCGCTCGGCGGTAAATTTTGTTTCACTAATCACATAAGGGTACCACAATTCTATGGTAACAATTTTTTTGGAAAATGCTTACTTTAGAACAAATTGATTGATGAGAGAACTATCATTTTAAATTACAATTAACGTCAAGAATCTCATGAAAATGGATTTGTACATAACTTGATGGATCTAGAAGATTCAAATAATTAAGCATAATTGGCTTGAGATTGATCAAAATTCTTTTAAATGCAGGTTTTTTGTTGATAAGGTTCCGATAAGGGTATACAAGAACGCGAATCACACGAACAATTTTTTTCCAGCTGAGAGGCCAATGTACGTGTTTTCTAGCATATGGAATGCAGATAATTGGGCTACTAGAGGAGGATTGGACAAGATAAACTGGACAAGTGCACCATTTATAGCAAGTTATAAGGATTTTATTTTAGATGCTTGTCAATGGAAAGATCCTTTCCCTGCTTGTGTTTCCACCACTACACAGCATTGGTGGGATCAATATAATGCTTGGCACCTATCAAGTAAACAGAAGATTGATTATGCTTGGGTGCAGAGAAACTTTGTAGTTTATGATTATTGCCAGGATAGTGTGAGAAACCGTTATAAGCCCCAAGAGTGTTGGTTAAGTGCATTGGACTAA

# >NtXTH7

ATGGAGAGAAATATGGGTGATCTTCTCTTATTTGCAGCACTAGTGGCTACCCTTTTTTCATCATCACATGCTCAACTTATCAAAGGTGCATTTGAAAACACCTTCAGTAAAAGCTGTCCGGGTACTCATTTCAAGACTTCTCAAGATGGACAGATCTGGTATCTCACCTTAGACCAAGTATCAGGTGATTAAATAAACCCCAAGATTTCTAATGCATTTCATTTCTTACATTCTTTTTCTTTTCTTAACATTTGAATATTCATGTTCTTGGTATACACACAGATTGTGGGTTTATCACCAAGCAGAGCTATAGATTTGGTTGGTTTAGCACAAAGTTGAAATTGGTAGGAGGTGACTCTGCTGGTGTTGTGACAGCCTTTTATGTAAGTTCAGAAAATTTGTGCATGAAATAACAAATCAATAGTACTATATACAAATGGGTGTAATGTAATGGGGATTTTAATTTACATTGGGTGCAGATGTGCTCGGAAGTAGAGGCAGGACCATTGAGAGATGAGATAGATTTTGAGTTCTTGGGAAACAGAACAGGGCAGCCTTATCTTATTCAGACCAATGTGTACAATAATGGCAGTGGTGGACGTGAGATGAGGCATCTTCTCTGGTTTGACCCTACTCAGGACTTTCATACCTATTCCATTCTTTGGAACTCCCACCAAATTGTGTAAGTCCCTCTTTTTCTTTCTCCTTATTTTTAATCATATAACATTCAAAAATCACACGGGTTCGATTAATTTGAATTCACGTCGGATAAATTCATTGAGATTGGAGTATCTTAATTGTCCCGACATCCAATGGAGGTAAATTTTATTTCACTAAACACACATGTCTACCACAATTCTATGGTAACAATTTTTGGAAAATGCTTACTTGAGAACAAGCCGAGCGGTCTTTCGTAAATAGTCTCTCGATCTCTTCGGGTAGGGTTAAGGTCTGCATACACACTGAATTGTTGTTGTTGTTGCTTACTTGAGAACAAATTGATTTTGTGAGAACGGTCATTTAAAATTACAATTAACATCAAGATTGTCATGAAAATGGATTTTTATATAACTTGATGGATCTTGAAGATTCAAACAATTGAGCGTTGAGATTAAGGTGCAGTACTGTAGTTGATGATTTGATCAAAATTATTGTAAATGCAGGTTTTTCGTTGATAAGGTTCCAATAAGGGTATACAAGAACGCGAATCACACTAACAATTTTTTTCCAGCTGAGAGGCCAATGTACGTGTTTTCTAGCATATGGAATGCAGATAATTGGGCTACTAGAGGAGGGTTGGACAAGATAAACTGGACAAGTGCACCATTCGTAGCAAGTTATAAGGAGTTTACTTTAGATGCTTGTCAATGGAAAGATCCTTTCCCAGCTTGTGTTTCCACCACTACACAGCACTGGTGGGATCAGTATAATGCTTGGCACCTATCAAGTAAACAGAAGATTGATTATACTTGGGTGCAGAGAAACTTTGTAGTTTATGATTATTGCCAGGATAGTGTAAGAAACCGTTACAAGCCTCAAGAGTGTTGGTTAAGTCCATTGGACTAA

# >NtXTH8

ATGAAGCAAGTAATTGAATATCGTTGCCTTCTGATTTTAGGATGTGGGTTTGCTTCCAAAAGCAAATACCTCTTTGGACGTGTTAGCATGAAGATCAAGCTCGTTCCTGGTGACTCTGCTGGAACTGTCACCGCCTTTTACGTAAGTAGAGTGTACTTAGTTTATGTAATAGCCCTGTCCACTAGTAATATTGTACGATTTGGGCTCAAGCTTACTCAAATTTATACCCAGTATCTCTCTCGTATTTTTATCAATGAGAAATTTACTTGGGGTGTCACAATTTACCCCTAGATTTCATTTCCTGGAAAAGTAAAAGAAAGAGGTTTAATCTAAATATGAACAGAATTTAGTTATATAAATTTCAGTGTAAAATATTTAACTATGGTTTTTAATCACTTAAAGCATTGCATTTTCATGACATCATGTTACCAATTTAAAATCACTATAGATAACGTAATTATAATACGTAAAATAATTATATACTATTAGTGCAAAGAAGTTAAAGCTCGCGGAAATTAATTATTGCATGTGACATTATCATTTGTAGATGAACTCGGACACAGATAACGTAAGGGACGAGCTAGACTTCGAGTTCTTGGGAAACAGGTCAGGCCAGCCGTACACTGTCCAAACGAATGTTTATGTCCATGGAAAGGGTGACAAGGAACAAAGGATCAACCTTTGGTTCGATCCATCCGCTGATTTTCATACCTACACCATTCTTTGGAACCACCATCACACTGTGTAAGCCGTACTTAACAATTATTCTTCCATCGATCTTTTAATTAAAAATCACAAAAAATAATACTATACTGTTTGTACTATTGAAGATTCTACGTGGACGCAGTACCCATTAGAGTGTACAAGAATAACGAAGCAAAAGGAATCCCATTCCCTAAATTCCAACCCATGGGAGTGTACTCAACATTGTGGGAAGCCGACGACTGGGCAACAAGAGGTGGATTAGAGAAAATAAATTGGAGCAAATCCCCATTTTACGCATACTACAAGGACTTTGACATAGAGGGATGTGCAATGCCAGGACCAGCAAACTGTGCCTCAAATCCACGCAATTGGTGGGAAGGTGCTAATTACCAACAGCTCAGTGCTGTGGAAGCAAGGCAATATCGCTGGGTTAGAACGAACCACATGATCTATGATTATTGCACTGACAAATCCAGAAATCCAGTTCCCCCACCAGAATGTGTGGCCGGAATATGA

# >NtXTH9

ATGATTTCCTCTTCTTTAAAATATTCAACTGTCATTCCAATCTTGCTATATGCCTTGACCTTTTCTTCCTCAGTAAGTGCACGACCCGCCACTTTTTTACAGGACTTTAAAGTGGCATGGGCTGACTCTCACATCAAGCAAATCGATGGCGGCAAGGCTATTCAGCTTATACTCGACCAAAACTCAGGATGTGGGTTTGCTTCCAAAAGCAAATACCTCTTTGGACGTGTTAGCATGAAGATCAAGCTCGTTCCTGGTGACTCTGCTGGAACTGTCACTGCCTTTTACGTAAGTAGAGTGTACTTAGTTTATATAACAGCTCAGCCCACTAATGATATTGTACGCTTTGGGCTCAAGCTTACACGACTTTAACCCATTATCTCTCTCATATTTTTGTTAATGAGAGATTTACTTGGGGCGTCACAATTTACCCCAAGATTTCATTTCCTGGAAAAGTAAAAGAAAGAGGTTTAATCTAAATAAGAACAGAATTTAATTATATAAATTTCAGTGTAAAATATTTAACTATGGTTTCTAATCAGTCAAAGCATTGCATTTTCATGTTACTAAGTTAGAATCACTATAGACAGTTACTCATCATTGTAAGCAGTTATAATACGTAAAATAATTATACTCTACCAGTGCAAAGAAGTTAAAGCTCGCGGAAATTAATTATTGCATGTGACATTATCATTTGTAGATGAACTCGGACACAGATAATGTAAGGGACGAGCTAGACTTCGAGTTCTTGGGAAACAGGTCAGGCCAGCCGTACACTGTCCAAACGAATGTTTATGTCCATGGAAAGGGTGACAAGGAACAAAGGATCAACCTTTGGTTCGATCCATCCGCTGATTTTCATACCTACACCATACTTTGGAACCACCATCACACTGTGTAAGCAGACATATAGTACTACTTAACAATTATTCTTCCATCGATCTTTTAATTAAAAATCACAATAAATAATACTTAACTGTTTGTACTATTGAAGATTCTACGTAGACGCAGTACCAATTAGAGTGTACAAGAACAACGAAGCAAAAGGAATCCCATTCCCCAAATTCCAACCCATGGGAGTTTATTCAACATTATGGGAAGCCGACGACTGGGCAACGAGAGGTGGATTAGAGAAAATAAATTGGAGCAGATCCCCATTTTACGCATATTACAAGGACTTTGACATAGAGGGATGTGCAATGCCAGGACCAGCAAACTGTGCCTCAAATCCTCGCAATTGGTGGGAAGGAGCTAATTACCAACAACTCAGTGCTGTGGAAGCAAAGCAATATCGCTGGGTTAGAATGAACCACATGATCTATGATTATTGCACTGACAAATCCAGAAATCCAGTAACCCCACCAGAATGTGTGGCCGGAATATGA

# >NtXTH10

ATGGGCAAATTGACGTCCTTAAAATATTCAGCTGCAATTCTAATATTGCTATATGCCTTGACCTTTTCCTTCTCAGTGAGTGCACGACCCGCCACTTTTCTACAGGACTTTAAGGTCTCTTGGGCCTACTCTCACATCAAACAAATCGATGGCGGCAGGGCCATTCAGCTTATTCTCGACCAAAACTCAG**GC**ACTAATTCATTTTTTTTTGGTTCTGCTTTAGGTTTTATGTCGTACTCTTGCATGAGGTGTAAAACTGATCGTTGATTGATTATTTCTTTCAGGATGTGGGTTTGCTTCCAAAAGCAAATACCTCTTTGGACGTGTTAGCATGAAGATCAAGCTCGTGCCTGGTGACTCTGCTGGAACCGTCACCGCCTTTTACGTAAGCACAGAGTTTGTAAATTTTACACTAGACTTCATGTTCAATAAAAATTCTGCATGACATTTTTTTTTGCAGATGAATTCGGACACAGACAACGTAAGGGACGAGCTAGACTTCGAGTTCTTGGGAAACAGGTCAGGCCAGCCGTACACTGTCCAGACGAATGTTTATGTTCATGGAAAAGGTGACAAGGAACAAAGGGTCAACCTTTGGTTCGATCCATCCGCTGATTTTCACACTTATACCATTCTTTGGAACCACCACCACGCCGTGTAAGTAGACACATCTTGTAAAATCGATAAAAACATGGATTTGAGTTATATATACTGACCATATAGTTTACCTTGTTATAGCATATGACATGTTAAATAGTATCATTTTTCTATCCAAGAAATTTAGTTATGATTAACTTATTTGACCAGATGCAAGTAAGTATTACTGATAAACTGTTTGTACTATTGAAGATTCTACGTGGACGCAGTACCCATTAGAGTGTACAAGAACAACGAAGCAAAAGGAATCCCATTCCCCAAATTCCAACCCATGGGAGTGTATTCCACATTGTGGGAAGCCGATGACTGGGCAACGAGAGGTGGATTAGAGAAAATAAATTGGAGCAAATCCCCATTTTACGCATACTACAAGGACTTTGACATAGAGGGATGTGCAATGCCAGGACCAGCAAACTGTGCCTCAAATCCACGCAATTGGTGGGAAGGAGCTAATTACCAACAACTCAGTGCTGTGGAAGCAAGGCAATATCGCTGGGTTAGAATGAACCACATGATCTATGATTATTGCACTGACAAATCCAGAAATCCAGTCACCCCACCAGAATGTGTGGCCGGAATATGA

# >NtXTH11

ATGGCCAGATTGACTTCCTTAAAATATTCAGCTGCAATTCTAATATTGCTATATGCCTTGACCTTTTCATTCTCAGTGAGTGCACGACCCGCCACTTTTTTACAGGACTTTAAGGTCTCTTGGTCCGACTCTCACATCAAACAAATTGATGGTGGCAGGGCCATTCAGCTTATTCTCGACCAAAACTCAG**GC**ACGAATTCAATTTTTTTTTTCTGGTATAGGTTTAATGTCATACCCATGCATGAGTTATGAAAATGATAATTTACTGATACTTTTATTTCAGGATGTGGGTTTGCTTCCAAAAGCAAATACCTCTTTGGACGTGTTAGCATGAAGATCAAGCTCGTACCTGGTGACTCTGCTGGAACCGTCACTGCCTTTTACGTACGTAAGAACAGAGTTGTTAATTTTATACTAGACTTCATGTTCAATAAAAATTCTGCATGACATTTTTTTGCAGATGAACTCGGACACAGACAACGTAAGGGACGAACTAGACTTCGAGTTCTTGGGAAACCGGTCAGGCCAGCCGTATACTGTCCAGACGAATGTTTATGTTCATGGAAAAGGTGACAAGGAACAAAGAGTCAACCTTTGGTTCGATCCATCCGCTGATTTTCACACTTATACCATTCTTTGGAACCACCACCACGCCGTGTAAGTAGACATATATTGTAAAATTGATAAAAACAAGGATTTAAGTTATATATGGAGTACTAACTATAGAAATTTAGTTATGACTAACTTACTTCACCAGATGCAAGGAAATATTACTGATAAACTGTTTATACTATTGAAGATTCTACGTGGATGCGGTACCCATTAGAGTCTACAAGAACAACGAAGCAAAAGGAATTCCATTCCCCAAATTCCAACCCATGGGAGTGTACTCAACATTGTGGGAAGCCGACGACTGGGCAACGAGAGGTGGATTAGAGAAAATAAATTGGAGCAAATCCCCATTTTACGCATATTACAAGGACTTTGACATAGAGGGATGTGCAATGCCAGGACCAGCAAACTGTGCCTCAAATCCTCGCAATTGGTGGGAAGGAGCTAATTACCAACAACTCAGTGCTGCGGAAGCAAGGCAATATCGCTGGGTTAGAATGAACCACATGATCTATGATTATTGCACCGACAAATCCAGAAATCCAGTCACCCCACCAGAATGTGTGGCTGGAATATGA

# >NtXTH12

ATGGTGTCTTTTCCTATGGAATTTAAGTGGGTTTTCTTGGGTATTTCTCTAATGTTGGTTGGTTTGGTTAGCTCCTCAAGATTTGAGGAACTATATCAGCCCAGCTGGGCAACAGACCATTTGACAAATGAAGGAGAAATTCTCAGGATGAAATTGGACAACCTTTCTGGTACTTGACATATTTAATATTGTTCATCAAGTTCAGTATTATAAGCTGATAATGTGACATTAACATTCTTATGTGCAATTTCAGGTGCTGGATTTTCATCAAAGAACAAGTATATGTTTGGGAAAGTTACTGTTCAGATTAAGCTTGTAGAGGGTGACTCTGCTGGAACTGTCACTGCTTTCTACGTAAGTTTCTTGCTTTGCTCCGCGGTAGTTGTTATTTTGTTCTTGTTTCTAGCCCCAATCACAATACTAAAACATAAACAAACTGTAACAATCTTTCATTGTGCTGAGGAAGTAGAAAAGTTAAAAGATTAACATATGTTGAAATGACTGGTCCATTTCATTGAATCCATTAATAATGAAATTTCCGGTGGCCGACAATATTAGTAGTGTGACATTATATCAAATTCAGTACATGTATTCGGCCATTGTTTCCTTGTGAACATCTTGAAATGCAATAACTCAAGCATTTACTTCCTTTTCACCTCTCGTCATATCCAGGAAATAGTTGCTCCCCTCTTGTCCAATTATTTTCTATTGGGTAAATGTCAGTTTGAGTGATCACATTTAGGAACTATATATTCTATTTATTAAGTAAATCTAGCTTGACTTTACTGACTATTTAACCTTTTTAATAATGAAATTATCTGCTTGCTGATAAGTATCTTCACTTGTGATATATATGCAGATGTCATCAGAGGGACCAACCCACAATGAGTTTGATTTTGAGTTTCTAGGCAACACTACTGGTGAACCATACTCTGTACAAACCAATGTGTACGTAAATGGCGTGGGTAACAGAGAACAAAGATTAAACCTTTGGTTCGACCCATCCAATGAATTCCACTCCTATTCCATCTTGTGGAACCAACACCGAGTTGTGTAAGTCTCCTTATTATTCCTATTGTTTTATCACATTAAATTAGAAAGATTGTTAAAATATATGAGACAGGGCAAGTTCCAACCATGGTATAAGGCTAACGTGCTGATTTTCAAGAATTGAAAAGCTAAAAGAAATGACTAATAGAGATTGGTTCCTTTTTCTTGACATGCTAAATGGGTCGTGCTTTTTTCAGAATGTCTTTCTTGATAACTCCAAATAATATAAACTACTAACACAAAGTTATCATATAAAATTTCTATTGCTTATACTAAGCTGTTAATTTTAAGTATTTGTGCAGATTTTTAGTAGATGAAACACCAGTTCGTGTGCATTCGAATTTGGAGCACAAGGGAATCCCATTTCCAAAGGACCAAGCCATGGGTGTGTACAGTTCAATATGGAATGCAGATGATTGGGCTACACAAGGCGGAAGGGTCAAGACTGATTGGTCACATGCACCCTTTATTGCATCCTACAGAGGATTTGAGATTGATGGCTGTGAATGTCCAGCAACTGTTGCAGCTGCTGAGAATTCTAAGCGGTGCAGCAGCAGTGCTGAGAAAAGGTATTGGTGGGACGAACCAACAATGTCTGAGCTGAGTCTGCACCAGAGCCATCAGTTGATTTGGGTCAGGGCTAACCATATGGTCTATGATTATTGCACAGACACTGCTAGGTTCCCTGTTGCTCCGGTTGAGTGCCAGCACCACCAGCACAAGACTCGCAACTAG

# >NtXTH13

ATGGTGTCTTTTCCTATGGAATTTAAGTGTGTTTTCTTGGGTATTTCTCTAATTATGGTGGGTTTGGTTAGCTCCTCAAGATTTGAGGAGCTATATCAGCCCAGCTGGGCGACAGACCATTTGACAAATGAAGGAGAAATCCTCAGGATGAAACTTGACAACCTTTCTGGTACTTGACATATTTGTAATATTGTCCATTAAGTTTAGTTAAAAGAATATTAGAAGCTGATAATGTGACATTGATATTCTTATGTGTAATTTCAGGCGCTGGATTTTCATCAAAGAACAAGTATATGTTTGGGAAAGTTACTGTTCAGATTAAGCTTGTAGAGGGTGACTCTGCTGGAACTGTCACTGCTTTCTACGTAAGCCTTTTGCTTTACCCTTCGTTGGTTATATTTTGTTCTTGTTTCTAGCCCGATCACAATATATACTAACACCGAACTGTAACAATCTTTTATTGTGTTGAGGAAGGAGAAAAGTTAAAAGATTAACATATGTTAAAATGACTTGTGGATTTTATTGAATCCATTAACTGTGAAATTTCCAGTGGCCGACAATATTAGTAGTGTGATATTATTTCTGATTCAGTACACATTCAGTCATTGTGTCCTTGTGAACATGTTAAATTTCAATAACTCTAGCATTTACTTCCTTTTCACCTCTCGTCATATTCAGGAGATAGTTGCTCCCCTCTTGTGCAATTATTTTCTATTGGGTAAATGTTAGTTTGATTGGTCAAATATAAGAGCTATTAGAGTATTTACTAAGTAAAGCTGGCTTGACTTTATTGACTACTTAGCCTTTTTAATATTGAAATTATGTGTTTGCTGATAAGTATCTTTTGGACTCTGTTTACTTGTGATATATATGCAGATGTCATCAGAGGGACCAACCCACAATGAGTTTGATTTTGAGTTTCTCGGTAACACTACTGGTGAACCCTACTCTGTGCAGACCAATGTGTACGTAAATGGTGTGGGTAACAGAGAGCAACGACTGAACCTTTGGTTCGACCCATCCAAGGAATTCCACTCATATTCCATCTTGTGGAACCAACGCCGAGTTGTGTAAGTCTCCTTATTATTATTCCTATTGCTTTATCTCATTAAATTAGAAAGATTGTTAAAATAAATGAGACAAGGCAAGTTCCAACTATGGTATAAGCTAAAATAACGTGCTGATTTTCAAGAATTGAAAAGCTAAAAGAAATGACTAAATTGAGAGTTAGATTCTCTTTTCTTGATATGTCAAATGGGTCCTCGTGCTTTCTTCAGAATGTCTTTTCTTTATTACTCTAAATAACATAAAACTATTAACACATAATTATCATTGATCTATATAAAATTTCTATTGTTTATACTAACTTGTTAATTTTAAATATTTGTGCAGATTTTTAGTAGACGACACACCAATTCGTGTGCACTCAAATTTGGAGCACAAGGGAATACCATTTCCCAAGGACCAAGCCATGGGTGTGTACAGTTCAATATGGAATGCTGATGATTGGGCTACACAAGGTGGAAGGGTTAAGACTGATTGGTCACATGCACCCTTTATTGCATCCTACAGAGGATTTGAGATTGATGGCTGTGAATGCCCAGCAACTGTTGCAGCTGCTGAAAATTCTAAGCGGTGCAGCAGCAGTGCGGTGAAAAGGTATTGGTGGGACGAACCCGTTATGTCCGAACTGAGTCTGCACCAGAGCCACCAGCTGATTTGGGTTAGGGCTAACCATATGGTCTATGATTACTGCACAGACACTGCTCGGTTCCCTGTTGCACCGGTTGAGTGCCAGCACCACCAGCACAAGTTTCATAACTAG

# >NtXTH14

ATGCCATCCTCTATGATTGTCTTTTTGATCCTAGCTATGCTACTAAACACAGGAGTTGGTGTCAACTTCGCCGAAGTTTTCGAGTCGAGTTGGGCACCTGACCATATTACTGTGGTAGGAGACCAAGTTATGCTCACCCTTGACAATGCTTCTGGTGGTTACCAATATGTCTCACTTCATTTCTACTTACTCTCTCTTCCTAGTCTTTTATTATATTTTCTGTGTTTTCAAGGTAATATAATTATATAACCATTTGATATATTATGTTGATATTTACAAAGGCTGCGGGTTTCAGTCGAAGAACAAATATTTGTTCGGGAAAGCCAGCGTGCAGATCAAACTAGTTGGAGGAGATTCAGCTGGAACAGTCATTGCTTTTTATGTACGTAACCAAACCCAAACAAAATATTCATCAAGAGTACTCTTACATTGTTTCTTCAAACTATAACCAACATTATTGGAATTAATATGTGGTTGCAGATGTCTTCGGAGGGAGCTAATCACGACGAATTGGACTTTGAGTTTCTTGGGAATGTTTCAGGAGAACCATACCTAGTACAAACAAATGTGTACGCGAATGGCACCGGAGACAGAGAGCAGAGGCATAGTCTCTGGTTCGATCCAACAACGGACTTTCACACTTACTCTTTCTTCTGGAATCATCATACCATTATGTAAGTAGTTCTTACTTCAACCATCTCTAAATGTGTTTGATCTTTCCTTTAAATAATTTTAGAGGGTTGCAACTTTGACAATCAAGTAAATTTCTATAACGAGTTGGTAACCTGCATTGTCGGTGTATATAAGTTAAACCTCAACTAATAAAGAAGAATTTTGAATTTTTGTAGCTTTTCAGTTGATGACATTCCTATTAGAGTGTTCCAAAACAAGGAGAACAAAGGCGTGGCATACCCGAAAAATCAAGGCATGGGAATTTATGGATCATTGTGGAATGCAGATGATTGGGCTACACAAGGAGGGAGAGTGAAGACCAACTGGAGCCACTCTCCATTTGTTGCAACATTTCGAGCGTTCGAGATCGACGCTTGTGATTTGTCTGGTGAGGACACAGTTGCTGCCGGTGCAAAATGCGGCAAGTTAGCAGAATGCTGGTGGGATAAGCCAGCTGTGAAGCAGCTGAACAAGAGCAAAAAGCGCCAATTCAAAATGGTTCAATCTAAGCACTTGGTCTATGATTATTGTAAGGATACTGCAAGATTCACTCAAATGCCTAAAGAATGCTTGGACTAG

# >NtXTH15

ATGAGAAGAAAAAGCTGCATGCTGACGACAGTGCCATGGCTGCCACTAAAACATTCTCTTGCTCGCTGTAATCCATGATCCTAATTTAAACAATCCAGTCCATAAATATATGTTAATACTAACTTATATATAAAACTAAAGTGCCTCCAAGTTCTTGATTGGCGTTTTTCCTAATAGACCAATATAGACTTGAACCTTTTACTTCTAGACCCTAAAATTAATTGGTTTCAAGTTCCAGACAGCAATGTTATCCTCTATGATTGTCTTTTTGATCCTAGCTATGTTACTAAACACAGGGGTTGGTGTCAACTTCACCGAAGTTTTCGAGTCGAGTTGGTCACCTGACCATATTACTGTGGTAGGAGACCAAGTTATGCTCACCCTTGACAATGCTTCTGGTAGTTACCAATATGTCTCACTTCATTTCTACTTACTTTCTCTTCCTAGTCTTTTATTATATTTTCTGTTTTTCCAGGCAATATAATTAAATAACCATTGATTTATTATGTTGATATTTTGCAAAGGCTGCGGGTTTCAGTCGAAGAACAAATATATGTTTGGGAAAGCCAGCGCGCAGATCAAACTAGTTGATGGAGATTCAGCTGGAACAGTCATTGCTTTTTATGTACGTAACGAAAACCAAACAAATATTCATCAAGAGTACTCTTATATTGTTTCTTTAAACTATTACCAACATTATTGGAATTAATATGTGGTTGCAGATGTCATCAGAGGGAGCTAATCACGACGAACTGGACTTTGAGTTTCTAGGGAATGTTTCAGGAGAACCATACCTAGTACAAACAAATGTGTACGCGAATGGCACCGGAGACAGAGAGCAGAGGCATAGTCTTTGGTTCGATCCAACTGCGGATTTCCACACTTACTCTTTCTTTTGGAATCATCATACCATTATGTATGTAGTTCTTACTTCGACCATCGCTAAATGTGTTTGATCTTTCCTTTAAATAATTTTAGAGGCTTGCAACTTTGGCAATTTAAAGTAAATTTTTATAACGAGTTGATAACATATATAACGAGTTAAATTCACATCTAATAATGAAGAATTTTACTTGTCATATGAATTCTTGCAGCTTTTCGGTTGATGACATTCCTATTAGAGTGTTCAAAAACACAGAGAAAAAAGGCGTGGCATACCCGAAAAATCAAGGCATGGGAGTTTATGGATCGTTGTGGAATGCAGATGACTGGGCTACACAAGGAGGGAGAGTGAAGACCAACTGGAGCCACTCTCCATTTGTTGCAACATTTCGAGCGTTCGAGATTGATGCTTGTGATTTGTCTGGTGAGGACACAGTTGCTGCAGGCGCAAAATGTGGCAAGTTAGCACAATGCTGGTGGGATAAGCCAGCCATGAGGGAGCTGAACAAGAGCAAAAAGCGCCAATTCAAAATGGTTCAATCTAAGCACTTGGTCTATGATTATTGTAAGGATACTGCAAGATTCACTCAAATGCCTAAAGAATGCTTGGACTAG

# >NtXTH16

ATGGGGATGAATATGTTGTTGGTGTGTGTGTTATTTGTCGTAGGAGCAATGGCTGCTGCGCCAAAGAAGCCAATGGATGTACCATTTGGAAGAAACTATGAGAATACTTGGGCTCCTGATCATGTCAAATACTTTAATGGTGGCAGTGAGATCCAGCTCTTCCTTGACAACCGCACTGGTAAAATACTATTTCTTAGTGTATTAGCTTTGAAGTACCGTTAAAGTTTGATTTTCTCCGTGTAACTTATAGGTCACGGGTTCGAGCTGTGCAATCAGCCACTAATGTTTGCATTAGTATAGACTGCAGTCTACGTCACATTCTTTAGGTTGCGATCCTTCCCGGACCCTACGGGATGTCTTGTGCACTATGCTGTGCTTTTTAGTGTATTTGGTATAACATAAGTCATTTTTCAGGAAAATATCTAGCATTTGAATATTAGAAAATATTTTCCACAGAAATGGGATAAATGACTTTGCTTTCTTATGAGAAAAAAGTGACTTTCTGCTAGATCTACAGTTAAACATTATTACTAATCTTTAGTATTCAAAATGTTCATTAAGGCCCTTACCGATTTGCTAATAATATTATTAATTTTAATTTAATGTATATTATTTAAAATAGATATTCGACCAACCAAACACTGGAACACAATTTTCATGAAAATATTTTTTCGAAAAATAACTTCGGTCATACCAAACACGCTCTAAGTATCTGCTACATCTTCTATGTAGAAGTTGTGTTATATTTTATTTTATTTTCCTTATTTTTTTTTATATTAATGTCAATGTGAAAGTATCAAAAACCCCAAAGTTCTGATTTTGCATTTGTTTTGGGACATAGGTACTGGATTCCAGTCAAAAGGATCTTACCTATTTGGGCACTTTGCTATGCACATAAAGATGGTTGCTGGTGATTCTGCAGGCACTGTCACTGCTTTCTATGTAAGTCATAACATATAGTACTATATTCGATAGCGACGCGAGGAATTTATTCGGAGGCGTACTCTCAAACTTGAAAGAAGTAAAAAATTTCCCGATAAAAAGTGTACAAATATATGTTATATACCCCTAAAACCTAATATTTTACCTATATATATAATATAATATTTCGACGAAGGTTGATCAATTGACCACCATTGATAGGTTGTAGCTTCGCCCTTGAGTATATCAATATTGTATATTAATAAATGGATAACATAATTGATCGATATCGCTTGACAATAATATAAAGTAAATATTAATTTGCGACAAAAAGTAAAGTCGTAAAAATTTCTACAGATCCCAACTCAAACTCATATACGAGTTAAGTTGTGATAATAAGCTGACACCACTTGTACAAAATAATAAGCCAAAGCCACTTGTATAAAATCTTATGTTTTTGGCTTATTTATTGGTTTTTGTTTGTCTTACCTGGGAATTATATTTACGGTAAAAACTTATCCATATCGGGTGTTTATATCCAACCAATGTATGTAAGACACATGTCTTTCACGCACAAATTTATCATTCACCATGATTTGCATGTTGATATGTATTCTTCACTTTTCGTTAACTAAGGTTAAAATGATTGATCGGATGTAAATATTCAATACAAATAAGTATTTACGTACGCTATAATTACCTTTATGTGCAGCTGTCTTCACAAAATAATGAGCATGATGAAATAGACTTTGAGTTTTTGGGGAACAAAACAGGAGAACCATATGTGGTACAAACAAATATATACACAGGAGGGAAAGGTGACAAAGAGCAGAGGATTTACTTATGGTTTGATCCAACCAAAGATTACCACACCTATTCTGTTTTGTGGAATCTCCACCAGATTGTGTAAGCTACTCTCTTTTCTATATTTTACTCCTACCTTTTATTCATTTATCGAATTCGAGGGTGATAGATTCTATCCGTCCTGTCACAATCTTTGCTAATCTACTCTTACATTGACATTTGCTTCCATGTATTAGCGCTAATTTTAATATTAATTTCTCAAAAGGCCATCAAACTTATGATTTTTTCTACAAAAGACACTAAATTATTTTTTGTTATTTTAAAATCACTTAACTTTTGATTGGTTACTTAAAAAATTCATTAAATATGATCGGGATGCCATGTAGGCATGTCAAATCTGCTAAAGTAGGCACGTCATTTTGACTCCAGCAAACTTTGGACGTGTTTCATTGCGACCCCTTTATTAATTTGACCCATTTTGACGCGTCCAAGTTTAACCCAACCCTTTCATTACCCATCTAAAATTACTGAACTTTTGGTTGGTTATAACTCATTAAATATTTTCGGGCCAAATAACCATGTCAAATCAATTAAAATGTCATTTTGGAGGGGTTGGATCATAACCTGTTTATCAATTTGACTTGTCTGACCCAAATCCGTCTATTTGACACCTCCTCTTGTAATAATTTATGATAAACAAATTAAGGGCATGCAATATTTGCTGAGACTTGAAATTAAACACTACGAAAAGGAGTCTTGGACCACCTATAAAATTTTCTGCTTGTGATCTATAGGCCACGGGTTCAACTCGTGAAAACAGCCAATGATGCTTGCATTAGGGTAGGTTGTCTATATTATATCCTTTGGGGTGCGGCCTTTCTTCGAACCCTACGTGAACACGAGATGCTTCGTGCATCGGACTGTTATTTTTCTTGGAATTAAACACTATGAAGGGGATTGTTGGAGTAAACGGTAAAATTATCTTTGTATGACTTATAGGTCACGGGTTCGAGTCATGGAATTAGTTATTAATACTTGTATTAGGGTACACAGGCTGCCTACCTCACACTCCCTTGAGGTGCGGCCCTTCCTCAAGTTCTCCATGAACACGGGATACTTCACGTACTGGACTGACGCTTTTACTTGAAAATTAAACACTTTATGCATATATGCAGGTTTTTTGTAGATGAGTACCCAATCAGAACATTCAAAAACAGCAAAGATTTAGGTGTCAAATTCCCATTTGATCAACCAATGAAGATATACTCAAGTCTATGGGAAGCAGATGATTGGGCAACAAGAGGTGGACTTGAAAAAATAGATTGGTCAAATGCACCTTTTGTTGCTTCTTACAAAGGATTTCACATAGATGGATGTGAAGCTTCAGTAAATGCAAAATTATGTGCAAATCAAGGCAAAAAATGGTGGGATCAAAAAGAATTTCAAGATTTGGATAAACAACAATGGAGACTTTTACGTAGAGTAAGGGATAAATACACTATTTATAACTATTGCACTGATAAAAAGAGGTTTGCAACTCTGCCAAAAGAGTGCAGGAGGAATAGAGATGTGCCAAGAAAATCATCAAAGAAGTCTCCTTAG

# >NtXTH17

ATGGGGTTCAAATGGATGAATATGTTGTTGTTTTGTGCGTTATTTGTCGTAGGAGCAATGGCTGCTGCACCAAAGAAGCCAATGGATGTACCATTTGGAAGAAACTATGAGAATAGCTGGGCTCCTGATCATGTCAAATACTTTAATGGTGGCAGTGAGATCCAGCTCTTCCTTGACAACCGCACTGGTAAAATATTACTCATTTCTTAGTGTATTAGCTTTCGAGCTGTGGAAGCATAGTGTAGGTTGTCCCCGTCCCGTCACACCTTTTGAATGCGGCTCTTTCTCGAACCCTACGGGATGCCTTGTGCACCGAGCTACCCTTTTTAGTGCATTTGATATAACATAAGTCATTTTTCAGGAAAATATCTATCGTTTTTATAACGGAAATATTTTCCAAAGTAAAGGGATAAATGACTTTGCTTTCTTACGGGAAAAAAAGTGACTTTCCGCTAGCTCTACGGTTAGACATTATTGTTAATCTTTAGTACTCAAAAATTTCATTTAAACCCTCTCCGATTTGCTAATACTATTATTAACTTTTACTTAATGTATATTACTTAAAATAGATATTCAATCGGACCTATTTACTAGCTATCGCTTTTGTTTTGCATCTTTCTCCTGGATTTCATGTTGTTTATATTTTTCCTATGATTTCTGTGATGATACTGATATTGTCTCCTTTTGTCTTTTTGTTCTCTTGAACCGAGGGTATTTCAGAAATAGCCTCTCTACCTCTTCGGGATAGAGATAAGGTTTGCGTATACACTACCCTCTCCAGACTCCACTAGTGAGATTTCACTGGGTTGTTGTTGTTGTTGTAGATATTCAACCAACCAAACATCGGAACACATTAATTTTTCATGGAAATATTTTTTCCAAGTATTAGCTCCACATTGCTGATCTGCTCCATGTTGTCTGTAGTATAATGTTATAGTTTTCTTTCTTTTCTTTTTGGTATTGATGTTATGGTGAAAGTATAAAAAGTCAAAATTTCTGATTTTGGTTTTGTTTTGGGACATAGGAACTGGCTTCCAATCAAAAGGATCTTACCTATTTGGGCACTTTGCTATGCACATAAAGATGGTTGCTGGTGATTCTGCAGGCACTGTGACTGCTTTCTATGTAAGTCTTAGCATAAAGTATATTAAATTATTAATATAGTATTATCAACAACAAACCTAGTGGTCCGATAGGTGGGGTCGGTGTAGATTATTATATATATCGATATCGCTTGACAATAATATAAAGTAAATATTAATTTGCAACAAAAAGTAAAGTCGTAAAAACTTCTACAGATCCCGACTCAATCTCATATACAAGTTAAGTTGTGATAATAAGCTGACGCCCCTTGTACAAAATAATAAGTTGACGCCACTTATACAAAATCTTGTGTTTCTGGCTTATTTATTAGTTTTTGTTTGTCCTGGAAATTATATTTACGGTAAAAACTTACCTGTATCGGGTGTTTACACTCAAACAATGAATGTAAGTCATATGTCATGCACAAATTTATCAATTAACCATGATTTGTTGATATGTATTCTTACTCTAACTTTTAGTTAACAAAATGACTGGTCGGGTGTAAAAATCCGATACGGATAAGTATTTACCCTATATTTACATTTATTGCAGTTGTCTTCACAAAATAATGAGCATGATGAAATAGATTTTGAGTTTTTGGGGAACAAAACAGGAGAGCCATATGTTGTACAGACAAATGTATACACAGGAGGGAAAGGTGACAAAGAGCAGAGGATTTATTTATGGTTTGATCCAACCAAAGATTACCACACCTATTCTGTTTTGTGGAATCTCCACCAGATTGTGTAAGCAACTTTATTTCATGTTTCTATTGCGGTTTTTTATTCATTTTCTGAACCCGAGACCTCTAGTTAACGTTATTCATCCTACCACAATCTGATCTAATGTTACCTTCTCATTTCCTTCCCTATACTATAATACTTAATTCTAGAATTAATTTCTCAAATGATCATCAAATTTATAATTTTTTCTATAAAGATCATTAAACTGATTTTTCTCATTTTAAAATTATTGAACTTTTGATTGGTTACTAAGAAAATTATTTCGGCCAGAAAATCAATTAAATATGACAGGAATGCCAAGCAAGCATGTCAAATCAGCTAAAACGTCATTTTGACGTAGAAATGACATCAGGTGGCTACTCAAAATGCTGATGTTTAATAATTATTTAATTTTTTGAGATAAAAATGTCCTGAAGCTACGATTGTTTTTTAAAAAAATTCAGGATAAAATAAATACTAGTCAATTTTTAAATAACATCCCTAATAATGATTGTTTTTGCACCTACCCCGTTGTGACTGGAACAAACTTTGGACGTGCTGCATTGTGACCCCTTTATTAATTTGACCCATTTCGATAAGTCCAAGTTTAACCCAACCCTCTCATTAGCCATCTATAAACTAATTTTTGTCATTTTAAAATTACTAAACTTTTAATTGGTTACTCATAAAATATTTTCGGGTCAAATAGACATGTCAAATCAATTAAAATGTTATTTTGGACGAGTTACATCATGACATGTTTATCAATTTGACTCGTCCCCAATCCGTTTGTTTGACACCTCTACTTGTAATATTTTATGATAAACTAATTAAGGGCATGCAATATTTATTGAGACTTGAAATTAAACATTGTGAAAGAGGAGCCTTGGACCAACTATAAAGTTGTTTTCGTGTGATCTATGGGTCACGAGTTCAAGTCGTGAAATCAGTGTAGACTACCTACATTATGCCCCTTGGGGTGCGGCCCTTCTCCGAACCCTGCATGAACACGAGATACTTCATGTATCGGACTACCCTTTTACTTGAATTAACCACTATATGCATATATGCAGGTTTTTTGTAGATGAGTACCCAATCAGAACGTTCAAGAACAGCAAAGATTTAGGAGTCAAATTTCCATTTGACCAACCAATGAAGATATACTCAAGTCTATGGGAAGCAGATGATTGGGCAACAAGAGGTGGACTTGAAAAAATAGATTGGTCAAATGCACCTTTTGTTGCATCTTACAAAGGATTTCACATAGATGGATGTGAAGCCTCTGTAAATGCAAAATATTGTTCAAATCAAGGCAAGAAATGGTGGGATCAAAAAGAATTTCAAGATTTGGATAAACAACAATGGAGACTTTTACGTAGAGTAAGGGATAAATACACTATTTATAACTATTGCACTGATAAAAAGAGGTTTGCAACTATGCCAAAAGAGTGCAGGAGGAATAGAGATGTGCCTAGAAAATCATCAAAAAAGTCTCCTTAG

# >NtXTH18

ATGGGTCTAAAAGGACTTTTGTTTAGTATTGTTTTGATTAATTTGTCATTACTAGGACTTTGTGGGTATCCCAGAAAACCTGTGGATGTACCCTTTTGGAAAAACTATGAGCCCAGTTGGGCTAGTCACCACATCAAGTACCTCAATGGTGGTTCCACTGCTGATCTTGTTCTTGACAGGTCTTCAGGTACTAATATTAATTAGTACTAATATAGCTGCCTGTTTTTTTCTCTCCTTATTTTGTCTAATTTTTTCATTTGATTTGTATGAATGAAGGAGCTGGATTTCAGTCAAAGAAATCATATCTATTTGGGCACTTTAGCATGAAACTGAGGCTTGTTGGTGGAGACTCCGCTGGTGTTGTTACTGCATTTTACGTAAGTTGTATGGGCGGAACTTGCCCTTCAGTCACGAGTTTAGCTGAACACATTAGTTTTGGTCTTTTAAATCTGTATCTGTTTTAAGAAATTGATTGAATATGTAAAATTATTAATTTAAAATTTAATAACTTAATAGGACTAGAATTATGAACCTATAAACTTCAAATCTTAGCTCCACCTATATATAGTCCGTTCATTTTTTGCACCATACTAGAAAATGGACACTGCTTAGATCTGCAATTTTCTCCATTTTTGCAGCAACATATAGTTTTTCATTTCCTTCACAATGAACAAACTAAGTTATGTGTTTTTGGACTGATTTTAATTATATTTTTGGGAATGTTTCAGCTGTCATCGAATAATGCAGAGCACGATGAGATAGATTTTGAATTCTTAGGGAACAGGACTGGGCAACCATACATTTTGCAGACGAATGTGTTTACGGGAGGAAAAGGAGACAGAGAGCAGAGAATCTATCTTTGGTTTGACCCAACCAAGGGTTACCATTCTTATTCCGTTCTTTGGAATACCTTCCAGATTGTGTAAGTACCTTAACATTTACAGTACTTACTAGTAGCTTTCTTTTCCGTAAATTTCTTGCTTTTTTTAAAAAATTTCCTAAAACAGAAAATATTACTAGATTATTGTATTGTCTGTCATCTTTTCTTCTATGGTCCCATGTATTTCTCGGAGCGGGTGCACTTGGTTTTATTCTACGTGATCCCTATTTGCTATGTTTAAAAATAGTATGGTAACAATTGTTGTTTAAGAAAAAAGCAAAATGCACATTTGTCGAGGCATTATTATTGTATTAAAATAAAGCACGAAAACTTGTAAGTTCACGATGGTCTAAAAATAGTCAAGATTATAGTAAAGACCTCTCCCATGTGAGCATCCTCTCGAGTTTATGTTTGTCTTTTTGTGTTAAACAAGAATTTTAAATTTTTGATATACTCAATATATATGTATATATTCCTCAATATTTACGCTTAAATTTATGCGTCTATATTGTTCATTTCAAATTATCTGATCCACATACTTTAATAAAGCAGCTAGCGAATTGGTGTCCTTGCAACAAAATTTTTGTCTTCTATCTTGCACCTAAATCTTGAAAAAATAATGTTTCCTTGTAGAATGTGCATCTTTGTCTTTCAAAAGACTTATTACTATTCACCGCTTTGAGCAAAGTGGTGGAAAGGATGCTCAAAAGCAATGAACTTTTTCTTCTATTTCATACTAAATATATAAAAAAGATATTTAATTTATTCTTATATATTAATTAGTATATATTTTATCCATTTCAGTACTCCTATTATTTTTGAGAGATGCTATAATGAGTAGTTTTTTCAAAACATTATTCTATCCCACCTTTGGGCCTCCACCAAAATGGACATGGCTAAATTGTCTTTAACGTTACAGGATCTTTGTGGATGACGTCCCAATACGAGCATTCAAGAACTCGAAAGACCTAGGTGTGAAATTCCCATTCAATCAACCCATGAAAATATACTCAAGCCTTTGGGATGCAGATGATTGGGCCACAAGAGGTGGATTGGAGAAAACAGACTGGTCAAATGCCCCATTTACTGCCTCCTACACATCATTCCACGTGGACGGCTGTGAAGCTGCCACCCCACAAGAAGTCCAAGTTTGTAACACCAAAGGCATGAGATGGTGGGATCAAAAGGCTTTCCAAGATTTAGATGCTTTGCAATATAGGAGACTTCGTTGGGTTCGTCAAAAATACACTATCTATAACTATTGCACTGATAGGAAGAGATACCCTACTCTTCCACCAGAATGCACTAAGGACAGAGATATTTAA

# >NtXTH19

ATGGGTGTAAAAGGACTTTTGTTTAGTATTGTTTTGATTAATTTGTCATTACTAGGACTTTGTGGGTATCCCAGAAAACCAGTGGATGTACCCTTTTGGAAAAACTATGAGCCCAGTTGGGCTAGTCACCACATCAAGTACCTCAGTGGTGGTTCCACTGTTGATCTTGTTCTTGACAGGTCTTCAGGTACTAATATTACTAGTACTAATATGGCTGCCTGTTTATTTTTTGTTATTGTTATTTTGTCTAATTTATTTTAATGAATGAAGGTGCTGGATTTCAGTCAAAGAAATCATATTTGTTTGGGCACTTTAGCATGAAACTGAAGCTTGTTGGTGGAGACTCAGCTGGCGTTGTCACTGCATTTTACGTAAGTTAATAGTTCAGTTGTTGCACCATACTAGAAAATGGACACTACTTAGATCTGCAATTTTCTCCATTTTTGCAGCAACATATTTTGCGTTGCAGAATTTTTTCATTTCTTTCACAATGAACAAACTAATTTATGTGTTTATGTACTGATTTTAATTATATTTTGGGGAATGTTTCAGCTGTCATCGAATAATGCAGAGCACGATGAGATAGATTTTGAATTCTTAGGGAACAGGACTGGGCAACCATACATTTTGCAGACAAATGTGTTCACGGGAGGAAAAGGAGACAGAGAGCAGAGAATCTATCTCTGGTTTGACCCAACCAAGGGTTACCATTCTTATTCTGTTCTTTGGAATACCTTCCAGATTGTGTAAGTACCTTAACCTTAGCTTTTTTTCCTGTAAATTTCTTGCCTTGTCTTTAAATTTCCTAAAACAGAAAATATTACTAATAGTTTAAGTTTTGATTATTGTATTGTCAGTCATATTTTCTTCAATGGTCCCATGTATTTCTTGGAGCGGGTGCACTTGATTTTATTCTACGTGGTCCCTGTCCTGCTATGTTTTAAAATAGTATGTTAACAATTGTTGTTTGAGAAAAAAAGCAAAATACACATTTCTCTAGGCATTAGTATTATTTAATTAAAATAAAGCATGAAAACTTGTAAGTTCACTATGGTCTAAAAATAGTCAAGATTATAGTAAGGACCTCTCCCATGTGAGCATCCTCTCGAGTATATATGTTGGTCTTTTTGTGTTAATCAAGATTTGAAGCTTTCGATATACTCTTATATATAGGGATAATATATGAAAGAAAAGAAGGGTAGTTCGGTGCACCGAGCTTCTATTATGTGCGGGGTTCGAAGAATGTCCGGATTATAAGGGTCTATTGCAGTAGCACGATGATCAGGATCATCTGTAGCAACTTTACCATTTTGTCAGGGTTTCACAATAATAATATATGTATATATTTCTCAATATTTACACTTAAATTTATGTGTCTAAATTGTTTATTTCAAATTATCTGGTCCACATACTTTAATAAAGCAGCTGGTGAATTGGTGTCCTTGCTACAGAATTTTTGTCTTCTATCTTGCACCTAAATCTTGAAAAAATAATGTTTCCTTGTCGAATGTGCAACTTTTTCTTTCAAAAGACCTGCGACTGTTTACTACTTAGCTTTGAAATTTGAGCACAATGGTGGAAATTTGGTCATGCTCGGGGGAGTTGCACGGTTCGTTACTCGGCCAAACATAATTACTAGTCAAATATACAAAAAAACATATATTTTACACTTTATTATTTTTTAGAGCAGACTCTATTGCGTAAGTTTTTATAAACACTCTATACTATTGGGCTTTATACTTTTCCTTCGGGCCACCACCAAATGGACAAGGCTAAATTGTCTTAAACATTGCAGGATCTTTGTGGATGACGTCCCAATTAGAGCATTCAAGAACTCAAAAGACCTAGGTGTGAAATTTCCATTCAATCAGCCCATGAAAATATACTCAAGCCTTTGGGATGCAGATGATTGGGCCACAAGAGGTGGATTGGAGAAAACAGACTGGTCCAATGCCCCATTTACTGCCTCCTACACATCATTCCACGTGGACGGCTGTGAAGCTGCCACCCCACAAGAAGTCCAAGTTTGTAACACCAAAGGCATGAGATGGTGGGATCAAAAGGCTTTCCAAGATTTAGATGCTTTACAATACAGGAGACTTCGTTGGGTTCGCCAAAAATACACTATTTATAATTATTGTACTGATAGGAAGAGGTACCCTACACTTCCCCCAGAGTGCACTAAGGACAGAGATATTTAA

# >NtXTH20

ATGCAACTCAAACTTGTCCCTGGAAATTCTGCTGGCACTGTCACCACCTTCTTCGTAAGTCATTCAACTTTTCCCTTACAAAATTGTATTAGTTCTATTTCATGTCCAAGTTCTGATGATTTAATATTTTTTCTTGTTCTTTGTTTCTTTCATCAGTTATCTTCACAAGGAGCTGGACATGATGAGATTGATTTCGAGTTCTTAGGCAATGTTTCTGGCCAACCTTACACAGTTCATACCAATGTTTACTCGCAAGGCAAAGGCAACAAAGAACAACAATTCCATTTGTGGTTCGACCCAACTGCTGCATTTCACACTTACTCCATTATCTGGAATGCTCAGAAGATCATGTAAGTTCCTTATATTAATCGCACTGTAAGAATTAATGATGTTTGCTTGCAATTTCTTCTTATTTTGGTACTGATGATTTCCATATGAATTCCCATCTTCAGTTTCTTGGTAGATAATAGTCCAATCAGAGTATACAACAACCACGAAAGCGCTGGCATTCCATTCCCAAAAAGCCAACCAATGAAAGTGTACTGCAGCTTATGGAATGCAGATGAGTGGGCTACACAAGGAGGTAGAGTCAAGACAGATTGGACACATGCTCCTTTCACTGCATATTACAGAAATTTCAATATTGATGGCTGCGCAGTCACATCCGGCGCCTCTTCGTGTAAGTCCACTGATTCAGCAAACAATGCTAGGCCATGGCAAAATCAAGAACTTGATGCTAAGGGCAGGAATAGGCTACGATGGGTGCAGAGCAGACACATGGTTTACAACTATTGTGCTGATTCTAAGAGGTTTCCTCAAGGCTTTTCTCATGAGTGCAAGCGTTCGAGGTTCCTCTAA

# >NtXTH21

ATGTCGCCTCGTTTCTCTTTCAAAATGTTAATCCTTCCTATAGTCATGGCAAGTCTATGGGCAGCCGCCTCAGCTGGTAATTTTTATAATCTTGCAGATATCACTTGGGGCGAAGGACGTGGTAAAATAACAGAAGGAGGCAGAGGCCTCTCTCTGTCCCTTGACAAATTATCTGGTTCAGGTTTTCAATCCAAGAATGAGTATTTATTCGGAAGATTTGACATGCAACTCAAACTTGTCCCTGGAAATTCTGCTGGCACTGTCACCACCTTCTTTGTAAGTCATTCAACTTTTCCCTTACAAAAATGAATTAATTCTATTTCATGTCCAAGTTCTGTTGATTTACTATTGTTTCTTTCTTCAGTTATCTTCACAAGGAGCAGGACATGATGAGATTGACTTCGAGTTCTTAGGCAATGTTTCTGGTCAACCTTACACAGTCCACACCAATGTTTACTCGCAAGGCAAAGGCAACAAAGAACAACAATTCCATTTGTGGTTCGACCCAACTGCTGCATTTCACACTTACTCCATCATCTGGAACGCTCAGAAAATCATGTAAGTTCCTTATATTAATCTCAGTGTAAGAATGTTTGCTTGCAATCTCTCTTCTTTAGTACTGATGATGTCTGTATAAATTTCCATCTTCAGTTTCTTGGTGGATAATAGTCCAATCAGAGTATACAACAACCACGAAAGCAATGGCATTCCATTCCCAAAAATCCAACCAATGAAAGTGTACTGCAGCTTATGGAATGCAGATGAGTGGGCAACACAAGGAGGTAGAGTCAAGACAGATTGGACACATGTTCCTTTCACTGCTTACTACAGAAACTTCAATATTGATGGCTGCGCAGTTACATCCGGCACCTCTTCGTGTAAGTCCACTGATTCAGCCAACAATGCTAGGCCATGGCAAAATCAAGAACTTGATGCTAAGGGCAGGAATAGGCTACGATGGGTTCAAAGCAGACACATGGTTTACAACTATTGTGCTGATTCTAAGAGGTTTCCTCAAGGCTTTTCTCATGAGTGCAAGCGTTCGAGGTTCCTGTAA

# >NtXTH22

ATGGCTTCTCATTTGTTTCTAATTTCCATTCTAATGGGCAGCCTAGTTGCTGCCTCAGCTAATTTTAATAATCTTGCAGAGATCACTTGGGGCGAAGGACGTGGTAAAATAACAGAAGGAGGCAAGGGCCTCTCCCTGTCCCTTGACAAACTTTCTGGTTCAGGTTTTCAATCCAAGAATGAATATCTCTTTGGAAGATTTGACATGCAACTCAAACTCGTTCCTGGAAACTCTGCTGGCACTGTCACCACCTTCTTTGTAAGCTATTCATCAACTTTCGATTTTTATAAATTTAAGTCTATTTTCATTTCCAAGTTACGATGATTTGACTTTTGTTTCATGTTCTTTATTTCTTGATCAGTTATCTTCACAAGGAGAAGGACATGATGAGATCGATTTCGAGTTCTTGGGTAATACGACGGGCGAGCCCTACACTGTCCATACCAACGTCTATTCTCAAGGAAAGGGAAACAAAGAACAACAATTCCACCTTTGGTTCGATCCAACTGCAGCATTTCACACTTACACCATTGTGTGGAATTCTAACCGCATAGTGTAAGCTGTTTTTTTAATCATGTTATATACTGATAGTATGTTATCTGATGGTTTAAATATTTTTTATATTATTAATACATAAAGAAGTTAAATTCTATTTTTATTACTCCACAACATTTTATTTTGCTTACATTTTTTTCAATATGATGGATCAGGTTCTTGGTGGATAACATTCCAATTAGAGTATACAACAACCATGAAAACAATGGCATTCCATTCCCAAAGAGCCAACCAATGAAAGTGTACTGCAGCTTATGGAATGCAGATGAGTGGGCTACACAAGGAGGCAGAGTCAAGACTGATTGGACACATGCTCCTTTCACAGCTTACTACAGAAACTTCAAAATAGATGGCTGCGCAGTCACATCCGGCGCCTCTTCATGTAAGTCCACTGATTCTGCAGGCAATGCTAAGGCATGGCAAAATCAAGAACTTGATGCTAAGGGCAGGAATAGAGTCCGATGGGTGCAAAGTAGACACATGGTTTACAACTACTGCGCTGATAAAAAGAGGTTTCCTCAAGGCTATTCTCATGAATGCAAGAGCTCAAGGTTTTAA

# >NtXTH23

ATGGCTTCTCATTTTCTTCTGATTTCCATTCTAATGGGCAGCCTAGTCGTTGCATCAGCTAATTTTAATAATCTTGCAGAGATTACTTGGGGCGAAGGACGTGGTAAAATAACAGAAGGAGGCAAAGGTCTCTCTCTGTCCCTTGACAAACTTTCTGGCTCAGGTTTTCAATCCAAGAATGAGTATTTATTCGGGAGATTTGACATGCAACTCAAACTTGTACCTGGAAACTCTGCTGGCACTGTCACCACCTTCTTTGTAAGCTATTCAATTTTCGATTTTTATAAATATAAGTCTATTTTTATTTCCATGTTACGGTGATTTGATTTTTATTTTATGTTCGTTATTTCTTGATCAGTTATCTTCACAAGGAAAAGGACATGATGAGATTGATTTCGAGTTCTTGGGTAATACGACTGGCGAGCCCTACACTGTCCACACCAACGTGTATTCTCAAGGAAAGGGAAACAAAGAACAACAATTCCACCTTTGGTTCGACCCAACTGCAGCATTTCACACCTACACCATTGTGTGGAACGCTAACCGCATACTGTAAGCTATTTTATAATTATGTTATATACTGACAGTATGTTATCTGATGGTTTAAGTATTTTTAGTTAAATTCTATTTTTGTTACTCCACAACGTACATTTTTTTATTTTGCTTACATTGTTTTCAATATGATGGATCAGGTTCTTGGTAGATAACATCCCAATTAGAGTGTACAACAACCATGAAAGCAATGGCATTCCATTCCCAAAGAGCCAACCAATGAAAGTGTACTGCAGCTTATGGAATGCAGATGAGTGGGCTACACAAGGAGGCAGAGTCAAGACTGACTGGACACATGCTCCTTTCACTGCTTACTACAGAAACTTCAAAATTGATGGTTGCGCAGTCACATCGGGGGCCTCTTCATGTAAGTCCACTGATTCTGCAGGCAATGCTAAGGCATGGCAAAATCATGAACTTGATGCTAAGGGCAGGAATAGGGTCCGATGGGTGCAGAGCAGACACATGGTTTACAACTACTGTGCTGATAAAAAGAGGTTTCCTCAAGGCTATTCTCATGAGTGCAAGAGCTCAAGGTTTTAA

# >NtXTH24

ATGGCTTCTAAATTTTCATCAGTAATGCTTCTGCTTTGCATAATAATGAGCATACAATTATTAGCAGCCTCAGCTGGTAACTTCTACAGAGATGCTGTAATTACTTGGGGTGAAGGACGTGGCAAAATACAAGAAGGTGGCAGAGGTCTTGCCCTCACTCTTGACAAATTATCAGGCTCTGGTTTTCAGTCCAAGAATGAATATTTATTTGGAAGATTTGACATGCAACTCAAGCTTGTACCTGGAAATTCCGCTGGCACTGTCACCACTTTCTTTGTAAGTTGCTCTGCTTAAATTGAACACTAGTATCCTATAATTCTATTTCGCCATATATGTGCTCATGGATTGTTCTTTATTTTCTTTGTTTTTCATCAGTTATCTTCACAAGGAGAAGGACATGATGAGATTGACTTTGAGTTCTTGGGCAATGTTTCTGGACAGCCTTACACTGTCCATACCAATGTTTATACACAAGGAAAAGGAAACAAAGAACAACAATTCCACCTTTGGTTCGACCCAACTGCCGCATTTCACACTTACACCATTGTCTGGAACCCTCACCGCATAGTGTAAGTTAGACAATCACCTTACATATATCATTCTTATATATCCGGAGTTTAACTTCTAAACACTGAAAGGGTAAAAGAAATTTTTACACTATCAAGTCATCTAACAGATATCTATAATAAGTCAAACTAGTTACCTGAAAAATAAAGCATATTCTATGGCTTTCTATAACATATTAAATTTTACACTGATAGTGTAACAAGAAAAACTTACACTGTTAATGTATATAAGTTTAGATCTAAGTTCTTGTATTTCTGTTAGTTTTACATTTTATCTTACCTATGCATTCTTTGATATAATCAGGTTTTTAGTGGACAACAGCCCCATTAGAGTATACAACAACCATGAAAGCATAGGCATTCCATTCCCAAAGAGCCAAGCAATGAGAGTATACTGCAGCTTATGGAATGCAGATGAGTGGGCAACACAAGGAGGCAGAGTCAAAACAGATTGGACACTTGCTCCTTTCACTGCTTACTACAGAAACATCAATATCGATGGTTGTGCAGTGTTATCCGGTACCTCGTCATGTAAATCCAGCAATTCAGCAAACAATGCTAAGCCATGGCAAACTCATGAACTTGATGGAAAGGGAAGGAATAGACTAAGATGGGTGCAAAGCAGACACATGGTTTATAATTATTGTGCTGATTCTAAGAGGTTTCCTCAAGGTTTTTCAGCTGAGTGCAAGAGTTCAAGATTTTAG

# >NtXTH25

ATGGCTTCTAAATTTTCATCAGCAATGCTTCTGCTTTGTATACTAATGAGCATCCAATTATTAGCAGCCTCAGCTGGTAACTTCTACAGAGATACTGTGATTACTTGGGGCGAAGGACGTGGTAAAATACAAGAAGGTGGCAGAGGTCTCGCTCTCACTCTTGATAAACTTTCAGGCTCTGGTTTCCAGTCCAAGAATGAATACTTATTCGGAAGATTTGATATGCAACTCAAGCTTGTGCCTGGAAACTCTGCTGGCACTGTCACCACTTTCTTTGTAAGTTGCTCTGCTTAAATTGAAACCTACTATCTTAATTCTTGTAATATGTCATATATGCTAATGGATTGTTATTGTTCTGTTCTTTTCTTTGTTCATCAATAGTTATCTTCGCAAGGAGAAGGACATGATGAGATTGATTTTGAGTTCTTGGGTAATGTTTCTGGCCAGCCTTACACTGTCCATACCAATGTTTATACACAAGGAAAAGGAAACAAAGAACAACAATTCCACCTTTGGTTCGATCCTACTGCTGCATTTCACACTTACACCATTGTCTGGAACCCTCACCGCATAGTGTAAGTTAGACAATCACCTTACATATATCTTTCTTATATATCCAGAGTTTAACTTCTAAACACAGAAAGTATAAAAGATGTTTTTACACTATCAAGTCGTCTAGCACTGACTGTCTGTAATAAGTCAAACTAGTTACCTGAAAAATAAAGCATATCTACGACTTTCTATAATAGATTAAATTACACTGTTATACTGTTAATGTATTTAAGTTTAAATCTAAATTCTTATATTTCTGTTAGTTATACATTTTATCTTACCAATTCTTTGATATAATCAGGTTCTTAGTGGATAACAGCCCCATTAGAGTATACAACAACCATGAAAACATTGGCATTCCATTCCCAAAGAGCCAAGCAATGAGAGTATACTGCAGCTTATGGAATGCAGATGAGTGGGCTACACAAGGAGGCAGAGTCAAGACAGATTGGACACTTGCTCCTTTCACTGCCTATTACCGAAACATCAATATTGATGGTTGTGCAGTGTTATCCGGTACCTCGTCGTGTAAGTCTAGCAATTCAGCAAACAATGCTAAGCCATGGCAAACTCATGAACTTGATGGTAAGGGAAGGAATAGGCTAAGATGGGTACAAAGCAGACACATGGTTTATAACTATTGTGCTGATTCTAAGAGGTTTCCTCAAGGTTTTTCTGAAGAGTGCAAGCGTTCAAGGTTTTAG

# >NtXTH26

ATGTCATTATCCTCTGCTTCCTCCAGAATTCCAAAAATGTTCCTTCAGCTCTCTGTTCTTGCAGTTTTCCTCCTATGCACTGCTTGTGCTGATAATTTCTACCAAGACGCGACTGTCACCTGGGGTGACCAGCGGGCTCACATACAAGAAGGTGGCCGTCTTCTAACCTTGTCTCTCGATAAAATTTCAGGCTCTGGCTTTCAATCCAAGAGTGAGTTTTTATTCGGAAGGTTCGACATGCAGCTCAAGTTAATACCTGGAAATTCTGCTGGCACTGTCACCACTTTCTACGTAAGCCACCTTCATTTAACTGTTATATATATAGGACATACATATTTGACACAATTTATTAACCTGGGGTGACTATTTGTTTTTGTAATCTGCAGTTGTCGTCTCAAGGAGCAGGGCACGACGAAATTGATTTTGAATTTCTGGGAAATTCATCAGGCCAGCCTTACACAGTTCACACCAACGTTTATTCTCAGGGAAAAGGCAACAAAGAACAACAATTTCACCTCTGGTTCGATCCCACCACATCGTTTCACACCTACTCTATCATTTGGAACGCTCAACGCATCATGTACGTTACAAATTAATTAACATCTTTGTAATCTAGTATATACAAAACATACTTCACTAGAAAACAATTTTTACTTGGAACAATTCATGTCTGACATTTTGGGGTTTTGTCTATTTCCAGATTTTTGGTGGATAACATACCAATAAGAGTGTACAACAATCACGAAGCACTTGGGGTTGCATTTCCAAAGAATCAAGCAATGAGAGTGTACGCTAGCCTATGGAATGCTGATGACTGGGCAACACAAGGCGGGCGAGTGAAAACGGACTGGTCCATGGCTCCATTCACAGCTTCTTACAGGAATTTCAATACAAATGCTTGTGTTTGGTCAGCGGCATCATCTACTTCATCTTGTGGAGGCTCTAAATCCACTGATTCAGCGAATAATGATCAGACATGGCAAACTCAAGAACTGGACGCTAATGGCAGAAATAGGCTTAGATGGGTGCAGCAGAAATACATGACATACAATTACTGTACAGATGCTCAAAGGTTCAATCAAGTCATTCCTCCTGAATGCAAGCGTTCAAGGTTTTAA

# >NtXTH27

ATGGGGTCAAGAATTTTCTTGGTTCTAGCACTTGTGTTTAGTTCTTGCATGGTTTCTTATGGTGGAAATTTCTTTCAAGAATTTGACTTTACTTGGGGTGGAAATAGGGCTAAGATTTTCAATGGAGGTCAGCTTATGTCTTTGTCTTTGGACAAAGTTTCTGGCTCTGGTTTTCAATCTAAGAAAGAGTATCTCTTTGGGAGAATTGATATGCAAATCAAACTTGTTGCTGGAAATTCTGCTGGAACTGTCACTACATACTATGTAAGTATTCTTTACAAAAAAAATTAAAGAAAGCAAAGTTGAACACATTATTTTATGGTTAGCAATAATATTAGTAGTTCATTTGGAAGGAAATCACGGAACAATGGTAAAGTCGTTTCGGTGGAAGGAGAATCGCCACTAATGTTTGTATTAGTGTAGGCTGTTTACATCACATCCCTATTGTGCGGCCTTTTTTCGGACCCTACATGAATACGAAATACTTTGGATATTAAACTGCTCTTTTTTTAATATAGTAATATTAGTAATTAATTCATTTACTTGAAAAGAATGTTTGTGAAAGCTATTTGTAATTTAATTTACAAGTTCGATCGAATCTAGTAGCTTTGATTCGTCCCGTATTTGTCACAAAAAATTATTAAATATATACTAAGAATTATTAATTTAGAAAATTCAATAACTTAAACATCACATTTTTAGTATGTGCCTACTTTAAGAAAGTGAAATTTGATTCATGTTGATATAACCACAAATTATGCCTACTCTTATGTATTGTGGTCTTATGAAACAACTTGGTTGGTACTCATGTGACTATTTTGGTGTGGCCAAGAATTTTTCTTTTTAGCTGTTAATTTTTATTTGTGATAATGACTTCTTTAAATTCCCTATTCCTTTTCCTTTAATTATTTGGATTCCTCATGCATATTACATGGAGTATATCAAACTTTACAAGTTTTCAACTCTTTTCTCATAATATTGTACTCATCCCAATCCTTTATAGCTCTAATTAGTAAATAAATATTGCCTTTTTTTTTTACTAATTATTTATTTGCTTTTATTGCAGTTATCTTCTCAGGGACCCACACATGATGAAATTGACTTTGAATTCTTGGGAAATGTTACTGGTGAACCTTATATTCTCCACACAAACATTTATGCCCAAGGCAAAGGAAACAAAGAGCAGCAATTTTACCTTTGGTTTGATCCTACCAAGAACTTCCACACCTACTCAATCATATGGAAACCCCAACATATCATGTAAGCAAAGCCCTCAATATTAATTATCTCAACATATTACCAAACAATTAACAAATTAAAAGAATTTAAAAATCTGTGTACGATATTTTTAGACCGGCAGATTAAGCTAGCATTTGAACAAAGATTTGGTTAACTTGAAAAAAAGGAGTTTTTGAAGTTATGTTGAAAAATAATTTTTAAATGTTAAAGCCAGTTTTTGAAAACTTGAAAATTTTGAACTTCAAAAATAGATCATATTCCACGACCAAACAATATTTTTAATTTTTTTTTGAAAAAAAAGTAGCCAAAAATCTATGGTCTGAGCTAAGTTGATATCTAACAAAAGTAGCATGCATGATCTAGTAACTAGAAAAATATGTCAAAAAATCTATTATAACATAGTTAAATTAGGCTGATAGTGTCAAAAGATGTACCTAGACTACAAGGATTGATTTCTATTCATATTTATCATTAACTAAAACCTATCTTGCTTGAGCTTTTCAAAACGTTGTCGTACCCGTGTCACATGCCTTGTTTTTTAAGGATCGGACAAGAACATGATAGTATTTTTAAAAAGTTCGAACAACGTAGCAAAAAATACAAGATTTTCCTTGATCCAATATAACATTTTCTCATATTTACTTTTTATATATTTCAGTTTCTTGGTCGACAACACACCAATAAGAGTTTACAAGAATGCTGAATCCATTGGTGTGCCATTTCCCAAGAACCAGCCCATGAGAATTTACTCTAGCCTTTGGAATGCTGATGATTGGGCAACAAGAGGAGGCCTAGTGAAAACTGATTGGTCTAAAGCACCATTTACAGCCTACTATAGAAATTTCAATTCTCAAACTTTTAGCAGTTCACAATTTTCAAATGAAAAATGGCAAAATCAAGAACTTGATGCCAATGGCAGAAGAAGACTCAGATGGGTGCAGAGGAATTTCATGATTTATAATTATTGTACTGATTTTAAGAGGTTTCCTCAGGGTTTTCCTCCAGAATGCAAAAGATTTTGA

# >NtXTH28

ATGGCAAGGTTTTCGTCTTCTTCATCTAGGTCCAGGTCTTCTCTTCCATACATTGTATTGCTCTTCGTTGCTGCCCTTTTTGTCTTTAAGGTAAGAATTTTAATAAATATTCTTAATTATTTCTCTAATTATCTTCTTAAAGATTGTATAATCATCCTTCATTTGCATCTTTAGCTCACACTGACATGAAAATTAAATTGATATGATAATTCTGATTTTGTTTTTGTACGATTGTGTAAAGTGCATATTTTCTCATTGTGTTTTTCTTATAATCGTTTCAATTTATGGAATGTCCAAACTATGTTTTCTTTTCGATTATAAAACACACACATCTCATTTCCTCTTCTTCTTTTTTATCCTTGAAAATAAAGATCCCAATAGATGAAGATTATGCTTAAAACTAGGGATATGATTTCACTGTCTCCTATCTCTATCTGCATAGTCATGTTCGTATGAAGTTGGGATTTTGAGTTTATGAATTTTGAATTATTGAAACGGATCATTGAATGTCAGATCAATTATTATATACTAAGTGAATTTGTTAACACAAATTAAGATCTTGGTCGAATTGTAGCTCCGCCATGGGTCTTGTCATTTTTTTCTTCTCAAGAGTTAAAAATTAATTTGTGGAATTAATTACATGAGCAGATAGATGTTATCATATCTCAGACATTTAGTTCAGCCCGTCGCAACCTGGAGAACACTCCTAACCGTATCTTAGTGAAGTCTAAATCCCAAGAAACTGATGACAGGTATACAAATTCTTTCCTTCTTCCTATTTTTATTTTTATTTTCTTTCAATATTTTTTCTTGGTGAGTAAATTTTTTATTTTACAAACCAAATATAACATTACAGCACTTGTTTTACAAGGAACTTGGACATCATGCCCCAAGTCTTATACTCATCAGACTCTATCATTGAGTACAAGAATGAAAACAACTAAGGCTATCCTCAATCTCTAGGGTACCACTTCATAGCTTGCAACTTTCTAGTTAGCTTCTTATACTGATTTCCTCGACTATGGACTTCCTGTGCTATCAATCGTAAGATGATCTCCTTTGTCCTGGACTTGCCTTGAAATATCCTCAAGTTCCATCCATATATGGTAGACACACGCTGCCATGCATAGTCTATACATATTAGCCATTGCTGACTGTCCATTTGCATGAACCTCTGCCCATTAAACTTCTTCAGACCATCCTTGAGCTCTCTTTTTGATTCCCTGCTATTTCAATAATCTCTTCCACATTTGCGATGTCATTTCACATTTAAAGAATAGATGCTCAGCTGTTTCTGGTTCTCTAGCACACAGTGGGCATGTCAAGTCTTCAATCTGACTCTATTTAGCTATTCTATCTCTTGTTTGCAATCTCCCTTGTAAGTTTAAATACAGTATAAACACCCATCTAGGAGCCTCGTAGTTGTTACATATCAGTCTTCCCCAAGAGCATTTAGGGAACTCACCTCGAAGCTTCATGTACATTCTTTGAATAGAAAAGGTCCTTATCAGTATGAATTCCTTCTCTTGTAGTCCCACTTCTTCTAGGTATTTTGCTGCTTTTAAGATCTTCTGTACAATTCAAGATGCTTGCTTAGGTTGACTCATACACCGTGTGGTTCTTCTCCCATAGTAGCAATGTATCCACTTCACCCATAGCCTTTTCTTTTTGGTACAAACATTCCACAGTAGTTTGCATATAGCAGCCTTGTTCCAAGCTTGAATATCTATGATATTTAGTCCCCCTACAACTTTTGGAATACAGAATTTATCCCACGCAATTAGTGCCCTTTTTGAATTATCTACTCCTCTTGTCCATAAGAATCTTTTGCATACACTTTCGACCAATTGAATCAGATTCTTTAGTAGGGTAAATATTTGAGACCAGAATGTTTGGATGGAGAAGAGAACACTCTTGATTAGCTGAAGCCTCCCTGCATAAGACAGTAGCTTTGATGTCCAGCTTGTGACCCTGCCTATTATCTTATCAATTAGGGGCTTACATTGAGCCAAGGACAGTCTTTTTGTGCTAAGAGGTACCCCTAAGTATCTTATAGGAAGTTCTCCTTTTAAGAAATCAAGGTGGTCAAGTATGTCAAGCTGTAATTGCTGATTAACTCCACCAAAGTAAATACTGCTTTTCTCCACATTTGCCTCCAGGCCTGACACTATTCAGAACATCTGAAAACAAACATACAAAGCTTTCACAGAAGCCATATCACCTTTACAAAACAGCAGAAGGTCATCTGCAAAACCAAGTTGTACTATATTCAACTTTGCACACTTCGGATGCAATTTAAACTCCTTATTCTCCTTCAACCCCTTTAGAACTCTTGTGAAATATTCCATGGCTAGTACAAATAGGAAAGGGGACATAGGGTCCCCCTGTCTGAGTCCTTTCTTTGATGGGAATGGCTTCATACGATTCCCATTAATGATAATGGAATAGGAGACTGTAGTGACACACTTCATGACCCATTGAATGAAGATCTCTGGGAAATTCAGTTGAATTAGAATCTGTTCTAGAAAGCACCACTCCACAGAATTATATGCCTTCCTCATATCAATCTTCATACAGCATCTTGGAGAGATAGACTTCCTGTTATACCCTTTAACTAGTTCATGACTTAGTATAATATTATCTGATATTACCCTTCCAGGAACAAATGCAGACTGACTATCATCTACCAGATAATCCATTACCTCTTGTAGTCTGCTAGTAATGAGTTTGGAAATGATCTTATATATGGTGGTGCAACAGGATATTGGTCTGAACTCAGCTATTCTGGATGGATTATGCACTTTTGGTATCAAAGTCACTGTGGTGCAATTAATTGGCTTGTAAACCTTAGTTGTTCTGAAAAACTCTTGTACTACCTCAACTACTTCATCTCCTATGATTTCCCATATCTTCTTAAAAAATACAGCATTATATCCATCACACCCTGGGGCTTTAGAATCTTCTATACTTTGTAGAGCCTGGTAGATCTGTTCCTTAGTCATAGGAGCTATTAGCTTCAATTGCTGATCTCTTATCAACACATTCCCTCTCTTCATTACTTCAAGTTCAATAGCTGGAATTTTCACTGCTGTTGATCCTAGAAGCTGTTTGTAGAATTGAATAATTTTCTGTTGCACCTCCTCTTGATTTTGGGTATGCATCCCTGTCTGAGTGAGCAGCCTAGTGATTTTCTTTTGGGCATTTCTATTCTTGATACTAGCATGGAAGAAAGCAGAGTTTGAGTCACCCAGCTTTAGCCACTGAATTCTAGACTTCTGATTGAGAATACTTTCCTCTATCATAATCCACTTCTCCAGCTTCTCTTTCAGATCTTTTTCACTGTTCTTCAATCCCTCTGAGTGATTAGGTATTCTCATTTCCAATTGTACTTGTTGTAGTTTCTCTCGAATAGCTTGGATCTTTTCAGGAATTCTACTATACTCCTCAACATTTAACTGCTTCAATCCTTTTTTCAGCCTCTTTAACTTCATCCAAATTTTTTGCATAGCATGCCTCAGCATGCTAACTTCCCAGGTATCTTTGACTAGCCTTTGAAACTCCTTATACTCAGCTAGGTTATTGAAGAACTTGAATGGTTTAGGCATAGGTTGGTGATCATGTTCAAAGTAAACACAAATAGGGGTATCATCAGAGAAGAGAGGATCCTGCAGTACCACCTCTAACTGTGTCATATTCATCATCCATCTAGTATTAACTAAAGCTCTGTCTATTCTGCTATATACATGATTGTTTGTCCACATAAATTCTCTGCCAACTATTTTAAGTTCTGTCATTCTTGTGTTCTCTAGAAAATCAAAGAAGTCTCTAGTTTCTGCCTCCATAACAGGGTTACCATTATACCTATCTTCAAAACTTTGAATGGCATTATAGTCTCCCATAGCAATCCAAGGTCTATGTAGCTCATTATGCAGCTGTTCCAACTCTTCCCACATGGCATTTCTATATTCAATGGTATGCAAACCATATACTGCTGTCATCCAGAATTCTTTATGACTTTGGTGTATGATAATCTTCCCAGTGATTAACTGTTCTAAACAACTATGAATGGAAAAGTCCAGTACTCCAGGATCCCACAAGATCCATATTCTCCCTCTGTTACTAGCCACATAATTATCTGTCCACTTCCAATTCCTAGAGATATTTTGGATAGTTTTCTCTGCCTAGGTTGTTTCACCTTGTGCTCAATTATTGCTATCAACACTACTTTATTGTCTCTAAGATACTGCTTTATTTCTCTTTGTTTATAGACTTTATTCAAGCCTCTAACATTTCATGATACTATATTCATCTTGGTATAAAAGGATCATTTGTCTCCTACATCAATTCCCACCTATTTATGCTGCTAGGCCCCAAGCTTACTTGTGCAGTCACCTGAGAACTTGATGGATCAAGGGTATTGAAACCATTGGATATTCTCACCCCATGTTCTTTTCCATATTTCTTGAGCCCTTTGACACAGACTTGTTCTTAACAGTCTTCCAACCTTCATCATCTGCATTCATTGCTGGACTTTGTGAAGAGTTCTGTGGCTCCAATTTCCTCTGAGCTCTAGGCTCTCCCGTTCCCCTGTCACCTCCTGTTTGTTTAGGTCCTTTAATTATGACAGTTGCACTCTTACTCTGCACAGATTGTACGGAATTTGAACTTCCAGCTATTATCGGCGGGGTCTTAAGCCCTTTTGTTAGGGCTGGCCACTGTTCCATTGGTTTTTCCTCAGCTATAACTGTAATTGCCTTATCTAATTTCTCTACTGGCTTCGTGATTGAACCCTTCGATGGTAACTCCTTCAATTCCACACTGATTGCATGTCTCCGAGGTCGACCTCGTGGCATTTTCCGGTGAAGGTGGCGCACAATAGCACTAGCACTCTATCATACGCCGAGAGCGTATATTCTTCTGAGATCTTACAATACTTAATTGGACCTTATTTTCACATTCCCAATATTGACTAGTATGTGAACCTTTTTTTCTAAAAGCTTTGAAATAAATTGCAGCTGTAAAGTACTAATAGTAAACACTAGAAAAAGGTTATGCATTAATTTTTGCATTACACAAAATAATATGTGAGAACTGCTCTGCCATGAGTTAGACATCAAAAAATTAGAACAAACCAAAGGATTTAACTTATATACACTGATAATGTATTTTAACGTTATAAGTTTATCTTAATATGATGTAGCAGTTTTATTTTTCAGGTTACTAACCTCATAATCTATGGAAAATTATTTATAATTATCTTTTAAATGATACGATAATTATTTGTTTCTCTTGTTTTCTGCATATTCTTTTAGGTGACGTGACAGTCTTTTGTTTCTCTTGGCCCGTTTACTTTGGCAAGCAACTAAATTAATTTTGGTGAATTATGTATATATGCAGTATACCTGTAGTATTAGTAAATGGTACATTTCACCGGCATTTTATATTATCATGGGGAGACGATAGAGGAAAGATACATGAAAATGGAGAACTTTTAACACTCTCCTTAGATAAGCAATCTGGATCAGGATTTCAGTCCAAGAAAGAGTATCTCTTTGCCAAAATTGATATGCAAATTAAGCTCGTCCCTGGAAATTCAGCTGGCACTGTTACTACTTTTTACGTAAGTGCTCTCTAATATCACGTTTTTACTGTGATTATGTATGTTAACTTTGAGCTGAGGGTCTATAGGAAATAGTCTCTCAACCTACACAAGGTAGGGATAAGGCTGCGTATCCAGGGGCGGATCTACCTTGCGGCCTACGGGTTCACATGAACCCAATAGCTTTTGTCCAAACAATGTAAATGTGTTCGAAAAATTCACTAAATATGTATAAATGTTTGAATGTGAACCTAGTTACTATTGAAGATTTACTCGAGATCGTTGCAGGAACCCATAAACATTAAATCTTAGATCCGCCTCTGTGCGTATCTCACTATCCTCCCCAAACCTCACTTATAAAATTACACTAAATTTGTTGTTGTTGTTGCTCTCTCTAATATAACGTTTAATTGGTTGAAAATAAATTCAAAAGAAAATTGACAAAAATATTTTATGAATAATGTCACCTTAATATTTTTTTTGTCTTTGCTGCAGCTGTCATCACAAGGCAACAAGCATGATGAAATAGATTTTGAATTCTTGGGAAATTCCACAGGAAATCCTTATACTCTTCATACTAATATTTTCAGTCTAGGCCAAGGCAATAGAGAACAACAATTTTTCTTGTGGTTCGATCCTACTGCAGATTACCATACCTATTCAATCCTTTGGAATCCAAAATGTATTATGTAAGTTATTATCAGTAACTTTTTTGCTTCTGATTTCTTTTATAGCCTGTTTGGTCAATTTTTTTTTTCTTTTGCGCTCAAAAATGTTTTTTTTAAAGTTAAGGTATTTGGCTAAATTTTTGGTAGGGAAAAAAGGTGTTTTTGAGTAGAAGCAGAAAATGTTTCTCAGAAGCAGAAAAAATAGCTTCTTCCCAAAAGCACTTTTTTAAAAAGTACTTTTGAAAAAATTAACTTAATAACTCGTCTGGCCAAACTTATAAAATAAACTTATTTTGAAAAGTATTTTTGATGATAAGCAGTTTGTGTTTGGCTTATTAAGTTGAAAAACACTTCTGAACAACAGTTAGTGTTTGACCAAACTTTTAAAAAGTGTTTCTAAGTGTATCTTTTTCAAAAATGATTTTCAGAAAAGTGTGCTTCTCCAGAACTACTTCTGTTTCAATTCAAAAATACTTTTTTTTTCTATAAAAACTTGGCCAAACACTTCAACTTTGAAAAAAATATCTTTTTTTAAGAAAAAAAGTGATTTTGGTCTTGGAAAAGCTTGGCCAAACAGGCTATTAGAAACACTTTTTAAGAACTTGGTCAAATACTAATTGTAATTCAAAAGTATTTTTCAAATTAATTAGTCAAACACAAACTATTTCTCACTAAAAGTACTTTTCAAAATAAATTGATTTTAAAAGTTTGGCTAAACAGACTATTAAATATGTTGAGTACTTTGCTTTAGAAATTTTGATCTTGACCCCCTTTTTTGAGTACTTTGGTAATTACATTGCAATTAGTAACTTAATTTCCAAGATTCTTTAATGGGATCTTTCCTCATTTATTCTACTTTTTTTTTTCCAATGTAGATTCTATGTTGATGGTACACCAATTAGGGAGTTCAAAAATGCAGAAAAAATTGGTGTTCCATTTCTAAAATACCAACCAATGAGACTATACTCAAGTCTATGGAATGCAGATGATTGGGCTACACAAGGTGGTCGTGTTAAAACTAACTGGAAATTAGCACCTTTTATTGCTTCTTACAAAAATTTTACTTATGAAGCCTGCATTTATTCAAGATTAACTAGTTCGTCTTCGTGCAATATCAACTCTCCTCCTTTTGGTAACAACGCGTGGCTAACACACGAATTGGATCGAAGAAGTCGAGCAAAAATGAAAATTTTGCAGAAAAAACATATGATTTATGATTATTGTAAGGATAAATGGAGGTTTCCTAAAGGACCTGCTCCTGAATGCAAGCTTCAATAA

# >NtXTH29

ATGGCAAGGTTTTCATCTTCTTCATCTAGGTCTAGGTCTTCTCTTCCATACATTATATTGCTCTTCGTTGCTGCCCTTTTTGTCTTTAAGGTAAGAATTTTAATAAATTTCTTAATTATTATCTTAAATATTGTATAATCATCCTTCATTTGCATCGTTAACTCGTATTAATATGATAATTTAGTCGATATAATAATTTGATTTTGTTTTTTTAAGATTGTGTAAAGTGCATATTTTCTTTTTGTGTTTTTCTTATAATCGTTTCAATTTTATCGAATGTCCAAACCATGTTTTCTTTTCGATTATAAAACACACATCTCATTTCCTCTTTTTTTTTTTTTTTTTTTTTTTTTTTTTTTTTTTTTAATCCTTGAAAATAAAGATCCCAATAGATGAAGATTATGCTTAAAACTAGGGATATGAATTCAGTGTCTCCTATCTCTATCTCCCTCTAGCATAATTGCATAGTCATGTACGAAACTAGGATTTTGAGTTTATTAATTCCGAATTATTGAAAAGGTAAATCATTCGGTTTCGGATAAATTATTACATATTAAGTGGACTTGTTTAACATAAATATAAGATCTTGGTCAAAACTACTGCATTTTACTGAACTCGTAAGCAGAATTGTAGCTACAGCCACGGGTCCAGGGGTGGATTTATAGCCTATTATATAGGGCACGTGAGCTCATGGTCTTTCGGTCAAACTATGTATTTTATGTACATATTTTTTAGAATTGATCTAATGTTATCTGCTGACACTCATGCTTCAAAAGAGGTTAAATGTTGTACTTTGTTGAATGTTGAGTTATTTACCTAGATGTGTAGGGATCAATTCACACTTAAAATTTTTTTTAGTGGTGCACTCATTTTATAATAATCCTAGATTCGCCTCTGCACGGGTCATGTCATAATCATGTTTAATTTGTGGAATTAATTACATGAGCAGATAGATGTTATCATATCTCAGTCGTTTAGTTCAGCCCGTCGCAACCTGGAGAACACCCCTAATCATATCTTGGTGAAGTCTAAATCCCAAGAAACTGATGACAGGTACACAATTTTTTTCCTTTTTCAAATTTTTATTTGCTTTTTATTTTTATTTTTTTACAATACTTAATTAGACCTTATTTTCACATTTCCCAATATTGAATAGTATTTGAACTGAAAACCGAATTATATATGTTGCGACTGCTTATCCTTTTTAGGACTAGAGTTGGGCATGATCATGTATTTTTTTTTTTTACTTAAGGCTTTGAGTTAAATTGCAGTTGTCAATTACTAATACGGAGTAGTAAACACTAGAAAAAGGTTATGTATTAATTTTTGCATTACTTAATTAATAATATTGGTTAACAAAATAATATGTGAGAACTGCTCTGCACATATAACAAATTTTACACTAATGAGTTAGTCATCGAAAAATTAGAATAAACCAAAGGATTTAACTTATAAGGCGTAATGACTTCCTGACCACTTAAACTTGTAAGACTTTTGAAAGCCGATACACAAACTTTGAACTTTCCCATTTGAACACTCAAACTCGTGAAACCCTTAACTAATAAACACATTTGACCCTTGACCCTACGCGCGTGCATTACACATTCTCATACGTGTCCATCCAGTCAGCAAATGACCAATGGGAATGTTACACGTCAAGGGCGCGAAAAATAACGCCATAGCTGTGCTGGAATAAATTTATTAATTTCTAATTTTTTTTTCTATTTCCCTTATTTTCTTCTTCACCATAGTTGTGCAACTTTTACCTAACTCAAGGTACCAAAATGACCCAGCAAACCAAATTAACTACGAAGAGGGCCATAAATAATTTACACAGTACAACGGCAGAAAAAATCGAACACAAAGGAATAATAATGGATACTTAGAACTGTTCAAGTGAATTAGAGAAAACGAATAAAAACATGGATAATATATGATCAACAGAAATCTTTAAATTGTACCATGAACCGGAAAAAAAACAACTCGCCAGCGATCTCGAACGTCCTCAACACTTAAACCGAACAACCACAAAAATATTTTTGTCCCTTTGTTTTTTCGCTCACTTCCCCATCTCCTTTTTCTCTCATTCTNNNNNNNNNNNNNNNNNNNNNNNNNNNNNNNNNNNNNNNNNNNNNNNTGTTTATCTTGCAGCCGTTTCTGGTGGTAGGCGGCTAGCTTTTAGATTGAGAACAAGAAAAGCTCAAAATTCGAAGGGGGGGAGGCGCTATTAGGCGTAGATGGGGAGGAGGGACTGATTGGGTTTGTTTGGTCGTTGCTGTGGGTTATGGGTGAAGCCTTGTTTCTGGTCGTTGGGGATGGTGCAAAGATACAGGAGTGGTATGTAGAAGACGAGATCCAGGGGAGTGTTTTTATTATGGAAATCGGCGGATGAGATTTCTTCTTTTTTGGTTTTCCTTATAGTTTCTGTTTACTTTTTTGATTTTACAATCTTTTTAATGAATAGTAACAGATACACGCGTCATTTGAATGTGACTTGTACGCACTATATCCACGTAAGATAAGCTACCGCCACATAAGCATAGTCAATGGTCAAAGGTGTTTATTAGTTATAGGTTTCACGAGTTTGAGTGTTTAAATGGGAAAAACTAAAGTTTGTGTATCGGCTTTCAAAAGCCCTACAAATTTAAGTGTCCAGGAAGCCATTTTGCCTACTAATAATGTAAAGTATTTCTACATTATAAGTTTATCTTAACATGATATAGCAGGTATAATTTTCAGGTTACTAATCCCACATTTTACAGAGAATTACCTATAATTATTTTTAAGTGACCCTGTAGGTCATATGCCCTAATGCAAATATTAGTGGCTGACCATAGCTCGAACTCATGACCTAGGTCACCCTTAGTGGTGCAGTATTTCCCTGGATAGCAGCGGAGCCAGGATTTTCATTAAGAGGGGTCAAAATATAAAGAAATAAACTCACCAAGAAGTCAAGGGGTGTCAATACATAGTATACATATACATATTTTATATTACCTAACTACACAGTGTAATTTTCGAACAAAGGAATATCGGTTGACACCCCTTAAGTGCATGTGGCTGCGCAACTGGCCCCGAACCTTGCGTAAACACTAGATACTTAGTGTATCGGGCTGCCCTTTTATCTTTTATATGACCCAATAGTTTTTTAGTTCTCTTTGTTTCTTGCACATTCTTTTAGGTGACCCGATAGTCTTTTGTTTCTATTTGTCCTTTTACTATGGCAAGGAACTGAATTTATTTTTGGTGAATTATGTATGCAGTATACCTGTAGTATTAGTAAATGGTACATTTCACCGGCATTTTATATTATCATGGGGAGACGATAGAGGAAAGATACATGAAAATGGAGAACTTTTAACACTTTCCTTAGACAAGCTATCTGGATCAGGATTTCAGTCCAAGAAAGAGTATCTCTTTGCCAAAATTGATATGCAAATTAAGCTCGTTCCTGGAAATTCAGCTGGCACTGTTACTACTTTTTACGTAAGTGCTCGATAATATAACGTTTCTAGTGTATTTCTTTCTCTATATTGTTGTGATTATGTATGTTTGCTTTGAGTCGAGGGTTTATCGGAAATAGTATCGCTACCTGCACAAGGTAGGGATAAGGCTGTGTATTCAGTATTCACTTTCCTCTTCAGGCCCTACTTGTGAGATTATATTGAGCTTATTGTTGTTGTTGCTCTCTACTATAACGTTTAATTGGTTAAAAAGAAATTTTAAAAGTATTTTATGAAGAATGTCACCTTAGTATTTTTTGGGGGCTAATACAACGTTTTTATTGCATTTCTTTCTCTATGCTGTTCTGATTATGTATGCGTGGTTTGAGTTGAGGGTCTATCGGAAATAGTCTCTCAACCTGCACAAGGTCCATAAGGCTACGTATCCAGGGGTGGATCCACCTTGCGACCAACGGGTTCACATGAACACAATAGCTTTTGTCCAAATAGTATAAATGTGTTCGAAAAATTCACTAAATATCTATAAATATTTGAATGTGAACCCAGTTACTATTGGAAATTTACTCGACGTCGTTGCAAGAACCCATAAACATTAAATTCTAGATCCGCCTCTGAGCATATCTGACTATCCTTTTCAGACCCCATTTATTAGATTACATTAGGTTTGTTGTTGTTGCTGCTCTCTACTGTAATGTTTACTTGGTTGAAAAGAAATTCAAAAGAAAATTGACAAAATTATTTTATGAATAATGTTTTTTTGTCTTTGTTGCAGCTATCATCACAAGGAAACAAGCATGATGAAATAGACTTTGAATTCTTGGGAAATTCAACAGGAAATCCTTATACTCTTCATACAAATATTTTCAGTTTAGGCCAAGGCAATAGAGAACAACAATTTTTCTTGTGGTTTGATCCTACTGCAGATTACCATACCTATTCAATCCTTTGGAATCCAAAATGTATTATGTAAGTTATTATCAGTAACTTCTTTGCTTCTGATTTCTTTTATAGCATGTATGGTGAGGTTTTTTTTGGCCAAAAGTATTTTTTTCAAAGGAAAAAAAATTATTTCGAGTAAAAGCTGCAATTTTGAGAAGCAGAAAAAAGTAGTTTCTATCAAAAAATATTTTTTTTTGAAAAACACTTTTGAAAAAATTAAACTTAAAAGCACTTTTTAAGAGCTTGGTCAAACACTAATTGCCGCTCAAAAGTATTTTTCAAATTAATTCGTCAAATCCAAACTGCATCTCACTAATAGCCTGTTTGGCCAAGCTTCTAAAATCTATTTATTTTGAGAAGTGTTTTTTTCAAAAGTGCTTTTCAAAAACGTACTTTTGGTGAGAAGTAGTTTGTGTTTGACTAATTAATTTGAAAAGAATTTTTGAGCAACGATCAGTATTTAGCCAAGCTTTTAAAAAGTGCTTCTAAGTGTATTTTTCTCAAAAGTTCTTTTCAAAAAAGTGTTTTCAAAGAGAAACTACTTTTTCCTGCTTCTGTTTTTGCTTCTACTCAAAAATACTTTTATTTCTTCAAAAAAATTGGCCATAACACCTTAATTTTGGTTATAAAAAAAAAGCATTTTTAACAAAAAAAAAAAAANNNNNNNNNNNNNNNNNNNNNNNNNNNNNNNNNNNNNNNNNNNNNNNNNNNNNNNNNNNNNNNNNNNNNNNNNNNNNNNNNNNNNNNNNNNNNNNNNNNNNNNNNNNNNNNNNNNNNNNNNNNNNNNNNNNNNNNNNNNNNNNNNNNNNNNNNNNNNNNNNNNNNNNNNNNNNNNNNNNNNNNNNNNNNNNNNNNNNNNNNNNNNNNNNNNNNNNNNNNNNNNNNNNNNNTAAAAAAAAAGAATTTTTAAAAAAAAAAAAAAAAGCTTGGCCAAATAAGTTATAAAAGTACTTTTCTAAAAAATACTTTTAAGAAAAGTACTTTTCAAAATAAATTAATTTTAAAATTTTGGCAAAACAAACTATTAAACATATTGATTACTTTGCTTTAGAAAATTTGATCTTGACCCCCTTTTTTGAGCATTTTGGTTATTAAACTGCAATTAGTAACTTAATTTCCAAGATTCTTTAATGGGATTTTTCCTCATTTATTCTTCTTATTTTTTTCAATATAGATTCTATGTTGATGGTACACCAATTAGGGAATACAAAAATGCAGAAAAAATTGGTGTTCCATTTCCAAAATACCAACCAATGAGACTATACTCAAGTCTATGGAATGCAGATGATTGGGCTACACAAGGTGGTCGTATTAAAACTAATTGGAAATTAGCACCTTTTATTGCTTCTTACAAAAATTTTACTTATGATGCTTGCATTTATTCAAGATTAACTAGTTCATCTTCGTGCAATATCAACTCTCCTCCTTTTGGTAATGACTCGTGGCTAACGCACGAATTGGATCGAAGAAGTCGAGCAAAAATGAAAATTTTGCAGAAAAAACATATGATTTATGATTATTGTAATGATAAATGGAGGTTTCCTAAAGGACCTGCGCCTGAATGCAAGCTTCAATAA

# >NtXTH30

ATGATGAAAACTTCAAGTTGTATGTTTTCTTTCTTGTTTCTGAGTTTCTTGGTGTTGGTGGCTTTGGCAGAAAATTTCAACCAAGAATTTGATGTTACATGGGGTGATGGCAGGGTAAAAATACTTGAAAATGGGCAGCTTCTCACCCTTTCCCTTGACAAAACTTCAGGCTCTGGATTTAGGTCAAAAAGACAATATATGTTTGGAAAGATTGACATGAAGATCAAACTTGTTCCTGGCAATTCTGCAGGCACTGTTACTACATACTATGTAAGTCCCCAATCATTTTATCATGCTTAACATATATAGTACTTATTTTCTTCCTATAGTTAAACTTATTATAATATGAATTTGAGTTAAATGCAATGATAGTATAACTTTTTACACGATAACAATATTTTAATCTATTAGAGTTCATACAATTAATACCTCTCATAGTAAAATTATTGTAATTATCTTAATACATTAATTATATTTTTTGTCAATGGCCAATGAAAAATGCAGTTATCTTCACTTGGACCGACTCATGACGAGATTGACTTTGAGTTTCTAGGCAACCTAAGTGGAGACCCTTATATTCTTCATACAAATGTGTTCGTACAAGGCAAGGGGGAGAGAGAGCAACAGTTTTATCTTTGGTTCGACCCCACTAAGGATTTTCACACCTACTCTATTCTCTGGAATCCTCGAAGCATCATGTAAGCATACTTTTTATTTTTATTTTCTCTCGAGATATAAGATTTTTGCTGAACTAATTATTACATATCTCATATCTCTGCGTGCTGTTTTTATTCGTAACGTTTATGCTTCTTAGATTACTTAAAGATTTCCCATAAACTTGTGTAAAATGATAAATATTTAAAAGTTTCATGTAATTTTATTTGGATCAATTATGTCCTTTCTCTTCCTATTATGGAGTATATATTTGGATCAATTATGTCCTTTCTCCCCCTATCATTAACCCAGGGGCGGATGTAACCCTATACCAACGAGTTCAATTGAACTCATAACTTTCGATGCGGAGTATAAATTTACGTGCAAAACCTCACTAAAATCGTAACAAATTATAGATATGAACCCATAACTTTAAGGTTATAATTAGTTCGATCAATACTAAGAATCTTAAAAGTTGAACCCATAAAATTTACATCCTGGATCCGTTTTTGCATTAACCTATCTTAATTATTCAATATTATAAAATCTTTAAGATGGGAGATCACTCAGAGACACACATATTTTCAGATCTAGAACATTAAAGGGTGGGACGGATGAGATCTCTCTATCCTTAATTAGATGTTTCATATTTGAACCATTGGAATGGAAAAATCAAGTGCTTTTTTCTCTAATGGACTATACAAGGCACAAATCTAAATGTACTTCTAAGTTTTGAATAATTCAGCCGCTAGCCAAATATACAAAATATACATTAATTATGTATAATATGTATATTATATATTAATATACAAAAAATATATAATTATCAATATTATTTTGAGAAGCAGCTATACTATATAAAAGTTCCAATCTAAGGTTAGTCCGGCCAGTAAAATTTTGAAACACTAGATACAACAACAACAACAACAACAACAACCTAATATCATAGCAAGTGGTGAAATAGTATTACTTATCTTAAATGTTCCTATTATTCCATGCTTAACTTGTATGTACAATGAATTAGTGGGAAAAATACAGTTAAGCAACTATTTGAAAATCACCAAATTAAAACTCATTATTAAGATAAGAATTTAACTTATATTCATTTATAGCATAATAAAATTTTGGCTGTAATTCTTTTAGTTAGAGACATAACTGCTAATTAGGCAAATTTATATCAGATTTTCAGTAGATGGGACGCCAATTAGGCAATTCAAGAATCTTGAAGCTTCAAGGGGAATACCTTATCCCAAAAATCAACCAATGTGGATATACTCAAGCTTATGGGATGCTGAAGATTGGGCAACAAGAGGAGGACTTGTCAAAACTGATTGGAGCAAAGCCCCTTTCATTGCTTCTTACAGAAATTTTAATGCCCAAGCATGTGTTTGGTCTTCTGGTTCTACTTCTTCTTGCTCCATAAATTCCACAGCCAATTCTTGGATAACTGAATCATTGGATAACTCTGGCCAAGCAAGGATTAAATGGGTGCAAAAGAATTACATGGTCTATAACTACTGCACTGATACTAAACGTTTCCCTCAAGGATTTCCCCTTGAATGCTCTCTAAATTAA

# >NtXTH31

ATGATGAAAACTTCAATTAGTTGTATAATTTCTTTCTTGTTTCTGAGTTTCTTGCTGGTGGTGATGGCGGCTTTGGCTGGAGATTTCAACCAAGAATTTGATGTTACATGGGGTGATGGCAGGGTAAAAATACTTGAAAACGGGCAGCTTCTCACCCTTTCCCTTGACAAAACTTCAGGTTCGGGGTTTAGGTCAAAAAGACAGTATATGTTTGGAAAGATTGACATGAAGATCAAACTTGTTCCTGGCAATTCTGCAGGCACTGTTACTACATACTATGTAAGTTCCCTATTATAAGTTAATCATGCTTAACATATATACTTTTAATTTCTTCCTATAAATGTAGCTCATCTTATTTTTGCGTAGTCAAATATTTCAGGCAACTGCTCTTGAGTCGTTTAATTGATTGATTAGTTGTTATTGTTTTTGTATGCTACTAATTATTCATGTATACACTGCCATCGTAAAAGAATTCTTAGAATATCAATTTTGTTTAACAAACTGTAACAAATAATCTGTCTTACATTCACGTTACTATGTCGTAATTCTCAATGATGATTATTTAATGTACTAGAGTATATTAATTATTATTTTTATCGGATCATTAATACCTCTCATTGTGAATTATTGTAATTACCTTAATACATCAATTATATATAAGCCACTTAATTATTACAGTTCAATATTGAGGACTTTCTAGTGTTGTACTTATATTTTGTGCCAATGGTGAATGAAAAATGCAGTTATCTTCGCTGGGACCGACTCATGACGAGATTGACTTTGAGTTCCTTGGCAACCTAAGTGGAGACCCTTATATTCTTCATACAAATGTGTTCACACAAGGCAAAGGAGACAGAGAGCAACAATTTTATCTTTGGTTCGACCCCACTAAGGATTTTCACACATACTCTATTCTTTGGAATCCTCGAAGCATCATGTAAGCATACCTTTTATTTTTGGTTCGACCCCCTTAATTTTATTTGGATCAATTATGTCCTTTCTCTCCCTTTTATGGAGTATATATTTGGATCAATTATGTCCTTTATCCCCCTATGATCACTACCCTATCTTAATTATTCAATATTATAAAATCAATATGAGAAACACAAATTTTCAGATCTAGAATGTTAAAGGATGACATGGATATGATCCCTCTATTTTCAATAAGATATTCCTTATTTGAATTATTGGAATGGAAAAATCCTAACAGAAAATGTTTTTTCTTAAATGGGCTCTACGAGGCACNATATATATATATATATATATATATATATATATATATATATATATATATATTATGTATTAATTAATATACAAAAAAAATATAATTATCGGTATTATTTTGAGAAGCAGCTATACTATATAAAAACTCCAATCTAAAGTTAGTCTAGCCGGTGAAATTTGGAACACTAGATAGTCATACAAACAAAATTCATATTAAGTGGTGAAATAATATTACTTATCTTAAATGTTCTTATTATTCCATGCTTAACTTGTATGCTACGTTGTGTGGACTCTTCAAAATGATGCGATCCTCCAAAAATATATTATTTTGGAAGATTCGACACTCACCCGGCAACATTTTCGAGAGTTCGAGCAACATAGCTTGTATGTACAATGATAGTGGGAAAAATACAGTTAAGCAACTATTTGAAAATCACCAAATAAAAACTCATTATTAAGATAAGAATTTAAATTATATATATTCACTTATAGCATAATAAAATTTTGGCTGTAATTCTTTTAGTTAGAGACATGTCTGCTAATTACTCATCTTCTTCTATTTATCAGATTTTCAGTAGATGGGACACCAATTAGACAATTCAAGAATCTTGAAACTTCAATGGGAATACCTTATCCAAAAAATCAACCAATGTGGATATACTCAAGCTTATGGGATGCTGAAGATTGGGCAACAAGAGGCGGACTTGTCAAAACCGATTGGAGCCAAGCCCCTTTTGTTGCTTCTTACAGAAATTTTAATGCCCAAGCATGTGTTTGGTCTTCTGGTTCTACTTCTTCCTGCTCCAGAAATTCCACAGCTAATTCTTGGATAACTGAATCATTGGATAACTCTGGCCAAGCAAGGATTAAATGGGTGCAAAAGAATTACATGGTTTATAACTACTGCACTGATATTAAACGTTTCCCTCAAGGATTTCCCCTTGAATGCTCTCTAAATTAA

# >NtXTH32

ATGATGAAATCTTTCTTGTTTCAGATGATGTTTTTGGTGGTGGCTTTTGCTGGAAATTTCAACCAAAATTTTGATATTACATGGGGTGATGGCCGAGCTAAAATACTCGAAAACGGACAACTTCTTACCCTTTCCCTTGATAAAACCTCTGGCTCTGGTTTCCGATCCAAAAATCAGTATTTGTTTGGAAAGATTGATTTGAAAATCAAACTTGTCCCTGGTAATTCTGCTGGCACCGTTACTACATATTATGTTAGTCCTTTTTTACTATGGAGCAACTATTGTTAAATGGTTATTTTTTGATATTTTATTGATTGAATATGGTATGTTTTACTTATGTTGAGTTTTAATGGTGTGAAAAAAATGCAGTTATCTTCAATAGGATCAAGTCATGACGAGATTGATTTCGAGTTTCTTGGGAATCTAAGTGGAGATCCCTATATTCTTCACACAAATGTATTCACACAAGGGAAGGGAAATAGAGAGCAGCAGTTTTATCTTTGGTTCGACCCTACTAAGTACTTTCATACTTATTCTATTCTTTGGAATCCTCAGAGCATCATGTAAACGTCTCTCTCTCTCACACACACGTACTCCGTTGATTACGTACGTGTATATGTTTTTGACATGTTTACTTAGTTGTGGTATTTATTTTGTTACCTTTCTTAAATTGTTAATAGTCAATAGACTTATGGTTGTTAATAATCAAAGATTTCACGTAATTCTGAGTGATTAGACGTTCTTTCTTTCGCCTAAAAAAACTTACCTTTCTTGATTATTCAAAATTAAACAAATAAATTATTTTGAATGATTTTTTTGGGATTCAACGGTTGCTGCATAAGTTAAATAAATTTTTAGCTTTAAGTGATTTCAGAATAATAAATCTTTGGAATTAATGCACTTAAAGCACAATGAAAGTAGTGGCGTAGCCATCCTAGGCAAAGGGTGGCCAAGCGCACACCCTTCCTCGAAAAATTCTATTGTTTATATAAGTTAAATACTACTCCCTCCGGTTCACAATAAATGACCATTTTGCTTTTTCATTTTGGTTCAAAATAAGTGTCTAGTTATGTAATCAAGAAATAATTCAATTTGTTTTTACAAAATAACCCCTATGTACATATCCCTAAAAAGTTTTCTTACTCCTCACATTAAATGTTTAATTAGGGGTAGTTTAGTCATAGTAGGTATTTTTGTATAGAATTTAGTATTTTCTTAATGGGCGTGCTAAAAGCAAATTGGTCACTTATTGTGAACCGGAGGGAGTATAGTACTTTATATGGTTATATGTTATATATTGGACACCCTTAAACAATTGAGTGAGGCGGTCCAGTAGTCCAAGTTGTTCAAAGTGACGTGTTGTTCCCGGGTTCAAAATTTTGCTCATTTTGTTTGCCAAAATTAAACGGAGACCACCATATGCGGTCTATTTGATTCTTTCAATCTAAAAAATTTCCTAATACTAACTCGTAAAACTTAAAATTTGTGTAAAAATAAACAACTAATCTTAAAAGTAATTTTTAACTAAAAGATATTCTTAATTATTTATAAGTCAAGCTCCACAAACATTTCTTCAATAAAAGCTCCTTACATACTACTAGCCCTCTCGACTGTCTTTTTTCTTTGGTTTTATGCTTACTTTTATTAGTGAAATTTCTAATGTTGTTTTTTTGTTATTTTAATTTGATTTATAAAACTATAGTTCTATGAAAAAGGCTTTTTTTTTTTATTGTTTAGCTAAATATTGCTTAGTTTGACATAAAAAAATTTGTACACCAATCGGTTATCCTATTTAACTTATGGTTAAGATATGTATCACTTTTTGTTTCATTTAATTTTCACAAATACTGTGTGCCATTTAAATTTAAAAAAATAAATTAAAAGTGTCCTTTAGGTCCCCAGTTACATTATTATAAAAGAAATTACTTTAAGTATTTTTTTTTCCTTCAATTTCTATCATATATGATGATTACTAATTACTATTCTTTTTCCTAATTTATTAAAATAAATCAGCTTTTCAGTAGATGGGACACCAATTAGGCAATTCAAGAATTTAGAAGCAAGTGGGATACCTTATCCAAAGAACCAACCAATGTGGATATACTCAAGCTTATGGAATGCAGATGATTGGGCAACAAGAGGAGGATTAGTTAAGACTGATTGGAGCAAAGCCCCATTTATAGCTTCTTACAGAAATTACAATGCCCAAGCTTGTGTATGGTCTTCAACTTCTTCTTCTTCCTGCAGCCCCAACAACTCCACAGAAAATTCTTGGCTAAGTGAATCCTTGGATAACACAGGCCAATCTAAGATTAAATGGGTGCAAAATAATTACATGATTTATAATTATTGCACTGATACTAAACGCTTCCCTCAAGGATTTCCTCCTGAATGTTCTCTCAATTAG

# >NtXTH33

ATGATGAAATCTTTCTTGTTTCTGATGATATTTTTGGTGGTGGCTTTGGCTGGAAATTTCAACAAAGATTTTGATATTACATGGGGTGATGGCCGAGCTAAAATACTCGAAAACGGACAACTTCTCACCCTTTCCCTCGATAAAACCTCTGGCTCTGGTTTTCGGTCCAAAAATCAGTATTTGTTTGGAAAGATTGATTTGAAAATCAAACTTGTCCCTGGTAATTCTGCTGGCACCGTTACTACATATTATGTAAGTCCTTTTTCCTATATGGAGCAACTACTTTTAGGAGCTCTAATCATTTTATTTTTTGATATTATATTTATTGAATGGTAAGTTTTCAGGTGTTTTATTTATGTTGAGTTTTAATGGTGTGAATAAAATACAGTTATCTTCAATAGGATCAAGTCATGACGAGATTGATTTCGAGTTTCTTGGGAATCTAAGTGGAGACCCCTATATTCTTCACACAAATGTATTCACACAAGGGAAGGGAAATAGAGAGCAGCAGTTTTATCTTTGGTTCGATCCTACTAAGGACTTTCATACTTATACTATTCTTTGGAATCCTCAGAGCATCATGTAAGCATCTCTCTCTCACACACACACGTACTCCGTTTATTATGTATTATACGTGTATATGTTTTTCACATATTTACTTAGTTGTGATATTTATTTAGTTACCTTTCTTATATTGTTAATAGTCAATAGACCTTTGGTTGTTAATAATCAAAGATTTCACGTAATTCTGAGTTATTAGACGTTCTTTCTTTCACCCCAAAAAACATACCTTTCTTGATTATTCAAAATTAAACAAAAAAAATATTTTTAATGATTTTTTTGGATTTAACGGTACTGCATAAGTTAACCAAAATTTTAACGTTAAGTGATTTCAGAAGAATAAATCACATGAAATGAAATTGTCTCGAAAGACCTACGTCAAATCCTTGAAATCAATACAGACTTAGCTAAAAATAAGATACAATGGAAGAGAAAAATCCATATAGGCGATATTTGAGAATAGGTTATAGACTTGGTAGAGAATTTATTATTATTCTTATTACTACTACTACTTCAGGTTAAATTTCACAAATGGTCATATAACTATGACTTTTTTTTACCAAAGACATATAACTTTATTTCCTCACACAAAAATTATATAACTATGACTTTTTTTCGCCAAAGTCATATAACTTTGTTTTCTCACATAAAAATCATAAATCTTTCATTAACTAACACAAAAATCATAGTGGTCGAAAAATAAATTTTTCGGTAGTATTTTTTTATTTTTTTATTTTCAGTCTAATAAATAATTATCCAATNTATATTTATTGGCTAGCAAATGCAATTATTTATATATATATATATATTTATTGGCTAGCAAATGCAATTAGTTTCGACCGATCGGTTAATTTATATTTTTTTCTTAAAATAGGAATGACCCAACCCACCCTGACTGAATACACATATAAAATAATACCAAAAGTGAATTTTTCATCCATAAAAAGTTTAGTTCGGAATGCATGAGATTCTAACAAAAAAATAAAGATATAAAGGTATAATTAAAGAGCACTTGAAATAAAAATACACAATTTGAATAAAAATTTTATAAAGAAAATAAAAGATAGAAGTATCATGTTTTGAAGGATGTACTATTCACTCTTAGAATTATTTTAATTTCTATATATGAGTATTGGGTCGGGTGGGCCAGGACGGGTTAGGTCATCCCTATTTTAATTGTATTATTATTTATTAGACTGAAAATAAATAATAAAAAGTATAAAATAAGAAAATATTACTGAAAAATTATATTTTTCGATCACTATGATCTTTCAATGAGTTTGTAAAAGTTTTATGATTTTTGTGTGAGAAAACATAGTTATATGACTTTGATGAAAAAAAGTCATACTTATATAATTTTTGTGTGAGAAACAAAATTATATGACTTTAATGAAAAAAAGCCATAGTTATATGACCATTTGTGAAATTTACCCTACTGCTTCACTTGCACTTTTAGTTTATTTTGGTATTGATGATGTTGATGTGAATTGAGCTAAAAGAAATGAGAACTCATATCGCTATTGAGACATAATTGTTGTTAATGATTTCAGAATAAAGTACTCCCTCTGTCCAATTTGTGTCATAGTTCGAATTTTGAGAGTCAAACTTCTTAATTTTGATTTGTAATTCGAACAAAGAATCTTTAAATTTTTTAAAATAAAATTTATATATTTAAAAATTACATAAAAAGTACTATAATTTAAAAGACATATAGAAAATGGAGTCAAAGTTAAAATTGCATGACTCTCGAAATTCGAACATCGACACATAAATTCGGACAGAGGGAGTATGAATTAGTGGAGAAAACATATATGTGAGAAAATAATTATGTAAAGTGCCATACTTATGACCTAACTAGAATCGGTTATCCCATTTAACTTATGGTTAAGACATTTATCACTTTTTGTTTCATTTAATATTCACAAATACCGCGTGCCATTTAAATTTAAAAAAAGAAATTAAAAATGTCCATTAGGTCGGCAGTTGCATTATTATAAAAAAAAGTTACTTTAAGTGTGTTCTTTCTTCAATTTTTATCGTATATGATAATTACTAATTACTATTCTTTTTCCTAATAATTTATTAAAATAAATCAGCTTTTCAGTAGATGGGACACCAATTAGGCAATTCAAGAATTTAGAAGCAAGTGGGATACCTTATCCAAAGAATCAACCAATGTGGATATACTCAAGCTTATGGAATGCAGATGATTGGGCAACAAGAGGAGGACTAGTTAAGACTGATTGGAGCAAAGCCCCATTTATAGCTTCCTACAGAAATTACAATGCCCAAGCTTGTGTATGGTCTTCAAGTTCTTCTTCTTCCTGCACCTCTAACAGTTCCACAGGGAATTCTTGGCTAAGTGAATCATTGGATAGCACAGGCCAATCTAGGATTAAATGGGTGCAAAGTAATTATATGATTTATAATTATTGCACCGATACTAAACGCTTCCCGCAAGGATTTCCCCCTGAATGCTCTCTCAATTAG

# >NtXTH34

ATGTCTTCTTTTTCTTCTAAATTAGTACTAGCTCTTATTGTTAGTGCTTTCGCTATTGCAATTGCGGGTACTATTGACGAAAATTTTGAAATTACATGGGGTGAAGGCAGAGCAAAGATGCTAAATAATGGAGAGCTTCTAACTCTATCACTTGACAAAATCTCAGGCTCAGGATTTCAATCCAAGAATGAATATCTCTTTGGTAAAATAGACATGCAACTCAAACTTGTCCCTGGAAATTCTGCTGGCACTGTCACTGCTTACTATGTAAGTGTTTTTCCTTCTTTTTTTGTCTAAATCATTTTTTAGCTAGCTAGATTATATGCTTATTAAGTTGAAATGAAATTGATTTTTTGAATATATTGCAGTTGTCATCACAAGGACCAACACATGATGAAATAGATTTTGAATTCTTGGGAAATCTAAGTGGTGATCCTTATACACTTCACACTAATGTATTTAGCCAAGGCAAAGGCAACAGAGAGCAACAATTCCATCTTTGGTTTGACCCTACTGCTGATTTCCACACTTATTCCATCCTCTGGAATCCACAACGCATCATGTAAGTTAAAGAACTTGACCTTTTTTCTTCTTTTTTTCAAAATCTTTGACTTTTAGGGTCAATGTTGACTTGGGATTTTCTTGTTATTTTGATTGAACAGATTTTATGTAGATGGAACACCAATTAGAGAATACAAGAATGCAGAATCAATTGGAGTATCATATCCAAAGAAGCAACCAATGAGAATATACTCAAGTCTATGGAATGCAGATGATTGGGCTACAAGAGGAGGACTTATTAAAACTGATTGGAGTAAAGCACCCTTTAGTGCTTCCTACAGAAACTTCAAATCTGCAACTTCAACCTCTGCAGCCACTAGCAATTCATGGTTGAATGAAGAGTTGGATAATACAAGTCAAGAAAGGCTGAAATGGGTGCAGAAAAATTATATGGTTTACAATTACTGCAATGATTCCAAGAGATTTCCACAGGGATTTCCTGCAGATTGTGCTATGTAA

# >NtXTH35

ATGGCTTCTTTGTTAGCTCAATATTTGGTTTTTCTTGCCTTATGCTCTTTGCAATATCATAGTTTGGCTTATAATAACTTTAATCAAGATTTTGATGTTACATGGGGAGATGGTAGGGCAAAGGTTCTCAACAATGGAAAACTTCTTACTCTCTCCCTTGACAAAGCCTCTGGTTCCGGTATTCAATCCAAGAGAGAGTATTTATTTGGAAGGATTGATATGCAGTTGAAACTCGTACGTGGAAATTCAGCTGGCACTGTTACTACATATTACGTAAGTGTTATATCATAGTTATTCGAAGGGGAGGGACGTGTTGGAGTAACGGTAAAATTATTTTTCGAGTCGTGAAAGTAGTCACTAATGTTTGCATTAGGGTAGACTGTCTACATCATACCCCTTGGGGTGTGACCCTTCCTCGGATCCTGCTTGAATGCGGGAGTCTTTATGTACAAGGCTGTGCAACGGTAAAGTTGTCTCCGTTGTGACCTATAAGTCTCGGGTTCGAGTCATGGAAGCAGTCACTAATGCTTGTATTATGGTAGATTGTCTACATCATTACTTCTTGGGGTGCAACCCTTCCCCGGACCCTGCATGAATGCGGGAGTCTTTATGTATCAGGCTGTGCAACGGTAAAGTTGTCTCCGTTATGACCTATAGGTCTCGGATTCGAGCCGTGGAAGCAGACACTAATGCTTGTATTATGATAGACTATGCATATTATATCCCTTGGGGTGTGATCCTTCCATAGGCCCTGCATGAATAGGTTTTGTGTACCGGAATGCCTTATCTCATAGTTATTGGTGATGTTGTCATTTAATCTACATCAATAATTATGATCATATGGTAATTTTTTTTCTTATTTTTATATCATATGATTACTAAGATCTTGTTTTATTTTAATTTTCTATGTAGTTATCATCACAAGGGGCAACACATGATGAGATAGATTTTGAATTCTTGGGCAATCTTAGTGGTGATCCTTATATTATTCATACAAATGTTTACACTCAAGGCAAAGGTGACAAAGAACAGCAATTCTACTTATGGTTTGATCCAACTGCTGGTTTTCATACCTACTCCATTCTTTGGAACCCACAAACAATTATGTAGGTATCCTTTTACTATACAAACCAACAACAACAACCCAGTATAATCTCACTTAGTGGGGTCTGAGGAGGGTAGTGTGTACGCAGACCTTACCCCTACCCTGGGGTAGAGAGTCTGTTTCCGGCATCCTTCCCTCCAAAAACTTCCCACCTTGCTCTTGGATAGACTCGAACTCACAACCTCTTGGTCAGCATCCTTTTACTATACAAACCATATATCATTATTCATTAGAACCATTACTGCTCCTTCAATTTTATTTTATTTTGAAATAAAATTTACACAATTTGAAAACTATGGAAAAAATTACGTAAGATTTGCTAAGATAGTTAGTAATCAATGTATATTTTTTTTTTTAAAAAAAAAAAAGAGAAATTAAAGTTAAAAAAAATTAGCTTAACTCCTCAAATAGTAATGGTGTCATATATCCAAACTTATATATCTCTCTTGGTTTTTGCTCCTAAAGTTCTATAAATTTAACTTTATCTTTATTTTGACTTGCTCTATTTGACATTTCTTATAAAAATAACCAATATACTGGAATATTAAGCAAACTATAAGCTGAATAATGTTCATAATATTGATATAACTAGTTAGAATCAGGACGCACGAGAATTTAATTAAGGGCCCATTTGGCCATAAAAAAGTTTATTTTTTCCGATTTATTTTTTATTTTTTTCAGAATCAGTATTTGGTCATGAAAATTTTAAATTACACTTGCAGTTGAATTTCGAAATTTTTCAAAAATTTGAAAAACTCCAAAAAAGTTATTTTTCAAAATTTTCACTTCAAATCACTCACAAAAATTCAGAAACAACTCAAATTGTATTCATGTCCAAACACATCTGTGATTTTCAAATACATTTTCACTTATATTTTTTCTTTTTTTTTTTTAATTTCACAATTCTTATGTTCAAGCGCCCACTAAATCTAATAAATTATTGTTAATTCTTAATATTTGAATATGTAACAGCAACAACAACAACAACAACCACCATAACTATTGCTCCTATGCCTCAGTCCCAAAACAAATTGTCTTGGTTAATAGTTGAACATGACATTATTTTATTTCTGCTTCTACAAATTGACAAAAACTTATACATATATTTTTATGTTTGATTTACCAATTTTGTAGATTTTATGTGGATGGCACACCTATAAGAGTGTTCAAGAACATGAAGTCAAGAGGAATACCATACCCAAACAAGCAACCAATGAGAGTATATGCAAGTCTATGGAATGCAGATGATTGGGCTACTAGGGGTGGCCTAATTAAAACAGATTGGTCCAATGCTCCATTTATAGCCTCTTTTAGAAATTTCAAAGCCAATGCTTGTGTTTGGGAATTTGGAAAATCATCATGTAATAGTAGCACAAATCCATGGTTTTTTCAAGAACTTGATTCAACAAGCCAAGCTAAGTTACAATGGGTGCAGAAAAATTATATGGTTTATAATTATTGTACTGATATTAAAAGGTTTCCTCAAGGTTTTCCTCTAGAATGTAATTTCAACTCCACAACTAGTTAA

# >NtXTH36

ATGGCTTCTTTGTTAGTTCAATGTTTGAATTTTCTTGCCTTATGCTCTTTGCAATATCATATCTTGGCTTCTAGTAATTTTAATCAAGATTTTGATGTTACATGGGGAGATGGTAGGGCAAAGGTTCTCAACAATGGAAAACTTCTTACCCTCTCTCTTGACAAAGCCTCTGGTTCTGGTATTCAATCCAAGAGAGAGTATTTATTTGGAAGGATCGATATGCAGTTGAAACTCGTACGTGAAAACTCAGCCGGCACAGTTACTACATATTATGTAAGTATTATATCATAGTTAATATTATGTACTTGGAAGGAAAGTTTTGGAGCAACGATAAATGTGTCCCTGTGTGACCTATAAGTTATGGGAACAAGTCGTGGAAGTAGTCACTAATGCTTGTATTAGGGTAGACTGTCTACATCATATCCCTTGGGATGCGATCCTTCCCTGGGCTGCCTTATATCATAGTTATATTGGTGATGCTGTCATTTAATCTACACCAAGAATTACGATCATATGGTAATTTTTCTTCTTATTTTTATATCATATGATTATTAAGATCTTATTTTATTTTAATTTTCTACGCAGTTATCATCACAAGGGGCAACACACGATGAGATAGATTTCGAATTCTTGGGAAATCTTAGTGGTGATCCATATATTATTCATACAAATGTTTACACTCAAGGCAAAGGTGACAAAGAACAACAGTTCTACTTATGGTTTGATCCCACTGCTGGTTTTCATACCTACTCCATTCTTTGGAACCCCCAAACAATTATGTAGGTATCCCTTTACTATACAAGCCATATCATTATTCATTAGAACCATTACTGCTCCATCAATTTTATTTTATTTTGAAATAAAATTTACACAATTTGAAAACTACGGAAAAAATTACGTAAAATTTGCTAAGATAGTTAATAATCAAAGTATATTTGAAAAAAAAAATATGAGAAAGTAAAACAAAAAACAGTTTAACTCCTCAAATAGTAATAGTATCACATAACCATCCAAACTTATATCTCTCTCTTGGTTTTTGCTCCTAAAGTTCTATAAATCTAACTTTATCTTTATTTTGACTTGCTCTATTCGTCATTTCTTATAAAATTGACCAATATACTAGAATATTATGCAAACTATAAGCTGAATAATGTTCATAATATTGATGTACCTAGTTAGAATCATGACGCACAAGAAGTTAATTAAGGCCTCGCTTGGCCATAATTTGTTTTTTACTTTTTTTCAGAAATTTTCAAATCAGTATTTCCCATGAAAATTTCAAACTTCACTTGAAGTTGAATTTCGAAAGTTTTCAAAAATTTAAAAACTCCAAAAAATTATTTTTCAAAATTTTCACTTCAAATCATTCACAGAAACTGAAAAAATAGCTCTAAATTGTATTCATGTCTAAACATAACTATGATTTTTAAATACCACTTCATTTGATTTTTTTTTATTTTTTTCGTAATTTCACAATTTTTATGTCCAAACTCCCACTAAATCTATTAAATTATTGTTAATCCTTTATATTTGAATATAACAGCAACAACAATAACTATTACTATCACGTCTCAGTCCCAAAATAAGTTGTGTCCGCCTAATAGTTGAATATGACATTATTTTATTTCTTCCTTTACAAATTGACAAAGACTTATACATGTATTTTTATGTTTGGTTTACAAATTTTCTAGATTTTATGTGGATGGTACACCAATAAGAGTGTTCAAGAACATGAAGTCAAGTGGGGTACCCTACCCAACCAACCAACCTATGAGGGTATATGCAAGTCTATGGAATGCAGATGATTGGGCTACTAGGGGTGGCCTTATTAAAACAGATTGGTCCAAAGCTCCATTTATAGCTTCTTTTAGAAATTTCAAAGCCAATGCTTGTGTTTGGGAATTTGGAAAATCATCATGCAATAGTAGCACAAATTCCACAAAGCCATGGTTTTTTCAAGAACTTGATTCCACAAGCCAAGCTAGGTTACAATGGGTGCAGAAAAATTATATGGTTTATAATTATTGTACTGATATTAAAAGGTTTCCTCAAGGTCTTCCTCAAGAATGCAATTTCAACTCCACGACTAGTTAA

# >NtXTH37

ATGGCCAAATTCATAGCTTTTAATTCCTTGGTTTTGATCATTGCAACATTTGCATTTCATTGTGCTATAGTCAATGCAAAGATCTCAAGTAGCATGTATATCAATTGGGGTGCTCATCATTGTCAAATGCTTGGGGATGATCTTCAACTTGTCCTTGATAAATCTGCAG**GC**AAGTTTAGTTTTCTTTTCATATGTATGATATGTGCAAAAAAATACTTAATTACTTGTGTAGCCTTCCCTTGTGACTGCTCGAGCTGTAGGGGAATCCAGAATTTAGAGTCTGCCGGTTAAGTATACGTGTGATCTTACTATATATGTGGATATATTGAAAAGAATTTCAAATTATTTTTACATATGTATTCATATATAGTTTGCATCATGGAAAAAAATGGTCGCTTTAGTTGAAATCACAGAACCTGCTCTAAATCCACCTCTGGCTCGTGGCGAGTGTCTGCCCAAATAACTAAGTGAAAAAGGGGTTACAAACAATGACAACTTATGTTTGATCGAGCAGAAGATAACTAAATTTTGAATCTTCGGGTTGACAATATTACTGACTAATTTGGTTTTAACTTCAGGTTCTGGTGCGCAATCAAAGAGAACATTTCTCTTTGGTAGCTTTGAAATGCTTATCAAGTTGGTACCTAACAACTCCGCTGGAACTGTTACAACATACTATGTAAGTACAATTGTACAATATTTAAGTTTGAATTTTTCTTAATAAACCTTTTTGGAATTCAACTTACTACATATAAAAGTTGTTAGATTACTCAACCAGTTGTACAAAAAATTAGGAGTTTTAAACTATTTAAACTACTAGTGTATAATAAGACATTGTTTGTTTATTGATTCGTGCAGCTATCTTCTACTGGCACCAAACATGATGAGATTGGTTTCGAGTTTTTAGGAAATGTATCAGGACAACCTTACATTATCCACACAAACATTTACACCCAAGGTGTTGGAAACAAGGAGCAGCAATTCTATCCTTGGTTTGATCCAACTGCAGATTTTCACAACTACACCATTCATTGGAATCCTAATGCAGTCGTGTAAGTCTATTTTTTTCGACTATCTTGTTTGCTATATATATCATTATCAAACAGACGTCCAAGAGTATATATATATATATATATAAAGAGCTAGATAATCTTGAAATTTGATGTGTCACTACTTATTTTTTCAGATGGTATATTGATGGTATTCCAATTAGGGTATTTAGAAATTATCAACTCAAAGGAATTCCATTTCCAAACCAACAAGGAATGAGAATATACTCTAGCCTTTGGAATGCAGATGAATGGGCAACAAGAGGTGGACGTGATAAAATTGATTGGACAAATGCACCATTTATTGCAACATATCGTAAGTTTAGGCCAAGAGCTTGTTATTGGAATGGACCATTGAGTATTGTTCAATGTGCTATTCCTACTAAATCCAATTGGTGGAATTTTCCTTTATACAGTAAATTGAGTGCTCCTAAAGTGGATCAAATGAACTCAATTAGGAGCAAATACATGATTTATGATTATTGCAAAGATACTACACGATTTAAGGGAGTTATGCCTACTGAGTGTACATTGCCACAAAACTAG

# >NtXTH38

ATGGCCAAATTCATAGCTTTTAATTCCTTGGTTTTGATCATTGCTACAATTGCTTTTCATTGTGCTATAGTCAATGGAAAGATTTCAAGTAGCATGTATGTTAATTGGGGTGCTCATCATTGTCAAATGCTAGGGGATGATCTTCAACTTGTCCTTGATAAATCTGCAG**GC**AAGTTTAATTTTCTTTTCATATGTATAATATATGCAAAAATTCTTAATTACTTGTGTAGCCTTCCCTTGTCAGCCTCGTTTGTTCCGATATTTTTTGGCTGCTATAGTGAAGTGCTGTTATAGAAATGATATATTATAACATTACACAAAACGGTTCCGAAAAATTTTGGCTTTTATAGTGAATAGTTGTTATATATAGAGATGATGTTATAGAGAGGCGTGACTGTATATATACATATTTTATATATAGTTTGAGTCGAAAATAATGGTTTCGATTGAAACCACAGAACCTGCTCTATATTTACCTCTGGCGGGAGGGGAGTGCCTCCCCAAATAATTAAGTGAAAAGGGGTGCCAAAATAAGAATGACATTTTTGTTTGATTGAGAAAAAGAAAAAAAAAAGCAGAATTTTTAAATTTTTAGGTTGACAATATTATTGACTAATTTGGTTCTAACTTTCAGGTTCTGGTGCGCAATCAAAAAGAACATTTCTCTTTGGTAGCTTTGAAATGCTTATCAAGTTGGTACCTAACAACTCAGCTGGAACAGTCACAACATACTATGTTAGTATATATATAGGCAAACCTCTCTATAACAATCTCGTTTGTTATGATGTTCTTTTGCTGCTATAGTGAAGTGTTGTTATAACGGACATATATTATAACACAACATAATAATCTTTTCCAACAAAAGCTTGACTTTTATAGTGAGTGACTGTTATATGGAGATGTTGTTATAGAAAGGTATGACCGTACAATATTCAAATTTGAATTTCTTAATAAGCAACTAACAAAATCAAAAAGGATGTTAAGAGTCAATCAATTGTACAAAAATTATTAGAATTCTATGTACTGATGATATTTTTATTTATTTATCATTTGCAGTTATCTTCTACGGGTACCAAGCATGATGAAATCGACTTCGAGTTTTTAGGAAATGTATCAGGACAACCTTACATTCTCCACACAAATATTTATACCCAAGGTGTTGGAAATAGGGAGCAACAGTTTTATCCTTGGTTTGATCCAACTGCTGATTTTCACAACTACACCATTCATTGGAACCCTAATGCTGTCGTGTAAGTCGATTTTTTCAGACAACTTTCTTCCATTATACGAAAACCAGCTTATATGTCAACTTTCCCTACTCTTAGTATCTATTTATTTATAAAAAAATTGGAGTGTATTTTGTATCACGAAATATACTTTTTGGAAAATAGTTTTTTCATAAAATAATCATTTTTTCGGTATTTGGTTGCAAATCCAAAAGGAGAGAATTGACTTCCCTCACTCTAGGAAGGAAGTCATTTTTCTCCAAAGATATCTCAATTCTTAAGTTTCAATCACCACCACTTAGACATTATTATTATCAATCTTTAATAAATATGATTTTTCGAAAAATATTTTTCACCATCCATCCGAATATGATCAAAAGCAATTTTCAGAAAGTAAATCATTTTCCGGAAAATGACTTTCATTATTCCAAATAGACGTGCAACCATATACAGTCAAACCTCTCTATAACAACCTCGTTCGTTCAGATATTTTTATTGGCTGCTATAGTGAGTGTTGTTATAGAGGACATATATTATAACATAACATAAAAATTGGTTTCGAAAAAAATTTAGTTTTTATAGTGAATAACTATTATATAGAGATGTTGTTATAGAAAGGTTTGACTGTATCTAATTAGAAAGCAAGAGAATCTTGAAATTTGATATGTCACTACTTTTTTTTTCCAGATGGTATGTTGATGGTATTCCAATTAGGGTATTTAGAAATTATCAATTCAAAGGAATTCCATATCCAAACCAACAAGGAATGAGAATATACTCTAGCCTTTGGAATGCAGATGAATGGGCAACAAGAGGTGGACGTGACAAAATTGATTGGACAAATGCACCATTTATTGCAACATATCGTAAGTTTAGGCCAAGAGCTTGTTATTGGAATGGACCATTGAGTATTGTTCAATGTGCTATTCCTACTAAATCCAATTGGTGGAATTCTCCTTTATACAGTAAATTGAGTGCTCCTAAAGTGGATCAAATGAACTCAATTAGGAGCAAATACATGATTTATGACTATTGCAAAGATACTACACGATTCAAGGGAGTTATGCCTATTGAGTGTTCATTGCCACAATACTAG

# >NtXTH39

ATGGCCAAATTTGTAGCTTTTAATTCCTTGGTTTTGATCATTGCAACAATTGCATTTCATTGTGCTATAGTCAATGGAAAGATCTCAAGTAGCATGTATGTCAATTGGGGTGCTCATCATTGTCAAATGCTAGGGGAAGATCTTCAACTTGTCCTTGATAAATCTGCAG**GC**AAGTTTAATTTTCTTTTACATACGTATGATGTGCAAATAATTCTTCCTTGTGTAACCTTCCCTTGTGACCTATTCAAGGCACAGGTGAATCCAAAATTTAGAGTTGGTCGGTTCAGAATAAGTATAATCTTATTATATATGTATACATTTTATATATAGTTTGAGTCGAAAACAATGGGTTCAATTGAAATCATAGAATATGCGTTAGATCTACCTCTGTCTCGAGGCGATTACCTGCCCAGCTAATTAAGCGCAAAGGGGGCGAAAAAACGAATGACATTTTTTATTTACCAAAAGATAATTAGAATTTTAAATTTTTTTGGATTGACAATATTACTAACTAATCTGATTTTAACTTCAGGTTCTGGTGCGCAATCAAAAAGAACATTTCTTTTTGGTAGCTTTGAAATGCTTATCAAGTTGGTACCTAACAACTCTGCTGGAACTGTTACAACATACTATGTAAGTATAGTTATACAATATTCAAGTTTGAAAAATAAAATTTACAACTTCAAAATTTTTGTTGGAGTCAACCAACTGTACAAAAACTATTAGAGTTTAAATTTTGGGCACTAACGATGTTTGTTTATTAATCAACTATTAGAGTTTAAATTTTGTGTACTAACGATGTTTGTTTATTAATCGTTTGCAGTTATCTTCTACTGGTACCAAGCATGATGAAATCGACTTTGAGTTTTTAGGAAATGTATCGGGACAACCTTACATTCTCCACACAAATATTTATACCCAAGGTGTTGGAAATAGGGAGCAACAATTCTATCCTTGGTTTGATCCAACTGCTGATTTTCACAACTACACCATTCATTGGAACCCCAATGCTGTAGTGTAAGTCGATTTTTCTAGACATCTTCCTTCATTATATGAAAACCAGCTTATTTGTCAACTTTCCCCACTCTTAGTGTCTATTTATTTCTAAAAAAAATTAGAGTGCATTTTGTATCACGAAATATACTTTTTGGAAAATAGTTTTTTCAGAAAATAAGTCACTTTTCCGGTATTTATTTGCCAGTTCAAAAGGGAGAAATGACTTCCCTCACTCTAGGGCTGAAGTCATTTTTCTCCAAAGATATCTCAATTCTTAAGTTTCAGCCACTACTTTAACCAACACTTAGACATTATTATTATTATTATTATTATTATTATTATTATTATTATTATTATTATTATTATTATTATTATTATTATTATTATTATTATTATTATTATTATCAATCGTTAATACTCAAAAGTTCCATCAAAGAAGGTACTAACTTGTTTGCTAATATTTCATCGGTCTTTAATAAATTTGGGTTTTCGAAAAATAAATTTCACTCTCCATCCAAATATGAAAAATATTTTTGAGAAAAAAATTATTTTCCGAAAAATGGCTTTTGTTATACCAAATAGACGTGCAACCATATATATAGTAGAAAGCAAAATAATCTTGAAATTTGATGTGCCACTACTTATTTTTTCAGATGGTATGTAGATAGTATTCCAATTAGGGTATTTAGAAATTATCAACTCAAAGGAATTCCATTTCCAAACCAACAAGGAATGAGAATCTACTCTAGTCTTTGGAATGCTGATGAATGGGCAACAAGAGGTGGCCGTGACAAAATTGATTGGACAAATGCACCATTTATTGCAAAATATCGTAAGTTTAGGCCAAGAGCTTGTTATTGGAATGGACCATTAAGTATTGTCCAATGTGCAATTCCAACAAAATCCAATTGGTGGAATTCTCCTTTATACAGTAAATTGAGTGCTCCTAAAGTGGACCAAATGAACTCAATTAGGAGCAAATACATGATTTATGACTATTGCAAAGATACTACACGATTCAAGGGAGTTACGCCTACTGAATGTTCATTGCCACAAAACTA

# >NtXTH40

ATGGCCAAATTCATAACTTTTTCCTTGGTTTTGATCATTGCAACATTTGCATTTCGTTGTACTCTAGTCAATGGAAAGATCTCAAGTAGCATGTATATCAATTGGGGTGCTCATCATTGTAAAATGCAAGGGGATGATCTTCAACTTGTCCTTGATAAATCTGCAG**GC**AAGTTTAGTTTTCTTTTCATGTATGATGTGCTAAAAAAAATGCTTATTTGCCTACCCTTCCCTTGTGACTGCTCGAGCTGTAGGAGAATCCAGAATTTAGAGTATATCCGTTCAGAATACGTGTGATCATATTACATGTATATATTTTGAAAAGAATTTTTAATTTATTTTGACATATGTATAAATATATAGTTTAATTAAAAAAATAATGACTTTAGTTGAAACGATAGAACCTGCTTTAGATCCGTCTTTGGCTAGAGGAAAGTGCCTGCCCAAATAATTTGTGCTTATCAATTTCAATTGACAATATTACTGATCTGGTTTTAACTTCAGGTTCTGGTGCGCAATCAAAAAGAACATTTCTCTTTGGTAGCTTTGAAATGCTTATCAAGTTGGTACCTAACAACTCCGCTGGAACTGTTACAACATACTATGTAAGTACAACTATACAATATTCTAGTTTTAATTTTCTTAATAAACCTTTTGGAATTCAACTTACTACATATAAAAGTTGTTAGATTACTCAACCAGCTGTTCAAAAAATTAGTGGAGTTTTAAACTATTTACACTGCTAGTGTATAATAAGACATTGTCTGTTTATTGATTCGTGCAGCTATCTTCTACTGGCACCAAACATGATGAAATCGACTTCGAGTTTTTAGGAAATGTATCAGGACAACCTTACATTATCCACACAAATATTTACACCCAAGGTGTTGGAAACAAGGAGCAACAATTCTATCCTTGGTTTGATCCAACTGCAGATTTTCACAACTACACCATTCACTGGAATCTCAATGCTGTCGTGTAAGTCGATTTTTACTTTTTATCTTTCCCCCAATACTTTTTAGAAAATATTCTCAGAAAATAAATTATTTTCCCGGTATTTAGTTGCCAGTCCTGGGATGACTACCTTCATTCACGTCGGGAGTTATTTTCTTTCACAAATATCTCACCGCTTAATGTTATATCAGCTTAATCAACACTTGGATATTATGTTTAATATTCAAAATTTTCATCAAACCATTATTATGTTTACTAATATTGTTATCAATCTTTAATAAATTAAATAATTTTATTTTCGAAAAATATTTTCCATCCTTTTGCCAAACATTGCAACATCTTCTAGAAAACAAATCATTCTTCAAAAACATTTTTCGGAGAATGACTTCCGCTATACCAAACATACGACGCGCATATATATNTCAGATGGTACGTAGATGGTATTCCAATTAGGGTATTTAGAAATTATGAGCTCAAAGGAATTCCATTCCCAAACCAACAAGGAATGAGAATCTACTCTAGCCTTTGGAATGCTGATGAATGGGCAACAAGAGGTGGCCGTGATAAAATTGATTGGACAAATGCACCATTTATTGCAACATATCGTAACTTTAGGCCAAGAGCTTGTTATTGGAATGGACCATTGAGTATTGGTCAATGTGCAATTCCCACAAAATCCAATTGGTGGAATTCACCTTTATACAATAAATTGAGTGCTCCTAAAGTGGATCAAATGAACTCAATTAGAAGCAAATACATGATTTATGACTATTGCAAAGATACTAAACGATTCAAGGGAGTTACGCCTACTGAATGTTCATTGCCACAAAACTAG

# >NtXTH41

ATGTTCAAAATTATGGCCAGCTCTCGACTTCTTTCTTTGGCTAATTTGTTCATTTTGGCAATTGCATTTCATTTGGTTTCAGTCAATGGTATGTTCTCAGATAACATGTATATTGGCTGGGGTGCCCATCATTCTTGGATGCAAGGAAATGATCTTCAGCTTGTTCTTGATCAATCCTCAGGTAAAGTTTCATTTACCTTTCTCATGTTTAAACATGTCGCATATGTTGCAACGGTAAAGTTGTCTCATTATGACCTATAGTTATTAATACGTATATTAAATAGTCTATCTACATCACACCCGACCTTGGGGTAAATGACCCTGCTCCAGAACGAAAAGACCTAACGGACTAGCTCTCTTCCTTAGTGCAACTGCCCAGTCATAATTATTTATTTAATAGTTAGATTGGAATTTACCCTGATGAAATCCCTTGTGGGACGTCTGGCGGAATGCTTGGCCTTCTGCGCTGGAGAAAAAAAAAGAAAAGAAATGTTGAACAAAAACGATCTTTAACTTATATATACTGACATTGTCTCCCTTTTCACATAAAATAAACAGATAAATCGGTATATCATTCGTCGGAATTTACTGAGTTCGACCATAGAGCTTTATCTAGCTTTTATTTTTTGAGTGGTTTCTTCGTTCTCTATTTGGATGGAAATAAAGGAAAAAACTTCCCTTTTTTCTCATCTTTATTTTTGTTTTATCCCTTCGACGAATTTAGGCTATGTTAATGTGCCACTTGCTAAATAGGCTAAGAAAAACGCTACCTGAAAAAACAAAATACAACTATTTTTATACTAGCAGTATAATTCAACTTGTTATATAACAAATTATCTGTCTTATTTTCATATTACTAATTGTTCTACTCATTAGAGTAGAAGTTAGTTAAACTTTTTGTCAAGAAACACTTGATATGACACAGCAAAAGTTTAATGTAATCAAACACTGGAAGTGAAACAGAATTCATAGATTGACTGGCTAGGAAGTTTACCAACCTCTAGCTAAATATAGGATAATCAAGATCTCATTTCACACATCTTTCTTTTTCTTCTTCTTGTTTTTTTTTTTGGGTTATATCACTCAACACTACTACTTTGGTTTTCATTTTCAGGTTCAGGTGTACAATCAAAAGGGGCATTTCTTTTTGGAAGCATACAAATGCAAATCAAATTGGTGCCTGGAAACTCTGCTGGAACAGTTACTGCATACTATGTAAGTTCCCTGTTGGTGGCAAAAGTAAAAATATCAAGAAATAGGTTAACTTCCTGATTAATTAGGATGTTATTCTAAACAACTATGACTGAAAAGCCTGATTTCTGGTTGTTAATTCTACAGTTATCCTCTACTGGTGACAAACACGACGAGATCGACTTCGAGTTTTTAGGGAATGTATCAGGGCATCCATATATTATACACACAAATATTTTTACTCAAGGTGCAGGAGGCAGGGAACAACAATTCTATCCATGGTTTGATCCAACTGCTGATTATCATAACTACACCATTCATTGGAACCCCAGTGCAGTTGTGTAAGTCGTTCTATTCCATAAATTTTCCTAATAGTCCCGATGCGGATCTAGGATTATAAGAATATGGGTACATACTAAAAAAGGAGAAAAAGAAAGTATTTAGTTGGAATAAATCCCTCTTCTTTGAGCAAATAACTCAGCATTCGACTAAATGCGTCACTCAACCTTTTGGAGCATGGGGCCAGCAAATAATATTAGATTAGTTCTAGGAAATATGTACATAAAGTACATAGTTTGGCGAAAAGACCATGTGTTCACGTGCACCGTAATTTGGGCCTAAATCCACCCCTATCTAACACTAAAAACACAAAACTCCCGTTTTCTCAAAATTCCTAAGTGCCTTATAGCTTCCATGGGCAGACGCGGATGTAGCATGTCTACCATGGGTTCAGCATATTTTCGACACAAAATATAGATATCTATGTAAAAGTTTAGTACTAAAATTTCAACAAATATTACATTCTGACAATAGGTTCAGTGGTAAGAACATTAAAAGTTGAACTCATCAAGTTTGAATCCTGAATCTGTCTCTGTCCTTAGATCACAAGCCAGGCAGTAGTTGACTCATTGTTTAACCATTCCTATTGCAGATGGTACGTTGACGATATACCAATCAGAGTATACAAGAATTATCAAAGCCAGGGAATTCTCTATCCGAACGCACAAGGAATGGGGGTTTACTCTAGCCTTTGGAACGCCGATAACTGGGCAACTAGAGGCGGCCTTGACAAGATTGACTGGACCAATGCTCCATTTATAGCCAAGTACAGAAATTTCGCGCCACGAGCTTGTCCCTGGTATGGACCAGGTAGCATTAGCCATTGTGCTGCTCCAACTCCAAATAATTGGTATACTTCTCCTGAGTATAGTCAATTGAGCTATGCTAAGCAAGGGCAAATGAATTGGGTAAGGAACAATTACATGATCTATGATTATTGTAAAGATACGACGCGATTCAATGGACAGATTCCTGGAGAATGTTTTAAGCCTCAATTCTAA

# >NtXTH42

ATGTTCAAAATTATGGCCAGCTCTCGACTTCTTTCTTTGTCTAATTTGTTCATTTTGGCAATTGCATTTCATTTGGTTTCAGTCAATGGAATGTTCTCAGATAACATGTATATTAACTGGGGTGCCCATCATTCTTGGATGCAAGGAAATGATCTTCAGCTTGTCCTTGATCAATCCGCAGGTAAAGTTTCATTTACCTTTTCTTTTGTATGTTTAAACTTGTCGCATATGTCGCAACTGTAAAGTTGTCTCAGTGTGACCTATAGGTCAAGGGTTCGAGCTAGGGAAGCAGCCACTAATGCTTGCATTAGGGTAGGTTGTCTGCATCATACCTTTTGGATACAGCCCCTTCCCGGACCCTGCATGAATACGAAATGTTTTATACATCGGGCTTCGTATGTCAAAAGTTTCTAATAGCATTTAATTTATATATAGTGACACTATCTTCCTTTTAACATAAAATAGACAGATGAATTGGTATATTTATTGAATTCGTCAAAACTTACTGAGCTGACCTCGGAGTTTTATCAGGCTTTCATTTTTTGAGTGGTTTCTTTGTTCTCTATTTGGATGGGAATAAAGCAAAAAAACTTCTCTTTTTCTATTCTTTATTTTTGTTTTATCCCTTTGACGAATTTCGACTATGGTAATGCGCCACTATTTAGGCGTAAGAAAAACTTTACCTGAAAAAGCAAAATGCACCTAATGTTTATTTTTTACCCTATCAATGTAATTTAACTCGCTATAACAAATTACCTCTCTTATTTTCATATTACTACTAATTGTTTCACTCATTATGAAATTACCTGTAAATATTTTACCCTGTTAGAGTATAGAAGTTAAACTTTTCATGATAAATGGTTTGTCAAAATAAGGCTCAGCAAAAGTTTAATCTAATCAAAAACTAGGAAGTAAAAACAGAATTCACAGATTGACTAGAAAGTTTTCCAACTTCTGCCTAAATATAGCATAATCAAGATCCCATTCCAACCCCTCCCCCCCCCACCCCACATTGATTTTTGCCTTATTGGATTATGTCACTCAAAAAAAAATGCTACTTTGGTTTACATTTTCAGGTTCGGGTGTACAATCAAAAGGAGCATTTCTTTTTGGAAGCATAGAAATGCAAATAAAATTAGTACCTGGAAATTCTGCTGGAACAGTCACAGCATACTATGTAAGTGTTAGCTTAAATACTCAATAGACAACACTATAACAAATGATCAAAGCCTTTTTCCTGCTTAATTCTGTTTATAATTTTTTCAAATAGATACCATGAGAAGTGTCTGATTTCTGTTTATTTTTCCACAGTTGTCATCTACTGGTGACAAGCACGACGAGATCGACTTCGAGTTTTTGGGAAATGTATCAGGGCAACCATATATTATACACACAAATATTTTTACTCAAGGTGCAGGAGGCAGGGAACAACAATTCTATCCGTGGTTTGATCCAACTGCTGATTACCATAACTATACCATTCATTGGAACCCCAGTGCAGTTGTGTAAGTCGTTCTATTCCATAAATTTTCCTAATAGTCCAGGGGCGGATTTAGGATTTTAAGAACATGAGTACACCATTAGGATAAGAAAAGAAGTATATTTAGTGTGAATTGATCTCTCTTCCTTTGGGTAAATAACTCAGCATTTGACCAAATGCATCATTTAACATTTTTGGAAAATTGGAGCCAACAGATAATAATAGATCAGTTCTAGGAAATATATAATACATAAAATATCAAGTTTGACGAAAAGTCCACGGGTCCACGTGTACCGCAATTTGGGCCTAAATCTGCCCTTGTCTAACACTAAAAACACAAAACACCCAATTTTCTCAAAATTCCTAAGTGCCTTAGCTTCCACGCAGACATGGATGTAGCATGTCTACCATGGATTCAGTACATATTCGATACAGAATATAGATATCTTCGTAAAAGTTTAGTACTAAAATTTCAACAAATATCATATTCTGACAATAAGTTCGGCACCAAGAACCTTAAAAGTTGAACCCATCAAGTTTAAATCATGGATTTGCCGCTGTCTACAGATTAGTAGCCAGGTAGTTCTTGACTCATTGTTTAACCATTCAACAAATATAGATTCTGACAATAGGTTCAGTGCTAAGAACCTTAAAAGTTAAACTTATCAAGTTTAAATCCTGGATTTGCCGCTGTCTACAGATTAGTAGCCAGGCAGTTCTTGATTCATTGTTTAACCATTCATGTTACAGATGGTACGTTGACGGTATACCAATCAGAGTATACAAGAATTATCAGAGCCAGGGAATTCTCTATCCGAACGCACAAGGAATGAAGGTTTACTCTAGCCTTTGGAACGCCGATAACTGGGCAACCAGAGGCGGCCTTGACAAGATTGACTGGACCAATGCTCCATTTATAGCCAAGTACAGAAATTTCGCGCCGCGAGCTTGTCCCTGGTATGGACCAGGTAGCATTCGCCAATGTGCTGCTCCAACTCCAAATAATTGGTATACTTCTTATGAGTATAGTCAATTGAGCTATGCTAAGCAAGGGCAAATGAATTGGGTAAGGAACAATTACATGATCTATGATTATTGTAAAGATAAGACGCGATTCAATGGACAGATTCCAGGAGAATGTTTTAAGCCTCAAATCTAA

# >NtXTH43

ATGGCAATCTTTTTTCTCCATTTTCTTCTCTTGCTCATTGTTGTCCCTTCTACAAATGCTGGTTATTGGCCACCTTCTCCTGGCTATTATCCAAGTTCCAAGTTTAGGTCTATGAGCTTTTATCAAGGATTTAGAAACCTTTGGGGCCCTAATCATCAAAATGTAGATAATAATGGCATTAATATTTGGCTTGATAGAAATTCAGGTAGTTTTAATTACTCCCTTCTTTACTTGAATAGTATTACTACTTCTAATTAATTTCTTGTAACTACAAAAAAATCTGGCGAATCCATCGCTAACAGAATTTTCTAATAATCTGTCGCTAATCCGTAGCTAAATAACATTAGCAACAGATTTTGCAGTTTAGCTACAGAATATTTCCGTCGCTAATTTCAGTTAGTAGTATTATGGAAATTCTTGCTCCTCATTGATATATTCTTGGTGATGATATATAGGAAGTGGATTCAAGTCAATTAAACCATTTCGATCAGGGTATTTTGGTGCTTCCATTAAACTCCAACCTGGTTATACTGCTGGTGTTATTACAGCTTTTTACGTAAGAATTTCGCTTATATATATTTGTAGTTAGTACATTTTAGTTGATTTAATTCATTAATGAGTGGAATATATGGCAGCTTTCAAATAATGAAGCTCATCCAGGGTACCATGATGAAGTGGACATAGAATTTCTTGGAACAACATTTGGGAAGCCTTATACATTGCAAACCAATGTTTATATTAGAGGAAGCGGAGATGGGAAAATTGTAGGAAGAGAAATGAAGTTTCATTTGTGGTTTGATCCAACAAAGGAATTTCATCACTATGCTATTTTGTGGAGTCCTAGAGAGATCATGTAAGCACTACTTAATTTATCATTTGAATCCTCTTATTTATTTCATTGAAGTAAAGTTAATGATACAACGGGAGCCTTGTAGCAACGACAAAGTTGTCTCTGTGTAATCTATAGGTCACGTGTTCGAGCAGTGGATTAGCCATTTATACTTAAATCAAGGTAGGCTAGAGTACGGCCTTTTCTGAACCCTATGTGAATATTGGATGCTTTGTGCACCGGGCTGTATAAGTAAATTTAATGATAAGGAAATATTCTACCGACAAAAATCATTTGACACTAAAGTATGGTTGTCCTCCAATTAAATTTAGTCATGGTTGCTCACCAATGAATTTAAGGACATCTGTTATATTTTTTGATTACTTCAATTTCTTTTTTTAGTATCGAGGGGACCATGTTAAAGGATTAAATTCATGTTGTAGTCCTTTTTTCCATAATAGCTAAAAGGTCCACCATTTTTTTTTCACTCACATAGATTGAATGTGAGTCCAAAATATATTAGGCCCACACACACGCAAAAAATTGCATGTTATTTTATTTTATAGAATTTTCTAAATTTGTCAGAGGGTTTATCAGTTCTCTAACTTTACAATGTGGGTATAAGGTTTGCGAACAGCTATCCTCCTCAGATTTCATTTATGAGATTTTACTAAATTTTTTATTATTGTTATTATCGTAGAATTTCCTAAATCTGAAAACTAATTATATTGTTAATTTGATTGGTTTTGGTTCAGATTTCTTGTGGATGATGTGCCAATAAGGAGGTATGCTAGGAAGAGTATTGCAACATTTCCACTAAGGCCAATGTGGTTATATGGATCAATATGGGATGCATCTTCTTGGGCAACTGAGGATGGAAAATACAAAGCCGATTATAGGTACCAACCATTCTACGGGAAATTCACGAACTTTAAGGCAAGCGGTTGCACCGCCTATTCATCGCGATGGTGCCACCCCGTGTCCGCTTCACCATCCAGGTCCGGAGGCCTTACCAGGCAACAACGTCAAGCCATGAATTGGGTTCATAGTCACTACTTGGCTTATGACTATTGTCGAGACTCCAAAAGAGACCATTCCCTAACACCGGAATGCTGGCGTTAA

# >NtXTH44

ATGTCAATCTTTTTCCTCCCTTTTCTTCTCTTCCTCATTGTTCTCCCTTCTACAAATGCTGGTTATTGGCCACCTTCTCCTGGCTATTATCCAAGTTCCAAGTTTAAGTCCATGAGTTTCTATCAAGGTTTTAAAAACCTTTGGGGTCCTAATCATCAAAATGTAGATAATAATGGCATTAATATTTGGCTTGATAGAAATTCAGGTAGTTTTAATTACTCCCTTCTTTACTTGAATAGTATTACTACTTCTAATTAATTTCTTATCACTACAAAAAAAATCTGGATTTAGCTGCTAAGTTCGTCGCGAATGTCGCTAACAGAATTTTCCTTAAATAAGATTAGCAACAGATTTTTCAGTTTAGCTACAAAATATTTACGTCACTAATTTCAGTTTTTTTAGTAGTTATATAATTATATGGAAATACTTACTCCTCGCTGATATATATTCTTGGTGATGATATATAGGAAGTGGATTCAAGTCAATTAAACCATTTCGATCCGGGTATTTTGGTGCTTCTATTAAACTTCAACCTGGTTACACTGCTGGTGTTATTACAGCTTTCTACGTAAGAATATAATTTCGTTTATATATACTGTCAGTGTAAATATTTTTAGTTAGTACATTTTAGTTGAAATTAAGTTATTAATGAGTGGAATATATGGCAGCTTTCAAATAATGAAGCCCATCCAGGGTACCATGATGAAGTGGACATAGAATTTCTTGGAACAACATTTGGGAAGCCTTACACATTGCAAACCAATGTTTATATTAGAGGAAGTGGAGATGGTAAAATTATAGGAAGAGAAATGAAGTTTCATTTGTGGTTTGATCCAACAAAGGATTTTCATCACTATGCTATTTTGTGGAGTCCTAGAGAGATCATGTAAGCACTACTTAATTTATTATATGAATCCTTTTATTTATTTCATTCAAGTAAATTTAATGATAGAAATGAAAGTCTTAGAGCAACGACAAAGTTGACTTCGTTGGATCTATAGTTTACAAGTTCGAACTGTGGAATCAACTACTGATGTTTGCATTAAGGTAAGCTGATTATATCACACTTGTTGGAGTACGGTTTTTTTCGAACCCGGCATGTATGTCATATGTTTCGTGGACCGAGCTGTATAAGTAATGTTAATGATAAGGAAATATTCTACCGACAAAAATCATTTGACACTAAAGTATGGTTGTCCTCCAATTGAATTTAGTCATGGTTGCCCACCAATGAATTTAAGGACATCTGTTGTATTTTTTGATTACTTCAATTTCTCTTTTAGTATAGAGGGGACCATGTTAAGAGGATTAAATTCATGTTGTAGTCCTTTTTTCCATAATAGCTAAAAGGTCCACCATTTTTTTTTCACTCACATAGATTGAATGTGAGTCCAAAATACATTAGGCCCACACACAAAAAAAGTTTACATGTAATTTTTTTTGTGTGTAGAATTTCCTAAATCTGAAAAATAATTCATTATATTGTTATTTTGATTGGTTTTGGTTCAGATTTCTTGTGGATGATGTGCCAATAAGGAGGTATGCTAGGAAGAGTATTGCAACATTTCCACTAAGGCCAATGTGGTTATATGGATCAATATGGGATGCATCTTCTTGGGCAACTGAGGATGGAAAATACAAAGCCGATTATAGGTACCAACCATTCTACGGGAAATTCACGAATTTTAAGGCAAGCGGTTGCACCGCCTATTCATCGCGATGGTGCCACCCTGTGTCCGCTTCGCCATCCCGGTCCGGAGGCCTTACTAGGCAACAACGTCAAGCCATGAATTGGGTTCATAGTCACTACTTGGCCTATGACTATTGTCGAGACTCCAAAAGAGACCATTCCCTTACACCGGAATGTTGGCGTTAA

# >NtXTH45

ATGGCTAATTTATTCCTTCTTTCTTTACTTCTCATTTTCTTGTTCAATTCAAGCAATGCTCAGGGTCCCCTTTCTCCTGGCTACTATCCTAGTTCTAAGGTTCAATCGTTAGGGTTTAACCAGGGTTTTAGAAACCTTTGGGGTCCTCAACATCAATCTTTGGACCAAAGTGCTTTAACAATATGGCTTGATAAAACCTCAGGTCTTTAGTTCTTGTCTCTCTTTTATATATTCGTATGCATTATTATACTTATTAATTTTTGCAACTAAGTTATATACACCGATCATGATCAATGCTGTTTAACCTGTCATAATATACTAACTATCACTTTTATCGTAGAGATTTATTTACATTATAAGCGATATTTGATCGTGTGTAAATATCTCTTTTCATCATCCTTGCTCGAATTCTCCAAAAATATCATCGAGCGCGTGTTGGATCCTCCAAAAGGGGTATATTTTTGAAGGATTCGATATGGGTGTAACAACATTTTAGAGGGTACCCACAACATAGCCCTCCACTAGAATTTTTCTAATCACATCTTCAATTTCCTTTATATTGCACATCTTTTTTGAATAGCCTCTCATATTTCTAATTTCTTGGATTTTCTCGTGTATTTTAATTACAGGGGGAAGTGGCTTTAAATCTCTGGAAAATTATCGTTCCGGTTATTTTGGCACTTCTGTGAAGCTACAACCTGGTTACACTGCTGGAATTATTACTTCTTTCTATGTGAGAACAAATTTAATAAATTTTTATTTTTATTTTTTATATATATAATACTAGTCATTATTTTTTTTTCCTCCTGAATTCTGTGGTAGCTTTCAAACAATCAAGATTATCCAGGGAACCATGATGAAATTGATATTGAGTTTCTTGGAACAACGCCAAATAAGCCTTATACTTTACAAACAAATGTATACATCAGAGGAAGTGGAGATGGAAATATTATTGGGAGAGAAATGAAATTTCACCTTTGGTTTGATCCAACTAAAGCTTACCACAATTATGCTATCCTTTGGGATCCCAATGAGATCATGTTAGTCTTTTATTTAATTGTCTATTTTAGTCTTTTTTATATTTTTCTTTTTCCATATCGTTTTTATCAACTTTAAATCAACATCATGCATGCATGTATATATACATATATAATTCATATGACGTATTTGATTCGTCTCATACATGATAGGCAAAAAGAATCTTTACAACGTTTGAGTGATATGATAGCGTAAAAATTCTTCAGTGTATAGTAATTAACTCCACAAACAAAAATTATCACATGATAAGAACAACATATATAATTCCATATAAAGGTGTAATTCATGTGTGTTTAACAGTATGTTGTCGACTACAAAATGGTTGAGATTAATTTAAAGATTGACAAGCAACTACTCAACTAGACATAGAATTGAGTAAAACTCAATAGCAACAAGCCAAGAGATATTGATATGTATCTATCCATAAAATATATAGTTATGCTATTTTTGTCAAATATCAAAAAGTATTCTCATTCATTAATGTAACTCACTTTAGGCCCCATGCAAATGTTTTCTTTTACTAGTTGTTTTTTTTCTAACGTGTCTTTGCTATTGTTTTCTTTGATTTTGACTTGTAGATTTTTTGTCGACGATGTTCCAATCAGAAGATACCCTAGGAAAAATGATGCTACATTTCCACAAAGACCTATGTATGTGTATGGTTCAATTTGGGATGCTTCATCTTGGGCAACAGAGGAAGGAAGAATTAAAGCCGATTATCGGTACCAACCATTCGTCGGAAAATATAACAATTTTAAAATTGCTGGTTGTACAGCTAATGAGAACCCCTGGTGCGGACGCTCGCCCTCCAGCTCTCCGTCTAGAGCTGGTGGGCTGAGCCGCCAACAAATAGCGGCCATGCTATGGGTGCAGAGGAACTATAAGGTGTATGATTATTGTCGGGACCCTAGGAGAGACCATACTCACACTCCTGAGTGTTAG

# >NtXTH46

ATGGCTTTATTCCTTCTTTCTTTGCTTCTCCTTTTCTTGTTCAATTCAAGCAATGCTCAGGGTCCCCCTTCTCCAGGCTACTATCCTAGTTCTAAGGTTCAATCTTTAGGGTTTAGCCAGTGTTTTAGAAACCTTTGGGGTCCTCAACATCAATCTTTGGACCAAAGTGCCTTAACTATATGGCTTGATAAAACCACAGGTCTTTTAGTTCTTGTCTCTCTTTTATTTCTTTGTATGCAATATTAGTAGGGGAACTTGGAGCAACAGTAAAGTTGTCTCCGCGGTGACCTATAGGTCACGGGTTCAGCCGTCGCCGTTAATGCTTGTATTAAGGTGACTGTTTACTTTACACCTCTTGGGGTGCGGCTCTTCTCCGAACCTTACGTTAATGCGAGATGCTTTGTGCATCTGACTGTCTTTTGAACTATATATAATGATGATATAATGTGTTGTTTAACCTGTCATAATAGATTAATTATCATTTTTATCAAGCTGATCAATTAATGTCATCATAGAGATTTATATATTACATTATAAGCGATATTTGATTGTGTGTAAATATCTTTTTACACCCTCCACTAGAATTTTTCTAATCACATCTTCATTTTCCTTTATTGCACATTTAAGTTTTGCATAGCATCTCATATTTCTAATTTCTTGGATTTTCTCATGTATTTTAAATACAGGGGGAAGTGGCTTTAAATCTCTAAAAAATTATCGTTCCGGTTATTTTGGCACTTCTGTGAAGCTACAGCCTGGTTACACTGCTGGAATTATTACTTCTTTCTATGTGAGAACAAATTTAATAAAAAAAATTATTTTTACTGATATTTTGATTTTTTATATAATAATCATTATATTTCTTTTTTCATGAATTCTGTGGTAGCTTTCAAACAATCAAGATTATCCAGGGAACCATGATGAAATTGATATTGAGTTTCTTGGAACAACGCCAAATAAGCCTTATACTTTACAAACAAATGTATACATCAGAGGAAGTGGAGATGGAAATATTATTGGGAGAGAAATGAAATTTCACCTTTGGTTTGACCCAACTCAAGCTTACCACAATTATGCTATCCTTTGGAATCCCAATGAGATCATGTTAGTCTTTTATTTAATTGTCTATTTTTAGTCTTTTTAAGATTTTTTCTTTTTCCATATCGTTTTATCAACTTTATATCAACATCATGCATGTAAGTATACATAATTCACATGAAGTATTTGACTCATCGCACACAGGATATATAGGCAAAGAGAGTCGTTTATACCGTATAAGTGATATGATAGCATAAAAATTCTTTAGTGTACAATAATTAACTCTACAACTTTTAAATCAAGAGATTAGTTAAAGATTGACAAAGCAACTAGACAGAGAATCGAGTAAAACCTAATAGCAAGAAGTTAAAAGATATAGATATGTATCTACTCCTTTAGGTGACATAAAATATATAGTTATGCTATTTTTGTCAAATATCAAAAAGTATTCTCATTCATTAATGTAACCACTTTAGGCCCCATGCAAATGTTTTCTTTTAGTTGTTTTTTTTTTCTAACGTGTCCNTGATATGTATCTATCCATAAAATATATAGTTATGCTATTTTTGTCAAATATCAAAAAGTATTCTCATTCATTAATGTAACTCACTTTAGGCCCCATGCAAATGTTTTCTTTTACTAGTTGTTTTTTTTCTAACGTGTCCTTGCTATTGTTTTCTTTGATTTTGACTTGTAGATTTTTTGTCGACGATGTTCCAATCAGAAGATACCCTAGGAAAAATGATGCTACATTTCCACAAAGACCTATGTATGTGTATGGTTCAATTTGGGATGCTTCATCTTGGGCAACAGAGGAAGGAAGAATTAAAGCCGATTATCGGTACCAACCATTCATCGGAAAATATAACAATTTTAAAATTGCTGGTTGCACAGCTAACGAGAACCCCTGGTGCGGACGCTCGCCCTCCAGCTCTTCGTCTAGAGCTGGTGGGCTGAGCCGCCAGCAGATGGCGGCCATGCTATGGGTGCAGAGGAACTATAAGGTGTATGATTATTGTCGGGACCCCAGGAGAGACCATACTCACACTCCTGAGTGTTAG

# >NtXTH47

ATGGATTTCTTTCATCATAATAAAACCTTCCTATTATCACAGTTCTTGATTTTCTGCATGATAGTTGTCGTTTCATGTCGAGGTCCAGTCTATAAGCCTCCAGAAGTAGAGAAATTAACTGATCATTTCAGCCGATTATCGGTTAATCAAGGTTATAATGTATTCTTTGGAGGTGCTAATGTTCGTATGACAAACAATGGGTCCAGTGCTGATCTTATCTTAGATAAATCTTCAGGTACACTACAACTTCATTGTACAAAAAAATGTTTTTGCTTAAGTTAATATATAGTTGTTGAAGTATTTACAACAATTTCCTTTTTGTCCATAGGTTCTGGACTGATCTCTAAGGAGAAATACTACTATGGTTTCTTCAATGCTGCTCTAAAACTGCCTGCTCATTTTACATCCGGAGTTGTAATTGCCTTTTATGTAAGTTTTCATGCACGTCTTGTAACTAATCTCTGTATAACTAATACGTACTAAGCTCTAAATGCGTTATGTAGAGTACGATATAGAAATCAAGAATTTTCTCTTTCGTTGATCATCAGGGCTAATTAAGGCAAGAATTTTTATTTTATTTTTTCCAATTTTCAGATGTCTAATTCAGATGTGTTCCCACACAACCATGATGAAATTGACTTTGAGTTGCTTGGGCATGATAAGAGAAGAGATTGGGTTCTGCAGACTAATCTATATGGAAATGGAAGTGTTCACACAGGGAGAGAAGAGAAGTTTTACCTCTGGTTTGATCCAACACTGGATTTTCATGACTACACCATCCTCTGGAATAATCATCACATAGTGTAAGCGCATTGAGATAATCTAACTACTTCCTCGGTTTCTTTTTATATGATGATGTTTGATTAGATATGGAGTTAAAAAAAACAATATTGGCGCATACTAAATGTATCCTTAGAATTTGTCGTCTTAAACATGTCATGATATTTCTATGACTATAAAAGCCGAAACACTTAAAGAATTTCTAAATATAAAAACTATGTGATTCTTTTTGGAACAAACTAAAAAAAAAGGTAACGTACAACCATGGGCAAAGCTACCTTTAGTAAAGGGTGGTCAACTGACAACCCTTCGCAGAAAAATTACGAAATATTACATTGTGTATATAAATAAAATATTAATTTTAGATATATAAAACTTATATTGAACACCCTTTGTCGGAATTTTTTTTACTTCTTTTAAATTTGAATACCTTTGGAAACATTTCTGGCTTCGCCACGGCGTACAACATAAATAAAGGGAGTACTATTTTTTACTTTAGCCTTAATCGGCCACCACACGAGATAGGAATTAATTTCTAAAATTCTGTGTTAGGTCCATACATTCAATTCCCCTTGAGGTACAACAAGAGAATTTTCCACCTATTAACCCTAGTCAGCAACAAATCCTTATTATTCAATAGGTTTTTCATGAGGTTAATCCAACGCATGTTTTTACCTACAAATGTTGATTTTTTTTTTCTCAAAATGGAACTTCTGATGGGATAGAGCTATACGATTGATAGCAAGTTCAGAACTCATAAACTTCAATCCTGACTCCCCCTCTGTCCCTAGCTATTGGAAAAATATATCTTAGTTCCTTTATTTCTATTCTTTTTTGTGGTAAAAATTAAAGTATCACAGTTTATTGTAAATATAATGCAGATTTCTTGTGGACAATGTGCCAATAAGAGAGGTAGTTCATAACACAGCTATATCTTCTGTTTACCCATCAAAGCCAATGTCTGTTATAGCAACAATATGGGATGGATCAGAATGGGCAACTCATGGAGGAAAATACCCTGTAAACTACCAATATGCACCATTTGTAACATCAATGAAAGAAGTAGAATTAGAAGGATGTGTAAGACAACAAAATACTTCAGCAACTTCTACATGTTTTAGGAGAAGTACTTCAAGTTTGGATCCTGTTGATGGGGAAGAATTTATGAAATTATCACAACAGCAGATGACAGGGCTGGATTGGGTAAGGAGAAAGCACATGTTCTACTCATATTGTCAAGATACTAATAGATACAAAGTTCTACCACCAGAGTGCACTTCTAATTAA

# >NtXTH48

ATGGAATTCTATCATCAGCACAAAACATGCTTATTTTCAGGATTCTTGATTTTCTGCATGATAGCTGTGGCTTCATCTCTAGGTCCAATCTATACTCCTCCAGAGGCCGAGCGGTTAACTGATCGTTTCAGTAGATTATCCGTTAATCAGGGATATAATGTGTTTTTCGGAGGTGCTAATGTTCGTCTAACCAACAATGGGTCCAATGCTGATCTTATCTTAGATAAATCTTCAGGTAGACTCCCACATCATAATATGAGAAATGCTTTGCTTAAGTTTAAACTAGTGAAGTAACTAACTCCACGTCTTCTTGGTCCCTCCATAGGTTCAGGACTAGTCTCAAGAGACAAATACTACTATGGTTTCTTCAATGCTGCACTAAAGCTGCCTGCAAATTTTACATCAGGAGTGGTAGTTGCTTTTTATGTAAGTAGAATATATATGGTATTATGAATCATAATTTCTCTTTTATTTGATATTCGGGCTAATGCAAGAGCTTTTTTCCCCCATTTTCAGCTTTCTAATCAAAATATTTTCCCACACAACCATGATGAACTAGATTTTGAACTGCTTGGGTATGATAAGAGAAGGGATTGGGTTCTACAAACCAATATTTATGGAAATGGAAGTGTCAGCACAGGGAGGGAAGAGAAGTTCTACCTCTGGTTTGATCCAACACAAGATTTCCATGACTACAGTATTCTCTGGAACAATCATCACATTCTGTAAGCTTGTGAAAAGAAATTGAGTACTTTATGAGTTCCATTTACCTTGGTTCTAAATATAACAATTGTCTGTAAACTTGTGCAGATTTCTAGTGGACAATGTGCCAGTAAGAGAGGTTGTCAATAATACTACAATCTCTTCTGTTTACCCATCTAAGCCAATGTCTATTTATGCAACAATATGGGATGGATCACAATGGGCAACTCGTGGAGGGAAATACCCAGTAAATTATACTTACGCCCCGTTTGTAACATCAATAAAAGGAGTAGAGTTAGAAGGGTGTGTAAGCGAGCAAAACGCATCAGCAGCTAGTGCATGTGCTAGGAGAAGCACATCAAGTTTGGATCCTGTTGATGGAGAAGAGTTTGTCAAGCTGTCACAGCAGCAAATGACGGGGTTGGACTGGGCAAGGAGGAAGCATATGTTTTACTCGTATTGCCAAGATACTAGGAGATACAAAGTCCTACCACCAGAGTGCACTGCCACATAA

# >NtXTH49

ATGGAATTCTTTCACCAGCACAACACACTCTTATTATCAGAGTTCTTGATTTTCTGCATGATATCTGTGGCTTCATCTCTAGGTCCAATCTATACTCCTCCAGAGGTTGAGCGGTTAACTGATCGTTTCAGTAGATTATCCGTTAATCAGGGATATAATATGTTTTTTGGAGGTGTTAATGTTCGTCTAACGAACAATGGGTCCAGTGCTGATCTTATCTTAGATAAATCTTCAGGTAGACTCCCACATCATAATATGAGAAATGCTTTGCTCAAGTTTAAACTACTGAAGTAATTAACTCCTTGTCTTCTTGGTCCCTCCATAGGTTCAGGACTAGTCTCAAGAGACAAATACTACTATGGTTTCTTCAATGCTGCACTAAAGCTGCCTGCAAATTTTACATCGGGAGTGGTAGTTGCTTTTTATGTAAGTTTCTTGCATATCCCACAGTATTATGAATCATAATTTCTATTTTATTTGAAGCATAATGCAAGAGCTTTTTTCCCATTTTCAGCTTTCTAATCAAAATATTTTCCCACACGACCATGATGAACTAGATTTTGAATTGCTTGGGTATGATAAGAGAAGGGATTGGGTTCTACAAACCAATAATTATGGAAATGGAAGTGTCAGCACAGGGAGGGAAGGGAAGTTCTACCTCTGGTTTGATCCAACACAAGATTTCCATGACTACACTATTCTCTGGAACAATCATCACATTCTGTAAGCCAGTGAAAAAAAAATTGAGTACTTTATGAGTTCCATTTACCTTGGTTCTAAATATAACAATTGTCTGTGAACTTCTGCAGATTTCTGGTGGACAATGTGCCAGTAAGAGAGGTTGTCCATAATACTGCAATCTCTTCTGTTTACCCATCAAAGCCGATGTCCATTTATGTGACAATATGGGATGGATCACAATGGGCAACTCGCAGAGGGAAATACCCAGTAAATTATACTTACGCCCCGTTTGTAACATCAATAAAAGGAGTAGAGTTAGAAGGGTGTGTAAGCGAGCAAAACGGATCAGCAGCTACTGCATGTGCTAGGAGAAGCACATCAAGTTTGGATCCGGTTGATGGAGAAGAATTTGTCAAGCTGTCACAGCAGCAAATGATGGGGTTGGACTGGGCAAGGAGGAAGCATATGTTTTACTCGTATTGCCAAGATACTAGGAGATACAAAGTCCTACCACCAGAGTGCACTGCCACATAA

# >NtXTH50

ATGGATTATCGAGTTCTTTCATCTCTATCAAAATCGTTGACACCCTTCTCTCTCCTTATGTTGTTATATATTTTCCCGGCGGCTGAGACGGCAACGGCGACCACCGCAAAGGCTTTTAACCTCTCCACCATCACATTCGAAGAAGGATATTCCCCTCTTTTTAGTGATTTCAATATCGAACGATCTCCTGATGATACAAGCTTTCGTCTCCTCCTTAATCGTTTCTCTGGTAATTAACCTTTTTAATTATTATTATTGTAAGTATTTATTTTAATTTCTTAATTTTTATGTATATTTTATTTGATTTAGACTTTTCTATTTCAATATTAATGTTTGTCTTTTACCTTTTCAGGGTCTGGTGTAATTTCGACAGAATATTACAATTATGGATTTTTTAGCGCTAGTATTAAGCTGCCAGCCATATATACTGCCGGCATCGTTGTCGCATTTTATGTAAGTAATATATGCATGTTGTTGTATGTGTATAGTTTCTTTATGTAAAATATATGCATGTACATGATCTGAAATCCAAAACAAGATTATTTATTGAAGGAAAAAGCACATTTGTTATTTTGCACGGTGAAGAGTTCCATGAAAATGCGTCTACTTTGACTATTAATGCATCATTGGAATTTAAAATAAGTGATAATACATGAAATTTCTTTTCTCTTTTCAAACATCTATAGTTGAAAGATGTTGTATTAATTGATATATAGACGTCAAATGTGGACACATTTGAGAAGAATCATGACGAGTTAGACATCGAGTTTCTGGGGAATGTGAACGGGCAGCCATGGAGATTTCAGACCAACTTGTATGGAAATGGGAGCGTAAGCCGTGGGAGAGAAGAGAGGTATAGAATGTGGTTTGATCCTAGCAACGACTTCCATCACTACAGCATTCTTTGGACCCCCAAAAATATCATGTAAGCACTTTAATCATTTTTAAATTTTTTAATTGCCTTCTTCCAATTTATTTGGATAGTTAACTTTAAGATGAAATTTAAATTTTTCCTAATTGCTTCTTCTCTTTAATTTTATTCCTTTTATAATAGGAGTATAATAATTTTGTTAGTTATGGAAAGCATTAGGCAGCAATTAGTTGCTGTAAAAATTGGCATTATATTGTGCCTACCTTTTGGCCAAACGCGGAGAGAGAAATTAATAAGAAAGAAAGGTTGCTAGCTATTCGTTTTCGAAAAAGTTAAGCACCTAAATAATTTTGGTTACCTATTGCGTTTGTTGGATGAGACCATGCTTAGCTTTCTTTTAGGTACTTTATGGTTGGTTTTTTTATGAACTTTTCCGAATTTCTATTTTAATGATTAATAGTTGCTATATATACAAACATATGAAAACATGTGATGATTTATAACTTCCACAAATTACTGCCATATATTCACAAGTGTTCTTGCATAGAATAGAGACTCTTTCTTGGAGGAACTGAAAGTTAATTTTGTACATATTTTTTCTCTGGAGTTTATTTTTCATACTTTTTACTACTTTTTTTTTTTTTTTTTTTTTTTTTTTTTTTTTTTTTTTTTTGCATTTGGAATGGGTGACCTTAGTGTCGAAAGAGGGGGTCATAACATGCCAAAGAAGTTATGGTCCAGTTCACGGGAGGAAATAATTAAACTGTTGATTAGCTCATTAATGAGAGTGACTAATCTTCTAAAAGGGTAAAAATTGTAATTGCACGGTTTACGGCAATGCAAATATTACTTTTTCCTTTTCTAAGAAACGTCTTTAAAGCTGGAAAACTGGCGTGATGGGCTGTTTTCCAAGTGACGTTTGCACTAGCTAATTGTATACGTTAAGACTTAAAAGAGTTTGTCTATACTTTGAACTTATCACGCTTAATAAATGTCGTTGTTAGCACAAAATTCTTATAAGTGCCCTCATAACAAGTGTTTAGGTTATGTTGCCTCAAAGTGTACTTACCGTCAAGTTTATATGATATAAACTGCCCAAGATATAGCCAACTGAAAGCTTTATAAAGATAATCTTGTTTCTACCCATAAAATATATACTACTAAACTAAGAGTACAATAGGAAATATCAATGTGTTTCTTGGCTAGCTATAAGGAAGGTTCAGTGTGGGATGAGTCTGGCCAAATCTGAACTGTTTTGTCATTCTTTTATGGACTAATTTAAGGTTTAAAAGCTGTTTTAAAAGGTTGAGAATGAGTATTGCCCTAATTCTTCTTTAATGTTCTTGAAAACCTGGATATTCACAATGTTACTAAAGGCACAGATTTGTATATCTCTGCTCCATTTGGCACTCCACAATTGAGATTATTTAGCTTCTACCTCCGAATACTAGCTAAACATTCTATTATCGTGGTAGATATTTATCAAAATCGCTCTTCTAAAAGATTTTCGTGATGTTTCCTACTTGGAATATTGAGTAAAATCCATACTCAACTACTTACTGATGTTTTCAATAACCTAGATTTGGAAAACCATTAAAATGAGCAATTTAGCCAAGTACATTGACAACTAGCATACTTTGTTCTATTCGCTTTTCTTGATTCTCTAATCTCTTAAATTGCCAAAATAAATTATTTAAGAAATCAAGAAAAAAGGAGAATGTCCCCTTTACAGTTGCACATTACTAGAAAGTTGTCGAACAAAAGATTCCTTAGCTTGTTGAAGATTGTAATGTAGTGGCAGATTGTAATGTAACAACTTCCAATACTTTGTAACTTACAAGTTTTTGTTGCATAAGATGTCGATAAAAATCATGTATATCAAAAAGAATTAAGTTACGACAGTGTAAAAGTTATTACAAAGCTAAAAATTTGTTCATTCATTAGTGTTTCTTTTTAACTAACCTCCAAAACCTTGGTTTTGTATAGATTCTACGTTGATGAGACACCAATAAGAGAAGTAAATCGTAATCCAGCAATGGGAGGGGACTTTCCATCAAAACCAATGTCCTTATATGCCACAATTTGGGATGCATCTTCTTGGGCTACAAATGGCGGCAAGGCTAAAGTTGACTACAAACATGAACCTTTTGCAACTGAGTTCAAAGACTTGGTTCTTGAAGGCTGTATAGTAGATCCCATTGAGCAAATTTCATCTACAAATTGCACTGATAGAATTGCCAGACTGCTTTCTCAAAACTACTCTATCATGACACCCGAAAGGCGAAAATCAATGAAATGGTTTAGAGAAAGATACATGTATTATTCTTATTGTTATGATAATATTAGGTACCCTGTGCCACCTCCAGAATGTGTTATTGTTCAATCAGAAAGAGACTTATTTAAGGATAGTGGAAGGCTTAGGCAGAAGATGAAGTTTGGTGGCAGCCACAGCCACCGGAAACACCGCCCTGGACGGAGCTCTAGGCGGCGGAATAGGGCTGCTGGTGGTGGTTCATCAAAGTCTGGCCAAGCTGCTGCAATGTAA

# >NtXTH51

ATGGATTTCATCAGAAAGAAGATATGTCTGTCTGTCTTCTTGTTTTTCCATGTCTGGTTTAGTACAGCCCTTAATGTCTCCACCATACCTTTTAGCGATGGCTTCAGCCATCTCTTTGGCGAAGGAAACATTCTTCATGCTACTGATGATAAGAGCCTTCAACTTCACCTCAACCAACGCACAGGTAAAAATATGTATATTCAACCTGTTTAGTACGACCGAAGTCATAAAAAATACTTTTTGGAAATTAAGTTAACGTAACTTCATGAAAATCCTTTATTTTCCTCTACAGATGTACATATAACTATCATATGATTTTCTTTTTCTTTGTTTTATGTCTTCCAGCTGTTCTGTTATCGTAGTTTTTACCATACCATATATATATTTCTAAATTTATTTCCCATTGTAATTATTTAAAATTCTTACAGGTTCAGGGTTTAAATCTTCTGACCTCTACAACCATGGTTTCTTCAGTGCTAAGATAAAATTGCCATCAGATTATACTGCAGGAATCGTTGTTGCCTTCTATGTATGCATCTCTACTATTCTTTTTTCCAAATATTATGATTTTCATATCGTAGTTTTAATTAATACGATCACGTATATATTTATTTATTTGTTTGTTTTGGGCAGACGACGAATGGTGATTTATTTACAAAGACACATGATGAACTGGATTTTGAGTTTCTGGGAAATATAAGAGGAAAAGCTTGGAGATTTCAGACAAATATGTATGGAAATGGAAGCACAAGTAGAGGAAGAGAAGAACGATATTATCTTTGGTTCGACCCTTCTAAAGAATTTCATCGTTACAGTATCCTGTGGACCAACAAAAACATCATGTGAGTTTTTTTAACTTCTTTCTTCAATTTTAAGAAAAGTACAAACATCATATCATCCTTTTACCAACGCGGGTTCGTTGAATTTGACAAATTTGCGGCTTCTTTTGATATTTGACCATTTCAAAACTACAGTTCCTTTTCTAGAAAGAAATATGGTACACATAACTTCCAAGGATCACATGTAAAATATGTTAAGTATATTATAAATAGGAATTTTTTACACCATTAGATTACTTGAAAATTAATTACTGTAGAAAATTACTCCCTTAAAAAAGAGACAAATAACCTCTTAGAACATATTAAATTGCACTGATAAAATTAAAAAAGGAAGCATTTGTATTCTTATAATTCAACTTGGATAACTTTCAAAATGATCTGGGACAAGAATCTGCCGTAAATTAAATGTGAAATTTGCTATGTAAATATATTGTAGATATAAGTTAAAATTCCTATAAATATTAGGAAATTTATGATCGGAAATCTGAAATCTACAACAAAAAAGAAGACCATTTCAGGTAATTAGGTATCAGAAAAGTACAAACAGAAATATAAAATTAGGAAATTGAAAGTTCAGGGAGATATTACCAATTAATAATATGCTTTTTGAATATTGAGAACGTAAGGTGCGACTTTTCTTTTTTCTTTGACGTGATGAATTTGTACTTGACTGGTTCAATGAATCTATTCATTTAACTTGTCAGATAGTTTATCTGATTTATTAAATGGACGACGTATAGCTGAACTGAAAAGTTTGATGTTCCGTGATCAAATTGTAGATAACCTAATTAATAATCTCCAATATTAAAGTAACTTATTGACAAATGACAATTACGTCTATCAATTTCAAGGTCTTAATTGATGATTTGACAATTTAATTTGCTTATTTTTTTCTTTTTGGATGTTAACATGAGTTTGGATTACCAGTTTATCACATACAAAACATATGGCTATAATATTTTTTAGAAGATAATATGAAAAAGGAAAACACACAGAAACAAACAATAGGAGACGTTGAGAATTTTCTTTCTAAAACCTCTTCAAGATACGCCAACCATTCAACCAATAGTCTGGTTGAATTAGTTTAACCACTTTGTATTACATTGAATTAACTAACATAAATTATTTCTCACTAGAAAAAAGAGAGATAAATATAGTTTAACCACTTTGTATTACATTGAATTGACAACATATATTATTTCTAATTAGAAAAAAAGTTCATATAACCTAATCCACATTAAAGTAATTAAATGATAGATATCTTTTAATATTGAACCAACTCAATTATTGACAATTTTTTAATTAAATTCTTTTTTAATTTTATTTGAGAACAGATTTTATATAGATGATGTTCCAATTAGAGAAATCGTACGTAATGATGCAATGGGAGGAGACTATCCATCAAAGCCAATGGGACTATATGCAACAATATGGGATGCTTCAGATTGGGCTACTTCAGGAGGCAAATATAAAACAAATTACAAATATGCACCATTTATAGCTGAATTCACTGATTTAGTACTAAATGGATGTGCAATGGATCCATTGGAACAAGTAGTAAACAACCCTAGTTGTGATGAGAAAGATGATGAACTTCAAAAGGCAGATTTTTCAAGAATTACACCAAGACAAAGAATGGCTATGAAAAGATTTAGGTCAAAATATATGTATTATTCTTATTGTTACGATTCTTTGAGATACTCAGTGCCACCACCAGAATGCGAGATAGATCCAATTGAACAACAACATTTCAAAGAGACGGGGAGGTTGAAGTTTAACAAGCACCACCATCGCCATCCAAAGAGAACAAAAAGTCAAGTTCTTGATGCTAGGAATTATGGAAATCAAGATGAAGAGTGA

# >NtXTH52

ATGGATTTCATCAGAAAGAAGATATGTCTGTCTGTCTTCTTGTTTTTCCATGTCTGCTTTATTACAGCTGATGCTGCCTTAAATGTCTCTACCATACCTTTTAGCGATGGCTTCAGCCATCTCTTTGGCGAAGGAAACATTCTTCATGCTACTGATGATAAGAGCCTTCAACTTCATCTCAACCAACGCACTGGTAAAAATATACGTATTTTTTTCGAACTGAGTGTGTTTGACACGACATAATTTATTTTTTTGCTGAAGAACTTCTTGAAAATATTTATGTTCTCCATAAAGAATACGTAAATATATATATGTATAACCATCCTATTCTTCTTCTTTGTTCTTCTTCCAACGGTTTTTTTAACATACCAATATGTGTCTAAATTTATTTCCCTTTTTTATTATTAAAATCTTTTACAGGTTCAGGATTCAAGTCGTCTGACCTCTACACCCATGGTTTCTTCAGTGCTAAGATAAAATTGCCATCAGATTATACTGCAGGGATCGTTGTTGCCTTCTATGTATGTATCTCTACTGTTTTTTTTTTTTTCCTTCATTCGTATATAGTAGTTTTAATACGATTGCGTGTATATATATATATTTATTTATTTATTTGCTTTATTGCTAGACGACGAATGGTGATTTATTTACAAAAACACATGATGAACTGGATTTTGAGTTTCTGGGAAATATAAGAGGAAAAGCATGGAGATTTCAGACAAATATGTATGGAAATGGAAGCACAAGTAGAGGAAGAGAAGAACGATATTATCTTTGGTTTGACCCTTCTAAAGAATTTCATCGTTACAGTATCCTGTGGACCATCAAAAACATCATGTGAGTTTTCTTTCTTCAATTCTAAGAAAAGTACAAACATCATATCTTGCTTTTACCAACGCGGTTCGTTGAATTTTAGTACAAATTAATTTGTGGCCGCTTTTGATATTTGACCATTTCACAATTACAGTTCCTTTTCTAGAAAGAAATATGCTACACAACTTACATATGCTAACACATGTATAATATGTTAAGTATATTATAAATAGAGTTTAACTTTTATATATTTACTGATATTTTAAGATTTTTTGCACCATTTAGCCGTCCATATTCATTGTTTATTTTTTCTAGTCATATACGTAGTTTATATATTGATTATATATAATTACACACGTATAATACATAAATTAAGCATATATATTATCTTCACCGGCTATTTTTAATTTAAGCGATTGGATTATGTGGGTTAATTCTTCGAAATAATCACCTAAAAATAATGACAAATAACCTCTTAAAACATATTAAATTACACTGATAAAATTGAAAAAGAAACTATTCGTACTCTCATAATTCAACTTGGATAAGTTTCAAAATGATTTGGGGCATAGAATCTCATAAATGTGAAATTTGTTGCTATGTGCAAATACCGTAGATATAAGTTAAAGTCCTATAAATATTAGGAATTTATGATCGAAAATCTGGAAACTAAAACAAAATAATAGATCATTTCAGGTAATTATTAGGTATCAGAAAAATAGAAATAGAAATATAAAATTAGGAAAATTGAAAGTTCAGGGAGATATTACCAATTAATAATATGCTTTTTGAATATTGACAACGTAAGGTGCGACTTTCCTTTTTTCTTTTACGTGATGAATTTGCACTTGACTGGTTCAATGAATCTATTCATTTAACTTGTCAAATAGTTTATCTGATTTATTAAATGGACGACGTATAGCTGAACTGAAAAGTTTGATGTTCCGTGATCAAATTGTAGATAACCTAATTAATAATCTCCAATATTAATTAAAGTAACTTATTAACAAATGACAATTACGTCTATCAATTTCAAGTTCTTAAATGATGATTTGACAATTTAATTTGCTTATTTTTTTCCCTCTTTGGATGTGAACATGAGTTTGGATTATCAGTTTATCATATACAAAACATATGGCTATAATATTTTTTAGAAGATAATATCAAAAAGGAAAACACGCAGAAACAAACAATAGGAGACGTTCAGAATTTTCTTTCTAAAACCTCTTCAAGATACTCCAACCAATAATCTGCTAGAATTAGTTTAACCACTTTGTATTACATTGAATTAACTAACATAAATTATTTCTCACTAGAAAAAAGAGAGATAAATATAGTTTAACCACTTTGTATTACATTGAATTGACTAACATATATTATTTCTAATTAGAAAAGTTACATATAACCTAATCCACATTAAAGTAATTAAATGGATAGATACTAAGAGATATCTTTTGATATTGAGCCAACTCAATTATTGATAATTTTTAATTATTTTTTTTTTCTTTTAAATTTTATTTGAGAACAGATTTTATATAGATGATGTTCCAATTAGAGAAATTGTACGTAATGATGCAATGGGAGGAGACTATCCATCAAAACCAATGGGATTATATGCAACAATATGGGATGCTTCAGATTGGGCTACTTCAGGAGGCAAATATAAAACAAATTACAAGTATGCACCATTTATAGCTGAATTCACTGATTTAGTATTAAATGGATGTGCAATGGATCCATTGGAACAAGTAGTAAACAACCCTAGTTGTGACGAGAAAGATGATGAACTTCAAAAGGCAGATTTTTCAAGGATTACACCAAGACAAAGAATGGCTATGAAAAGATTTAGGTCAAAATATATGTATTATTCTTATTGTTACGATTCTTTGAGATATTCAGTGCCACCACCAGAATGCGAGATAGATCACGTTGAACAACAACATTTCAAAGAGACGGGGAGGTTGAAATTTAACAAACACGGCCACCATCGTCATGCAAAGAGAACAAGAAGTCAAGTTCTTGATGCTAGGAACCATGGAAATCAGGATGAAGAGTGA

# >NtXTH53

ATGGTGAACTATCATCTTGTTACTTTCATATTTTTCTCTGTTGTTGAATTGGTTTATGGGTCTTCAAGAAATTTGCCAATTTTAGCGTTTGATGAAGGGTACTCCCATCTCTTTGGTGATGATAACGTTATGATCCTTAAAGATGGAAAATCTGCTCATATTTCTCTAGATGAAAGAACAGGTTCCTTTTTTCCCTTATTTGTAGTACTATTTTTTATGGATTTTATTATTATTAAAGAGAATTTATTTTTTGGATGAGTTACAGGGGCTGGATTTGTGTCTCAAGACCTATATCTTCATGGATTCTTCAGTGCTTCTATTAAGCTGCCTGCTGATTACACTGCTGGTGTGGTTGTTGCATTTTATGTAAGTTATTATTAATTCACTGATGCTCCTTTTATTCTTTATTTTAATAAACAAACATTAAATCCTGATGTTTTTTCAACTGGATTTTTAATTTATTTATTTATGTGTATTTTTCATTTTCTAAATTAGTACTAGCAGTTTTTGCTTCTTGTGGGTGGAAGTTCTTATTGTTTTCGCACCCATATGGACTGTTGATGATTTTTTTATATGTTAATTAATTCTGTAAATTTCTCCCTTTGTCCTCACTCAGTTATTTTTAAAATCTTTTTTACCTTTAAAGAATGGGGTTTTCACCACCCAATGGAAATTGGTAATTCTGGAATTGTGTTAAATTATTTTCTGGGATTTCTCCAAATAAAAATTGGATTTTTTTAACCCTTGAAACCCATCCTTTTAATATTTGTTTTGCTAATTGGGTTGATGACACAGATGTCTAATGTGGACATGTTTGAGAAGAACCATGATGAAATTGACTTTGAGTTCTTGGGAAATATTAGAGGTAAAGACTGGAGAATTCAGACCAATATTTATGGGAATGGTAGCACTAGTGTTGGCAGAGAAGAAAGATATGGACTCTGGTTTGACCCTTCTGAAGATTTCCATCACTACAGTATCCTTTGGACTGAGAATTTCATCATGTAAGCAACATCTCTCTGCCCTTTCAATTTCTTTTTATGTTTCTAATTTTTGTCTGAAATAGCTTTTTGCTTCGTATACAATGAGTTACTTTCAAGTCCAGCTAGCTATACTGAACAGTCTTTTGTCCTCTGAGCTGTTTTTACTGCTTTTGGTGTTTATGTTTTGATCTCTGTTTGATATGATGGATACATGGTTTTCCTTTATCAGAAAGGTCCATTTATATGTGCTTGCTTTCTCTTTATATTTGGTATCAATTTTTGTTTTTTTCTATTTTGATGATACTAATGTTGTGCAATTACCTTATAGTTTGCTCTTATCTTGGTTCTAAACATAGATATATATGATTACAATGACCTACTTGTACTCCTCAAAACTAACCATAGGAAACAAAAGTAATTTCATGTGAAAGTGCTGTAAGACAAGAATTCGGCGTGCGCAGGTGTTAATAATTTTTGATGGAGGGTTCTTGCTAAATCTGTTCTGTTGATGTTTGGACTTGCGATACGCATGCTTCTGTTTCTGAACTAATTATCCAATGAATGCAACTATCACAGCAGACCTTTTTGATTTATTTATGTTTCATATTTGAGCTTAAGAACTACTATTTGAGAAGAACATTAGCATCTGCACTAGTGAGTGACTGGAAAGTTTATACCAATTGATATATTTATGTGAATGTACAAGAATGATAGGGACATTCTCAACATACCCTTTTGTTGGCATGTCTGTGGGTTTCATGGGAGAGTAGGGCAGGCTTGCTTGTTTTTTATATTACTGTAAACAATTAGGATTTGTATACTGTCATGTGCTTGTGGTGTTTGGTAAACCAGGCCCTGATTCTGTTATGTTTGTCTTTGTGTGTTTGTGCATCTTGTTGCAGCTTTTATGTAGATAATGTCCCCATAAGAGAGATCAAGAGGACAGAAGCTATGGGTGGGGACTTCCCATCTAAGCCAATGTCTTTGTATGCTACAATATGGGATGGTTCTGGTTGGGCTACCAATGGTGGAAAATACAAAGTCAATTACAAATACGCCCCGTATATTGCCAAGTTCTCTGATTTCGTCCTCCACGGATGCGCAGTTGATCCGATTGAACTATCATCCAAATGTGACACTGCACCAAAAACTGCATCAATCCCTACCGGTATTACCCCTGATCAAAGAAGAAAAATGGAGAAGTTTAGAAAGAAGCAAATGCAGTATTCGTACTGCTATGACAAGACTCGGTACAAGGTCCCTCCACCGGAATGTGTGATCGATCCTAAGGAAGCTGAACGACTCCGAGCCTTTGACCCGGTTACATTTGGCGGATCCCGCCACCATCACGGGAAACAACACCGCCGGAGCAGATCAAGAGCTGAGGGTGATATATCCTTTCTGTAA

# >NtXTH54

ATGGTGAACTATCATCTTGTTATTTTCATATTTTTCTCTGTTGTTGAATTGGTTTATGGGTCTTCAAGAAATTTGCCAATTTTAGCGTTTGATGAAGGCTACTCCCATCTCTTTGGTGATAATAACCTTATGATCCTTAAAGATGGAAAATCTGCTCATATTTCTCTAGATGAAAGAACAGGTTCCTTTTTTCTTTTCTTTATTTGTAGTACTATTTTTATGGCTTTTATTATTTTTATTAATGTATGTTTATTAGCGAGAATTTATTTTTTGGATGAGTTACAGGGGCTGGATTTGTGTCTCAAGACCTATATCTTCATGGATTCTTCAGTGCTTCTATTAAGCTTCCTGCTGATTACACTGCTGGTGTGGTTGTTGCATTTTATGTAAGTTATTGTTAATTCACTGATGCTCCTTTTATTCTTTATTTTAATAAACATTAAAACTTGATGTTTTTTTCATCTGGACTTTTAATTTTCTAAGTTAGTACTAGCAGTTTTTGCTTCTCATGGATGAAAGTTCTGATTTTTTTTCACACCCATATGGATTATTGATGATTTTTATATGTTTATTAATTTTTTAAATTTCTCCCTTTGTCCTCACTCCTCAATTATTTTGATTTTTTTTTTACCTTTAAAGAATGGTTTTTTCACCACCCAATGGAAATTGGTAATTCTGGAATTGTGTCAGATTGAACAAAAAGTTAAAATTCTGGGACATAAAAATTGGAATTTTTTTTACCCCTGTATTGATAAATCTTAATCAATTGGGTTGATGACACAGATGTCTAATGTGGACATGTTTGAGAAGAACCATGATGAAATTGACTTTGAGTTCTTGGGAAATATTAGAGGCAAAGACTGGAGAATTCAGACCAATATTTATGGGAATGGTAGTACTAGTTTTGGCAGAGAAGAAAGATATGGACTCTGGTTTGACCCTTCTGAAGATTTCCATCACTACAGTATCCTTTGGACTGAGAATTTTATCATGTAAGCAACATCTCTCTGCCCTTTCAATTTCCTTTTATGTTTCTAATTTTGGTCTGCATTTTTGCTTCTTATACCAATGGTTACTTTCAATTCCAGCTAGCTATACTGAACAATCTTTTGTCCTCTGAGCTGTTTTTACTGCTTTTGTTTTTTATGTTGTGATCTCTGTTTGATATGATGGATACATGGTTTCCCTTATCAGAAAGGTCCATTTATATGTGCAATCTCCTTATATTTAGTCAGTTTTTTTTTTTTTTTATACTAAAGCTTGTGCAATTACCCTATGGTTTGCTCTTACATAATTCTGAACATAGATATAAATGATTACAGTGACATACTTGTACTCCTCAAAACTAACCATTGGAAACCAACATCATCTCCCTCCCTTACATAAAAGTAAAATAATGAAATCCCTGACATGAAAAAAGTTTCATGGGACTGCTGTAAGACGAGAATTTGGCGTGCACAGGTGCTAATAATTTTAGATGGAGAATTCTTGCTAAATCTGTTCTGTTTGGACTTGCGATACGCATGCTTCTGTTTTTGAACTAATTATCCAATGAATTCCACTATCACAACAGACCGTTTTGATTTATTTATGTTTCATATTTGAGCTTAAGAACTACTATTTGAAAAGAACATTAGCATCTGCACTAGTTAGTGACTGGAAAGTTAATACTGATTGATATGTTTTATGTGAATGTACAAGAATGATTGGACTTTCTTAACATACCATTATGTTGGCATGGCTGTTGGTTTCATGGGAGAGTAGGGGAGGCTTGCTTGCTTTTTATATTACTGTTAAATTAAACCTGTATTGCAGATTTATTGTCCTGTTTCAGTCTTATGATGTCAAAACAATTAGAATTTGTATACTGTCATGTGCTTGTGGTGTTTGGTAAACAGGCCCTGATTCTGTTATGTTTGTTTTTGTGTGTTTGTGCATCTTGTTGCAGCTTTTATGTAGATAATGTCCCCATTAGAGAGATCAAGAGGACAGAAGCTATGGGTGGGGACTTCCCATCTAAGCCAATGTCTTTGTATGCTACAATATGGGATGGTTCTGGTTGGGCTACCAATGGTGGAAAATACAAAGTCAATTACAAATATGCCCCGTATATTGCCAAGTTCTCTGATTTCGTCCTCCACGGATGCGCGGTTGATCCGATTGAATTATCATCCAAATGTGACACTGCACCAAAAACTTCATCAATCCCTACAGGTATTACCCCTGATCAAAGAAGAAAAATGGAGAACTTCAGAAAGAAGCAAATGCAGTATTCTTACTGCTATGACAAGACTCGGTACAAGGTCCCTCCAACGGAATGTGTGATCGATCCTAAGGAAGCTGAACGACTCCGAGTCTTTGACCCCGTTACATTTGGCGGATCCCGCCACCATCATGGGAAACGACATAGCCGGAGCAGATCAAGGGCTGAGGGTGATGTATCCTTTCTGTAA

# >NtXTH55

ATGGTGAATTTTCGTCTGGAAATTTTCATATTATGCTCTTTTCTTGTATTAGTTTGTGGGTCTTCAAAACAGCTCCAAACTTTACCGTTTGACGAAGGGTACTCACAACTCTTTGGCCATGATAATCTTATGGTTCTTGAAGATGGAAAGTCAGTTCATCTTTCTCTAGATGAAAGAACAGGTTCATTTCTTCTTTTGGCTTTATTTTGTAGCACTAGTTTAGTACTAATGTAATATTAAATGTGTAATATTATTTGTGTGACTATAAACCTGTTATTATTTTTGAAACAGACTAATACTGAAATAGTGTCGGAGAGGGAGTAGTTTTTGCTTAACATTTTTTTTGTTCACGGAAATTAAACTAATTTTATTGTTTGGGAATTAAATGGATGAATTACAGGAGCAGGATTTGTGTCTCAAGATCTTTACCTTCATGGCTACTTCAGTGCTTCTATTAAGTTACCAGCAGATTACACTGCTGGAGTGGTTGTTGCATTTTATGTAAGTAATAACTGATCGATTCTTTGTTTATTTAGTTCAAAATTAAGAAAAGGAAAATTCTTGATGGTTTTTGCATTAAGATTTTATATTTTTTTATGGTGTGTTGGGAAGTTTGAGCTTGGCAAAGTTTGGCATCCTCTAGTTCTTGATAAAGAATGAGAATAATGCAAGGTTGACGACGGTCAAGTGAAAATAGTCAGAATTATACTCTAATATAAATATAAAAGATTATTTATATAGCGGATATTATTTAGTTTAGGATTTCATAGGTGAAAGGTTTGGGTGCAATGAGGTCTTCTTTTATTTGTGCAAGTTTTGTTAGACAGAGTTACTTGGCAACAATATATTAATAAAAATTTTGAGACGTCCTAAGTTGGCCTGGACACCGCAATCATTAAAAATAAATAAATAATATGGCAAGCATGCTCATATGGGTTCTTGATGATTCTGGTTTTTTAATTACATTTTTCCTGAAGTTGGGTAAACATACCATCATATTACCAAAAAGGAAAAAGAGGAAGACAAGTATAGTGGAAGGTCTCCTAGATTAATCCCCTTTATTTCCTTCTTTTTACCCTTCAGACTGATTTTCACCACCTTTCAGAAACTGTAAATTTCAGAATCTGATTATTTGATGAATCAAAATTCTCATTTTGGTTTTTAAAAGACTTCAGTATAGTGTTATTGCTTTGATAAGTAATTGGATTGCAGTGTTACATAAAGTACTATCCTGCAACTTTCATTTTTTAAAATTTTTTTTTACTATTTAAATGATGACAGATGTCTAACGGCGACATGTTTGAGAAGAACCATGATGAAATTGACTTTGAGTTCTTGGGAAATATAAGAGCAAAAAAATGGAGGATTCAAACTAATATATATGGGAATGGTAGCACAAATGTTGGCAGAGAAGAAAGATATGGACTCTGGTTTGATCCCTCTGAAGATTTTCATCAATATAGCATCTTGTGGACTGAGAGCCAGATCATGTAAGTACCTAATTCCACCAGAAACAGCTCTCCCCTTTTAATTAATTTTTTTCAAAAAATATAAATGTATACCACTAAAACAAGTGTCTCGGATAGCATAAGCCACTGATACTTGCATCATGGTAGGCTTCCTACATCGCACCCCTTGAAGGTGCGGCCCTTCTCCGGACCCTGCGTGAATGCGGAATATTTCGTGCACCGGACTACCCTTTATTTTTTGTGTTCTTTTTGTCTCTATTGGGATTAAAACCGTTATAGCCTTTCTAGTTTAGTTGACAACTAGGCCTCACCTAGGATGTTTTTCGTGATTTTGATTTAAATACTTCCTATTAGATGTGCCATGTTGCTAATGCAGTGTTATCCATTTTCTTTTTGTGGTGGTACTAAAGCCAGTGGCTTTAATTCAACTCTAACCTTATGCATCTTATTACAGTGACTAGTATTCTCCTCTTATATAACCCTTCTAGTCAAACGTAACAACCACACACCACCTTAATCTATACAACACTTTTGATTGATTCTGTTTTTGTGTTTTTTCAGATTAAGGACCAATTAGAGGAAAAATCAGAACTTGAATTAGTGTGTAGCTTGAAAATTCATCCTTATCCATATCACGGGCAGGCTTGCTTTTTGTCACTGTAGTTCTTACCTTCCTTTCTGCAAGACTTTTTAAATGATTTCTACTTCTGCCTTTGGTCCACTGTTTCAGAATGCAGGGAATCTCTCTTTTCTGCCCTTTTTGNNNNNNNNNNNNNNNNNNNNNNNNNNNNNNNNNNNNNNNNNNNNNNNNNNNNNNNNNNNNNNNNNNNNNNNNNNNNNNNNNNNNNNNNNNNNNNNNNNNNTACATAACTCAGTAAAACTTTGAGCAACATTTCCTGGATTTGTTCTTGGCTTGAGTACTTAACTATTAGAAGTCCCTCTTGCTGTTCACTCTTATGGTATGAAACTACCAGTTTTTTAACACTGACATGTATTAGTGGAGTTTGGTAATCCAAACACTGATTATATTTTGTTTGATTTGTGTGTTTTGTGCATTTTGTTGCAGCTTTTATGTAGATAATATCCCCATAAGAGAGATCAAGAGGACAAAAGCAATGGGTGGGGACTTCCCTTCTAAGCCAATGTCTTTGTATGCTACAATATGGGATGGTTCTAGTTGGGCTACCAATGGGGGCAAATACAAAGTCAATTACAAATATGCCCCTTATGTCGCCAAGTTTTCCGACTTTATCCTTCATGGATGTGCAGTTGATCCAATTGAATTGTCACCAAAATGTGACACAACCCCTAATTCTGCATCCATTCCAACTAGTATATCCCCTGATCAAAGAAGAAAAATGGAGAGCTTCCGAAAGAAGTACTTGCAATATTCATACTGCTATGACCGGACTCGATACAATGTACCTCTATCTGAATGTGTAATTGATCCTAAGGAAGCTGACCGTCTCCGAGGCTTTGACCCCGTGACCTTTGGTGGCGTCCAGCGCCATCACAGCAAACGACACCACCAGAGGCAATCGAGGAGGGAAGACACGTCTTCTGAATAG

# >NtXTH56

ATGGTGAATTTTCGTCTGGGAATTTTCATACTATGTTCTTTTCTTGTATTAGTTTCAGGGTCTTCAAAAAAGCTCCAAACGTTACCGTTTGATGAAGGGTACTCGCAACTCTTTGGTCATGATAATCTTATGGTTCTTGAAGATGGAAAATCAGTTCATATTTCTCTTGATGAAAGAACAGGTTCATTTCTTCTTTTTTGGCTTTATTTTTGTTTCAATTTGTGTGACGCAGTTAAATGAATATATTTGGTTGTCTTAAATAATTTATAATATTTATGTAGCTATAAAACTATTGAAATTTGTAGTATCAAATATGTCATAATATTTGTGTGACAATAAAACTTGTCATTATAATTAAAGGCGGACCTAGGAATATTGGAAAGTGGAGGGTGCACTTTTGGATTCAACCAAAATTTGTTTTGTATATAGGGTGTCCACTATTAATATATCATTATTTTCTAAAGTTATATACATGAAATTTTCGCCAACCAAAATTTGTTTTGTATATAGGGTGTCTACTATTAATATATATCACTATTTTCTAAAGATATATACATGAAATTTTCGCCAAACTTTACGGGTGCCGGTGACCACTCTTGATATAACGTAAGTCAGCTTCTCATTAAGGCTATATAAGAAAATCAAGTTAGATTACTTCCAAATGTGTCATTATTTTTACGGAAGAACCTATAAGAAAATTCTGTAATATTTTATAAACTAGAACGAAAGGTGGCCTAGTTTAGTAGTAGCTTTTATATGGCTGTTTGTTTTTTTCAAGGAAATTTATTTATTTGGTTTTTGTGGAATTAAATGGATGAAATTACAGGAGCAGGATTTGTGTCTCAAGACCTCTACCTTCATGGCTACTTCAGTGCTTCTATTAAGTTACCTGCAGATTACACTGCTGGAGTGGTTGTTGCATTTTATGTAAGTAAAAAAACCAATACTATCTGATTGATTCTTTATTTATTTAACAAAAATAAGAGGAAGATTCTTGATGAATTTTGCATTTGGATTTTATGTTTTCAATTTTATTTTAAGCTTGGAAAAGTTTGGCATGTTCCAATTCTTGACAAAGGATGAGTGTCATGCTAGGTTGACGGTCAAGTGAAAATAGTCAGAATTATACTCTATTATAAATATAAAAGATTTATATAGTGGATATTATTTAGTTTAGGATTTCATAGGTGAAAGTTGTGGGTGCAATGAGGTGTCTAGGTAATTTCTTTCATTTCCGCAAGTTTTGTTAACAAAGTTACTCGATATTTATGTTGGTGGAAGATAATAATATATTCGATAAAGTATTTGAGATGCCCCTAAAAAATATACTCCTATATAATATGACAAGCATGCTCATTTGGGTTCTTGATGATTCTGGTTTTCTGATTATATTTTACCTGAAATTGGGTAAACATACCATCATATTACCAAAAAGGAAAAAGAGGAAGACAAGTATATTGAATTGTCTCCTAGATTAATCCCCTCTATTTCCTTCTTTTTATTCTTTTTTTTTTAATAATTGTTTGTGGGGCTGGGAACTGCCCACCTTCCCCTCTCTTCTTTCTTTTGTATCTTTTTACCTTTCAGACTGATTTTTACCACTTTTCAGAAACTGTAAATTTCAGAATCTGATAATATGATGAATCAAAATTCTCATTTTGGTTTTTAAAAGAATCATAGTGTTATTTCTTTGATAAGTAATTGGATTGTAATGTTACATAAAGTACTATCCTGCAACTTTCATTTTTAAAATTTTTTTACTATTTAAATGATGACAGATGTCTAATGGTGACATGTTTGAGAAGAGCCATGATGAAATTGACTTTGAATTCTTGGGAAATATAAGAGCAAAAAACTGGAGGATTCAAACTAATATATATGGGAATGGTAGCACAAATGTTGGCAGAGAAGAAAGATATGGACTCTGGTTTGATCCTTCTGAAGATTTTCATCAATATACCATCCTCTGGACTGAGAGCCAGATCATGTAAGTACCTAATTCCACCAGTAACAGCTCTCCCCTTTTAATTTATTTTTCTCCACAAAAAAAAAAAAACAGATAACGGCATGTTGGATCAGTTTGCACACACCTTAACTAATCCACGAACTAACTAATCCATGTATTAACTGGTAACTTTCACTAAAACAAGTGTCTCAGGTAGCATAAGCCACTGATGCTTGCATCAAGATAGGCTGCCTACATCACACCCCTTAGAGTGCGACCCTTCCCCGACCCTACGTGAATGCGGAATACTTCGTGCACCGGGCTACCTTTTATTTTTTGTGTTCTTTTTGTCTCTATTGAGATTAAAACCATAATAGCCTTTCTAGTTCCGTTGACAACTAGGTCACACCGGGGATGTTTTTCGTAATTTTGATTTAAATACTTCGTGTCGTATGTGCCATGTTCCTAATTCAGTGTTATCCCTTCTCTTTTTCTGTGGTACTAAAGCCAGTGGATTAGTGTTTAATTTAACTCTAATCTTATACCACATCTTATTACAGTGATTAATACCCCCCTCTTATATAACCCTTCCAGTCAAACATAACAAACACACACCACCTTAATCTATATAACACTTTTGATTGATTCTGTTTTTTTTTTTTTCAGCTTAAGAACCAATTAGAGGAAAAATTAGAACTTGAATTAGTATATAACAACAATATATCTAGTATTATCTCACACCGTGGAATACGAGAAAGAACTTGAACTAGTGTAAAAAAAAAAAGGGCAGCCTGGTGCACTAAGCTCCCGCTATGTGCGGGGTCCGATAAAGGGCCGGACCACAAGAGTCTATTGTACGCAACATTACCCTGCATTTGTGCAAAAGACTATTTCCGCGGCTTGAACCTGTGACTTCCTGGTCACATGGCAACAACTTTACCAGTTACGCCAAGGCTCCCCTCAAAACTTGAACTAGTGTGTAACTTGAAAAGATATCCTTATCCATATCACCAATTATGTACAAGACTAAGAGGGCCTCTTTCAACCAACTTTTTATTGGCTTTAGGGATTTTGGAGAGGGATTTTGCGGGGGANCAGTTCTTCCCTTCCTTTAAGCAAGACTTTTTAAATTATCTTTTACTTCTGCATTTGGTCCACTGTTTCAGAATGCAGGGAATCTCTCTTTTCTCCTTTTTTTTTTGGCCCGGGGAGCGGGGCAGACAATGGCGGAGCTAGGGGGTTAGAGGGAAATTACTTCGTATATATAAGGTTAAAAAAAATATATATATATATATGAGCAGTCTAATCCCCTAGGCTTTTAATTTTTTTTTTTTGAACCCTTTAGTGAAACTCCTGGCTCCGCCACAAGTAGGTACATAACTCAATATAAAATTTGAGCAACATTTCCTAAATTTGTTCTTGGCTTGAGTATTTAACTATTAGAGGTCCCTCTTGCTTTTCACTCTTATGGTATGAAACTACCAGTTTTTGTACACCCACATGTATTAGTGGAGGCACTGATTATATTTTGTTTGATTTGTGTGTTTTATGCATTTTGTTGCAGCTTTTATGTAGATAATATCCCCATAAGAGAGATCAAAAGGACAAAAGCAATGGGTGGGGACTTCCCTTCTAAGCCAATGTCTTTATATGCTACAATATGGGATGGTTCTAGTTGGGCTACCAATGGGGGCAAATACAAAGTCAATTACAAATATGCCCCTTACGTCGCCAAGTTTTCCGACTTTGTCCTCCACGGATGTGCAGTTGATCCAATTGAATTGTCACCAAAATGTGACACTGCACCTAAGTCTGCATTCGTTCCAACTGGTATATCCCCTGATCAAAGAAGAAAAATGGAGAGCTTCCGAAAGAAGTACTTGCAATATTCGTATTGTTATGACCGGACTCGATACAATGTACCTCTATCTGAATGTGTTATTGATCCTAAGGAAGCGGATCGTCTCCAAGGCTTTGATCCCGTGACCTTTGGTGGCGTCCAGCGTCATCACAGCAAACGACGCCGTCAGAGGCAATCGAGGAGAGAAGACGCGTCTTCTGAATAG
