# Supplementary material for: Genome-Wide Identification and Expression Profiling Analysis of the Xyloglucan Endotransglucosylase/Hydrolase Gene Family in Tobacco (Nicotiana tabacum L.)
Source: Genes (Basel). 2018 May 24;9(6):273. doi: 10.3390/genes9060273 (PMC6027287; doi:10.3390/genes9060273)
Supplement: Supplementary file 1 [file genes-09-00273-s001.zip › Supplementary File 5.docx]

**Supplementary File 5: Protein sequences of *N. tabacum* NtXTHs.**

# >NtXTH1

MNNFSTLIFFVTAFIYLFHITLASIVSTGDFNKDFIVPWSPNHVNTSADGHTRSLIFDKESGSGIASNDTYLFGQFDMKIKLIPGNSAGTVVAFYLTSYQPNRDEVDFEFLGNVPGKPYTLQTNVYVDGLDDREQRINLWFDPTQDFHTYSILWNLHQIVFMVDRVPIRTYRNHADKGAKYPRWQPMALQISIWNGESWATDGGKTKIDWSKAPFVASLGNYTIDACVWKGNARFCRGESENNWWNKEKFSTLTWTQRRLFKWVRKYHLTYDYCMDNQRFQNNLPIECSLPKY

# >NtXTH2

MKLKLVGGDSAGVVTAYYMCTEDGAGPTRDEVDFEFLGNRTGEPYLIQTNVYKNGTGGREMRHVLWFDPTEDFHSYSLLWNSHQLVFFVDEVPIRVYKNANYTNNFFPNEKPMYLFSSIWNADDWATRGGLEKTDWKNAPFVSTYKDFSVDGCQWEDPFPTCVSTTTKNWWDQYNSWHLSSDQKLNYAWVQRNLVIYDYCQDTKRYPEKPEECWLSPWD

# >NtXTH3

MANLLLIAVLIAIYCSLSQAEVKGSFDDNFSKSCPESHFKTSEDGQIWYLSLDHKAGCGFMTRQKYRFGWFSMKLKLVGGDSAGVVTAYYMCTEDGAGPTRDEVDFEFLGNRTGEPYLIQTNVYKNGTGGREMRHVLWFDPTEDFHSYSLLWNSHQLVFFVDEVPIRVYKNTNYTNNFFPNEKPMYLFSSIWNADDWATRGGLEKTDWKNAPFVSTYKDFSVDGCQWEDPFPSCVSTTTENWWDQYNSWHLSSDQKLDYAWVQRNLVIYDYCQDTERYPEKPEECWLSPWD

# >NtXTH4

MERMSSSIPKFLLIIALITVLFTLTQAEVQGSFDDNFSKSCPETHFKTSEDGQIWYLSLDKKAGCGFMTRQKYRFGWFSMKLKLVGGDSAGVVTAYYMCTEDGAGPTRDELDFEFLGNRTGEPYTIQTNVYKNGTGNREMRHILWFDPTEDFHTYSILWNTHQIVFFVDRVPIRVYKNANYTNNFFPNEKPMYLFSSIWNADDWATRGGLEKTNWKNQPFVSSYKDFSVDGCQWKDPFPACVSTTTKNWWDQYNSWHLSSDQKMDYAWVQRNLVTYDYCQDTERFPKKPEECWLNPWD

# >NtXTH5

MEKMASSIPKILLIIALITVLFSLTQAEVQGSFDDNFSKSCPETHFKTSEDGQIWYLSLDKKAGCGFMTKQKYRFGWFSMKLKLVGGDSAGVVTAYYMCTEDGAGPTRDELDFEFLGNRTGEPYTIQTNVYKNGTGNREMRHILWFDPTEDFHTYSILWNTHQIVFFVDRVPIRVYKNANYTNNFFPNEKPMYLFSSIWNADDWATRGGLEKTNWKNQPFVSSYKDFSVDGCQWKDPFPACVSTTTKNWWDQYNSWHLSSDQKMDYAWVQRNLVTYDYCQDTERFPKKPEECWLNPWE

# >NtXTH6

MERNASSMADLFFTAALMAALFSSSHAELIKGAFENNFSKSCPGTHFKTSQDGQIWYLTLDQISDCGFITKQSYRFGWFSTKLKLVGGDSAGVVTAFYMCSEVEAGPLRDEIDFEFLGNRTGQPYLIQTNVYNNGSGGREMRHLLWFDPTQDFHTYSILWNSHQIVFFVDKVPIRVYKNANHTNNFFPAERPMYVFSSIWNADNWATRGGLDKINWTSAPFIASYKDFILDACQWKDPFPACVSTTTQHWWDQYNAWHLSSKQKIDYAWVQRNFVVYDYCQDSVRNRYKPQECWLSALD

# >NtXTH7

MERNMGDLLLFAALVATLFSSSHAQLIKGAFENTFSKSCPGTHFKTSQDGQIWYLTLDQVSDCGFITKQSYRFGWFSTKLKLVGGDSAGVVTAFYMCSEVEAGPLRDEIDFEFLGNRTGQPYLIQTNVYNNGSGGREMRHLLWFDPTQDFHTYSILWNSHQIVFFVDKVPIRVYKNANHTNNFFPAERPMYVFSSIWNADNWATRGGLDKINWTSAPFVASYKEFTLDACQWKDPFPACVSTTTQHWWDQYNAWHLSSKQKIDYTWVQRNFVVYDYCQDSVRNRYKPQECWLSPLD

# >NtXTH8

MKQVIEYRCLLILGCGFASKSKYLFGRVSMKIKLVPGDSAGTVTAFYMNSDTDNVRDELDFEFLGNRSGQPYTVQTNVYVHGKGDKEQRINLWFDPSADFHTYTILWNHHHTVFYVDAVPIRVYKNNEAKGIPFPKFQPMGVYSTLWEADDWATRGGLEKINWSKSPFYAYYKDFDIEGCAMPGPANCASNPRNWWEGANYQQLSAVEARQYRWVRTNHMIYDYCTDKSRNPVPPPECVAGI

# >NtXTH9

MISSSLKYSTVIPILLYALTFSSSVSARPATFLQDFKVAWADSHIKQIDGGKAIQLILDQNSGCGFASKSKYLFGRVSMKIKLVPGDSAGTVTAFYMNSDTDNVRDELDFEFLGNRSGQPYTVQTNVYVHGKGDKEQRINLWFDPSADFHTYTILWNHHHTVFYVDAVPIRVYKNNEAKGIPFPKFQPMGVYSTLWEADDWATRGGLEKINWSRSPFYAYYKDFDIEGCAMPGPANCASNPRNWWEGANYQQLSAVEAKQYRWVRMNHMIYDYCTDKSRNPVTPPECVAGI

# >NtXTH10

MGKLTSLKYSAAILILLYALTFSFSVSARPATFLQDFKVSWAYSHIKQIDGGRAIQLILDQNSGCGFASKSKYLFGRVSMKIKLVPGDSAGTVTAFYMNSDTDNVRDELDFEFLGNRSGQPYTVQTNVYVHGKGDKEQRVNLWFDPSADFHTYTILWNHHHAVFYVDAVPIRVYKNNEAKGIPFPKFQPMGVYSTLWEADDWATRGGLEKINWSKSPFYAYYKDFDIEGCAMPGPANCASNPRNWWEGANYQQLSAVEARQYRWVRMNHMIYDYCTDKSRNPVTPPECVAGI

# >NtXTH11

MARLTSLKYSAAILILLYALTFSFSVSARPATFLQDFKVSWSDSHIKQIDGGRAIQLILDQNSGCGFASKSKYLFGRVSMKIKLVPGDSAGTVTAFYMNSDTDNVRDELDFEFLGNRSGQPYTVQTNVYVHGKGDKEQRVNLWFDPSADFHTYTILWNHHHAVFYVDAVPIRVYKNNEAKGIPFPKFQPMGVYSTLWEADDWATRGGLEKINWSKSPFYAYYKDFDIEGCAMPGPANCASNPRNWWEGANYQQLSAAEARQYRWVRMNHMIYDYCTDKSRNPVTPPECVAGI

# >NtXTH12

MVSFPMEFKWVFLGISLMLVGLVSSSRFEELYQPSWATDHLTNEGEILRMKLDNLSGAGFSSKNKYMFGKVTVQIKLVEGDSAGTVTAFYMSSEGPTHNEFDFEFLGNTTGEPYSVQTNVYVNGVGNREQRLNLWFDPSNEFHSYSILWNQHRVVFLVDETPVRVHSNLEHKGIPFPKDQAMGVYSSIWNADDWATQGGRVKTDWSHAPFIASYRGFEIDGCECPATVAAAENSKRCSSSAEKRYWWDEPTMSELSLHQSHQLIWVRANHMVYDYCTDTARFPVAPVECQHHQHKTRN

# >NtXTH13

MVSFPMEFKCVFLGISLIMVGLVSSSRFEELYQPSWATDHLTNEGEILRMKLDNLSGAGFSSKNKYMFGKVTVQIKLVEGDSAGTVTAFYMSSEGPTHNEFDFEFLGNTTGEPYSVQTNVYVNGVGNREQRLNLWFDPSKEFHSYSILWNQRRVVFLVDDTPIRVHSNLEHKGIPFPKDQAMGVYSSIWNADDWATQGGRVKTDWSHAPFIASYRGFEIDGCECPATVAAAENSKRCSSSAVKRYWWDEPVMSELSLHQSHQLIWVRANHMVYDYCTDTARFPVAPVECQHHQHKFHN

# >NtXTH14

MPSSMIVFLILAMLLNTGVGVNFAEVFESSWAPDHITVVGDQVMLTLDNASGCGFQSKNKYLFGKASVQIKLVGGDSAGTVIAFYMSSEGANHDELDFEFLGNVSGEPYLVQTNVYANGTGDREQRHSLWFDPTTDFHTYSFFWNHHTIIFSVDDIPIRVFQNKENKGVAYPKNQGMGIYGSLWNADDWATQGGRVKTNWSHSPFVATFRAFEIDACDLSGEDTVAAGAKCGKLAECWWDKPAVKQLNKSKKRQFKMVQSKHLVYDYCKDTARFTQMPKECLD

# >NtXTH15

MRRKSCMLTTVPWLPLKHSLARWVGVNFTEVFESSWSPDHITVVGDQVMLTLDNASGCGFQSKNKYMFGKASAQIKLVDGDSAGTVIAFYMSSEGANHDELDFEFLGNVSGEPYLVQTNVYANGTGDREQRHSLWFDPTADFHTYSFFWNHHTIIFSVDDIPIRVFKNTEKKGVAYPKNQGMGVYGSLWNADDWATQGGRVKTNWSHSPFVATFRAFEIDACDLSGEDTVAAGAKCGKLAQCWWDKPAMRELNKSKKRQFKMVQSKHLVYDYCKDTARFTQMPKECLD

# >NtXTH16

MGMNMLLVCVLFVVGAMAAAPKKPMDVPFGRNYENTWAPDHVKYFNGGSEIQLFLDNRTGTGFQSKGSYLFGHFAMHIKMVAGDSAGTVTAFYLSSQNNEHDEIDFEFLGNKTGEPYVVQTNIYTGGKGDKEQRIYLWFDPTKDYHTYSVLWNLHQIVFFVDEYPIRTFKNSKDLGVKFPFDQPMKIYSSLWEADDWATRGGLEKIDWSNAPFVASYKGFHIDGCEASVNAKLCANQGKKWWDQKEFQDLDKQQWRLLRRVRDKYTIYNYCTDKKRFATLPKECRRNRDVPRKSSKKSP

# >NtXTH17

MGFKWMNMLLFCALFVVGAMAAAPKKPMDVPFGRNYENSWAPDHVKYFNGGSEIQLFLDNRTGTGFQSKGSYLFGHFAMHIKMVAGDSAGTVTAFYLSSQNNEHDEIDFEFLGNKTGEPYVVQTNVYTGGKGDKEQRIYLWFDPTKDYHTYSVLWNLHQIVFFVDEYPIRTFKNSKDLGVKFPFDQPMKIYSSLWEADDWATRGGLEKIDWSNAPFVASYKGFHIDGCEASVNAKYCSNQGKKWWDQKEFQDLDKQQWRLLRRVRDKYTIYNYCTDKKRFATMPKECRRNRDVPRKSSKKSP

# >NtXTH18

MGLKGLLFSIVLINLSLLGLCGYPRKPVDVPFWKNYEPSWASHHIKYLNGGSTADLVLDRSSGAGFQSKKSYLFGHFSMKLRLVGGDSAGVVTAFYLSSNNAEHDEIDFEFLGNRTGQPYILQTNVFTGGKGDREQRIYLWFDPTKGYHSYSVLWNTFQIVIFVDDVPIRAFKNSKDLGVKFPFNQPMKIYSSLWDADDWATRGGLEKTDWSNAPFTASYTSFHVDGCEAATPQEVQVCNTKGMRWWDQKAFQDLDALQYRRLRWVRQKYTIYNYCTDRKRYPTLPPECTKDRDI

# >NtXTH19

MGVKGLLFSIVLINLSLLGLCGYPRKPVDVPFWKNYEPSWASHHIKYLSGGSTVDLVLDRSSGAGFQSKKSYLFGHFSMKLKLVGGDSAGVVTAFYLSSNNAEHDEIDFEFLGNRTGQPYILQTNVFTGGKGDREQRIYLWFDPTKGYHSYSVLWNTFQIVIFVDDVPIRAFKNSKDLGVKFPFNQPMKIYSSLWDADDWATRGGLEKTDWSNAPFTASYTSFHVDGCEAATPQEVQVCNTKGMRWWDQKAFQDLDALQYRRLRWVRQKYTIYNYCTDRKRYPTLPPECTKDRDI

# >NtXTH20

MQLKLVPGNSAGTVTTFFLSSQGAGHDEIDFEFLGNVSGQPYTVHTNVYSQGKGNKEQQFHLWFDPTAAFHTYSIIWNAQKIIFLVDNSPIRVYNNHESAGIPFPKSQPMKVYCSLWNADEWATQGGRVKTDWTHAPFTAYYRNFNIDGCAVTSGASSCKSTDSANNARPWQNQELDAKGRNRLRWVQSRHMVYNYCADSKRFPQGFSHECKRSRFL

# >NtXTH21

MSPRFSFKMLILPIVMASLWAAASAGNFYNLADITWGEGRGKITEGGRGLSLSLDKLSGSGFQSKNEYLFGRFDMQLKLVPGNSAGTVTTFFLSSQGAGHDEIDFEFLGNVSGQPYTVHTNVYSQGKGNKEQQFHLWFDPTAAFHTYSIIWNAQKIIFLVDNSPIRVYNNHESNGIPFPKIQPMKVYCSLWNADEWATQGGRVKTDWTHVPFTAYYRNFNIDGCAVTSGTSSCKSTDSANNARPWQNQELDAKGRNRLRWVQSRHMVYNYCADSKRFPQGFSHECKRSRFL

# >NtXTH22

MASHLFLISILMGSLVAASANFNNLAEITWGEGRGKITEGGKGLSLSLDKLSGSGFQSKNEYLFGRFDMQLKLVPGNSAGTVTTFFLSSQGEGHDEIDFEFLGNTTGEPYTVHTNVYSQGKGNKEQQFHLWFDPTAAFHTYTIVWNSNRIVFLVDNIPIRVYNNHENNGIPFPKSQPMKVYCSLWNADEWATQGGRVKTDWTHAPFTAYYRNFKIDGCAVTSGASSCKSTDSAGNAKAWQNQELDAKGRNRVRWVQSRHMVYNYCADKKRFPQGYSHECKSSRF

# >NtXTH23

MASHFLLISILMGSLVVASANFNNLAEITWGEGRGKITEGGKGLSLSLDKLSGSGFQSKNEYLFGRFDMQLKLVPGNSAGTVTTFFLSSQGKGHDEIDFEFLGNTTGEPYTVHTNVYSQGKGNKEQQFHLWFDPTAAFHTYTIVWNANRILFLVDNIPIRVYNNHESNGIPFPKSQPMKVYCSLWNADEWATQGGRVKTDWTHAPFTAYYRNFKIDGCAVTSGASSCKSTDSAGNAKAWQNHELDAKGRNRVRWVQSRHMVYNYCADKKRFPQGYSHECKSSRF

# >NtXTH24

MASKFSSVMLLLCIIMSIQLLAASAGNFYRDAVITWGEGRGKIQEGGRGLALTLDKLSGSGFQSKNEYLFGRFDMQLKLVPGNSAGTVTTFFLSSQGEGHDEIDFEFLGNVSGQPYTVHTNVYTQGKGNKEQQFHLWFDPTAAFHTYTIVWNPHRIVFLVDNSPIRVYNNHESIGIPFPKSQAMRVYCSLWNADEWATQGGRVKTDWTLAPFTAYYRNINIDGCAVLSGTSSCKSSNSANNAKPWQTHELDGKGRNRLRWVQSRHMVYNYCADSKRFPQGFSAECKSSRF

# >NtXTH25

MASKFSSAMLLLCILMSIQLLAASAGNFYRDTVITWGEGRGKIQEGGRGLALTLDKLSGSGFQSKNEYLFGRFDMQLKLVPGNSAGTVTTFFLSSQGEGHDEIDFEFLGNVSGQPYTVHTNVYTQGKGNKEQQFHLWFDPTAAFHTYTIVWNPHRIVFLVDNSPIRVYNNHENIGIPFPKSQAMRVYCSLWNADEWATQGGRVKTDWTLAPFTAYYRNINIDGCAVLSGTSSCKSSNSANNAKPWQTHELDGKGRNRLRWVQSRHMVYNYCADSKRFPQGFSEECKRSRF

# >NtXTH26

MSLSSASSRIPKMFLQLSVLAVFLLCTACADNFYQDATVTWGDQRAHIQEGGRLLTLSLDKISGSGFQSKSEFLFGRFDMQLKLIPGNSAGTVTTFYLSSQGAGHDEIDFEFLGNSSGQPYTVHTNVYSQGKGNKEQQFHLWFDPTTSFHTYSIIWNAQRIIFLVDNIPIRVYNNHEALGVAFPKNQAMRVYASLWNADDWATQGGRVKTDWSMAPFTASYRNFNTNACVWSAASSTSSCGGSKSTDSANNDQTWQTQELDANGRNRLRWVQQKYMTYNYCTDAQRFNQVIPPECKRSRF

# >NtXTH27

MGSRIFLVLALVFSSCMVSYGGNFFQEFDFTWGGNRAKIFNGGQLMSLSLDKVSGSGFQSKKEYLFGRIDMQIKLVAGNSAGTVTTYYLSSQGPTHDEIDFEFLGNVTGEPYILHTNIYAQGKGNKEQQFYLWFDPTKNFHTYSIIWKPQHIIFLVDNTPIRVYKNAESIGVPFPKNQPMRIYSSLWNADDWATRGGLVKTDWSKAPFTAYYRNFNSQTFSSSQFSNEKWQNQELDANGRRRLRWVQRNFMIYNYCTDFKRFPQGFPPECKRF

# >NtXTH28

MARFSSSSSRSRSSLPYIVLLFVAALFVFKIDVIISQTFSSARRNLENTPNRILVKSKSQETDDSIPVVLVNGTFHRHFILSWGDDRGKIHENGELLTLSLDKQSGSGFQSKKEYLFAKIDMQIKLVPGNSAGTVTTFYLSSQGNKHDEIDFEFLGNSTGNPYTLHTNIFSLGQGNREQQFFLWFDPTADYHTYSILWNPKCIIFYVDGTPIREFKNAEKIGVPFLKYQPMRLYSSLWNADDWATQGGRVKTNWKLAPFIASYKNFTYEACIYSRLTSSSSCNINSPPFGNNAWLTHELDRRSRAKMKILQKKHMIYDYCKDKWRFPKGPAPECKLQ

# >NtXTH29

MARFSSSSSRSRSSLPYIILLFVAALFVFKIDVIISQSFSSARRNLENTPNHILVKSKSQETDDSIPVVLVNGTFHRHFILSWGDDRGKIHENGELLTLSLDKLSGSGFQSKKEYLFAKIDMQIKLVPGNSAGTVTTFYLSSQGNKHDEIDFEFLGNSTGNPYTLHTNIFSLGQGNREQQFFLWFDPTADYHTYSILWNPKCIIFYVDGTPIREYKNAEKIGVPFPKYQPMRLYSSLWNADDWATQGGRIKTNWKLAPFIASYKNFTYDACIYSRLTSSSSCNINSPPFGNDSWLTHELDRRSRAKMKILQKKHMIYDYCNDKWRFPKGPAPECKLQ

# >NtXTH30

MMKTSSCMFSFLFLSFLVLVALAENFNQEFDVTWGDGRVKILENGQLLTLSLDKTSGSGFRSKRQYMFGKIDMKIKLVPGNSAGTVTTYYLSSLGPTHDEIDFEFLGNLSGDPYILHTNVFVQGKGEREQQFYLWFDPTKDFHTYSILWNPRSIIFSVDGTPIRQFKNLEASRGIPYPKNQPMWIYSSLWDAEDWATRGGLVKTDWSKAPFIASYRNFNAQACVWSSGSTSSCSINSTANSWITESLDNSGQARIKWVQKNYMVYNYCTDTKRFPQGFPLECSLN

# >NtXTH31

MMKTSISCIISFLFLSFLLVVMAALAGDFNQEFDVTWGDGRVKILENGQLLTLSLDKTSGSGFRSKRQYMFGKIDMKIKLVPGNSAGTVTTYYLSSLGPTHDEIDFEFLGNLSGDPYILHTNVFTQGKGDREQQFYLWFDPTKDFHTYSILWNPRSIIFSVDGTPIRQFKNLETSMGIPYPKNQPMWIYSSLWDAEDWATRGGLVKTDWSQAPFVASYRNFNAQACVWSSGSTSSCSRNSTANSWITESLDNSGQARIKWVQKNYMVYNYCTDIKRFPQGFPLECSLN

# >NtXTH32

MMKSFLFQMMFLVVAFAGNFNQNFDITWGDGRAKILENGQLLTLSLDKTSGSGFRSKNQYLFGKIDLKIKLVPGNSAGTVTTYYLSSIGSSHDEIDFEFLGNLSGDPYILHTNVFTQGKGNREQQFYLWFDPTKYFHTYSILWNPQSIIFSVDGTPIRQFKNLEASGIPYPKNQPMWIYSSLWNADDWATRGGLVKTDWSKAPFIASYRNYNAQACVWSSTSSSSCSPNNSTENSWLSESLDNTGQSKIKWVQNNYMIYNYCTDTKRFPQGFPPECSLN

# >NtXTH33

MMKSFLFLMIFLVVALAGNFNKDFDITWGDGRAKILENGQLLTLSLDKTSGSGFRSKNQYLFGKIDLKIKLVPGNSAGTVTTYYLSSIGSSHDEIDFEFLGNLSGDPYILHTNVFTQGKGNREQQFYLWFDPTKDFHTYTILWNPQSIIFSVDGTPIRQFKNLEASGIPYPKNQPMWIYSSLWNADDWATRGGLVKTDWSKAPFIASYRNYNAQACVWSSSSSSSCTSNSSTGNSWLSESLDSTGQSRIKWVQSNYMIYNYCTDTKRFPQGFPPECSLN

# >NtXTH34

MSSFSSKLVLALIVSAFAIAIAGTIDENFEITWGEGRAKMLNNGELLTLSLDKISGSGFQSKNEYLFGKIDMQLKLVPGNSAGTVTAYYLSSQGPTHDEIDFEFLGNLSGDPYTLHTNVFSQGKGNREQQFHLWFDPTADFHTYSILWNPQRIIFYVDGTPIREYKNAESIGVSYPKKQPMRIYSSLWNADDWATRGGLIKTDWSKAPFSASYRNFKSATSTSAATSNSWLNEELDNTSQERLKWVQKNYMVYNYCNDSKRFPQGFPADCAM

# >NtXTH35

MASLLAQYLVFLALCSLQYHSLAYNNFNQDFDVTWGDGRAKVLNNGKLLTLSLDKASGSGIQSKREYLFGRIDMQLKLVRGNSAGTVTTYYLSSQGATHDEIDFEFLGNLSGDPYIIHTNVYTQGKGDKEQQFYLWFDPTAGFHTYSILWNPQTIIFYVDGTPIRVFKNMKSRGIPYPNKQPMRVYASLWNADDWATRGGLIKTDWSNAPFIASFRNFKANACVWEFGKSSCNSSTNPWFFQELDSTSQAKLQWVQKNYMVYNYCTDIKRFPQGFPLECNFNSTTS

# >NtXTH36

MASLLVQCLNFLALCSLQYHILASSNFNQDFDVTWGDGRAKVLNNGKLLTLSLDKASGSGIQSKREYLFGRIDMQLKLVRENSAGTVTTYYLSSQGATHDEIDFEFLGNLSGDPYIIHTNVYTQGKGDKEQQFYLWFDPTAGFHTYSILWNPQTIIFYVDGTPIRVFKNMKSSGVPYPTNQPMRVYASLWNADDWATRGGLIKTDWSKAPFIASFRNFKANACVWEFGKSSCNSSTNSTKPWFFQELDSTSQARLQWVQKNYMVYNYCTDIKRFPQGLPQECNFNSTTS

# >NtXTH37

MAKFIAFNSLVLIIATFAFHCAIVNAKISSSMYINWGAHHCQMLGDDLQLVLDKSAGSGAQSKRTFLFGSFEMLIKLVPNNSAGTVTTYYLSSTGTKHDEIGFEFLGNVSGQPYIIHTNIYTQGVGNKEQQFYPWFDPTADFHNYTIHWNPNAVVWYIDGIPIRVFRNYQLKGIPFPNQQGMRIYSSLWNADEWATRGGRDKIDWTNAPFIATYRKFRPRACYWNGPLSIVQCAIPTKSNWWNFPLYSKLSAPKVDQMNSIRSKYMIYDYCKDTTRFKGVMPTECTLPQN

# >NtXTH38

MAKFIAFNSLVLIIATIAFHCAIVNGKISSSMYVNWGAHHCQMLGDDLQLVLDKSAGSGAQSKRTFLFGSFEMLIKLVPNNSAGTVTTYYLSSTGTKHDEIDFEFLGNVSGQPYILHTNIYTQGVGNREQQFYPWFDPTADFHNYTIHWNPNAVVWYVDGIPIRVFRNYQFKGIPYPNQQGMRIYSSLWNADEWATRGGRDKIDWTNAPFIATYRKFRPRACYWNGPLSIVQCAIPTKSNWWNSPLYSKLSAPKVDQMNSIRSKYMIYDYCKDTTRFKGVMPIECSLPQY

# >NtXTH39

MAKFVAFNSLVLIIATIAFHCAIVNGKISSSMYVNWGAHHCQMLGEDLQLVLDKSAGSGAQSKRTFLFGSFEMLIKLVPNNSAGTVTTYYLSSTGTKHDEIDFEFLGNVSGQPYILHTNIYTQGVGNREQQFYPWFDPTADFHNYTIHWNPNAVVWYVDSIPIRVFRNYQLKGIPFPNQQGMRIYSSLWNADEWATRGGRDKIDWTNAPFIAKYRKFRPRACYWNGPLSIVQCAIPTKSNWWNSPLYSKLSAPKVDQMNSIRSKYMIYDYCKDTTRFKGVTPTECSLPQN

# >NtXTH40

MAKFITFSLVLIIATFAFRCTLVNGKISSSMYINWGAHHCKMQGDDLQLVLDKSAGSGAQSKRTFLFGSFEMLIKLVPNNSAGTVTTYYLSSTGTKHDEIDFEFLGNVSGQPYIIHTNIYTQGVGNKEQQFYPWFDPTADFHNYTIHWNLNAVVWYVDGIPIRVFRNYELKGIPFPNQQGMRIYSSLWNADEWATRGGRDKIDWTNAPFIATYRNFRPRACYWNGPLSIGQCAIPTKSNWWNSPLYNKLSAPKVDQMNSIRSKYMIYDYCKDTKRFKGVTPTECSLPQN

# >NtXTH41

MFKIMASSRLLSLANLFILAIAFHLVSVNGMFSDNMYIGWGAHHSWMQGNDLQLVLDQSSGSGVQSKGAFLFGSIQMQIKLVPGNSAGTVTAYYLSSTGDKHDEIDFEFLGNVSGHPYIIHTNIFTQGAGGREQQFYPWFDPTADYHNYTIHWNPSAVVWYVDDIPIRVYKNYQSQGILYPNAQGMGVYSSLWNADNWATRGGLDKIDWTNAPFIAKYRNFAPRACPWYGPGSISHCAAPTPNNWYTSPEYSQLSYAKQGQMNWVRNNYMIYDYCKDTTRFNGQIPGECFKPQF

# >NtXTH42

MFKIMASSRLLSLSNLFILAIAFHLVSVNGMFSDNMYINWGAHHSWMQGNDLQLVLDQSAGSGVQSKGAFLFGSIEMQIKLVPGNSAGTVTAYYLSSTGDKHDEIDFEFLGNVSGQPYIIHTNIFTQGAGGREQQFYPWFDPTADYHNYTIHWNPSAVVWYVDGIPIRVYKNYQSQGILYPNAQGMKVYSSLWNADNWATRGGLDKIDWTNAPFIAKYRNFAPRACPWYGPGSIRQCAAPTPNNWYTSYEYSQLSYAKQGQMNWVRNNYMIYDYCKDKTRFNGQIPGECFKPQI

# >NtXTH43

MAIFFLHFLLLLIVVPSTNAGYWPPSPGYYPSSKFRSMSFYQGFRNLWGPNHQNVDNNGINIWLDRNSGSGFKSIKPFRSGYFGASIKLQPGYTAGVITAFYLSNNEAHPGYHDEVDIEFLGTTFGKPYTLQTNVYIRGSGDGKIVGREMKFHLWFDPTKEFHHYAILWSPREIIFLVDDVPIRRYARKSIATFPLRPMWLYGSIWDASSWATEDGKYKADYRYQPFYGKFTNFKASGCTAYSSRWCHPVSASPSRSGGLTRQQRQAMNWVHSHYLAYDYCRDSKRDHSLTPECWR

# >NtXTH44

MSIFFLPFLLFLIVLPSTNAGYWPPSPGYYPSSKFKSMSFYQGFKNLWGPNHQNVDNNGINIWLDRNSGSGFKSIKPFRSGYFGASIKLQPGYTAGVITAFYLSNNEAHPGYHDEVDIEFLGTTFGKPYTLQTNVYIRGSGDGKIIGREMKFHLWFDPTKDFHHYAILWSPREIIFLVDDVPIRRYARKSIATFPLRPMWLYGSIWDASSWATEDGKYKADYRYQPFYGKFTNFKASGCTAYSSRWCHPVSASPSRSGGLTRQQRQAMNWVHSHYLAYDYCRDSKRDHSLTPECWR

# >NtXTH45

MANLFLLSLLLIFLFNSSNAQGPLSPGYYPSSKVQSLGFNQGFRNLWGPQHQSLDQSALTIWLDKTSGGSGFKSLENYRSGYFGTSVKLQPGYTAGIITSFYLSNNQDYPGNHDEIDIEFLGTTPNKPYTLQTNVYIRGSGDGNIIGREMKFHLWFDPTKAYHNYAILWDPNEIIFFVDDVPIRRYPRKNDATFPQRPMYVYGSIWDASSWATEEGRIKADYRYQPFVGKYNNFKIAGCTANENPWCGRSPSSSPSRAGGLSRQQIAAMLWVQRNYKVYDYCRDPRRDHTHTPEC

# >NtXTH46

MALFLLSLLLLFLFNSSNAQGPPSPGYYPSSKVQSLGFSQCFRNLWGPQHQSLDQSALTIWLDKTTGGSGFKSLKNYRSGYFGTSVKLQPGYTAGIITSFYLSNNQDYPGNHDEIDIEFLGTTPNKPYTLQTNVYIRGSGDGNIIGREMKFHLWFDPTQAYHNYAILWNPNEIIFFVDDVPIRRYPRKNDATFPQRPMYVYGSIWDASSWATEEGRIKADYRYQPFIGKYNNFKIAGCTANENPWCGRSPSSSSSRAGGLSRQQMAAMLWVQRNYKVYDYCRDPRRDHTHTPEC

# >NtXTH47

MDFFHHNKTFLLSQFLIFCMIVVVSCRGPVYKPPEVEKLTDHFSRLSVNQGYNVFFGGANVRMTNNGSSADLILDKSSGSGLISKEKYYYGFFNAALKLPAHFTSGVVIAFYMSNSDVFPHNHDEIDFELLGHDKRRDWVLQTNLYGNGSVHTGREEKFYLWFDPTLDFHDYTILWNNHHIVFLVDNVPIREVVHNTAISSVYPSKPMSVIATIWDGSEWATHGGKYPVNYQYAPFVTSMKEVELEGCVRQQNTSATSTCFRRSTSSLDPVDGEEFMKLSQQQMTGLDWVRRKHMFYSYCQDTNRYKVLPPECTSN

# >NtXTH48

MEFYHQHKTCLFSGFLIFCMIAVASSLGPIYTPPEAERLTDRFSRLSVNQGYNVFFGGANVRLTNNGSNADLILDKSSGSGLVSRDKYYYGFFNAALKLPANFTSGVVVAFYLSNQNIFPHNHDELDFELLGYDKRRDWVLQTNIYGNGSVSTGREEKFYLWFDPTQDFHDYSILWNNHHILFLVDNVPVREVVNNTTISSVYPSKPMSIYATIWDGSQWATRGGKYPVNYTYAPFVTSIKGVELEGCVSEQNASAASACARRSTSSLDPVDGEEFVKLSQQQMTGLDWARRKHMFYSYCQDTRRYKVLPPECTAT

# >NtXTH49

MEFFHQHNTLLLSEFLIFCMISVASSLGPIYTPPEVERLTDRFSRLSVNQGYNMFFGGVNVRLTNNGSSADLILDKSSGSGLVSRDKYYYGFFNAALKLPANFTSGVVVAFYLSNQNIFPHDHDELDFELLGYDKRRDWVLQTNNYGNGSVSTGREGKFYLWFDPTQDFHDYTILWNNHHILFLVDNVPVREVVHNTAISSVYPSKPMSIYVTIWDGSQWATRRGKYPVNYTYAPFVTSIKGVELEGCVSEQNGSAATACARRSTSSLDPVDGEEFVKLSQQQMMGLDWARRKHMFYSYCQDTRRYKVLPPECTAT

# >NtXTH50

MDYRVLSSLSKSLTPFSLLMLLYIFPAAETATATTAKAFNLSTITFEEGYSPLFSDFNIERSPDDTSFRLLLNRFSGSGVISTEYYNYGFFSASIKLPAIYTAGIVVAFYTSNVDTFEKNHDELDIEFLGNVNGQPWRFQTNLYGNGSVSRGREERYRMWFDPSNDFHHYSILWTPKNIIFYVDETPIREVNRNPAMGGDFPSKPMSLYATIWDASSWATNGGKAKVDYKHEPFATEFKDLVLEGCIVDPIEQISSTNCTDRIARLLSQNYSIMTPERRKSMKWFRERYMYYSYCYDNIRYPVPPPECVIVQSERDLFKDSGRLRQKMKFGGSHSHRKHRPGRSSRRRNRAAGGGSSKSGQAAAM

# >NtXTH51

MDFIRKKICLSVFLFFHVWFSTALNVSTIPFSDGFSHLFGEGNILHATDDKSLQLHLNQRTGSGFKSSDLYNHGFFSAKIKLPSDYTAGIVVAFYTTNGDLFTKTHDELDFEFLGNIRGKAWRFQTNMYGNGSTSRGREERYYLWFDPSKEFHRYSILWTNKNIIFYIDDVPIREIVRNDAMGGDYPSKPMGLYATIWDASDWATSGGKYKTNYKYAPFIAEFTDLVLNGCAMDPLEQVVNNPSCDEKDDELQKADFSRITPRQRMAMKRFRSKYMYYSYCYDSLRYSVPPPECEIDPIEQQHFKETGRLKFNKHHHRHPKRTKSQVLDARNYGNQDEE

# >NtXTH52

MDFIRKKICLSVFLFFHVCFITADAALNVSTIPFSDGFSHLFGEGNILHATDDKSLQLHLNQRTGSGFKSSDLYTHGFFSAKIKLPSDYTAGIVVAFYTTNGDLFTKTHDELDFEFLGNIRGKAWRFQTNMYGNGSTSRGREERYYLWFDPSKEFHRYSILWTIKNIIFYIDDVPIREIVRNDAMGGDYPSKPMGLYATIWDASDWATSGGKYKTNYKYAPFIAEFTDLVLNGCAMDPLEQVVNNPSCDEKDDELQKADFSRITPRQRMAMKRFRSKYMYYSYCYDSLRYSVPPPECEIDHVEQQHFKETGRLKFNKHGHHRHAKRTRSQVLDARNHGNQDEE

# >NtXTH53

MVNYHLVTFIFFSVVELVYGSSRNLPILAFDEGYSHLFGDDNVMILKDGKSAHISLDERTGAGFVSQDLYLHGFFSASIKLPADYTAGVVVAFYMSNVDMFEKNHDEIDFEFLGNIRGKDWRIQTNIYGNGSTSVGREERYGLWFDPSEDFHHYSILWTENFIIFYVDNVPIREIKRTEAMGGDFPSKPMSLYATIWDGSGWATNGGKYKVNYKYAPYIAKFSDFVLHGCAVDPIELSSKCDTAPKTASIPTGITPDQRRKMEKFRKKQMQYSYCYDKTRYKVPPPECVIDPKEAERLRAFDPVTFGGSRHHHGKQHRRSRSRAEGDISFL

# >NtXTH54

MVNYHLVIFIFFSVVELVYGSSRNLPILAFDEGYSHLFGDNNLMILKDGKSAHISLDERTGAGFVSQDLYLHGFFSASIKLPADYTAGVVVAFYMSNVDMFEKNHDEIDFEFLGNIRGKDWRIQTNIYGNGSTSFGREERYGLWFDPSEDFHHYSILWTENFIIFYVDNVPIREIKRTEAMGGDFPSKPMSLYATIWDGSGWATNGGKYKVNYKYAPYIAKFSDFVLHGCAVDPIELSSKCDTAPKTSSIPTGITPDQRRKMENFRKKQMQYSYCYDKTRYKVPPTECVIDPKEAERLRVFDPVTFGGSRHHHGKRHSRSRSRAEGDVSFL

# >NtXTH55

MVNFRLEIFILCSFLVLVCGSSKQLQTLPFDEGYSQLFGHDNLMVLEDGKSVHLSLDERTGAGFVSQDLYLHGYFSASIKLPADYTAGVVVAFYMSNGDMFEKNHDEIDFEFLGNIRAKKWRIQTNIYGNGSTNVGREERYGLWFDPSEDFHQYSILWTESQIIFYVDNIPIREIKRTKAMGGDFPSKPMSLYATIWDGSSWATNGGKYKVNYKYAPYVAKFSDFILHGCAVDPIELSPKCDTTPNSASIPTSISPDQRRKMESFRKKYLQYSYCYDRTRYNVPLSECVIDPKEADRLRGFDPVTFGGVQRHHSKRHHQRQSRREDTSSE

# >NtXTH56

MVNFRLGIFILCSFLVLVSGSSKKLQTLPFDEGYSQLFGHDNLMVLEDGKSVHISLDERTGAGFVSQDLYLHGYFSASIKLPADYTAGVVVAFYMSNGDMFEKSHDEIDFEFLGNIRAKNWRIQTNIYGNGSTNVGREERYGLWFDPSEDFHQYTILWTESQIIFYVDNIPIREIKRTKAMGGDFPSKPMSLYATIWDGSSWATNGGKYKVNYKYAPYVAKFSDFVLHGCAVDPIELSPKCDTAPKSAFVPTGISPDQRRKMESFRKKYLQYSYCYDRTRYNVPLSECVIDPKEADRLQGFDPVTFGGVQRHHSKRRRQRQSRREDASSE
