# Supplementary material for: Genome-Wide Identification and Expression Profiling Analysis of the Xyloglucan Endotransglucosylase/Hydrolase Gene Family in Tobacco (Nicotiana tabacum L.)
Source: Genes (Basel). 2018 May 24;9(6):273. doi: 10.3390/genes9060273 (PMC6027287; doi:10.3390/genes9060273)
Supplement: Supplementary file 1 [file genes-09-00273-s001.zip › Supplementary File 8.docx]

**Supplementary File 8: Protein sequences of *N. sylvestris* NsyXTHs.**

# >NsyXTH1

MNNFSTLVFFVTAFIYLFHISLASIVSTGDFNKDFPVITWSPNHVNTSADGHTRSLIFDKESGSGIASNDTYLFGQFDMKIKLIPGNSAGTVVAFYLTSYQPNRDEVDFEFLGNVPGKPYTLQTNVYVDGLDDREQRINLWFDPTQDFHTYSILWNLHQIVFMVDRVPIRTYRNHADKGAKYPRWQPMGLQLSIWNGETWATDGGKTKIDWSKAPFIASLGDYTIDACVWKGNARFCRGESENNWWNKEKFSTLTWTQRRLFKWVRKYHLTYDYCMDNQRFQNNLPIECSLPKY

# >NsyXTH2

MANLLLIAVLIAIYCSLSQAEVKGSFDDNFSKSCPESHFKTSEDGQIWYLSLDHKAGCGFMTRQKYRFGWFSMKLKLVGGDSAGVVTAYYMCTEDGAGPTRDEVDFEFLGNRTGEPYLIQTNVYKNGTGGREMRHVLWFDPTEDFHSYSLLWNSHQLVFFVDEVPIRVYKNTNYTNNFFPNEKPMYLFSSIWNADDWATRGGLEKTDWKNAPFVSTYKDFSVDGCQWEDPFPSCVSTTTENWWDQYNSWHLSSDQKLDYAWVQRNLVIYDYCQDTERYPEKPEECWLSPWD

# >NsyXTH3

MEKMASSIPKILLIIALITVLFSLTQAEVQGSFDDNFSKSCPETHFKTSEDGQIWYLSLDKKAGCGFMTKQKYRFGWFSMKLKLVGGDSAGVVTAYYMCTEDGAGPTRDELDFEFLGNRTGEPYTIQTNVYKNGTGNREMRHILWFDPTEDFHTYSILWNTHQIVFFVDRVPIRVYKNANYTNNFFPNEKPMYLFSSIWNADDWATRGGLEKTNWKNQPFVSSYKDFSVDGCQWKDPFPACVSTTTKNWWDQYNSWHLSSDQKMDYAWVQRNLVTYDYCQDTERFPKKPEECWLNPWE

# >NsyXTH4

MERNASSMADLFFTAALMAALFSSSHAELIKGAFENNFSKSCPGTHFKTSQDGQIWYLTLDQISDCGFITKQSYRFGWFSTKLKLVGGDSAGVVTAFYMCSEVEAGPLRDEIDFEFLGNRTGQPYLIQTNVYNNGSGGREMRHLLWFDPTQDFHTYSILWNSHQIVFFVDKVPIRVYKNANHTNNFFPAERPMYVFSSIWNADNWATRGGLDKINWTSAPFIASYKDFILDACQWKDPFPACVSTTIQHWWDQYNAWHLSSKQKIDYAWVQRNFVVYDYCQDSVRNRYKPQECWLSALD

# >NsyXTH5

MISSSSLYYSPVIVILLYALTFSFSVSARPATFLQDFKVAWADSHIKQIDGGKAIQLILDQNSGCGFASKSKYLFGRVSMKIKLVPGDSAGTVTAFYMNSDTDNVRDELDFEFLGNRSGQPYTVQTNVYVHGKGDKEQRINLWFDPSADFHTYTILWNHHHTVFYVDAVPIRVYKNNEAKGIPFPKFQPMGVYSTLWEADDWATRGGLEKINWSKSPFYAYYKDFDIEGCAMPGPANCASNPRNWWEGANYQQLSAVEARQYRWVRTNHMIYDYCTDKSRNPVPPPECVAGI

# >NsyXTH6

MGKLTSLKYSAAILILLYALTFSFSVSARPATFLQDFKVSWAYSHIKQIDGGRAIQLILDQNSGCGFASKSKYLFGRVSMKIKLVPGDSAGTVTAFYMNSDTDNVRDELDFEFLGNRSGQPYTVQTNVYVHGKGDKEQRVNLWFDPSADFHTYTILWNHHHAVFYVDAVPIRVYKNNEAKGIPFPKFQPMGVYSTLWEADDWATRGGLEKINWSKSPFYAYYKDFDIEGCAMPGPANCASNPRNWWEGANYQQLSAVEARQYRWVRMNHMIYDYCTDKSRNPVTPPECVAGI

# >NsyXTH7

MVSFPMEFKWVFLGISLMLVGLVSSSRFEELYQPSWATDHLTNEGEILRMKLDNLSGAGFSSKNKYMFGKVTVQIKLVEGDSAGTVTAFYMSSEGPTHNEFDFEFLGNTTGEPYSVQTNVYVNGVGNREQRLNLWFDPSNEFHSYSILWNQHRVVFLVDETPVRVHSNLEHKGIPFPKDQAMGVYSSIWNADDWATQGGRVKTDWSHAPFIASYRGFEIDGCECPATVAAAENSKRCSSSAEKRYWWDEPTMSELSLHQSHQLIWVRANHMVYDYCTDTARFPVAPVECQHHQHKTRN

# >NsyXTH8

MSTSILSTWWRCLWVSTAHLSFDRFIQIAWQRVGVNFTEVFESSWSPDHITVVGDQVMLTLDNASGCGFQSKNKYMFGKASAQIKLVDGDSAGTVIAFYMSSEGANHDELDFEFLGNVSGEPYLVQTNVYANGTGDREQRHSLWFDPTADFHTYSFFWNHHTIIFSVDDIPIRVFKNTEKKGVAYPKNQGMGVYGSLWNADDWATQGGRVKTNWSHSPFVATFRAFEIDACDLSGEDTVAAGAKCGKLAQCWWDKPAMRELNKSKKRQFKMVQSKHLVYDYCKDTARFTQMPKECLD

# >NsyXTH9

MGMNMLLVCVLFVVGAMAAAPKKPMDVPFGRNYENTWAPDHVKYFNGGSEIQLFLDNRTGTGFQSKGSYLFGHFAMHIKMVAGDSAGTVTAFYLSSQNNEHDEIDFEFLGNKTGEPYVVQTNIYTGGKGDKEQRIYLWFDPTKDYHTYSVLWNLHQIVFFVDEYPIRTFKNSKDLGVKFPFDQPMKIYSSLWEADDWATRGGLEKIDWSNAPFVASYKGFHIDGCEASVNAKLCANQGKKWWDQKEFQDLDKQQWRLLRRVRDKYTIYNYCTDKKRFATLPKECRRNRDVPRKSSKKSP

# >NsyXTH10

MGVKGLLFSIVLINLSLLGLCGYPRKPVDVPFWKNYEPSWASHHIKYLSGGSTVDLVLDRSSGAGFQSKKSYLFGHFSMKLKLVGGDSAGVVTAFYLSSNNAEHDEIDFEFLGNRTGQPYILQTNVFTGGKGDREQRIYLWFDPTKGYHSYSVLWNTFQIVIFVDDVPIRAFKNSKDLGVKFPFNQPMKIYSSLWDADDWATRGGLEKTDWSNAPFTASYTSFHVDGCEAATPQEVQVCNTKGMRWWDQKAFQDLDALQYRRLRWVRQKYTIYNYCTDRKRYPTLPPECTKDRDI

# >NsyXTH11

MSPRFSFKMLILPIVMASLWAAASAGNFYNLADITWGEGRGKITEGGRGLSLSLDKLSGSGFQSKNEYLFGRFDMQLKLVPGNSAGTVTTFFLSSQGAGHDEIDFEFLGNVSGQPYTVHTNVYSQGKGNKEQQFHLWFDPTAAFHTYSIIWNAQKIIFLVDNSPIRVYNNHESNGIPFPKIQPMKVYCSLWNADEWATQGGRVKTDWTHVPFTAYYRNFNIDGCAVTSGTSSCKSTDSANNARPWQNQELDAKGRNRLRWVQSRHMVYNYCADSKRFPQGFSHECKRSRFL

# >NsyXTH12

MASHFLLISILMGSLVVASANFNNLAEITWGEGRGKITEGGKGLSLSLDKLSGSGFQSKNEYLFGRFDMQLKLVPGNSAGTVTTFFLSSQGKGHDEIDFEFLGNTTGEPYTVHTNVYSQGKGNKEQQFHLWFDPTAAFHTYTIVWNANRILFLVDNIPIRVYNNHESNGIPFPKSQPMKVYCSLWNADEWATQGGRVKTDWTHAPFTAYYRNFKIDGCAVTSGASSCKSTDSAGNAKAWQNHELDAKGRNRVRWVQSRHMVYNYCADKKRFPQGYSHECKSSRF

# >NsyXTH13

MASKFSSVMLLLCIIMSIQLLAASAGNFYRDAVITWGEGRGKIQEGGRGLALTLDKLSGSGFQSKNEYLFGRFDMQLKLVPGNSAGTVTTFFLSSQGEGHDEIDFEFLGNVSGQPYTVHTNVYTQGKGNKEQQFHLWFDPTAAFHTYTIVWNPHRIVFLVDNSPIRVYNNHESIGIPFPKSQAMRVYCSLWNADEWATQGGRVKTDWTLAPFTAYYRNINIDGCAVLSGTSSCKSSNSANNAKPWQTHELDGKGRNRLRWVQSRHMVYNYCADSKRFPQGFSAECKSSRF

# >NsyXTH14

MGSRIFLILALVFSSCMVSYGGNFFQEFDFTWGGNRAKIFNGGQLMSLSLDKVSGSGFQSKKEYLFGRIDMQIKLVAGNSAGTVTTYYLSSQGPTHDEIDFEFLGNVTGEPYILHTNIYAQGKGNKEQQFYLWFDPTKNFHTYSIIWKPQHIIFLVDNTPIRVYKNAESIGVPFPKNQPMRIYSSLWNADDWATRGGLVKTDWSKAPFTAYYRNFNSQTFSSSQFSNEKWQNQELDANGRRRLRWVQRNFMIYNYCTDFKRFPQGFPPECKRF

# >NsyXTH15

MTSLSSFNIKLSLLVVIFISCLVVEYSASDLNQDFGITWGNDRGKILNNGELLTLTLDNTSGSGFESKKEYLFGKIDMQIKLVPGNSAGTVTAYYLSSQGSNHDEIDFEFLGNLSGEPYTVHTNVYTQGKGEREQQFHLWFDPTADFHTYSVLWNPQTIVFSVDNVPIREFKNLEDKGVAFPKSQPMKLYSSLWDADEWATRGGLIKTDWSNAPFSASYRNFNAHTCTNNSNSSCSILAAKLDPVSQERLRWVQQKFMIYNYCTDTKRFPQGFPLECSTT

# >NsyXTH16

MMKTSISCIISFLFLSFLLVVMAALAGDFNQEFDVTWGDGRVKILENGQLLTLSLDKTSGSGFRSKRQYMFGKIDMKIKLVPGNSAGTVTTYYLSSLGPTHDEIDFEFLGNLSGDPYILHTNVFTQGKGDREQQFYLWFDPTKDFHTYSILWNPRSIIFSVDGTPIRQFKNLETSMGIPYPKNQPMWIYSSLWDAEDWATRGGLVKTDWSQAPFVASYRNFNAQACVWSSGSTSSCSRNSTANSWITESLDNSGQARIKWVQKNYMVYNYCTDIKRFPQGFPLECSLN

# >NsyXTH17

MMKSFLFQMMFLVVAFAGNFNQNFDITWGDGRAKILENGQLLTLSLDKTSGSGFRSKNQYLFGKIDLKIKLVPGNSAGTVTTYYLSSIGSSHDEIDFEFLGNLSGDPYILHTNVFTQGKGNREQQFYLCFSVDGTPIRQFKNLEASGIPYPKNQPMWIYSSLWNADDWATRGGLVKTDWSKAPFIASYRNYNAQACVWSSTSSSSCSPNNSTENSWLSESLDNTGQSKIKWVQNNYMIYNYCTDTKRFPQGFPPECSLN

# >NsyXTH18

MMKSFLFQMMFLVVAFAGNFNQNFDITWGDGRAKILENGQLLTLSLDKTSGSGFRSKNQYLFGKIDLKIKLVPGNSAGTVTTYYLSSIGSSHDEIDFEFLGNLSGDPYILHTNVFTQGKGNREQQFYLWFDPTKYFHTYSILWNPQSIIFSVDGTPIRQFKNLEASGIPYPKNQPMWIYSSLWNADDWATRGGLVKTDWSKAPFIASYRNYNAQACVWSSTSSSSCSPNNSTENSWLSESLDNTGQSKIKWVQNNYMIYNYCTDTKRFPQGFPPECSLN

# >NsyXTH19

MASLLAQYLVFLALCSLQYHSLAYNNFNQDFDVTWGDGRAKVLNNGKLLTLSLDKASGSGIQSKREYLFGRIDMQLKLVRGNSAGTVTTYYLSSQGATHDEIDFEFLGNLSGDPYIIHTNVYTQGKGDKEQQFYLWFDPTAGFHTYSILWNPQTIIFYVDGTPIRAFKNMKSRGIPYPNKQPMRVYASLWNADDWATRGGLIKTDWSNAPFIASFRNFKANACVWEFGKSSCNSSTNPWFFQELDSTSQAKLQWVQKNYMVYNYCTDIKRFPQGFPLECNFNSTTS

# >NsyXTH20

MSSFTSKLVLALIVSAFAVAIAGTFDDNFEITWGEGRAKMLNNGELLTLSLDKISGSGFQSKNEYLFGKIDMQLKLVPGNSAGTVTAYYLSSQGPTHDEIDFEFLGNLSGDPYTLHTNVFSQGKGNREQQFHLWFDPTADFHTYSILWNPQRIIFYVDGTPIREYKNSESIGVSYPKNQPMRIYSSLWNADDWATRGGLIKTDWSKAPFSASYRNFKSATSTSAANSNSWLNEELDNTSQERLKWVQKNYMVYNYCNDSKRFPQGFPADCAM

# >NsyXTH21

MSSFSSKLVLALIVSAFAVAIAGTFDDNFEITWGEGRAKMLNNGELLTLSLDKISGSGFQSKNEYLFGKIDMQLKLVPGNSAGTVTAYYLSSQGPTHDEIDFEFLGNLSGDPYTLHTNVFSQGKGNREQQFHLWFDPTADFHTYSILWNPQRIIFYVDGTPIREYKNSESIGVSYPKNQPMRIYSSLWNADDWATRGGLIKTDWSKAPFSASYRNFKSATSTSAANSNSWLNEELDNTSQERLKWVQKNYMVYNYCNDSKRFPQGFPADCAI

# >NsyXTH22

MQIKLVPGNSAGTVTTFYLSSQGNKHDEIDFEFLGNSTGNPYTLHTNIFSLGQGNREQQFFLWFDPTADYHTYSILWNPKCIIFYVDGTPIREYKNAEKIGVPFPKYQPMRLYSSLWNADDWATQGGRIKTNWKLAPFIASYKKFTYDACIYSRLTSSSSCNINSPPFGNDSWLTHELDRRSRAKMKILQKKHMIYDYCNDKWRFPKGPAPECKLQ

# >NsyXTH23

MAKFIAFNSLVLIIATIAFHCAIVNGKISSSMYVNWGAHHCQMLGDDLQLVLDKSAGSGAQSKRTFLFGSFEMLIKLVPNNSAGTVTTYYLSSTGTKHDEIDFEFLGNVSGQPYILHTNIYTQGVGNREQQFYPWFDPTADFHNYTIHWNPNAVVWYVDGIPIRVFRNYQFKGIPYPNQQGMRIYSSLWNADEWATRGGRDKIDWTNAPFIATYRKFRPRACYWNGPLSIVQCAIPTKSNWWNSPLYSKLSAPKVDQMNSIRSKYMIYDYCKDTTRFKGVMPIECSLPQY

# >NsyXTH24

MFKIMASSRLLSLSNLFILAIAFHLVSVNGMFSDNMYINWGAHHSWMQGNDLQLVLDQSAGSGVQSKGAFLFGSIEMQIKLVPGNSAGTVTAYYLSSTGDKHDEIDFEFLGNVSGQPYIIHTNIFTQGAGGREQQFYPWFDPTADYHNYTIHWNPSAVVWYVDGIPIRVYKNYQSQGILYPNAQGMKVYSSLWNADNWATRGGLDKIDWTNAPFIAKYRNFAPRACPWYGPGSIRQCAAPTPNNWYTSYEYSQLSYAKQGQMNWVRNNYMIYDYCKDKTRFNGQIPGECFKPQI

# >NsyXTH25

MRSSLHLVCLFAFLLVLGAFASGAKDLPFNVNYHILFGNEHVVSFNRGRELQISMDKTSGSGFGSKANYGSGFFHMRIKLPDRDSAGVVTAFYLRSNTNNDHDEVDFEFLGNREGKPYTLQTNIFANGQGNREQRMHLWFDPTADFHNYKILWNQHQIVFFVDNIPFRVFKNKANIGVNYPSKPMQILATIWDGDNWATDGGLTKTNWSFAPFKAHFQDFDIIGCPISSINTNCNSPNFWWNQKKYWKLSSKQRKKYEEVKAKYMTYDYCVDKSRFSTPPPECFS

# >NsyXTH26

MAYFLMNIVTSLILLFIVGLSNADISFNNSYEPTWGENHLSIINQGTEVTLLLDNSSGAGFKSKFLYESGLFVIRMKLPDKKTGGVITSLYLTSQVDGSPPGTHDEIDFEFLGTQGKLQTNVFANDWGFREQIFQLPFDPSQDFHTYQILYNPFQIVFFIDDIPVREFMNLKVKANVNYPTSPMQIEASVWYSNSSGWAGDIDWSLAPFIAHYQHFKISGCFPQPGNDCSSPVNQPWNRFKVLSPLQRQKMANFRKQYMTYDYCVPGFVQFPECSYNNS

# >NsyXTH27

MFHFPSGLLITLFLIATQYASNVNADEIPFNQTYYQIWGGNHLTISNEGKEVQLFIDQYSGAGFSSKQNFGSGDFRIKLKLPKKNSKGVITTFYLMSKEVDEPARPKHDEVDFEFFGGDGKYTLNTNIFANDEGHREQQFNLWFDPAADFHTYGILWNQYQIVFFVDDIPIRVFKNNTNHGVNYPSNKMHIEATIWNATAWVGEVDWSQGPFTAYFREFSINGCQYQQSNPQYCYRNSYYWNRINYWKLSPKQQQLYEVVREKQMTYDYCLRNAKDFPEC

# >NsyXTH28

MAIFFLHFLLLLIVVPSTNAGYWPPSPGYYPSSKFRSMSFYQGFRNLWGPNHQNVDNNGINIWLDRNSGSGFKSIKPFRSGYFGASIKLQPGYTAGVITAFYLSNNEAHPGYHDEVDIEFLGTTFGKPYTLQTNVYIRGSGDGKIVGREMKFHLWFDPTKEFHHYAILWSPREIIFLVDDVPIRRYARKSIATFPLRPMWLYGSIWDASSWATEDGKYKADYRYQPFYGKFTNFKASGCTAYSSRWCHPVSASPSRSGGLTRQQRQAMNWVHSHYLAYDYCRDSKRDHSLTPECWR

# >NsyXTH29

MALFLLSLLLLFLFNSSNAQGPPSPGYYPSSKVQSLGFSQCFRNLWGPQHQSLDQSALTIWLDKTTGGSGFKSLKNYRSGYFGTSVKLQPGYTAGIITSFYLSNNQDYPGNHDEIDIEFLGTTPNKPYTLQTNVYIRGSGDGNIIGREMKFHLWFDPTQAYHNYAILWNPNEIIFFVDDVPIRRYPRKNDATFPQRPMYVYGSIWDASSWATEEGRIKADYRYQPFIGKYNNFKIAGCTANENPWCGRSPSSSSSRAGGLSRQQMAAMLWVQRNYKVYDYCRDPRRDHTHTPEC

# >NsyXTH30

MDFFHHNKTFLLSQFFIFCMIVVVSCRGPVYKPPEVEKLTDHFSRLSVNQGYNVFFGGANVRMTNNGSSADLILDKSSGSGLISKEKYYYGFFNAALKLPAHFTSGVVIAFYMSNSDVFPHNHDEIDFELLGHDKRRDWVLQTNLYGNGSVHTGREEKFYLWFDPTLDFHDYTILWNNHHIVFLVDNVPIREVVHNTAISSVYPSKPMSVIATIWDGSEWATHGGKYPVNYQYAPFVTSMKEVELEGCVRQQNTSATSTCFRRSTSSLDPVDGEEFMKLSQQQMTGLDWVRRKHMFYSYCQDTNRYKVLPPECTSN

# >NsyXTH31

MEFFHQHNTLLLSEFLIFCMISVASSLGPIYTPPEVEQLTDRFSRLSVNQGYNMFFGGVNVRLTNNGSSADLILDKSSGSGLVSRDKYYYGFFNAALKLPANFTSGVVVAFYLSNQNIFPHDHDELDFELLGYDKRRDWVLQTNNYGNGSVSTGREGKFYLWFDPTQDFHDYTILWNNHHILFLVDNVPVREVVHNTAISSVYPSKPMSIYVTIWDGSQWATRGGKYPVNYTYAPFVTSIKGVELEGCVSEQNGSAATACARRSTSSLDPVDGEEFVKLSQQQMMGLDWARRKHMFYSYCQDTRRYKVLPPECTAT

# >NsyXTH32

MDYRVLSSLSKSLTPFSLLLLLYIFPAAAATTRAFNLSTITYEEGYSPLFSDFNIERSPDDTSFRLLLNRFSGSGVISTEYYNYGFFSASIKLPAIYTAGIVVAFYTSNVDTFEKNHDELDIEFLGNVNGQPWRFQTNLYGNGSVSRGREERYRMWFDPSKDFHHYSILWTPKNIIFYIDETPIREVNRNPAMGGDFPSKPMSLYATIWDASSWATNGGKAKVDYKHEPFAAEFKDLVLEGCIVDPIEQISSTNCTDRIAKLLAQNYSIMTPERRKSMKWFRERYMYYSYCYDNIRYPVPPPECVIVQSERDLFKDSGRLRQKMKFGGSHSHRKHRPGRSSRRRNRAAAGASSKSGQAAAM

# >NsyXTH33

MDFIRKKICLSVFLFFHVCFITADAALNVSTIPFSDGFSHLFGEGNILHATDDKSLQLHLNQRTGSGFKSSDLYTHGFFSAKIKLPSDYTAGIVVAFYTTNGDLFTKTHDELDFEFLGNIRGKAWRFQTNMYGNGSTSRGREERYYLWFDPSKEFHRYSILWTIKNIIFYIDDVPIREIVRNDAMGGDYPSKPMGLYATIWDASDWATSGGKYKTNYKYAPFIAEFTDLVLNGCAMDPLEQVVNNPSCDEKDDELQKADFSRITPRQRMAMKRFRSKYMYYSYCYDSLRYSVPPPECEIDHVEQQHFKETGRLKFNKHGHHRHAKRTRSQVLDARNHGNQDEE

# >NsyXTH34

MVNYHLVIFIFFSVVELVYGSSRNLPILAFDEGYSHLFGDNNLMILKDGKSAHISLDERTGAGFVSQDLYLHGFFSASIKLPADYTAGVVVAFYMSNVDMFEKNHDEIDFEFLGNIRGKDWRIQTNIYGNGSTSFGREERYGLWFDPSEDFHHYSILWTENFIIFYVDNVPIREIKRTEAMGGDFPSKPMSLYATIWDGSGWATNGGKYKVNYKYAPYIAKFSDFVLHGCAVDPIELSSKCDTAPKTSSIPTGITPDQRRKMENFRKKQMQYSYCYDKTRYKVPPTECVIDPKEAERLRVFDPVTFGGSRHHHGKRHSRSRSRAEGDVSFL

# >NsyXTH35

MVNFHLGIFILCSFLVLVSGTSKKLQTLPFDEVYSQLFGHDNLMVLEDGKSVHISLDERTGAGFVSQDLYLHGYFSASIKLPADYTAGVVVAFYMSNGDMFEKSHDEIDFEFLGNIRAKNWRIQTNIYGNGSTNVGREERYGLWFDPSEVFHQYSILWTESQIIMQGISLFFPFFGSGQAVAELGC

# >NsyXTH36

MQISLFLSYHFSPINMYISGSSKKLQTLPFDEGYSQLFGHDNLMVLEDGKSVHISLDERTGAGFVSQDLYLHGYFSASIKLPADYTAGVVVAFYMSNGDMFEKSHDEIDFEFLGNIRAKNWRIQTNIYGNGSTNVGREERYGLWFDPSEDFHQYTILWTESQIIFYVDNIPIREIKRTKAMGGDFPSKPMSLYATIWDGSSWATNGGKYKVNYKYAPYVAKFSDFVLHGCAVDPIELSPKCDTAPKSAFVPTGISPDQRRKMESFRKKYLQYSYCYDRTRYNVPLSECVIDPKEADRLQGFDPVTFGGVQRHHSKRRRQRQSRREDASSE

# >NsyXTH37

MGGDFPSKPMSLYATIWDGSSWATNGGKYKVNYKYSPYVAKFSDFVLHGCAVDPIELSPKCDTAPKSAFIPTSISPDQRRKMESFRKKYLQYSYCYDRTRYNVPLSECVIDPKEADHLQGFDPVTFGGFQRHHSKRRHQRQSRREDTSSEYKEGY
