# Supplementary material for: Genome-Wide Identification and Expression Profiling Analysis of the Xyloglucan Endotransglucosylase/Hydrolase Gene Family in Tobacco (Nicotiana tabacum L.)
Source: Genes (Basel). 2018 May 24;9(6):273. doi: 10.3390/genes9060273 (PMC6027287; doi:10.3390/genes9060273)
Supplement: Supplementary file 1 [file genes-09-00273-s001.zip › Supplementary File 9.docx]

**Supplementary File 9: Protein sequences of *N. tomentosiformis* NtoXTHs.**

# >NtoXTH1

MNNFSTLIFFVTAFIYLFHITLASIVSTGDFNKDFIVPWSPNHVNTSADGHTRSLIFDKESGSGIASNDTYLFGQFDMKIKLIPGNSAGTVVAFYLTSYQPNRDEVDFEFLGNVPGKPYTLQTNVYVDGLDDREQRINLWFDPTQDFHTYSILWNLHQIVFMVDRVPIRTYRNHADKGAKYPRWQPMALQISIWNGESWATDGGKTKIDWSKAPFVASLGNYTIDACVWKGNARFCRGESENNWWNKEKFSTLTWTQRRLFKWVRKYHLTYDYCMDNQRFQNNLPIECSLPKY

# >NtoXTH2

MRHVLWFDPTEDFHSYSLLWNSHQLVFFVDEVPIRVYKNANYTNNFFPNEKPMYLFSSIWNADDWATRGGLEKTDWKNAPFVSTYKDFSVDGCQWEDPFPTCVSTTTKNWWDQYNSWHLSSDQKLNYAWVQRNLVIYDYCQDTKRYPEKPEECWLSPWD

# >NtoXTH3

MGKILFSISTLDMTKSDQCTAFIIFVYFSKNFNLRSFHFSSSSRKRMERMSSSIPKFLLIIALITVLFTLTQAEVQGSFDDNFSKSCPETHFKTSEDGQIWYLSLDKKAGCGFMTRQKYRFGWFSMKLKLVGGDSAGVVTAYYMCTEDGAGPTRDELDFEFLGNRTGEPYTIQTNVYKNGTGNREMRHILWFDPTEDFHTYSILWNTHQIVFFVDRVPIRVYKNANYTNNFFPNEKPMYLFSSIWNADDWATRGGLEKTNWKNQPFVSSYKDFSVDGCQWKDPFPACVSTTTKNWWDQYNSWHLSSDQKMDYAWVQRNLVTYDYCQDTERFPKKPEECWLNPWD

# >NtoXTH4

MERNMGDLLLFAALVATLFSSSHAQLIKGAFENTFSKSCPGTHFKTSQDGQIWYLTLDQVSDCGFITKQSYRFGWFSTKLKLVGGDSAGVVTAFYMCSEVEAGPLRDEIDFEFLGNRTGQPYLIQTNVYNNGSGGREMRHLLWFDPTQDFHTYSILWNSHQIAFFVDKVPIRVYKNANHTNNFFPAERPMYVFSSIWNADNWATRGGLDKINWTSAPFVASYKEFTLDACQWKDPFPACVSTTTQHWWDQYNAWHLSSKQKIDYAWVQRNFVVYDYCQDSVRNRYKPQECWLSPLD

# >NtoXTH5

MISSSLKYSTVIPILLYALTFSSSVSARPATFLQDFKVAWADSHIKQIDGGKAIQLILDQNSGCGFASKSKYLFGRVSMKIKLVPGDSAGTVTAFYMNSDTDNVRDELDFEFLGNRSGQPYTVQTNVYVHGKGDKEQRINLWFDPSADFHTYTILWNHHHTVFYVDAVPIRVYKNNEAKGIPFPKFQPMGVYSTLWEADDWATRGGLEKINWSRSPFYAYYKDFDIEGCAMPGPANCASNPRNWWEGANYQQLSAVEAKQYRWVRMNHMIYDYCTDKSRNPVTPPECVAGI

# >NtoXTH6

MARLTSLKYSAAILILLYALTFSFSVSARPATFLQDFKVSWSDSHIKQIDGGRAIQLILDQNSGCGFASKSKYLFGRVSMKIKLVPGDSAGTVTAFYMNSDTDNVRDELDFEFLGNRSGQPYTVQTNVYVHGKGDKEQRVNLWFDPSADFHTYTILWNHHHAVFYVDAVPIRVYKNNEAKGIPFPKFQPMGVYSTLWEADDWATRGGLEKINWSKSPFYAYYKDFDIEGCAMPGPANCASNPRNWWEGANYQQLSAAEARQYRWVRMNHMIYDYCTDKSRNPVTPPECVAGI

# >NtoXTH7

MVSFPMEFKCVFLGISLIMVGLVSSSRFEELYQPSWATDHLTNEGEILRMKLDNLSGAGFSSKNKYMFGKVTVQIKLVVGDSAGTVTAFYMSSEGPTHNEFDFEFLGNTTGEPYSVQTNVYVNGVGNREQRLNLWFDPSKEFHSYSILWNQRRVVFLVDDTPIRVHSNLEHKGIPFPKDQAMGVYSSIWNADDWATQGGRVKTDWSHAPFIASYRGFEIDGCECPATVAAAENSKRCSSSAVKRYWWDEPVMSELSLHQSHQLIWVRANHMVYDYCTDTARFPVAPVECQHHQHKFHN

# >NtoXTH8

MGFKWMNMLLFCALFVVGAMAAAPKKPMDVPFGRNYENSWAPDHVKYFNGGSEIQLFLDNRTGTGFQSKGSYLFGHFAMHIKMVAGDSAGTVTAFYLSSQNNEHDEIDFEFLGNKTGEPYVVQTNVYTGGKGDKEQRIYLWFDPTKDYHTYSVLWNLHQIVFFVDEYPIRTFKNSKDLGVKFPFDQPMKIYSSLWEADDWATRGGLEKIDWSNAPFVASYKGFHIDGCEASVNAKYCSNQGKEWWDQKEFQDLDKQQWRLLRRVRDKYTIYNYCTDKKRFATMPKECRRNRDVPRKSSKKSP

# >NtoXTH9

MGLKGLLFSIVLINLSLLGLCGYPRKPVDVPFWKNYEPSWASHHIKYLNGGSTADLVLDRSSGAGFQSKKSYLFGHFSMKLRLVGGDSAGVVTAFYLSSNNAEHDEIDFEFLGNRTGQPYILQTNVFTGGKGDREQRIYLWFDPTKGYHSYSVLWNTFQIVIFVDDVPIRAFKNSKDLGVKFPFNQPMKIYSSLWDADDWATRGGLEKTDWSNAPFTASYTSFHVDGCEAATPQEVQVCNTKGMRWWDQKAFQDLDALQYRRLRWVRQKYTIYNYCTDRKRYPTLPPECTKDRDI

# >NtoXTH10

MSPRFSFKMLILPIVMASLWAVASAGNFYNLADITWGEGRGKITEGGRGLSLSLDKFSGSGFQSKNEYLFGRFDMQLKLVPGNSAGTVTTFFLSSQGAGHDEIDFEFLGNVSGQPYTVHTNVYSQGKGNKEQQFHLWFDPTAAFHTYSIIWNAQKIIFLVDNSPIRVYNNHESAGIPFPKSQPMKVYCSLWNADEWATQGGRVKTDWTHAPFTAYYRNFNIDGCAITSGASSCKSTDSANNARPWQNQELDAKGRNRLRWVQNRHMVYNYCADSKRFPQGFSHECKRSRFL

# >NtoXTH11

MASHLFLISILMGSLVAASANFNNLAEITWGEGRGKITEGGKGLSLSLDKLSGSGFQSKNEYLFGRFDMQLKLVPGNSAGTVTTFFLSSQGEGHDEIDFEFLGNTTGEPYTVHTNVYSQGKGNKEQQFHLWFDPTAAFHTYTIVWNSNRIVFLVDNIPIRVYNNHENNGIPFPKSQPMKVYCSLWNADEWATQGGRVKTDWTHAPFTAYYRNFKIDGCAVTSGASSCKSTDSAGNAKAWQNQELDAKGRNRVRWVQSRHMVYNYCADKKRFPQGYSHECKSSRF

# >NtoXTH12

MASKFSSAMLLLCILMSIQLLAASAGNFYRDTVITWGEGRGKIQEGGRGLALTLDKLSGSGFQSKNEYLFGRFDMQLKLVPGNSAGTVTTFFLSSQGEGHDEIDFEFLGNVSGQPYTVHTNVYTQGKGNKEQQFHLWFDPTAAFHTYSIVWNPHRIVFLVDNSPIRVYNNHESIGIPFPKSQAMRVYCSLWNADEWATQGGRIKTDWKLAPFTAYYRNINIDGCAVLAGTSSCKSSNSANNAKPWQTHELDGKGRNRLRWVQSRHMVYNYCADSKRFPQGFSAECKSSRF

# >NtoXTH13

MASKFSSAMLLLCILMSIQLLAASAGNFYGDTVITWGEGRGKIQEGGRGLALTLDKLSGSGFQSKNEYLFGRFDMQLKLVPGNSAGTVTTFFLSSQGEGHDEIDFEFLGNVSGQPYTVHTNVYTQGKGNKEQQFHLWFDPTAAFHTYTIVWNPHRIVFLVDNSPIRVYNNHENIGIPFPKSQAMRVYCSLWNADEWATQGGRVKTDWTLAPFTAYYRNINIDGCAVLSGTSSCKSSNSANNAKPWQTHELDGKGRNRLRWVQSRHMVYNYCADSKRFPQGFSEECKRSRF

# >NtoXTH14

MSLSSASSRIPKMFLQLSVLAVFLLCTACADNFYQDATVTWGDQRAHIQEGGRLLTLSLDKISGSGFQSKSEFLFGRFDMQLKLIPGNSAGTVTTFYLSSQGAGHDEIDFEFLGNSSGQPYTVHTNVYSQGKGNKEQQFHLWFDPTTSFHTYSIIWNAQRIIFLVDNIPIRVYNNHEALGVAFPKNQAMRVYASLWNADDWATQGGRVKTDWSMAPFTASYRNFNTNACVWSAASSTSSCGGSTSTDSANNDQTWQTQELDANGRNRLRWVQQKYMTYNYCTDAQRFNQVIPPECKRSRF

# >NtoXTH15

MGSRIFLVLALVFSSCMVSYGGNFFQEFDFTWGGNRAKIFNGGQLMSLSLDKVSGSGFQSKKEYLFGRIDMQIKLVAGNSAGTVTTYYLSSQGPTHDEIDFEFLGNVTGEPYILHTNIYAQGKGNKEQQFYLWFDPTKNFHTYSIIWKPQHIIFLVDNTPIRVYKNAESIGVPFPKNQPMRIYSSLWNADDWATRGGLVKTDWSKAPFTAYYRNFNSQTFSSSQFSNEKWQNQELDANGRRRLRWVQRNFMIYNYCTDFKRFPQGFPPECKRF

# >NtoXTH16

MMKSFLFLMIFLVVALAGNFNKDFDITWGDGRAKILENGQLLTLSLDKTSGSGFRSKNQYLFGKIDLKIKLVPGNSAGTVTTYYLSSIGSSHDEIDFEFLGNLSGDPYILHTNVFTQGKGNREQQFYLWFDPTKDFHTYTILWNPQSIIFSVDGTPIRQFKNLEASGIPYPKNQPMWIYSSLWNADDWATRGGLVKTDWSKAPFIASYRNYNAQACVWSSSSSSSCTSNSSTGNSWLSESLDSTGQSRIKWVQSNYMIYNYCTDTKRFPQGFPPECSLN

# >NtoXTH17

MASLLVQCLNFLALCSLQYHILASSNFNQDFDVTWGDGRAKVLNNGKLLTLSLDKASGSGIQSKREYLFGRIDMQLKLVRENSAGTVTTYYLSSQGATHDEIDFEFLGNLSGDPYIIHTNVYTQGKGDKEQQFYLWFDPTAGFHTYSILWNPQTIIFYVDGTPIRVFKNMKSSGVPYPTNQPMRVYASLWNADDWATRGGLIKTDWSKAPFIASFRNFKANACVWEFGKSSCNSSTNSTKPWFFQELDSTSQARLQWVQKNYMVYNYCTDIKRFPQGLPQECNFNSTTS

# >NtoXTH18

MSSFSSKLVLALIVSAFAIAIAGTIDENFEITWGEGRAKMLNNGELLTLSLDKISGSGFQSKNEYLFGKIDMQLKLVPGNSAGTVTAYYLSSQGPTHDEIDFEFLGNLSGDPYTLHTNVFSQGKGNREQQFHLWFDPTADFHTYSILWNPQRIIFYVDGTPIREYKNAESIGVSYPKKQPMRIYSSLWNADDWATRGGLIKTDWSKAPFSASYRNFKSATSTSAATSNSWLNEELDNTSQERLKWVQKNYMVYNYCNDSKRFPQGFPADCAM

# >NtoXTH19

MARFSSSSSRSRSSLPYIVLLFVAALFVFKIDVIISQTFSSARRNLENTPNRILVKSKSQETDDSIRVVLVNGTFHRHFILSWGDDRGKIHENGELLTLSLDKQSGSGFQSKKEYLFAKIDMQIKLVPGNSAGTVTTFYLSSQGNKHDEIDFEFLGNSTGNPYTLHTNIFSLGQGNREQQFFLWFDPTADYHTYSILWNPKCIIFYVDGTPIREFKNAEKIGVPFLKYQPMRLYSSLWNADDWATQGGRVKTNWKLAPFIASYKNFTYEACIYSRLTSSSSCNINSPPFGNNAWLTHELDRRSRAKMKILQKKHMIYDYCKDKWRFPKGPAPECKLQ

# >NtoXTH20

MAKFVAFNSLVLIIATIAFHCAIVNGKISSSMYVNWGAHHCQMLGEDLQLVLDKSAGSGAQSKRTFLFGSFEMLIKLVPNNSAGTVTTYYLSSTGTKHDEIDFEFLGNVSGQPYILHTNIYTQGVGNREQQFYPWFDPTADFHNYTIHWNPNAVVWYVDSIPIRVFRNYQLKGIPFPNQQGMRIYSSLWNADEWATRGGRDKIDWTNAPFIAKYRKFRPRACYWNGPLSIVQCAIPTKSNWWNSPLYSKLSAPKVDQMNSIRSKYMIYDYCKDTTRFKGVTPTECSLPQN

# >NtoXTH21

MAKFITFSLVLIIATFAFRCTLVNGKISSSMYINWGAHHCKMQGDDLQLVLDKSAGSGAQSKRTFLFGSFEMLIKLVPNNSAGTVTTYYLSSTGTKHDEIDFEFLGNVSGQPYIIHTNIYTQGVGNKEQQFYPWFDPTADFHNYTIHWNLNAVVWYVDGIPIRVFRNYELKGIPFPNQQGMRIYSSLWNADEWATRGGRDKIDWTNAPFIATYRNFRPRACYWNGPLSIGQCAIPTKSNWWNSPLYNKLSAPKVDQMNSIRSKYMIYDYCKDTKRFKGVTPTECSLPQN

# >NtoXTH22

MFKIMASSRLLSLANLFILAIAFHLVSVNGMFSDNMYIGWGAHHSWMQGNDLQLVLDQSSGSGVQSKGAFLFGSIQMQIKLVPGNSAGTVTAYYLSSTGDKHDEIDFEFLGNVSGHPYIIHTNIFTQGAGGREQQFYPWFDPTADYHNYTIHWNPSAVVWYVDDIPIRVYKNYQSQGILYPNAQGMGVYSSLWNADNWATRGGLDKIDWTNAPFIAKYRNFAPRACPWYGPGSISHCAAPTPNNWYTSPEYSQLSYAKQGQMNWVRNNYMIYDYCKDTTRFNGQIPGECFKPQF

# >NtoXTH23

MIHFQTGLLITLFLVATQYASNGNAYEIPFNQTYYQIWGGNHLTISDEGKEVQLLIDQYSGAGFSSKQNFGSGDFRIKLKLPKKNSKGVITTFYLISKEVNEPARPKHDEVDFEFFGGDGKYTLNTNIFANDEGHREQQFNLWFDPTADFHTYGILWNQYQIVLFVDDIPIRVFKNNTNLGVNYPSNKMHIEATMWNATAWIGEVDWSQGPFTAYFREFSINGCQYQKSNPQYCYRNSYYWNRINYWKLSPKQQQLYEDVREKHMTYDYCLRNAKDFPEC

# >NtoXTH24

MSIFFLPFLLFLIVLPSTNAGYWPPSPGYYPSSKFKSMSFYQGFKNLWGPNHQNVDNNGINIWLDRNSGSGFKSIKPFRSGYFGASIKLQPGYTAGVITAFYLSNNEAHPGYHDEVDIEFLGTTFGKPYTLQTNVYIRGSGDGKIIGREMKFHLWFDPTKDFHHYAILWSPREIIFLVDDVPIRRYARKSIATFPLRPMWLYGSIWDASSWATEDGKYKADYRYQPFYGKFTNFKASGCTAYSSRWCHPVSASPSRSGRLTRQQRQAMNWVHSHYLAYDYCRDSKRDHSLTPECWR

# >NtoXTH25

MANLFLLSLLLIFLFNSSNAQGPLSPGYYPSSKVQSLGFNQGFRNLWGPQHQSLDQSALTIWLDKTSGGSGFKSLENYRSGYFGTSVKLQPGYTAGIITSFYLSNNQDYPGNHDEIDIEFLGTTPNKPYTLQTNVYIRGSGDGNIIGREMKFHLWFDPTKAYHNYAILWDPNEIIFFVDDVPIRRYPRKNDATFPQRPMYVYGSIWDASSWATEEGRIKADYRYQPFVGKYNNFKIAGCTANANPWCGRSPSSSPSRAGGLSRQQIAAMLWVQRNYKVYDYCRDPRRDHTHTPEC

# >NtoXTH26

MEFYHQHKTCLFSGFLIFCMIAVASSLGPIYTPPEAERLTDRFSRLSVNQGYNVFFGGANVRLTNNGSNADLILDKSSGSGLVSRDKYYYGFFNAALKLPANFTSGVVVAFYLSNQNIFPHNHDELDFELLGYDKRRDWVLQTNIYGNGSVSTGREEKFYLWFDPTQDFHDYSILWNNHHILFLVDNVPVREVVNNTTISSVYPSKPMSIYATIWDGSQWATRGGKYPVNYTYAPFVTSIKGVELEGCVSEQNASAASACARRSTSSLDPVDGEEFVKLSQQQMTGLDWARRKHMFYSYCQDTRRYKVLPPECTAT

# >NtoXTH27

MDYRVLSSLSKSLTPFSLLMLLYIFPAAETATATTAKAFNLSTITFEEGYSPLFSDFNIERSPDDTSFRLLLNRFSGSGVISTEYYNYGFFSASIKLPAIYTAGIVVAFYTSNVDTFEKNHDELDIEFLGNVNGQPWRFQTNLYGNGSVSRGREERYRMWFDPSNDFHHYSILWTPKNIIFYVDETPIREVNRNPAMGGDFPSKPMSLYATIWDASSWATNGGKAKVDYKHEPFATEFKDLVLEGCIVDPIEQISSTNCTDRIARLLSQNYSIMTPERRKSMKWFRERYMYYSYCYDNIRYPVPPPECVIVQSERDLFKDSGRLRQKMKFGGSHSHRKHRPGRSSRRRNRAAGGGSSKSGQAAAM

# >NtoXTH28

MDFIRKKICLSVFLFFHVWFSTALNVSTIPFSDGFSHLFGEGNILHATDDKSLQLHLNQRTGSGFKSSDLYNHGFFSAKIKLPSDYTAGIVVAFYTTNGDLFTQTHDELDFEFLGNIRGKAWRFQTNMYGNGSTSRGREERYYLWFDPSKEFHRYSILWTNKNIIFYIDDVPIREIVRNDAMGGDYPSKPMGLYATIWDASDWATSGGKYKTNYKYAPFIAEFTDLVLNGCAMDPLEQVVNNPSCDEKDDELQKADVSRITPRQRMAMKRFRSKYMYYSYCYDSLRYSVPPPECEIDPIEQQHFKETGRLKFNKHHHRHPKRTKSQVLDARNYGNQDEE

# >NtoXTH29

MVNYHIVTFIFFSVIELVYGSSRNLPILAFDEGYSHLFGDDNLMILKDGKSAHISLDERTGAGFVSQDLYLHGFFSASIKLPADYTAGVVVAFYMSNVDMFEKNHDEIDFEFLGNIRGKDWRIQTNIYGNGSTSVGREERYGLWFDPSEDFHHYSILWTENFIIFYVDNVPIREIKRTEAMGGDFPSKPMSLYATIWDGSGWATNGGKYKVNYKYAPYIAKFSDFVLHGCAVDPIELSSKCDTAPKTASIPTGITPDQRRKMEKFRKKQMQYSYCYDKTRYKVPPPECVIDPKEAERLRAFDPVTFGGSRHHHGKQHRRSRSRAEGDISFL

# >NtoXTH30

MVNFRLEIFILCSFLVLVCGSSKQLQTLPFDEGYSQLFGHDNLMVLEDGKSVHLSLDERTGAGFVSQDLYLHGYFSASIKLPADYTAGVVVAFYMSNGDMFEKNHDEIDFEFLGNIRAKKWRIQTNIYGNGSTNVGREERYGLWFDPSEDFHQYSILWTESQIIFYVDNIPIREIKRTKAMGGDFPSKPMSLYATIWDGSSWATNGGKYKVNYKYAPYVAKFSDFILHGCAVDPIELSPKCDTTPNSASIPTSISPDQRRKMESFRKKYLQYSYCYDRTRYNVPLSECVIDPKEADRLRGFDPVTFGGVQRHHSKRHHQRQSRREDTSSE
